# Pharmacokinetics of Intramuscularly Administered Thermoresponsive Polymers

**DOI:** 10.1002/adhm.202201344

**Published:** 2022-11-07

**Authors:** Ondřej Groborz, Kristýna Kolouchová, Jan Pankrác, Peter Keša, Jan Kadlec, Tereza Krunclová, Aneta Pierzynová, Jaromír Šrámek, Mária Hovořáková, Linda Dalecká, Zuzana Pavlíková, Petr Matouš, Petr Páral, Lenka Loukotová, Pavel Švec, Hynek Beneš, Lubomír Štěpánek, David Dunlop, Carlos V. Melo, Luděk Šefc, Tomáš Slanina, Jiří Beneš, Sandra Van Vlierberghe, Richard Hoogenboom, Martin Hrubý

**Affiliations:** ^1^ Institute of Macromolecular Chemistry Czech Academy of Sciences Heyrovského náměstí 2, Prague 6 Prague 162 06 Czech Republic; ^2^ Institute of Organic Chemistry and Biochemistry Czech Academy of Sciences Flemingovo náměstí 542, Prague 6 Prague 160 00 Czech Republic; ^3^ Institute of Biophysics and Informatics Charles University First Faculty of Medicine Salmovská 1, Prague 2 Prague 120 00 Czech Republic; ^4^ Department of Organic and Macromolecular Chemistry Centre of Macromolecular Chemistry Ghent University Krijgslaan 281‐S4 Ghent 9000 Belgium; ^5^ Center for Advanced Preclinical Imaging (CAPI) First Faculty of Medicine Charles University Salmovská 3, Prague 2 Prague 120 00 Czech Republic; ^6^ FUJIFILM VisualSonics, Inc. Joop Geesinkweg 140 1114 AB Amsterdam The Netherlands; ^7^ Weizmann Institute of Science Department of Brain Sciences Rehovot 7610001 Israel; ^8^ Institute of Histology and Embryology First Faculty of Medicine Charles University Albertov 4, Prague 2 Prague 128 00 Czech Republic; ^9^ Department of Physical and Macromolecular Chemistry Faculty of Sciences Charles University Hlavova 8, Prague 2 Prague 128 00 Czech Republic

**Keywords:** biodistribution, LCST, poly(2,2‐difluoroethyl)acrylamide, poly(*N,N*‐diethylacrylamide), poly(*N*‐isopropylacrylamide), poly(*N*‐acryloylpyrolidine), polyacrylamide, rational polymer design

## Abstract

Aqueous solutions of some polymers exhibit a lower critical solution temperature (LCST); that is, they form phase‐separated aggregates when heated above a threshold temperature. Such polymers found many promising (bio)medical applications, including in situ thermogelling with controlled drug release, polymer‐supported radiotherapy (brachytherapy), immunotherapy, and wound dressing, among others. Yet, despite the extensive research on medicinal applications of thermoresponsive polymers, their biodistribution and fate after administration remained unknown. Thus, herein, they studied the pharmacokinetics of four different thermoresponsive polyacrylamides after intramuscular administration in mice. In vivo, these thermoresponsive polymers formed depots that subsequently dissolved with a two‐phase kinetics (depot maturation, slow redissolution) with half‐lives 2 weeks to 5 months, as depot vitrification prolonged their half‐lives. Additionally, the decrease of *T*_CP_ of a polymer solution increased the density of the intramuscular depot. Moreover, they detected secondary polymer depots in the kidneys and liver; these secondary depots also followed two‐phase kinetics (depot maturation and slow dissolution), with half‐lives 8 to 38 days (kidneys) and 15 to 22 days (liver). Overall, these findings may be used to tailor the properties of thermoresponsive polymers to meet the demands of their medicinal applications. Their methods may become a benchmark for future studies of polymer biodistribution.

## Graphical abstract

Herein, they investigate the pharmacokinetics and biodistribution of four different thermoresponsive polyacrylamides. After intramuscular administration, their polymers from muscle depots, which dissolve with biological half‐lives 2 to 20 weeks. Moreover, polymers form secondary depots in kidneys and livers with various pharmacokinetics. Finally, they correlated the pharmacokinetics of these polymers with their physico‐chemical properties toward rational polymer design for biomedical applications.

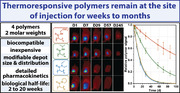

## 1 Introduction

Aqueous solutions of most hydrophilic polymers exhibit a lower critical solution temperature (LCST).^[^ ^1^ ^]^ In other words, when heated above a threshold temperature (cloud point temperature, *T*_CP_), solutions of these polymers reversibly phase‐separate into two phases, one with a low polymer concentration and the other, water‐insoluble, with a high polymer concentration (“hydrogel aggregates”).^[^ ^1^ ^]^ Thanks to this thermoswitchable behavior, these polymers have many in vitro and in vivo applications in medicine and biological research,^[^ ^2^ ^]^ including drug‐delivery systems,^[^ ^3^, ^4^, ^5^ ^]^ cell cultures,^[^ ^6^ ^]^ and, most importantly for this study, injectable thermogelling systems.^[^ ^7^, ^8^ ^]^

In injectable thermogelling, polymer solutions with a *T*_CP_ below body temperature (but above room temperature) are injected into the body at room temperature. Upon their administration, the temperature of these polymer solutions increases above their *T*_CP_, thereby in situ forming insoluble hydrogels, which remain at the site of administration.^[^ ^7^, ^9^ ^]^ The resulting soft hydrogel depots are highly compliant,^[^ ^10^, ^11^ ^]^ do not irritate the surrounding tissues,^[^ ^10^, ^11^, ^12^ ^]^ and can be used as carriers for local radiotherapy (brachytherapy),^[^ ^13^ ^]^ pharmacotherapy,^[^ ^14^, ^15^ ^]^ photodynamic therapy,^[^ ^16^ ^]^ immunotherapy,^[^ ^17^ ^]^ and vaccines.^[^ ^18^ ^]^ Furthermore, they can alter tissue growth, regeneration, and other properties,^[^ ^19^ ^]^ they can be applied in wound dressings^[^ ^20^, ^21^ ^]^ and healing,^[^ ^10^, ^21^ ^]^ as well as in tissue engineering,^[^ ^6^, ^21^, ^22^, ^23^, ^24^, ^25^, ^26^ ^]^ and they can be used as tracers for cell/ tissue tracking.^[^ ^12^ ^]^ When co‐administered with drugs/ hormones/ DNA/RNA, their polymer aggregates incorporate these compounds and release them for weeks, and even up to months, limiting side effects and prolonging the treatment while increasing their efficacy.^[^ ^7^, ^12^, ^15^, ^27^, ^28^, ^29^, ^30^ ^]^ One such thermoresponsive polymer, ReGel (OncoGel), has shown promising results in several preclinical and clinical trials.^[^ ^31^, ^32^, ^33^, ^34^, ^35^, ^36^ ^]^ Therefore, the use of thermoresponsive polymers as injectable slow‐release drug depots may bring major benefits over therapeutic approaches currently applied in medicine.

Such potential medicinal applications of thermoresponsive polymers may, however, be precluded by our limited knowledge of their fate and pharmacokinetics, particularly regarding aggregate elimination kinetics, rather than by their specific properties.^[^ ^12^, ^37^, ^38^ ^]^ For example, in our previous study on intramuscularly administered poly[*N*‐(isopropyl)acrylamide], we demonstrated that polymer content decreased quickly for the first few days but only slowly in the following weeks and months.^[^ ^37^ ^]^ Furthermore, intramuscularly administered polymers form secondary depots in other organs (most markedly in the liver and kidneys), which are subsequently eliminated from the body via urine and feces.^[^ ^37^, ^38^ ^]^ Our follow‐up study corroborated these results, suggested a two‐phase pharmacokinetics model, and showed that adding a non‐thermoresponsive comonomer enables fine‐tuning polymer dissolution kinetics.^[^ ^12^ ^]^ In summary, the in vivo analysis of thermoresponsive polymers for medicinal applications must include a comprehensive long‐term pharmacokinetics biodistribution study.

Long‐term pharmacokinetics studies require imaging techniques that do not involve ionizing radiation or irritate tissues, such as nuclear magnetic resonance imaging, especially fluorine‐19 magnetic resonance imaging (^19^F MRI).^[^ ^12^, ^39^, ^40^ ^]^ However, ^19^F MRI is only sensitive to polymers with a high fluorine content. Nevertheless, in vivo fluorescence (IVF) and photoacoustic (PAI) imaging have recently emerged as sensitive, non‐invasive imaging techniques for assessing the biodistribution and quantities of fluorescently labeled compounds over time.^[^ ^41^, ^42^ ^]^ These techniques are most effective when imaging compounds near the body surface of small laboratory animals because both skin and other tissues absorb a portion of the excitation and emission light.^[^ ^41^, ^42^ ^]^ The geometry of depots can also affect the signal, but both depot geometry and depth (and hence the light absorption/scattering factor) remain almost constant in long‐term depot dissolution studies. As a result, although the absolute signal in each depot may be distorted by tissue absorption, fluorescence imaging is a straightforward method for accurately assessing depot dissolution kinetics.

**Figure 1:**
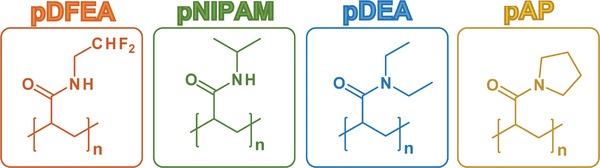
Chemical structures of our polymers: poly[(*N*‐2,2‐difluoroethyl)acrylamide] (**pDFEA**), poly[(*N*‐isopropyl)acrylamide] (**pNIPAM**), poly[(*N*,*N*‐diethyl)acrylamide] (**pDEA**), and poly[(*N*‐acryloyl)pyrrolidine] (**pAP**).

Considering the above, we conducted a comprehensive, long‐term in vivo biodistribution study of four thermoresponsive polyacrylamides, commonly used in biomedical research,^[^ ^4^ ^]^ namely poly[*N*‐(2,2‐difluoroethyl)acrylamide] (**pDFEA**), poly[*N*‐(isopropyl)acrylamide] (**pNIPAM**), poly[*N,N*‐(diethyl)acrylamide] (**pDEA**) and poly[(*N*‐acryloyl)pyrrolidine] (**pAP**; **Figure** **1**), with different structures but similar *T*_CP_ at similar polymer concentrations.^[^ ^1^, ^43^, ^44^, ^45^ ^]^ More specifically, these polymers met all of the following selection criteria: i) homopolymers of polyacrylamide derivatives (because the content of a co‐monomer^[^ ^12^ ^]^ and polymer architecture^[^ ^46^, ^47^, ^48^ ^]^ can alter their physico‐chemical properties and pharmacokinetics, adding more variables to the system); ii) water‐soluble polymers whose aqueous solutions show LCST‐type thermoresponsiveness with transition temperatures close to body temperature, but above room temperature (20 °C); iii) nonreactive, biologically inert,^[^ ^4^ ^]^ inexpensive, easy‐to‐prepare^[^ ^49^ ^]^ and biocompatible (as defined by ISO 10 993 norm for implants^[^ ^50^ ^]^) polymers whose structures represent all structural motives^[^ ^1^ ^]^ commonly found in thermoresponsive polymers (halogenated substituents, primary and secondary amides, linear and cyclic substituents). Furthermore, **pAP** was used as a control because its *T*_CP_ lies above body temperature, thus avoiding aggregation at the site of administration, in contrast to the other three polymers. To assess molar mass effects on polymer biodistribution and pharmacokinetics, each polymer was analyzed in two molar masses (≈20 kg mol^−1^ and ≈35 kg mol^−1^; Figure 1), both of which below the glomerular cut‐off (renal threshold) to prevent their accumulation in the body.^[^ ^38^, ^51^, ^52^, ^53^ ^]^ For this purpose, we labeled these polymers with a fluorescent dye, injected them into the thigh muscle of mice, and tracked their biodistribution. Lastly, we compared the biodistributions of Cy7‐traced polymers with the biodistribution of free **Cy7‐amine** as a control.

## 2 Results and Discussion

### 2.1 In Vitro Cellular Uptake of Polymers Varies with their *T*_CP_

**Figure 2:**
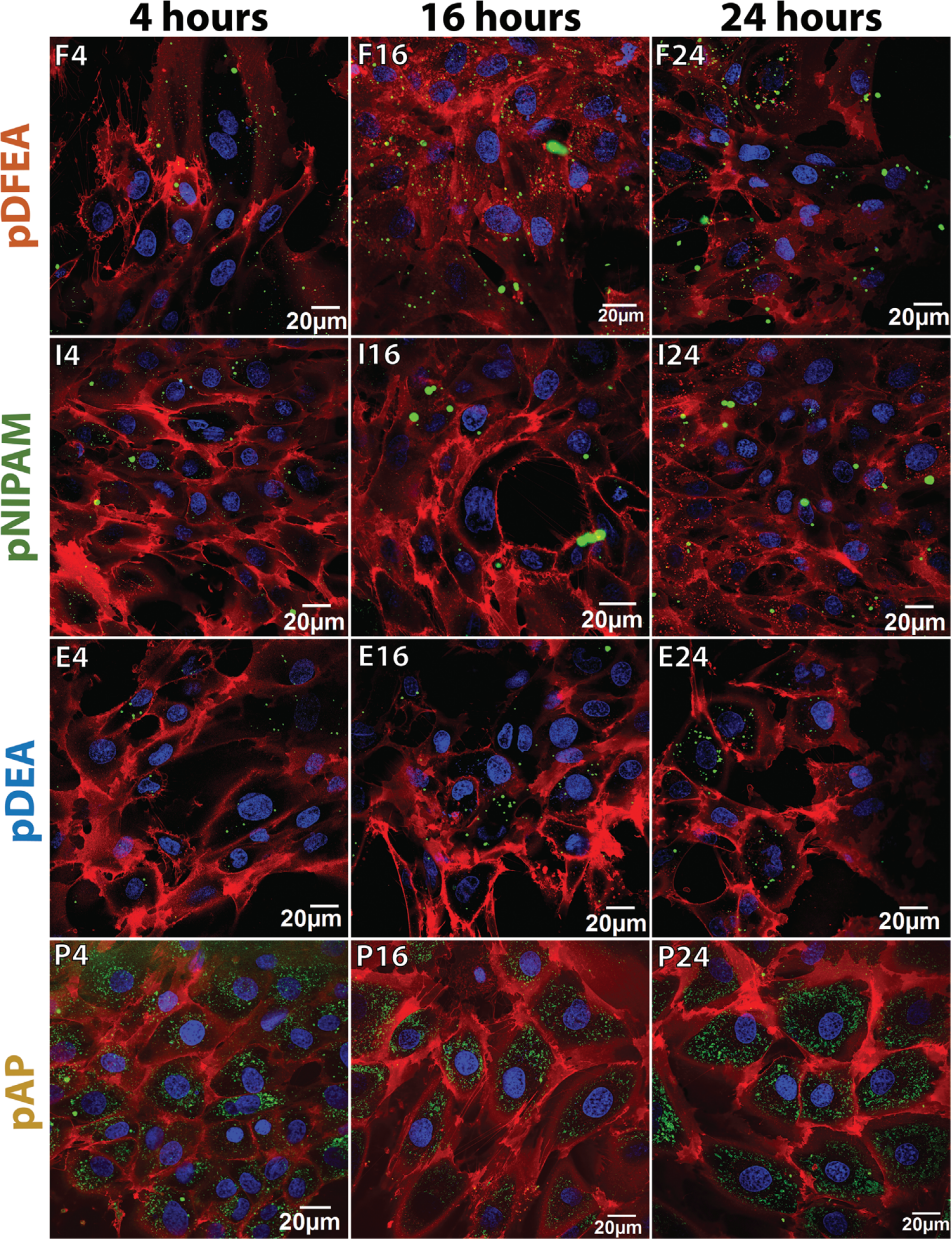
Cellular uptake of Dy‐505‐traced polymers into rMSC as a function of time; each polymer is shown in **green** (Dy505), cell membranes in **red** (CellMask Deep red), and nuclei in **blue** (Hoechst 33 342).

Before performing in vivo biodistribution studies, we analyzed cellular uptake and cellular localization using Dy505‐labelled **pDFEA**, **pNIPAM**, **pDEA**, and **pAP** (Section S3, Supporting Information). To differentiate the cells and their organelles, we stained the cytoplasmatic membranes, cell nuclei and lysosomes. In line with previous research,^[^ ^54^, ^55^ ^]^ all our polymers were located inside human fibroblasts (HF) and rat mesenchymal stem cells (rMSC). However, except for **pAP**, their cellular uptake was not uniform, with some cells showing strong polymer staining and others only negligible or no staining within one sample (**Figures** **2** and **S57**, Supporting Information). These differences in polymer uptake can be attributed to polymer aggregation because the cell culture is maintained at 37 °C. At this temperature, all polymers phase‐separated into dehydrated polymer mesoglobules (except for **pAP**, whose *T*_CP_ is approximately 50 °C, **Table** **1**), resulting in an uneven distribution across cells as some cells had limited contact with polymer aggregates. Conversely, **pAP**, remained soluble at 37 °C, so all cells exposed to **pAP** showed similar polymer uptake. Therefore, the polymers were internalized in both rMSC (Figure 2) and HF (Figure S57, Supporting Information), but cellular uptake depended on the state of the polymers (aggregates or solution) and on the surface area of contact with the polymers.^[^ ^56^ ^]^

**Table 1:** Number‐average molar mass (*M*_n_), dispersity index (*Đ*_M_) and cloud point temperature (*T*_CP_) of our polymers in fetal bovine serum at *c*_pol_ = 10.0 mg mL^−1^ and 1.25 mg mL^−1^,^[^ ^43^ ^]^ and glass‐point temperatures (*T*_g_) of neat polymers. Temperatures above body temperature (37 °C) are highlighted in red

		*M*_n_	*Đ*_M_	*T*_CP, 10.0 mg mL_^−1^	*T*_CP, 1.25 mg mL_^−1^	*T*_g_
Polymer		(kg mol^−1^)	(*M*_w_/M_n_)	(°C)	(°C)	(°C)
pDFEA	F1	24.2	1.08	22.6 ± 0.1	33.9 ± 0.1	113
	F2	35.1	1.03	30.1 ± 0.2	48.0 ± 0.2	
pNIPAM	I1	19.6	1.03	25.7 ± 0.1	33.6 ± 0.1	136
	I2	30.8	1.03	24.4 ± 0.1	30.9 ± 0.0	
pDEA	E1	21.2	1.06	24.4 ± 0.1	35.7 ± 0.3	95
	E2	31.7	1.09	25.8 ± 0.2	32.6 ± 0.2	
pAP	P1	17.6	1.11	56.7 ± 0.3	75.0 ± 0.8	144
	P2	32.9	1.09	48.6 ± 0.1	51.2 ± 0.1	

The intracellular distribution of the polymers varied considerably. On the one hand, we detected **pAP** in small pinocytotic vesicles^[^ ^57^, ^58^, ^59^ ^]^ (0.1 to 1 µm) and in lysosomes (Figure S57, Supporting Information). On the other hand, in line with previous research,^[^ ^54^, ^55^ ^]^ the remaining polymers were found in large phagosomes^[^ ^57^, ^58^, ^59^ ^]^ (0.5 to 10 µm) and lysosomes. These differences in intracellular distribution may also be explained by polymer aggregation: **pAP** remains soluble at incubation temperatures and can be internalized by pinocytosis (forming many small polymer‐loaded vesicles), whereas the remaining polymers that are collapsed at 37 °C can only be internalized by phagocytosis (forming few large vesicles). Nevertheless, we demonstrated that all four studied polymers are internalized into cells, albeit through different mechanisms.

Previous research has revealed that phagocyted material function as reservoirs of small molecules that slowly release their content into the cytosol and surrounding cells.^[^ ^56^ ^]^ Additionally, phagocyted polymer aggregates can serve as drug‐, gene‐ or enzyme‐delivery systems to lysosomes for the treatment or modulation of various lysosomal storage diseases.^[^ ^60^, ^61^ ^]^ Thus, these polymers may be used in intracellular drug or enzyme delivery.

### 2.2 Increasing *T*_CP_ Decreases the Density of Intramuscular Depots

**Figure 3:**
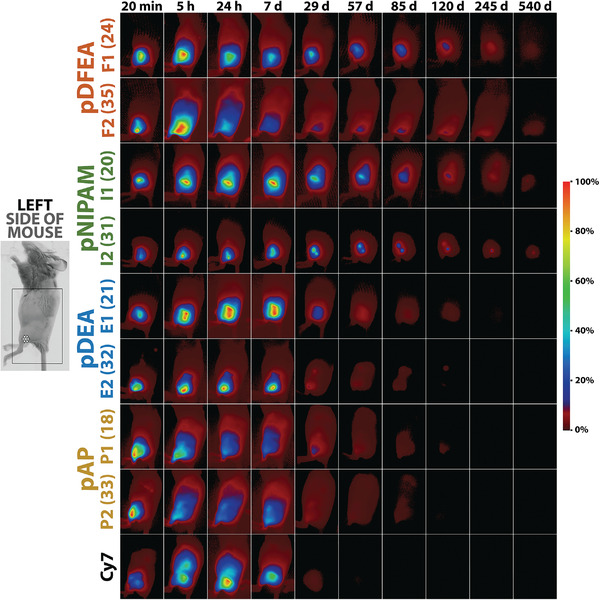
Biodistribution of our polymers (administered polymer concentration 0.10 mg µL^−1^; volume 5.00 µL) and Cy7‐amine on the **left** side of mice at various timepoints after IM administration; the intensity of each polymer is normalized to its maximum; left: field of view; an asterisk (*) indicates the approximate site of injection; middle: biodistribution of our polymers with various *M*_n_ (in brackets; kg mol^−1^); right: fluorescence intensity scale. The images show that primary depot diffuseness depends on *T*_CP_ at low polymer concentrations because polymers with a higher *T*_CP_ show an increased formation of primary intramuscular depots, with non‐aggregating **pAP** forming diffuse intramuscular depots regardless of molar mass. By contrast, polymers with *T*_CP_ well below body temperature (**pNIPAM** and **pDEA**) formed dense depots whose density increased with the molar mass. Lastly, **pDFEA**, whose *T*_CP_ is near body temperature at low polymer concentration, formed intermediately diffuse depots.

After their injection into mice, **pDFEA** (**F1** and **F2**), **pNIPAM** (**I1** and **I2**), **pDEA** (**E1** and **E2**), and **pAP** (**P1** and **P2**) formed intramuscular depots with various depot densities (**Figure** **3**, depot density was described using parameter *K*_10_, Section S5.6, Supporting Information). Initially (≈5 min after administration), all depots had similar volumes (Tables S43 to S52, Supporting Information), but they subsequently expanded due to polymer diffusion over 2 to 4 days. During this period, the polymers were partly eliminated into the bloodstream and absorbed by local cells (as shown in vitro in Section S9, Supporting Information, and ex vivo in Section S10, Supporting Information), eventually preventing further depot expansion. The polymers that were soluble at 37 °C (**P1**, **P2**, and **F2**,^[^ ^62^ ^]^ *T*_CP_ > 37 °C, Figure 1) formed diffuse depots over an entire side of the mice. By contrast, the polymers that collapsed at 37°C (**F1**, **I1**, **I2**, **E1**, **E2**; *T*_CP_ < 37 °C) formed dense polymer depots, whose volumes increased only slightly because they undergo only limited diffusion (Figure 3).

Higher‐molar‐mass **pNIPAM** and **pDEA** exhibited lower *T*_CP_ than the corresponding lower‐molar‐mass polymers. This decrease of *T*_CP_ accounted for their more extensive dehydration and collapse at body temperature, even at low polymer concentrations (Table 1), thus increasing the densities of their depots. Conversely, higher‐molar‐mass **pDFEA** ^[^ ^62^ ^]^ had a higher *T*_CP_ than its lower‐molar‐mass counterpart (Table 1), accordingly leading to lower depot density. Yet, the increased molar mass of soluble **pAP** had only a minor effect on the density of its intramuscular depots because both lower‐ and higher‐molar mass **pAP** did not aggregate at body temperature (Figure 1) and were thus free to diffuse through tissues. As assessed by a thorough histopathological examination (Sections S6 and S15, Supporting Information), all polymers showed in vivo biocompatibility in mice upon long‐term administration, causing no harm to the animals. Therefore, these polymers may be considered for human medicinal applications by selecting the corresponding molar masses and *T*_CP_ (both at high and low polymer concentrations) that meet the specific demands of depot diffuseness of various local applications (brachytherapy and drug release, among others).

### 2.3 Polymer Collapse Prevents Signal Migration to Remote Organs

**Figure 4:**
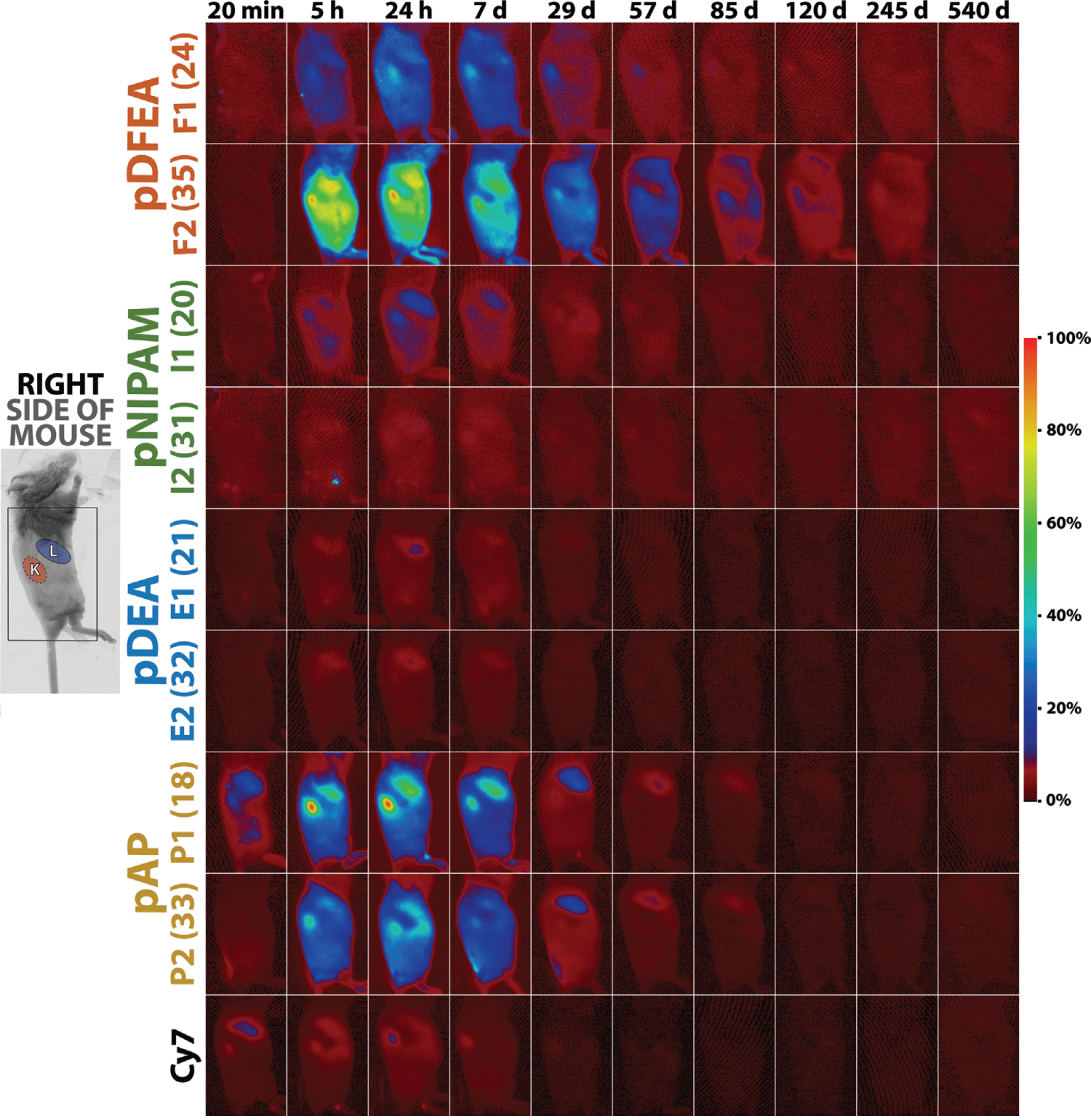
Biodistribution of our polymers (administered polymer concentration 0.10 mg µL^−1^; volume 5.00 µL) or Cy7‐amine on the **right** side of mice (opposite the site of administration) at various timepoints after their intramuscular administration; the signal was normalized to absolute maxima of all polymers (normalization to maxima of each polymer is depicted in Figure S62, Supporting Information);^[^ ^72^ ^]^ left: field of view with the positions of the kidney (**K**) and liver (**L**) areas; middle: biodistribution of our polymers with various *M*_n_ (in kg per mol); right: fluorescence intensity scale.

Shortly after injecting the polymer intramuscularly, we detected polymer signal on the right side of the mice (opposite to the injected muscle; **Figure** **4** and Figure S62, Supporting Information), primarily in the liver and kidneys. These results are in line with the findings of previous studies^[^ ^37^, ^38^, ^63^, ^64^, ^65^, ^66^ ^]^ because nanoparticles and polymers can be absorbed by the liver (predominantly by Kupffer cells^[^ ^64^, ^65^, ^66^ ^]^) and kidneys (intraglomerular mesangial cells^[^ ^67^, ^68^, ^69^, ^70^, ^71^ ^]^).

The intensity of the polymer signal in these secondary depots increased shortly after injecting the polymer, followed by a slow decrease over the following weeks, as discussed in more detail in Section 2.4, Supporting Information. The initial increase in the signal of secondary depots may be attributed to the initial fast release of polymers from the muscle tissue to the bloodstream and to polymer absorption by cells in remote organs (liver and kidneys).

The signal intensity of secondary depots was relatively high for **P1**, **P2**, and **F2** ^[^ ^62^ ^]^ but only minor for **F1**, **I1**, **I2**, **E1**, and **E2** (Figure 4 and Table S89, Supporting Information). The thermoresponsiveness of these different polymers may explain their differences in secondary depot formation, that is, polymers that show no (**P1** and **P2**) or only limited (**F2**) aggregation are partly eliminated from primary depots into the bloodstream before being absorbed by local cells. This blood‐dissolved polymer is then partly eliminated and absorbed by the liver and kidneys, thus forming intense secondary depots in these organs. On the contrary, the thermoresponsive polymers **I1**, **I2**, **E1**, **E2**, and **F1** that collapsed at 37°C remained in the administration site, and only a minor portion of administered polymer entered the bloodstream, limiting the formation of secondary depots in remote organs. Moreover, higher‐molar‐mass **pNIPAM** and **pDEA** had a lower *T*_CP_ and aggregated more extensively; consequently, their signal was weaker in secondary organ depots. Conversely, high‐molar‐mass **pDFEA** had a higher *T*_CP_ (Table 1) and thus a stronger signal in remote organs. These results demonstrate that polymers can be selected based on their *T*_CP_, which determines the fraction that is re‐localized in remote organs, towards limiting secondary depot formation and possible side effects of their biological applications.

### 2.4 Polymer Vitrification Slows Down Depot Dissolution

After the initial polymer redistribution, the signal from primary muscle depot and secondary liver and kidney depots decreased in the following weeks to months, with first‐order kinetics (**Table** **2**, Figures S4 and S5, Supporting Information).

**Table 2:** The signal intensity‐based half‐lives of primary intramuscular ($t_{1/2,I,\mathrm{IM}}$; 2 weeks for **pAP** and **pDEA**, 2 months for **pNIPAM** and 5 months for **pDFEA**) and secondary liver ($t_{1/2,I,\mathrm{LIV}}$; 14 to 22 days for all polymers) and kidney ($t_{1/2,I,\mathrm{KID}}$; 9 days for **pAP**, 17 to 24 days for **pDEA** and **pNIPAM**, and 38 days for **pDFEA**) depots of polymers varied considerably. A double dagger (^‡^) mark indicates distorted data caused by an excessively intense and delocalized signal. The half‐lives of depot primary and secondary dissolution do not correlate with the *T*_CP_ or molar mass of their polymers

Polym	Subtype	Muscle	Liver	Kidney
	*M*_w_	$t_{1/2,I,\mathrm{IM}}$	$t_{1/2,I,\mathrm{LIV}}$	$t_{1/2,I,\mathrm{KID}}$
	(kg mol^–1^)	(d)	(d)	(d)
**pDFEA**	**F1 (26)**	144 ± 14	20.3 ± 1.8	45.3 ± 4.1
	**F2 (36)**	145 ± 16	^‡^	^‡^
**pNIPAM**	**I1 (20)**	48.4 ± 2.6	14.4 ± 2.1	14.6 ± 0.9
	**I2 (32)**	65.8 ± 3.8	18.6 ± 2.0	24.4 ± 2.8
**pDEA**	**E1 (22)**	15.9 ± 1.1	17.8 ± 2.4	16.8 ± 1.2
	**E2 (35)**	16.3 ± 1.2	15.4 ± 2.0	17.9 ± 0.4
**pAP**	**P1 (20)**	17.3 ± 0.9	22.2 ± 0.9	8.0 ± 0.4
	**P2 (36)**	16.3 ± 1.2	21.1 ± 0.9	8.8 ± 0.6
**Cy7**		4.8 ± 0.3	10.9 ± 1.5	8.6 ± 0.4

The intramuscular depots of the polymers differed not only in their diffuseness but also in their dissolution rates (Table 2): **pDFEA** and **pNIPAM** had significantly longer half‐lives than **pDEA** and **pAP**. Surprisingly, the biological half‐lives of the polymers in muscle depots were statistically independent of their *T*_CP_ and molar masses as **pDEA** and **pAP** have similar half‐lives, but vastly different *T*_CP_ (Table 1 and Section S14, Supporting Information). Accordingly, **pDFEA**, **pNIPAM**, and **pDEA**, which have similar *T*_CP_ values, showed considerably different biological half‐lives in muscle depots.

We hypothesize that such a diversity of biological half‐lives can be attributed to polymer vitrification. Previous studies^[^ ^73^, ^74^, ^75^ ^]^ have demonstrated that **pNIPAM** aggregates can vitrify over time, which affects the demixing and remixing kinetics of this polymer. To test this hypothesis, we studied the thermal properties of phase‐separated polymers by differential scanning calorimetry (DSC) in their 20 wt. % solutions (Section S8.3, Supporting Information). We observed signs of aggregate vitrification in **pDFEA** and **pNIPAM** at 37 °C (also in **pAP** but only above ≈50 °C), whereas **pDEA** did not form a vitrified collapsed phase. Therefore, we propose that both **pDFEA** and **pNIPAM** aggregated and vitrified inside cells, which decreased their elimination rates in comparison with **pDEA** and **pAP**.

By contrast, **pAP** (non‐aggregated polymer at 37 °C) and **pDEA** (aggregated but not vitrified) had similar dissolution rates, both of which were significantly faster than the dissolution rates of **pNIPAM** and **pDFEA**. In this context, we have previously demonstrated that **pDFEA** aggregates exhibit extraordinarily strong interactions with proteins,^[^ ^43^ ^]^ which may further decrease the rate of in vivo elimination of this polymer from cells (Section S14, Supporting Information). As such, any rational design of polymers for biological applications must consider not only their depot diffuseness but also their biological half‐lives.

In line with the above, the signal of polymers with various biological half‐lives in the secondary liver and kidney depots also decreased with first‐order kinetics (Table 2). Previous research has shown that, upon cell death, the content of cells is released into urine (mesangial cells) or bile and stool (Kupffer cells) or re‐absorbed by other cells.^[^ ^64^, ^69^, ^70^, ^76^, ^77^ ^]^ In the liver depots, the biological half‐lives of all polymers (15 to 22 days, Table 2) were similar to the lifespan of Kupffer cells (≈ 21 days^[^ ^78^ ^]^). This observation suggests that the half‐lives of these polymers in the liver are determined by the lifespan of Kupffer cells rather than by their physico‐chemical properties (further discussed in Section S14, Supporting Information).

In the kidney depots, in turn, the half‐lives of the polymers varied significantly, ranging from 8 to 38 days, revealing a similar trend to that observed in intramuscular depot dissolutions (**pAP** < **pDEA** ≈ **pNIPAM** < **pDFEA**; Table 2, further discussed in Section S14, Supporting Information). Hence, in kidneys, the physico‐chemical properties of these polymers determine both their half‐lives and their elimination into urine by mesangial cells; their clearance is clearly not affected by the lifespan of these cells (≈ several weeks^[^ ^79^ ^]^). Combined, these findings highlight the importance of analyzing the formation of secondary organ depots when designing polymers with various half‐lives for medicinal applications.

### 2.5 Physiological Model of Polymer Pharmacokinetics

**Figure 5:**
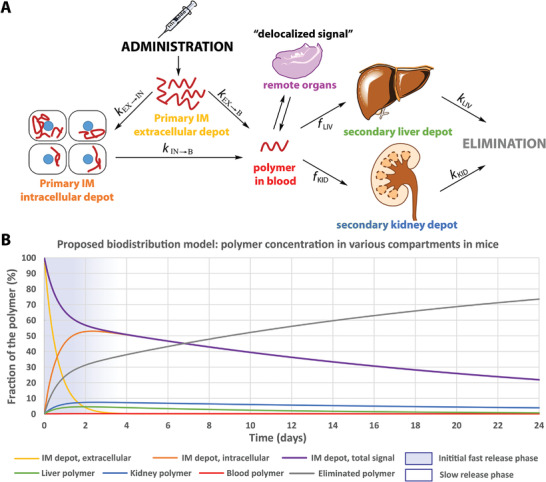
Proposed biodistribution model (**A**) and plot of calculated concentrations in each compartment (**B**), demonstrated by the biodistribution of a model polymer as a function of time.

Based on the data presented above and on our previous results,^[^ ^12^ ^]^ showing that thermoresponsive polymers bind to tissues, which slows down their dissolution kinetics, we propose here a pharmacokinetics model that describes the biodistribution of polymers in the body (**Figure** **5** and Section S7, Supporting Information). After their administration, the polymers form primary extracellular depots, which slowly enlarge due to diffusion, for a few days (*t*_*S*, max_), as a function of *T*_CP_. During this period, a fraction of the polymers (*A*) are absorbed into muscle cells (*k*_EX→IN_), forming primary intracellular depots, whereas the remaining polymers (1 – *A*) are drained via lymphatic and blood vessels into the bloodstream (*k*_EX→B_), where *A* is determined by the ratio of *k*_EX→IN_ and sum of *k*_EX→IN_ and *k*_EX→B_ (because *k*_EX→IN_ and *k*_EX→B_ are competing processes). The primary intracellular depots slowly redissolve, releasing the polymers into the bloodstream (most likely via exocytosis^[^ ^59^ ^]^) for weeks and up to months (*k*_*I*,IM_ or $t_{1/2,I,\mathrm{IM}}$). Through the blood, these polymers enter the liver and kidneys (and possibly, to a small extent, other organs), forming secondary depots therein (with variable affinities, *i*.*e*., initial signal fraction in kidneys, *f*_KID_, and liver, *f*_LIV_). Ultimately, the polymers are released from the kidneys into the urine and from the liver into the bile, thus being excreted (*k*_KID_ and *k*_LIV_). Overall, these processes can be characterized by specific kinetics constants and tuned by choosing polymers (or comonomers^[^ ^12^ ^]^) that meet the requirements of a desired application.

This model is in line with previous studies, which demonstrated that such polymers can i) enter the bloodstream after intramuscular,^[^ ^37^, ^80^ ^]^ intraperitoneal,^[^ ^63^, ^80^ ^]^ or subcutaneous^[^ ^63^, ^80^ ^]^ administration, ii) form primary depots at the site of administration and secondary depots predominantly in the liver,^[^ ^37^, ^38^, ^51^, ^63^, ^80^, ^81^, ^82^ ^]^ kidneys,^[^ ^37^, ^51^, ^81^, ^82^ ^]^ spleen,^[^ ^38^, ^51^ ^]^ among other organs, and iii) are ultimately eliminated via urine and stool (thus signal was detected in bladder and intestines).^[^ ^37^, ^38^, ^63^, ^81^ ^]^ Furthermore, the composition (co‐monomer content),^[^ ^12^, ^38^, ^51^, ^80^ ^]^ molar mass,^[^ ^38^, ^51^, ^63^, ^80^, ^81^, ^82^ ^]^ and aggregation^[^ ^12^, ^37^ ^]^ of the polymers affect their pharmacokinetics and biodistribution.

### 2.6 Rational Polymer Design

Lastly, we thoroughly analyzed how the properties of the polymers affect their pharmacokinetics (Section S14, Supporting Information). Our findings can be summarized into three simple rules for rational polymer design:

i) **At the site of administration, polymer depot diffuseness increases with *T*_CP_**. Polymers with a *T*_CP_^[^ ^83^ ^]^ lower than body temperature form dense primary depots at the site of administration, whereas polymers with a *T*_CP_ higher than body temperature form diffuse depots. In turn, the *T*_CP_ of a polymer can be fine‐tuned by changing its molar mass or its content of comonomers.^[^ ^12^ ^]^
ii) **In remote organs, the polymer content of secondary depots increases with *T*_CP_**. Polymers with a *T*_CP_ higher than body temperature accumulate in remote organs. The polymer content of these secondary depots increases with *T*_CP_. By contrast, polymers with a *T*_CP_ lower than body temperature are barely detectable in remote organs.
iii) **Polymer retention is limited by the lifespan of short‐lived cells but depends only on its physicochemical properties in long‐lived cells**. In long‐lived cells (such as myocytes), polymer retention increases with (a) the vitrification of polymer aggregates and (b) the affinity of polymer aggregates to proteins (both can be studied by calorimetry). Conversely, polymers that (a) do not aggregate or (b) aggregate but do not vitrify at body temperature have similar pharmacokinetics of dissolution (half‐life of ≈16 days). In some cells (mesangial cells), polymer retention mostly depends on its *T*_CP_ (Section S14, Supporting Information ). In contrast – in short‐lived cells, e.g., Kupfer cells, polymer retention is determined by the lifespan of these cells.

In conclusion, thermoresponsive polymers may have major advantages over non‐thermoresponsive polymers. Thermoresponsive polymers aggregate upon administration, effectively decreasing the dilution of the primary extracellular depot and thus the amount of polymer that is cleared into the bloodstream and that reaches the kidneys and liver (Figure 4 and Table S26, Supporting Information). Thanks to their increased retention at the site of administration, thermoresponsive polymers may considerably outperform non‐thermoresponsive polymers in some clinical applications by avoiding possible kidney‐ or liver‐related side effects, among other reasons.

## 3 Conclusions

In vitro, **pDFEA**, **pNIPAM**, **pDEA**, and **pAP** cellular uptake varies with *T*_CP_, and the mechanism of internalization of the polymers depends on their state (collapsed aggregates or solution) and on their surface area. In vivo, upon intramuscular administration, these polymers form depots whose density increases with dehydration and collapse, leading to a decrease in polymer diffusion rates. As such, polymers can be selected based on their *T*_CP_, which in turn can be fine‐tuned based on their molar mass, to meet the specific demands of depot diffuseness for local applications. Moreover, polymer thermoresponsiveness also determines the fraction of polymers localized in remote organs by secondary depot formation. Accordingly, their thermoresponsiveness can be used to limit or avoid possible side effects on those organs.

The intracellular depot dissolution of **pDFEA**, **pNIPAM**, **pDEA**, and **pAP** follows first‐order kinetics, albeit with different biological half‐lives. In muscles, the biological half‐lives of these polymers are highly variable (2 weeks to 5 months) and determined by their vitrification (if the *T*_g_ of the partly hydrated collapsed depot is lower than body temperature) and affinity to intracellular proteins. In the liver, their half‐lives are determined by the lifespan of Kupffer cells rather than by their physico‐chemical properties. In the kidneys, conversely, their half‐lives are determined by their physico‐chemical properties (inversely correlated with polymer *T*_CP_) rather than by the lifespan of mesangial cells. Their dissolution rates can be characterized by specific kinetics constants, which enable us to choose the polymers that meet the requirements of a desired clinical application. Therefore, thermoresponsive polymers have considerable advantages over non‐thermoresponsive polymers, e.g., by providing us with the ability to tune depot densities and half‐lives and to limit side effects on kidneys and liver.

Lastly, the biocompatible fluorescent tracer Cy7 can be used for long‐term (≥250 days) in vivo polymer tracking by fluorescence imaging. In pharmacokinetics research, this approach may be applied to other polymers for determining their biodistribution and biological half‐lives. Ultimately, the physiological biodistribution model proposed herein may become a benchmark for future studies of polymer pharmacokinetics and for the rational design of polymers for brachytherapy, drug delivery, and tissue engineering, among other applications.

## Conflict of Interest

O.G. is a stockholder of Bausch Health Companies Inc. (Laval, Canada), whose subsidiary licenses Visidic gel, which in turn was used in this study to prevent major side effects of anesthesia, such as keratitis sicca. The other authors have no conflict of interest to declare. No company, grant agency, or employer influenced the design of the study, its evaluation, or its conclusions.

## Supporting information

Supporting Information

Supplemental Video 1

Supplemental Video 2

Supplemental Video 3

Supplemental Video 4

Supplemental Video 5

Supplemental Video 6

Supplemental Video 7

Supplemental Video 8

Supplemental Video 9

Supplemental Video 10

Supplemental Video 11

Supplemental Video 12

Supplemental Video 13

Supplemental Video 14

Supplemental Video 15

Supplemental Video 16

Supplemental Video 17

Supplemental Video 18

Supplemental Video 19

Supporting Information

Supporting Information

Supporting Information

Supporting Information

Supporting Information

Supporting Information

Supporting Information

Supporting Information

Supporting Information

Supporting Information

## Data Availability

The data that support the findings of this study are available from the corresponding author upon reasonable request.
