# Supplementary material for: Pharmacokinetics of Intramuscularly Administered Thermoresponsive Polymers
Source: Adv Healthc Mater. 2022 Nov 7;11(22):2201344. doi: 10.1002/adhm.202201344 (PMC11468617; doi:10.1002/adhm.202201344)
Supplement: Supplementary file 1 — Supporting Information [file ADHM-11-2201344-s021.pdf]

ADVANCED HEALTHCARE MATERIALS

Supporting Information

for *Adv. Healthcare Mater.*, DOI 10.1002/adhm.202201344

Pharmacokinetics of Intramuscularly Administered Thermoresponsive Polymers

Ondřej Groborz, Kristýna Kolouchová, Jan Pankrác, Peter Keša, Jan Kadlec, Tereza Krunclová, Aneta Pierzynová, Jaromír Šrámek, Mária Hovořáková, Linda Dalecká, Zuzana Pavlíková, Petr Matouš, Petr Páral, Lenka Loukotová, Pavel Švec, Hynek Beneš, Lubomír Štěpánek, David Dunlop, Carlos V. Melo, Luděk Šefc, Tomáš Slanina, Jiří Beneš, Sandra Van Vlierberghe, Richard Hoogenboom* and Martin Hrubý**

ELECTRONIC SUPPORTING INFORMATION

OF THE ARTICLE

PHARMACOKINETICS OF INTRAMUSCULARLY ADMINISTERED THERMORESPONSIVE POLYMERS

Ondřej Groborz,[1,2,3,]* Kristýna Kolouchová,[1,4] Jan Pankrác,[5] Peter Keša,[5] Jan Kadlec,[6] Tereza Krunclová,[1] Aneta Pierzynová,[7] Jaromír Šrámek,[7] Mária Hovořáková,[7] Linda Dalecká,[7] Zuzana Pavlíková,[7] Petr Matouš,[5] Petr Páral,[5] Lenka Loukotová,[2] Pavel Švec,[2] Hynek Beneš,[1] Lubomír Štěpánek,[3] David Dunlop,[2] Carlos V. Melo,[8] Luděk Šefc,[5] Tomáš Slanina,[2] Jiří Beneš,[3] Sandra Van Vlierberghe,[4] Richard Hoogenboom,[4,]* and Martin Hrubý[1,]*

[1]Institute of Macromolecular Chemistry, Czech Academy of Sciences,
Heyrovského sq. 2, Prague 6, 162 06, Czech Republic
[2]Institute of Organic Chemistry and Biochemistry, Czech Academy of Sciences,
Flemingovo sq. 542, Prague 6, 160 00, Czech Republic
[3]Institute of Biophysics and Informatics, Charles University, First Faculty of Medicine,
Salmovská 1, 120 00 Prague 2, Czech Republic
[4]Department of Organic and Macromolecular Chemistry, Centre of Macromolecular Chemistry, Ghent University,
Krijgslaan 281-S4, 9000 Ghent, Belgium
[5]Center for Advanced Preclinical Imaging (CAPI), First Faculty of Medicine, Charles University,
Salmovská 3, Prague 2, 120 00, Czech Republic
[6]Weizmann Institute of Science, Department of Brain Sciences, Rehovot 7610001, Israel
[7]Institute of Histology and Embryology, First Faculty of Medicine, Charles University,
Albertov 4, Prague 2, 128 00, Czech Republic
[8]Department of Physical and Macromolecular Chemistry, Faculty of Sciences, Charles University,
Hlavova 8, Prague 2, 128 00, Czech Republic

*Corresponding authors:
ondrej.groborz@seznam.cz (Ondřej Groborz),
richard.hoogenboom@ugent.be (Richard Hoogenboom),
mhruby@centrum.cz (Martin Hrubý)

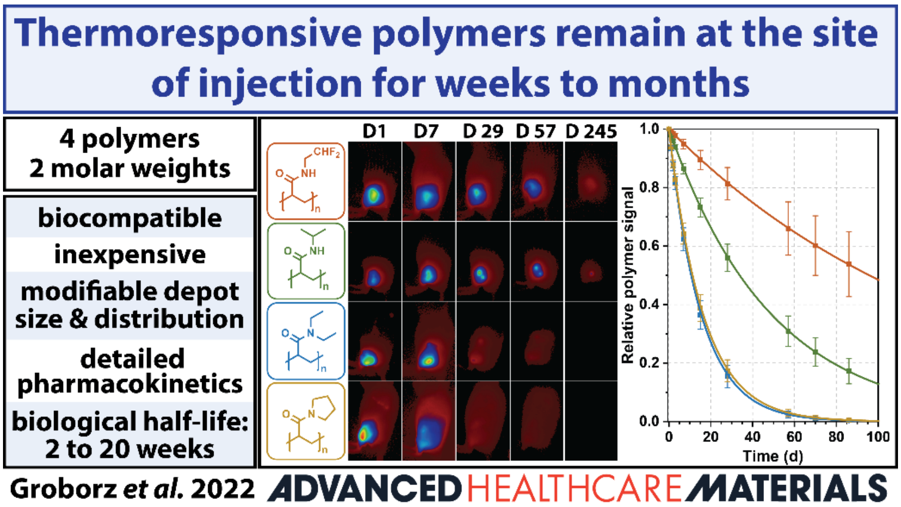

TABLE OF CONTENT

MATERIALS SECTION **5**

S1. Materials **5**
S1.1. Chemicals for synthesis – vendors, purifications, and storage 5
S1.2. Chemicals for *in vitro* cellular experiments 5
S1.3. Cell cultures 5
S1.4. Chemicals for histological examinations 5
S1.5. Animals and materials for *in vivo* experiments 6

EXPERIMENTAL SECTION **7**

S2. Polymer preparation and characterization **7**
S2.1. Synthesis and characterization of polymers 7
S2.1.1. Polymer synthesis 7
S2.1.2. Polymer characterization – nuclear magnetic resonance (NMR) 8
S2.1.3. Polymer characterization – size exclusion chromatography (SEC) 8
S2.2. Differential scanning calorimetry (DSC) 9
S2.2.1. Pure (bulk) polymers 9
S2.2.2. Aqueous solutions of polymers 9
S2.3. Conjugation of polymers with fluorescent labels 9
S2.3.1. Polymer conjugation (synthesis) 9
S2.3.2. Purity of Cy7-amine labelled polymers 11
S2.3.3. Amount of Cy7 in labelled polymers 11
S2.3.4. Chemical Stability of Cy7 amine label 11
S2.3.5. Fluorescence characteristics of Cy7-labelled polymers 11
S2.3.6. Purity of Dy505-amine labelled polymers 11
S2.3.7. Amount of Dy505 in labelled polymers 11
S3. *In vitro* biological experiments **13**
S3.1. Cytotoxicity assay 13
S3.2. Intracellular uptake and colocalization study 13
S3.2.1. Cell culture preparation and staining 13
S3.2.2. Microscopy details 14
S4. *In vivo* biological experiments **15**
S4.1. *In vivo* study design 15
S4.2. General preparation 16
S4.3. Ultrasound-photoacoustic imaging 16
S4.3.1. Instrument setup 16
S4.3.2. Experiment setup 17
S4.3.3. Data evaluation 17
S4.4. Polymer administration 17
S4.5. Long-term *in vivo* optical fluorescence imaging (Xtreme) 18
S4.5.1. Instrument setup 18
S4.5.2. Experiment setup 18
S4.6. Measurement of biological parameters of mice 18
S4.6.1. Instrumental and experimental setup 18
S4.7. *Ex vivo* experiments 19

S5. Biodistribution data processing **20**
S5.1. Study parameters 20
S5.2. Phases of signal 20
S5.2.1. Signal on right side of mice 21
S5.2.2. Signal on left side of mice 22
S5.3. Evaluation of extensive parameters 23
S5.3.1. Signal intensity in primary depot (I_{IM}) 23
S5.3.2. Primary depot size (S_{IM}) 23
S5.3.3. Depot volume (V_{IM}) 24
S5.3.4. Signal intensity in primary depot (I_{KID}) 25
S5.3.5. Signal intensity in primary depot (I_{LIV}) 25
S5.4. Determination of auxiliary parameters 26
S5.4.1. Noise threshold 26
S5.4.2. Additional auxiliary parameters 26
S5.5. Biological half-lives and statistical evaluations 27
S5.5.1. Dissolution kinetics 27
S5.5.2. Dissolution kinetics 27
S5.5.3. Kidney and liver accumulation factors (f_{KID} and f_{LIV}) 28
S5.6. Evaluation of depot density (K_{10}) 28
S5.7. Data processing 30
S6. Histopathological examination **31**
S6.1. Method 31
S7. Physiological model **33**
S7.1. Presumptions 33
S7.2. Model description 33
S7.3. Applied model 34

RESULTS & RAW DATA SECTION **36**

S8. Polymer characterization **36**
S8.1. Polymer characterization – NMR spectra of polymers 36
S8.2. Polymer characterization – size exclusion chromatography (SEC) 40
S8.3. Differential scanning calorimetry – results 43
S8.3.1. Glass point temperature (T_g) of pure (bulk) materials 44
S8.3.2. Cloud point temperature (T_{CP}) of aqueous solutions of polymers 47
S8.4. Purity of Cy7 labelled polymers & Cy7 quantification 52
S8.5. Purity of Dy505 labelled polymers & Dy505 quantification 56
S8.5.1. Fluorescence characteristics of Cy7-labelled polymers 59
S9. In vitro cellular assays **64**
S9.1. *In vitro* cytotoxicity 64
S9.2. Cellular uptake 67
S9.3. Cellular uptake assay – polymer-lysosome colocalization 68
S10. *Ex vivo* experiments – results overview **69**
S11. Polymer biodistribution **72**
S12. Polymer biodistribution & dissolution kinetics **74**
S12.1. Polymer pharmacokinetics - raw data 74
S12.1.1. Intramuscular depot dissolution data – signal intensity (I_{IM}) 74
S12.1.2. Intramuscular depot dissolution data – depot area (S_{IM}) 83

S12.1.3. Intramuscular depot dissolution data – depot volume (V_{IM}) 92
S12.1.4. Intramuscular depot dissolution data – depot distribution index (K_{10}) 96
S12.1.5. Kidney depot dissolution 105
S12.1.6. Liver depot dissolution 110
S12.2. Polymer pharmacokinetics - fitting curves 115
S12.2.1. Intramuscular depot dissolution data – signal (I_{IM}) 115
S12.2.2. Depot dissolution data – depot area (S_{IM}) 124
S12.2.3. Intramuscular depot dissolution data – distribution index (K_{10}) 133
S12.2.4. Kidney depot dissolution – signal as a function of time 142
S12.2.5. Liver depot dissolution – signal as a function of time 151
S13. Polymer pharmacokinetics – results 160
S14. Statistical analysis 165
S15. Histopathological examination 167
S15.1. Histopathological findings (overview) 168
S16. *In vivo* parameters 173
S16.1. Mice weights 173
S16.2. Observations of mice behavior after the polymer administration 174

ADDITIONAL INFORMATION 176

S17. Applicability of Cy7 for long-term studies 176
S18. Additional figures 176
S18.1. Formulas of dyes and labels 176
S18.2. Moieties in the study polymers 178
S18.3. Properties of the study polymers 179
S18.4. Preparation of phantoms for confocal fluorescence microscopy 180
S18.5. Binning test 180
S18.6. Raw image from fluorescence imaging (Xtreme) 181
S18.7. Preparation of phosphate saline buffer (PBS) 181
S18.8. Images from mice autopsy 182
S19. List of abbreviations 183
S20. Authors Contributions 188
S21. Ethic code 190

REFERENCES 191

ATTACHED FILES 195

S22. Attachments 195
S22.1. Video reconstruction of intramuscular depots (PAI-US) 195
S22.2. NMR files 196
S22.3. Size exclusion chromatograms 196
S22.4. Fluorescence imaging 196

MATERIALS SECTION

S1. Materials

S1.1. Chemicals for synthesis – vendors, purifications, and storage

Dimethylformamide (DMF), methanol (MeOH) and diethyl ether (Et_2O) solvents were purchased from Lach:Ner s.r.o. (Neratovice, Czech Republic) in analytical quality, and DMF was dried with 4.0 Å molecular sieves (208590-500G) for at least 7 days. Cyanine7 amine (Cy7-amine, 450C0, **Figure S116**) was purchased from Lumiprobe (Hannover, Germany), Dyomics DY-505 amino-derivative (505-02, **Figure S117**) was purchased from Dyomics GmbH (Jena, Germany). Argon gas (c_{Ar} ≥ 99.999%) was purchased from Messer Technogas s.r.o. (Prague, Czech Republic). *N*,*N*-diisopropylethylamine (DIPEA, ReagentPlus®, ≥ 99%, D125806-100ML), Sephadex™ LH-20 (by GE Healthcare, Chicago, IL, USA), and (benzotriazol-1-yloxy)tripyrrolidinophosphonium hexafluorophosphate (PyBOP, Novabiochem®, 8510090025) was purchased from Sigma-Aldrich s.r.o (Prague, Czech Republic).

Cy7-amine, Dy505-amine, and PyBOP were stored in dark at –22 °C. Monomers, their precursors, NMR solvents were stored at 4 °C; the remaining chemicals were stored in a dry, dark storage at ambient temperature. All chemicals were used without any additional purification, unless stated otherwise.

S1.2. Chemicals for *in vitro* cellular experiments

PrestoBlue™ (resazurin, **Figure S115**), penicillin, streptomycin, Hoechst 33342 blue (5 mg/mL, **Figure S118**), LysoTracker™ DND-22 (**Figure S119**), CellMask™ Deep red membrane stain (formula not disclosed by vendor, C10046, Invitrogen™), and Dulbecco's Modified Eagle Medium (DMEM; Gibco) were purchased from (Thermo Fisher Scientific, Waltham, MA, USA). Fetal bovine serum (FBS, F7524) was purchased from (Sigma-Aldrich, St. Louis, MI, USA). PrestoBlue™ and DMEM were stored in a refrigerator, and the remaining compounds were stored in dark at –20 °C until their use.

CO_2 flask was purchased from Messer Technogas s.r.o., (Czech Republic, batch no. 56657459). Sodium chloride, calcium chloride (anhydrous), magnesium chloride (hexahydrate), potassium phosphate monobasic, and sodium phosphate dibasic were purchased from Lach:Ner (Neratovice, Czech Republic) in analytical quality. These chemicals were stored in dark at room temperature.

S1.3. Cell cultures

Human Fibroblast (HF) were kindly provided by Institute of Experimental Medicine, Czech Academy of Sciences (IEM, Prague, Czech Republic) and rat mesenchymal stem cells (rMSC) were kindly provided by Institute of Clinical and Experimental Medicine (IKEM, Prague, Czech Republic) were used for experiments.

S1.4. Chemicals for histological examinations

We used H&E Fast Staining Kit (No. 9194) and Van Gieson Trichrome Staining Kit (9193.1) stains for histological examinations (Carl Roth, Karlsruhe, Germany). Corn oil (C8267-500ML, Quality Level: 100) and Paraplast® (Paraffin – polyisobutylene mixture, for tissue embedding, P3558-1KG, Quality Level: 200) was purchased from Sigma-Aldrich s.r.o (Prague, Czech Republic).

All chemicals were used without any additional purification, unless stated otherwise.

S1.5. Animals and materials for *in vivo* experiments

We performed all *in vivo* experiments using BALB/cOlaHsd strain female mice (6 weeks old, purchased from AnLab s.r.o, Prague, Czech Republic). The mice feed (Altromin 1324 Velaz - maintenance diet) was purchased from Velaz, s.r.o. (Prague, Czech Republic). Isoflurane for anaesthesia of the experimental animals was purchased from AErrane (100 %, Baxter Healthcare Ltd., Norfolk, UK).

All materials were stored in a dry storage at room temperature. For details about mice handling, housing, and maintenance, see *section S4.2*. Specific mass-manufactured products used in *in vivo* experiments (such as ultrasound gel *etc*.) are listed in *section S4*.

EXPERIMENTAL SECTION

S2. Polymer preparation and characterization

We synthesized our polymers *via* reversible addition fraction transfer (RAFT) living polymerization (further described in our previous article[1]), which enabled us to prepare polymers with low dispersity indices ($Đ_M \leq 1.10$) and specific end-moieties (**Figure S1**). After the synthesis, we modified the polymers to improve their biocompatibility and traceability for *in vivo* and *in vitro* experiments (**Figure S2** and **Figure S3**). Firstly, we removed the hydrolytically labile terminal trithiocarbonate moiety *via* aminolysis and protected the exposed thiol with methyl acrylate to form a hydrolytically stable terminal moiety (**Figure S1**).[2,3] Secondly, we traced the polymers with fluorescent tracers (Cy7-amine or DY-505 amine, see **Figure S1**). Lastly, we purified the polymers, assessed their purity (**Table S8**) and showed that they were chemically stable over time. Such materials contained no reactive moieties and, based on previous studies on similar materials,[3,4] were projected to be biocompatible.

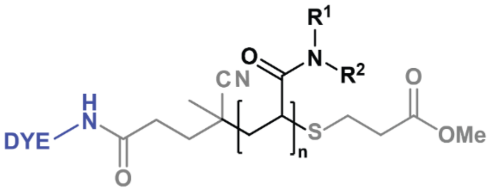

Figure S1. Structure of fluorescently labelled polymers, where **DYE** is Cy7-amine (for *in vivo*, **Figure S116**) or Dy505-amine (for *in vitro* and *ex vivo*, **Figure S117**)

S2.1. Synthesis and characterization of polymers

The synthesis and detailed physicochemical properties of the study polymers (until the conjugation with fluorescent labels) was described in our previous article.[1] Nevertheless, for completeness, we provide a brief report of the synthesis and relevant physico-chemical characterizations in ESI of this article as well.

S2.1.1. Polymer synthesis

We prepared poly[*N*-(2,2-difluoroethyl)acrylamide] (**pDFEA**), poly(*N*-isopropylacrylamide) (**pNIPAM**), poly(*N*,*N*-diethylacrylamide) (**pDEA**) and poly(*N*-acryloylpyrrolidine) (**pAP**) *via* reversible addition−fragmentation chain-transfer polymerization (RAFT)[5] with 4-cyano-4-[(dodecylsulfanylthiocarbonyl)sulfanyl]pentanoic acid as the RAFT polymerization chain transfer agent (CTA)[6] and 4,4′-azobis(4-cyanovaleric acid) (ACVA) as the initiator (**Figure S2**).[5]

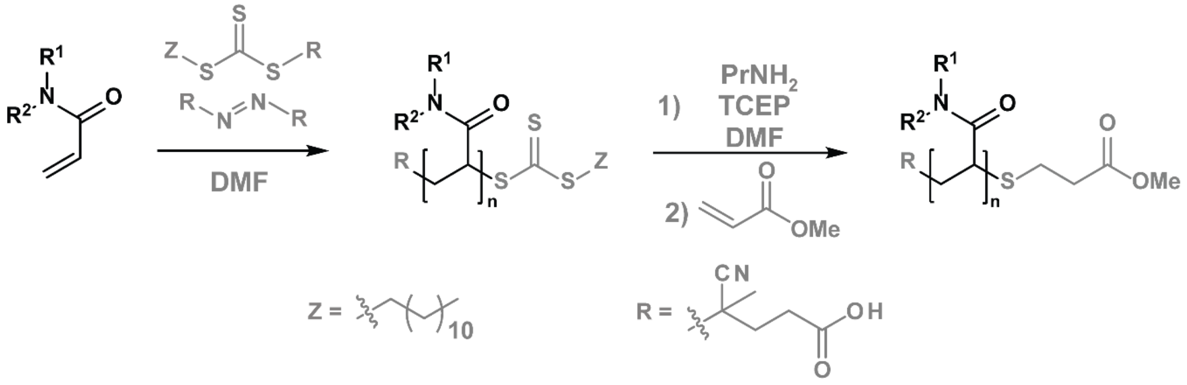

Figure S2. Polymer synthesis and subsequent modifications

The polymerization mixture consisted of a specific monomer (1.50 to 2.00 g), CTA and ACVA in various molar ratios and dried DMF (**Table S1**). The mixture was bubbled with argon in an oven-dried

Schlenk flask, heated to 70 °C in an oil bath and stirred with a magnetic stirring bar overnight. Subsequently, the product was precipitated in diethyl ether, filtered, and the polymer was re-dissolved in methanol and purified using a Sephadex™ LH-20 column and methanol as the eluent. The polymer-containing fractions were evaporated in a rotatory evaporator under reduced pressure, and the polymer was re-dissolved in water and isolated by freeze-drying.

Table S1. Volumes/ mass/ molar amount of solvent (V_{DMF}), monomers (m_{mon}, n_{mon}), charge transfer agent (m_{CTA}, n_{CTA}) and initiator (m_{ini}, n_{ini}) used in polymer synthesis

Polymer/code		V_{DMF} (mL)	m_{mon} (g)	n_{mon} (mmol)	m_{CTA} (mg)	n_{CTA} (µmol)	m_{ini} (mg)	n_{ini} (µmol)
**pDFEA**	**F1**	4.00	1.50	11.1	21.2	52.5	4.41	15.8
	**F2**	4.00	1.50	11.1	14.1	35.0	2.94	10.5
**pNIPAM**	**I1**	6.00	2.00	17.7	56.5	140.0	11.8	42.0
	**I2**	6.00	2.00	17.7	28.3	70.0	5.89	21.0
**pDEA**	**E1**	6.00	2.00	15.7	28.3	70.0	5.89	21.0
	**E2**	6.00	2.00	15.7	14.1	35.0	2.94	10.5
**pAP**	**P1**	4.00	1.50	12.0	42.4	105	8.82	31.5
	**P2**	4.00	1.50	12.0	21.2	52.5	4.41	15.8

After polymerization, the CTA group was removed by aminolysis using propylamine ($PrNH_2$), followed by nucleophilic addition of methyl acrylate to the thiol end-group in dry DMF under an argon atmosphere, as previously reported (**Figure S2**).[7] Polymer-CTA, propylamine, and tris(2-carboxyethyl)phosphine hydrochloride (to prevent the oxidation of thiols to disulfides[8]) were dissolved in DMF (5.00 mL). The reaction mixture was stirred for 120 min at room temperature under a nitrogen atmosphere. Methyl acrylate was added to the reaction mixture and stirred at room temperature for 24 hours. The polymers were purified using the procedure mentioned above. Yields and reactant amounts are summarized in **Table S1** and **Table S8**.

S2.1.2. Polymer characterization – nuclear magnetic resonance (NMR)

After the polymer synthesis, we measured nuclear magnetic resonance (^{1}H NMR, Bruker, 400 MHz, MeOH-4d) of all polymers to assess their structure and purity (**Figure S9** to **Figure S16** in *section S8.1*). Additionally, we provide raw NMR data (*section S22.2*). For details, ^{13}C and HSQC-edit, see our previous article.[1]

Note that the polymer signals are very broad and, therefore, (i) we do not provide a specific chemical shift rather than the general area where the peaks are located, and (ii) some peaks overlap with each other or with trace contaminants (such as solvents), altering the signal integrals.

pDFEA: 8.0 (traces), 6.1-5.8 (t, J = 56.1 Hz, 1H), 3.6-3.5 (t, J = 14.4 Hz, 2H), 2.3-2.1 (1H), 1.8-1.5 ppm (2H).

pNIPAM: 7.6 (traces), 4.0 (1H), 2.1 (1H), 1.7-1.4 (2H), 1.2 ppm (6H).

pDEA: 3.6-3.3 (4H), 2.7-2.6 (1H), 1.7-1.8 (2H), 1.1 ppm (6H).

pAP: 3.1-3.8 (4H), 2.3-2.7 (1H), 2.1-1.7 (4H), 1.7-1.4 ppm (2H).

S2.1.3. Polymer characterization – size exclusion chromatography (SEC)

We determined the number-average molar mass (M_n), weight-average molar mass (M_w), and polymer dispersity ($Đ_M = M_w/M_n$) by SEC using an HPLC Ultimate 3000 system (Dionex, Sunnyvale, CA, USA) equipped with an SEC column (TSKgel SuperAW3000 150 × 6 mm, 4 µm). We employed three detectors: UV/VIS, RI, and MALS; we used methanol and sodium acetate buffer (0.3 M, pH 6.5) mixture (80:20 v/v, flow rate of 0.6 mL·min^{-1}) as a mobile phase. The dn/dc the for given mobile and polymers was 0.110 (**pDFEA**), 0.1540 (**pNIPAM**), 0.2015 (**pDEA**), and 0.1646 mL/g

(**pAM**) at wavelength 620 nm and 20 °C. The resulting spectra are shown in **Figure S17** to **Figure S20**, see *section S8.2,* and we also provide raw data (*section S22.3*).

S2.2. Differential scanning calorimetry (DSC)

Previous studies[9–11] showed that aggregates of aqueous **pNIPAM** can vitrify over time, which can increase their demixing time. However, to date, vitrification of remaining polymers has not been studied. Thus, we used calorimetry to study vitrification of our polymers.[9–11] Moreover, we measured the T_g of our polymers in their pure (bulk) form.

We assessed the thermal behavior of our polymers using differential scanning calorimetry (DSC) and modulated-temperature differential scanning calorimetry (MDSC) on a Q 2000 calorimeter V24.11 Build 124 (TA Instruments, New Castle, DE, USA) using a refrigerated cooling system RCS90, calibrated with indium and sapphire standards (TA Instruments, New Castle, DE, USA).

S2.2.1. Pure (bulk) polymers

The samples (**F2**, **I2**, **E2**, and **P2**; each 0.5 to 1.5 mg) were encapsulated in aluminium pans. All DSC measurements were performed under a nitrogen atmosphere (at a flow rate of 50 mL/min) using a heat−cool−heat cycle in a temperature range of 0 to 200°C with a heating/cooling rate of 10 °C/min and 2 min isotherms inserted between the cycles. The glass transition temperature (T_g) values were extracted from the second heat scan and determined as the midpoint of heat flow change. All results are shown in **Figure S21** to **Figure S24** and **Table S9**.

S2.2.2. Aqueous solutions of polymers

The samples (20 *wt*. % aqueous solutions of **F2**, **I2**, **E2**, and **P2**; each 0.7 to 3 mg) were encapsulated in aluminium hermetic Tzero pans. We performed all DSC measurements under a nitrogen atmosphere (at a flow rate of 50 mL/min) using a temperature amplitude of ± 0.5 °C and a period of 60 s in a temperature range of –30 to 60 °C (**F2**, **I2**, **E2**) or –30 to 80 °C (**P2**) with a heating rate of 1.0 °C/min. The T_{CP} was determined as an onset point of endothermic peak on the reversible C_p curve (**Figure S25** to **Figure S32**), and the enthalpy change for the aggregation ($\Delta H_{aag,neat}$) was calculated from the area under the endothermic peak (**Table S10**).

S2.3. Conjugation of polymers with fluorescent labels

The fluorescent tracers enabled polymer visualization *in vitro* and *in vivo*. For *in vivo* fluorescence imaging, we used a biocompatible[12,13] Cy7 label (**Figure S116**) with excitation (750 nm*) and emission wavelengths (788 nm*) whose depth of penetration through the skin and tissues is greater than that of shorter wavelengths.[13–17] However, our confocal microscopes had low sensitivity at such a long wavelengths. For this reason, we labelled the polymers with biocompatible Dy505-amine (**Figure S117**, rhodamine derivative) with shorter excitation (507 nm*) and emission (528 nm*) wavelengths for *in vitro* experiments.

S2.3.1. Polymer conjugation (synthesis)

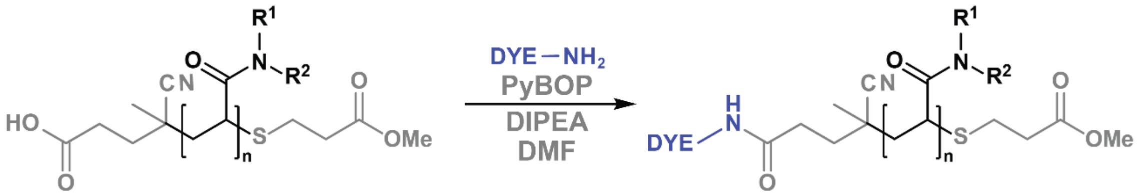

Figure S3. A scheme of polymer tracing with a fluorescent dye

We dissolved the polymers (100 mg each) in dry toluene (adding 15 mL, 0.1 to 1.5 mL of acetone if needed to dissolve the polymer) and evaporated the solvent on a rotatory evaporator. After repeating this process five times, we maintained the polymers in vacuum ($p \leq 10$ mBar) at room temperature for

3 days to remove trace solvents. Subsequently, we traced these polymers with fluorescent dyes (Cy7-amine (**Figure S116**) or Dy505 (**Figure S117**), as shown in **Figure S3**) under various reaction conditions (**Table S2** to **Table S4**) and dry DMF under an argon atmosphere. The reaction was performed in a 2-mL reaction vessel for 14 hours at room temperature. Subsequently, DMF was evaporated on a rotatory evaporator; the polymer was dissolved in methanol (0.5 to 1.2 mL) and purified by gel separation with flash chromatography (puriFlash®, Interchim, Montluçon, France) on a Sephadex™ LH-20 column using methanol as the eluent. Lastly, we evaporated the solvent from the polymer-containing solutions on a rotatory evaporator, dissolving the polymers in water freeze-dried (≈ –190 °C, ≈ 16.5 Pa, 20 hours). The temperature of the sample never exceeded 40 °C during the reaction, processing, or storage. The labelled polymers were stored in dark at –20 °C until their use.

Table S2. Mass and molar amount of previously prepared polymers (m_{pol}, n_{pol}), propylamine (V_{PA}, n_{PA}), tris(2-carboxyethyl)phosphine hydrochloride (m_{TCEP}, n_{TCEP}), and methyl acrylate (V_{MA}, n_{MA})

Polymer/code		m_{pol} (g)	n_{pol} (µmol)	n_{PA} (mmol)	V_{PA} (µL)	n_{TCEP} (µmol)	m_{TCEP} (mg)	n_{MA} (mmol)	V_{MA} (mL)
pDFEA	F1	1.00	38.2	1.91	81.2	38.2	11.0	6.69	0.55
	F2	1.00	27.6	1.38	58.7	27.6	7.9	4.83	0.40
pNIPAM	I1	1.00	49.5	2.48	105.2	49.5	14.1	8.66	0.71
	I2	1.00	31.6	1.58	67.2	31.6	9.1	5.53	0.45
pDEA	E1	1.00	44.8	2.24	95.2	44.8	12.8	7.84	0.64
	E2	1.00	28.8	1.44	61.2	28.8	8.3	5.04	0.41
pAP	P1	1.00	51.0	2.55	108.4	51.0	14.6	8.93	0.73
	P2	1.00	27.8	1.39	59.1	27.8	8.0	4.87	0.40

Table S3. Masses and volumes of reactants in tracing of polymers with Cy-7 amine label. The polymers (m_{pol}, n_{pol}) were dissolved in (V_{DMF}). Other reactants were dissolved in DMF, and their according volumes were added to the mixture, flushed with argon and mixed at room temperature for 14 hours.

Polymer		m_{pol} (mg)	n_{pol} (µmol)	V_{DMF} (µL)	m_{Cy7} (mg)	n_{Cy7} (µmol)	m_{PyBOP} (mg)	n_{PyBOP} (µmol)	m_{DIPEA} (µg)	n_{DIPEA} (µmol)
pDFEA	F1	20.0	1.02	500	0.89	1.24	6.47	12.4	268	2.07
	F2	20.0	0.65	500	0.62	0.85	4.46	8.57	184	1.42
pNIPAM	I1	20.0	0.83	500	1.10	1.53	7.97	15.3	330	2.55
	I2	20.0	0.57	500	0.70	0.97	5.08	9.76	210	1.62
pDEA	E1	20.0	0.95	500	1.02	1.42	7.38	14.2	306	2.37
	E2	20.0	0.63	500	0.68	0.94	4.91	9.44	204	1.58
pAP	P1	20.0	1.14	500	1.23	1.70	8.87	17.0	367	2.84
	P2	20.0	0.61	500	0.66	0.91	4.75	9.13	197	1.57

Table S4. Masses and volumes of reactants in tracing of polymers with Dy505 amine. The polymers (m_{pol}, n_{pol}) were dissolved in (V_{DMF}). Other reactants were dissolved in DMF, and their according volumes were added to the mixture, flushed with argon and mixed at room temperature for 14 hours.

Polymer		m_{pol} (mg)	n_{pol} (µmol)	V_{DMF} (µL)	m_{DY505} (µg)	n_{DY505} (nmol)	m_{PyBOP} (mg)	n_{PyBOP} (µmol)	m_{DIPEA} (mg)	n_{DIPEA} (µmol)
pDFEA	F2	20.0	0.65	500	502	832	5.07	9.75	1.30	10.0
pNIPAM	I2	20.0	0.57	500	502	832	4.45	8.55	1.30	10.0

**pDEA**	**E2**	20.0	0.63	500	502	832	4.93	9.47	1.30	10.0
**pAP**	**P2**	20.0	0.61	500	502	832	4.76	9.14	1.30	10.0

S2.3.2. Purity of Cy7-amine labelled polymers

Polymer purity was determined by SEC. The setup was identical to that in SEC measurements in *section S2.1.3*, but the UV/VIS detector was set to $\lambda = 665$ nm. The resulting spectra is shown in **Figure S33** to **Figure S41**, see *section S8.4*.

Note that, in some cases, the polymer absorbance and/or light-scattering surpassed the detection maxima of detectors. However, this was only a minor issue. Although the detector overflow prevented us from quantifying the concentration of free (non-bound) Cy7-amine accurately, the concentration of free Cy7 amine was, in all cases, negligible to undetectable. Even under these conditions, the purity of all measured polymers was higher than 99.5%.

S2.3.3. Amount of Cy7 in labelled polymers

We measured the amount of Cy7 in all polymers using spectrophotometry (Sunrise microplate reader; Tecan Group Ltd., Switzerland). We dissolved Cy7-amine in methanol in 50.0, 25.0, 12.5, 6.75 mg/mL and 3.13 $\mu g \cdot mL^{-1}$, the absorbance of these solutions was measured (**Figure S42**) and determined the molar extinction coefficient for Cy7-amine. Subsequently, we dissolved a known amounts of all eight Cy7-amine labelled polymers in methanol (c_{pol} = 1.0 mg/mL) and measured the absorption of these polymers. From the absorptions, we calculated the amounts of Cy7 in all polymers (**Table S11**).

S2.3.4. Chemical Stability of Cy7 amine label

We investigated the stability of the fluorescent as a function of time. We dissolved the polymers in a mixture of sodium acetate buffer (0.3 M, pH 6.5) and methanol (80:20 v/v; c_{pol} = 1.0 mg/mL) and incubated this mixture in dark at 37°C. Then, 1 hour, 50 hours and 200 hours after the preparation we measured the SEC with identical experimental setup as stated in *section S2.3.2*. We observed no increase of free Cy7.

S2.3.5. Fluorescence characteristics of Cy7-labelled polymers

Cy7-labelled polymers **F2**, **I2**, **E2**, **P2** and Cy7 dye as a control were measured as solutions in deionized water (c_{pol} = 1.25 mg/mL for polymers and ≈ $1 \cdot 10^{-6}$ M for Cy7). Fresh solutions were prepared and kept in the dark at room temperature for no longer than 2 h. Absorption and emission spectra were measured by diode array spectrophotometers in a specialized T-shaped fluorescence cuvette with thick walls and high thermal capacity (internal volume 100 µL, optical pathway 10 mm transmission, 2 mm emission) tempered to 0 and 60 °C with a water bath, respectively. The spectra were measured immediately (within 60 s) after the cuvette was removed from the water bath and the sample temperature did not vary by more than ± 5°C from the bath temperature. The absorbance of the fluorescent dye was kept below 0.1 and the excitation parameters were set so that the measured intensity is lower than ≈ 50% of the maximal intensity within the linear response regime of the fluorimeter.

S2.3.6. Purity of Dy505-amine labelled polymers

As in *section S2.3.2*, we used SEC to determine the purity of Dy505 labelled polymers. The setup was identical to that in SEC measurements in *section S2.1.3*, but the UV/VIS detector was set to $\lambda = 505$ nm. The resulting spectra are shown in **Figure S43** to **Figure S47**, see *section S8.5*.

S2.3.7. Amount of Dy505 in labelled polymers

Then, we measured the amount of Dy505-amine in all polymers using spectrophotometry (Sunrise microplate reader; Tecan Group Ltd., Switzerland). We dissolved Dy505-amine in methanol in 50.0, 25.0, 12.5, 6.75 mg/mL and 3.13 $\mu g \cdot mL^{-1}$, and we measured the absorbance of these solutions (**Figure S48**) and determined the molar extinction coefficient for Dy505-amine. Subsequently, we

dissolved known amounts of all eight Dy505-amine labelled polymers in methanol (c_{pol} = 1.0 mg/mL) and measured the absorption of these polymers. From the absorptions, we calculated the amounts of Dy505-amine in all polymers (**Table S12**).

S3. *In vitro* biological experiments

To evaluate the safety of the polymers, we assessed their cytotoxicity using human fibroblasts (HF) and rat mesenchymal stem cells (rMSC) and the Alamar blue assay (*section S3.1*).[18] HF were selected as model cells because they are commonly found in muscles, the site of injection of our polymer solutions. The rMSC were chosen as a highly sensitive model of normal healthy cells. We observed no significant differences in cell viability between the experimental groups (cells treated with the polymers) and the control group (untreated cells; **Figure S56** and **Table S13** to **Table S22**). Therefore, in line with previous results, our polymers were non-cytotoxic.[19,20]

S3.1. Cytotoxicity assay

We cultivated the cells at 37 °C in 5% CO_2 atmosphere in Dulbecco modified Eagle medium (DMEM) with phenol red indicator and supplemented with a heat inactivated 10% fetal bovine serum (FBS), penicillin, and streptomycin. The adhered cells were incubated overnight in 25 cm^3 flask at 37°C in 5% CO_2 atmosphere before treatment.

First, we seeded the cells (HF or rMSC) in a 96-well plate (TPP, Biotech, Switzerland, Tissue Culture test plates 96F Growth enhanced treated, product No. 92696) at a density $7 \cdot 10^3$ cells per well. Subsequently, we dissolved the thermosensitive polymers **F2**, **I2**, **E2**, or **P2** in PBS at concentration from 0 to 100 µg/mL at room temperature and added these solutions to the cell cultures. Subsequently, we incubated the cells for additional 72 hours, and assessed the cell viability using resazurin assay (PrestoBlue™, Thermofisher Scientific Waltham, MA, USA) according to manufacturer's instructions. Untreated cells (no added polymer; only pure PBS) were used as negative controls; cells treated with 4 mM H_2O_2 in PBS were used as positive controls.

We expressed the results as viability percentage relative to the negative control (**Figure S56**, **Table S13** and **Table S14**); the results were processed in GraphPad Prism5 software (GraphPad software, San Diego, CA, USA) by one-way analysis of variance (ANOVA), setting the level of significance at $p \leq 0.05$ (**Table S15** to **Table S22**), as described in *section S9.1*.

S3.2. Intracellular uptake and colocalization study

S3.2.1. Cell culture preparation and staining

We seeded HF and rMSC cells were seeded at 37 °C in 5% CO_2 at the bottom of µ-Slide 8 well dishes (IBlTreat; cat. No. 80826; polymer coverslip; ibidi, Gräfelfing, Germany) at cell density $4 \cdot 10^4$ per well in 100 µL DMEM medium and left to adhere overnight.

We dissolved the Dy505-labelled polymers **F2**, **I2**, **E2**, or **P2** in cold PBS to a fluorophore concentration of ≈ 70 µg/mL. Then, we added this solution to culture media, the final concentration of fluorophore in media was 7 µg/mL. We incubated the Dy-505 labelled polymers (**F2**, **I2**, **E2**, or **P2**) with cells for 4, 16, or 24 hours at 37 °C in 5% CO_2 atmosphere in dark (covered by aluminium foil). Then, the medium was decanted, and the cells were washed in cooled PBS 3 times (custom prepared, containing Mg^{2+}, Ca^{2+} ions; see *section S18.6*).

We stained cell membranes using CellMask™ Deep red membrane stain (C10046, diluted 1000-fold, at a final concentration of 5.0 µg/mL and treated the cells for 10 min at room temperature), and cell nuclei using Hoechst blue 33342 (**Figure S118**; diluted 1000-fold, at a final concentration of 5.0 µg/mL, and treated the cells for 10 min at room temperature), or lysosomes using the LysoTracker™ DND-22 (**Figure S119**, Molecular probes, life technologies; final working concentration in media was 75 nM; cells were treated for 30 min at room temperature). Lastly, we washed the cells with PBS and then visualized for maximum time 90 minutes. The resulting images are shown in **Figure 2** and **Figure S57**.

S3.2.2. Microscopy details

We evaluated the lysosomal colocalization of polymers by Laser scanning confocal microscopy (LSCM). We visualized cells under an Olympus FV10-ASV confocal laser scanning microscope (Olympus Czech group Ltd., Prague, Czech Republic) equipped with a 60× oil objective. We acquired images using an Olympus Plan Apo N 60x oil objective numerical aperture (NA) 1.42, pinhole size 110 µm and pixel dwell time set to 20 µs. Multicolour images were taken in line sequential mode. The pictures were acquired in a 2D and/ or 3D mode. The 3D model was reconstructed in some images to investigate the localization of the polymers (whether inside or outside the cells). 3D animations were reconstructed from a series of 7 images with variable *x* axis.

We visualized:

1) polymers (Dy-505 labelled) with λ_{ex} = 505 nm and λ_{em} = 530 nm; emission was acquired in the range 500 to 600 nm
2) cell nuclei (using Hoechst 33342) with λ_{ex} =350 nm and λ_{em} = 461 nm; emission was detected in the range 425 to 475 nm
3) cell membranes (using Cell mask red dye) witht λ_{ex} = 649 nm and λ_{em} = 666 nm; emission was detected in the range 650 to 750 nm
4) lysosomes (LysoTracker™ DND-22) with λ_{ex} = 375 nm and λ_{em} = 422 nm; emission was acquired in the range 425 to 475 nm

Note that we never used nuclei stain and lysosome stain simultaneously; we always used one or the other (see **Figure 2**, **Figure S57** and **Figure S58**). Lastly, we analyzed images and calculated and level of the colocalization (Pearson correlation coefficients, PCC) in the region of interest (ROI) under an Olympus Fluoview FV10-ASW ver.04.02b.

S4. *In vivo* biological experiments

After confirming the safety of the polymers *in vitro*, we studied their pharmacokinetics following intramuscular administration. For long-term polymer monitoring, we selected fluorescence imaging, a non-invasive imaging technique that enables us to assess the cellular distribution and quantities of fluorescently labelled polymers as a function of time. Advantageously, this technique does not expose the test animals to radiation,[14] thus avoiding pathologies caused by radiation in chronic studies. Disadvantageously, the signal can be strongly affected by the geometry and depth of the study depots because thicker tissues can absorb a portion of both excitation and emission light, possibly distorting the results. However, in our experiments, the geometry and depth of the polymer depots in each animal remained nearly constant as a function of time. In addition, we monitored the site of administration by ultrasound-photo-acoustic imaging (US-PAI, *section S4.4* and *S12*) to ensure that the polymers would (i) be located at similar depths in all mice and (ii) avoid major blood vessels or bones. As a result, we can use our imaging data to compare the signal from each mouse as a function of time to determine polymer dissolution kinetics.

S4.1. *In vivo* study design

In this study, we used thigh muscles of mice as a model tissue because they are easily accessible in mice, near skin surface, and muscle tissue is relatively homogeneous. Nevertheless, we have previously demonstrated that thermoresponsive polymers behave similarly in subcutaneous depots as well.[21]

Although all study polymers dissolved in water, so we were able to inject them as aqueous solutions, such solutions may partially solidify in the syringe during the administrations. Such a premature solidification would pose only minor issues in clinical applications but could result in an uneven distribution of polymers at the implant site (local hotspots; difficult to quantify), distorting the pharmacokinetics measurements. To avoid the premature solidification of the polymers during their administration, we administered the polymers in dimethyl sulfoxide (DMSO) solutions (common solvent in medicine, a single dose of DMSO is usually well tolerated[22]). Solutions of thermoresponsive polymers in DMSO do not show thermoresponsive behavior,[23,24] therefore we can avoid the premature solidification of polymers. However, immediately upon administration, DMSO is diluted with water and thermoresponsive polymers solidify forming hydrogel aggregates *in situ*.[23,24] Nevertheless, for future studies or clinical applications, aqueous solutions (or pH-responsive systems[21]) should be used.

Subsequently, we prepared our mice for pharmacokinetics experiments. Before the administration, we trimmed and depilated their fur to avoid signal skewing by fur (*section S4.2*). Subsequently, we anaesthetized the mice, and administered corresponding solutions into the thigh muscles of mice (*section S4.4*). To maximize the reproducibility of our study, we monitored the site of administration using US-PAI to set the depth and location of intramuscular bolus to avoid any major blood vessels (*section S4.3*). Study groups of mice received DMSO solutions of Cy7-amine labelled polymers (**F_1**, **F_2**, **I_1**, **I_2**, **E_1**, **E_2**, **P_1**, and **P_2**, see **Table 1**). Additionally, to determine any possible undesirable effects of polymers (toxicity, muscle damage, changes in behavior or feeding patterns; see *section S16*), we had three control groups: the control mice received pure DMSO (**DMSO group**), saline solution (**saline group**) or nothing at all (**naive group**). Lastly, one group of mice was administered a DMSO solution of Cy7-amine hydrochloride in DMSO (**Cy7 group**). In this control group, we assessed the dissolution kinetics of non-polymer bound Cy7-amine hydrochloride, which could function as an anchor holding the polymer in tissues. If so, the biological half-lives of Cy7-amine hydrochloride would be similar to those of Cy7-labelled polymers.

After the administration, we assessed the polymer signal and distribution *via* fluorescence imaging at planned intervals (**Figure 4** and **5**, *section S4.5*). From these data, we calculated the sum of signal (I, equal to sum of all pixels in the region of interest) and the depot size (S, equal to number of pixels with

signal above a set threshold; *section S5.4*). Additionally, we evaluated intensive depot distribution descriptors (distribution index, K_{10}), which provide information about the polymer distribution within the depot (*section S5.6*). Lastly, we evaluated the depot volume (V) using US-PAI (*section S5.3.3*). All these quantities are shown in **Table S25** to **Table S79** and were used to characterize the biodistribution and pharmacokinetics of the study polymers.

S4.2. General preparation

The experiments in this study were approved by the ethics committee of the First Faculty of Medicine, Charles University, and by the Ministry of Education, Youth and Sports of the Czech Republic. The experiments were performed according to ethic codes of the First Faculty of Medicine, Charles University and the Czech Academy of Sciences.

All experiments were performed in the accordance with Directive 2010/63/EU of the European Parliament and the council on the protection of animals used for scientific purposes, Act No. 359/2012 Sb. on the Protection of Animals Against Cruelty and Precepts 419/2012 Sb. and 299/2014 Sb. Ministry of Agriculture on the Protection of Experimental Animals (including relevant EU regulations as of 2020/2021). All mice experiments were conducted by authorized and experienced personnel according to the recommended laboratory guidelines.

The mice were housed according to the approved guidelines (in individually ventilated cages with the sterilised bedding and cellulose sheets, 12:12 hours light-dark cycle at 22 ± 1 °C and 60 ± 5% humidity), and they were fed Altromin 1324 Velaz (maintenance diet) and drank water (purified by Smart N-II, Heal Force Bio-meditech Holdings Limited, Shanghai, China) *ad libitum*. The BALB/c mice (n = 42) were let to acclimatize for 5 to 7 days. Subsequently, they were randomly divided into 10 groups: **F1** (n = 3), **F2** (n = 3), **I1** (n = 3), **I2** (n = 3), **E1** (n = 3), **E2** (n = 3), **P1** (n = 3), **P2** (n = 3), **Cy7 group** (n = 3), **DMSO group** (n = 6), **saline group** (n = 3) and **naive group** (n = 6). The mice were maintained in proper cages; one or two groups per cage; 3 to 6 mice per cage (**I2** + **F2**, **P2** + **E1**, **E2** + **I2**, **F1** + **P1**, **Cy7**, **saline group**, **DMSO group**, **naive group**). The mice in each group were marked by one to six bands on their tails with a black permanent marker (OHP marker permanent, alcohol based, waterproof; Centropen, Dačice, Czech Republic) and by ear punch patterns (long-term mark).

Subsequently, the fur on the back, abdomen and left hind leg of the mice was shaved before every imaging experiment (except for day 2 to 4). Firstly, the fur was trimmed using Aesculap® Exacta GT415 (Braun, Kronberg, Germany). Then, the mice were anaesthetized using isoflurane (AErrane® 100%; Baxter Healthcare, Deerfield, USA; 3.0 to 3.5% initial concentration; 1.0 to 2.0% maintenance concentration, 1.2 L/min air flow) using continuous flow Isoflurane Tec 3 vaporizer (ASA Ltd., Keighley, UK). Their residual fur was removed on the left hind leg and surrounding areas using Veet Silk Fresh™ (thiglycolic acid-containing depilatory cream; Reckitt, Slough, UK) under isoflurane anesthesia. The excessive depilatory cream was removed after 3 to 7 min, and the skin was thoroughly washed with warm tap water (warm to touch, *ca.* ≈ 30 °C) and dried by paper towels.

S4.3. Ultrasound-photoacoustic imaging

S4.3.1. Instrument setup

We performed the multimodal high-frequency ultrasound-photoacoustic imaging (US-PAI) using Vevo 3100/LAZR-X (ultrasound/photoacoustic) multimodal imaging platform (FUJIFILM VisualSonics, Inc., Toronto, Canada). We used a high-frequency ultrasound Mx400 transducer (40 MHz, 256 elements linear array, 50 µm axial and 110 µm lateral resolution, FUJIFILM VisualSonics, Inc., Toronto, Canada) equipped with an original plastic jacket for inserting the green narrow optical fibre bundle (14 mm) optical cable (FUJIFILM VisualSonics, Inc., Toronto, Canada) to record the US-PAI of hind limbs in B-Mode and Photoacoustic Multiwavelength Mode

using Mouse Large Abdominal pre-set (as default). The Time Gain Compensation (TGC) settings was used to see deep within hindlimb tissues by increasing the PAI gain of each pattern (10-55-65-72-75 dB), beginning 9 mm from the upper distance of the transducer surface and proceeding to the bottom hind limb layer at 18 mm (US-PAI field of view 9 to 18 mm).

S4.3.2. Experiment setup

Vidisic® 2 mg/mL gel (Bausch+Lomb, Laval, Canada) was applied in copious amounts to mice eyes before, during, after and in between all visualization experiments to prevent the drying out and *keratitis sicca*.[25,26] The mice were anaesthetized with isoflurane (AErrane® 100%; Baxter Healthcare, Deerfield, USA, 3.0% initial concentration; 1.5 to 2.0% maintenance concentration; 1.2 L/min air flow) and fixed in the prone position on heated table (38.8 °C, FUJIFILM VisualSonics Inc., Toronto, Canada). The feet of the mice were covered in SignaGel Electrode Gel (Parker Laboratories, Inc., Fairfield, USA) to provide a conductive contact with electrodes for ECG monitoring with a build-in ECG monitor. Furthermore, their breathing function was monitored with a build-in breathing monitor. Then, their left hind limb (supported with the cellulose square) was positioned to 10 mm from the transducer surface. The space between transducer surface and hind limb skin line was filled by a bubble-free clear transparent ultrasound gel (OXD professionalcare, OXD by TELIC, S.A.U., Barcelona, Spain). All 3D ultrasound records were acquired using a 100 µm step size on a motorized VevoRail system (FUJIFILM VisualSonics, Inc., Toronto, Canada). The 3D multiwavelength records were measured at 680, 695, 788, 924, and 970 nm (multiwavelength mode), which enabled us to distinguish between deoxyhemoglobin, oxyhemoglobin, and Cy7-amine dye (either free or a polymer bound). Photoacoustic spectra (680 to 970 nm; 5 nm increment) were recorded to differentiate oxyhaemoglobin, deoxyhaemoglobin and Cy7-amine (either free or a polymer bound) at the site of bolus injection and surrounding tissues.

S4.3.3. Data evaluation

The US-PAI data acquired during the injections and subsequent scans at time were postprocessed using Vevo LAB V.3.2.5. software (FUJIFILM VisualSonics, Inc., Toronto, Canada). We determined the volumes of hindlimb depots by volumetric analysis in software Vevo LAB and obtained values of PA intensity for Cy7 dye were plotted in software Origin Pro 8 (OriginLab, Northampton, MA, USA). Additionally, we provide video reconstructions of some IM depots as a function of time (*section S22.1*).

S4.4. Polymer administration

The mice were scanned using US-PAI before, during and after the administration of the solution. The mice from groups **F1**, **F2**, **I1**, **I2**, **E1**, **E2**, **P1**, and **P2** were injected with the corresponding polymer in **DMSO** (c_{pol} = 0.10 mg/µL, 5.00 µL). The **DMSO group** and **saline group** (control groups) were injected with pure DMSO (European Pharmacopoeia reference standard; 5.00 µL) or saline (Fresenius Kabi 0,9 %, Fresenius Kabi s.r.o., Praha, Czech Republic; 5.00 µL). Lastly, **Cy7 group** (control group) was injected with 5.00 µL of Cy7-amine hydrochloride solution (**Figure S116**, 1.10 mg was dissolved in 1.000 mL of DMSO and used for Cy7 control group).

These solutions were administered intramuscularly using Vevo Infusion Pump (volume 5.00 ± 0.05 µL; injection speed 1.6 µL/s; FUJIFILM VisualSonics, Inc., Toronto, Canada) using a 0.5 mL insulin syringe (Insulin U100; 0.5 mL; 29G × 1/2", Chirana T. Injecta, Stará Turá, Slovak Republic) under general anesthesia. During the administration, mice ECG and breathing were monitored using Vevo 3100/LAZR-X. After the administration, the mice were cleaned using cellulose tissues, placed on a plate heater with adjusted temperature (37.7 °C; until the mice regained awareness. Subsequently, they were either placed back into their cages, or they were immediately used in fluorescence imaging (Xtreme measurement, *section S4.5*).

The last *ex vivo* biodistribution group served as a control group without any treatment for first 9 days of the experiment (naive group) – no solution was administered; no measuring procedures were performed (except weight measurements and behavior observations). Their fur was shaved at the beginning, but not depilated during the first phase. The purpose of this group was to determine the short-term effect of the intramuscular administration and related procedures on the well-being of the mice.

S4.5. Long-term *in vivo* optical fluorescence imaging (Xtreme)

S4.5.1. Instrument setup

We used Xtreme In Vivo Imaging System (Bruker Biospin, Ettlingen, Germany) with excitation filter 750 nm and the emission filter 830 nm.

S4.5.2. Experiment setup

In the long-term, we monitored the fluorescence signal at specific timepoints (1-, 5-, 24-, 48-, and 72-hours past administration and then on day 7, 15, 29, 57[i], 70, 85, 120, 150, 180, 245, and 540[ii]). Before the measurement, both sides of the body and right leg of mice were shaved with an electric trimmer and hair removal cream (see *section S4.2*). We anaesthetized the mice with isoflurane (3.0% initial, 1.5 to 2.0% maintenance concentration) and acquired the images (in at least two independent imaging experiments at the given timepoints), the pixel binning was adjusted according to the need.

Each mouse from each group was given its number and an assigned position (slot) in the Xtreme Imaging system. For first three time-intervals (0-, 4-, and 24-hours past administration), we acquired the image of each mouse separately to avoid interference of signal (but every mouse was already placed into its assigned slot). During the following intervals, we acquired the fluorescence signal of all three mouse from each group simultaneously.

In most cases, we acquired fluorescence images with a resolution 1024 by 1024 pixels image (binning number 2). In rare cases, if the polymer signal was too high, we acquired the images with lower binning (binning number 1) to avoid detector signal overflow. These images with higher resolution (2048 by 2048 pixels) were subsequently transformed into 1024 by 1024 pixels images; the values of the individual pixels ranged from 0 to 70,000). In *section S18.5*, we demonstrated that acquiring images with lower binning and recalculation provides precise values of fluorescence signal without any value distortion.

We used the data were to evaluate the biodistribution of polymer pharmacokinetics (see *section S5*).

S4.6. Measurement of biological parameters of mice

S4.6.1. Instrumental and experimental setup

We measured the mice weights on day 1, 2, 3, 4, 7, 11, 15, 18, 28, 57, 72, 85, 123, 155, and 247 using CB 1001 Compact Balances scale (Adam Equipment Co. Ltd., Milton Keynes, U.K.), because major variations of mice weight (increase, decrease of stop of growth) may indicate major pathologies to the mice. We compared the weights of the testing groups (**F_1**, **F_2**, **I_1**, **I_2**, **E_1**, **E_2**, **P_1**, and **P_2**) with those of the control groups (**Cy7**, **DMSO**, **saline**, and **naive groups**). The values are shown in **Figure S112**, **Table S94** and **Table S88**.

---

[i] All fluorescence imaging data were acquired and processed by a single operator (J.P.), except for day 57, which was acquired by a different operator (P.M.). In some mice, data from day 57 were outliers (see *section S12.2*). This can be attributed to the slightly different acquisition techniques by the different operator (*e.g.*, the mice might have been acquired from slightly different angle than usual, which may explain the outlier values). As a result, data from day 57 were not used for fitting in any mice, however, we provide these data anyway to whom it may concern.

[ii] Although these data were acquired, they were not used for analysis of pharmacokinetics, because at that point mice were much older than at the beginning of the experiment, which may have altered the dissolution kinetics.

Additionally, we observed mice behavior after polymer administration (**Figure S113**) and lifespans (**Figure S114**).

S4.7. *Ex vivo* experiments

Lastly, the mice (12 specimens of C57BL/6 strain) we shaved and depilated on the left back limb (identical procedure to the rest of the animals). Subsequently, three groups of four animals were administered with **P1** and **F1** (c_{pol} = 0.10 mg/µL) or **DMSO** solutions (5.00 µL) respectively. After 30 min or 2/ 4/ 8 days, one mouse from each of three groups was euthanized (cervical dislocation) and samples of their muscles (the site of administration), liver and kidney were extracted. The tissue samples were immediately chopped using McIllwain Tissue Chopper (Stoelting Co., Wood Dale, IL, USA) into 150 or 200 µm wide slices respectively and incubated in Petri dish with Hoechst 33258 solution 1:80,000 in 0.1% triton (Sigma Aldrich, 861405; X100) for 10 minutes in the dark to stain nuclei for better orientation in the tissue. The stained slices were placed on microscope slides and covered with cover slips. The samples were immediately photographed using a confocal microscope Olympus Fluoview FV1000 Confocal Laser Scanning BX61 Microscope (Olympus Czech group Ltd., Prague, Czech Republic). The polymer was visualized using excitation wavelength 635 nm and nuclei by Hoechst 33258 (**Figure S120**) with excitation wavelength 405 nm. We acquired images using Olympus UPlanSApo 20× objective NA 0.75. Finally, we processed and visualized the images under an Olympus FluoView Ver.4.2b (**Figure S59** to **Figure S61**).

Note that visualization of the polymers was difficult because the polymers are soluble in water and alcohols, preventing the use of water- or alcohol-based fixatives and freezing methods of tissue processing. Therefore, we were unable to fix the extracted tissues before processing. As a result, the samples were processed quickly, on ice, to avoid autolysis (due to lack of tissue fixation).

S5. Biodistribution data processing

S5.1. Study parameters

We processed the data from long-term *in vivo* fluorescence imaging (Xtreme imaging, *section S4.5*) and evaluated the following extensive (1 and 2) and intensive (3) parameters:

1) **Total signal** (I) in the field of view of the corresponding mice/ organ (sum of all pixels with intensities greater than threshold) was calculated as a sum of all pixels in the region of interest. We evaluated this parameter for thigh (site of administration, I_{IM}), kidney (I_{KID}) and liver (I_{LIV}) depots
2) **Depot size** (S) was calculated as the number of pixels in the region of interest with values above predetermined threshold. We evaluated this parameter only for IM depots (S_{IM}).
3) **Distribution index** (K_{10}) was calculated according to **Equation S1** (further explained in *section S5.6*).

$$P_{10} = \frac{I_{+,0.9}}{I_{+}} \cdot 100\% \qquad (\mathbf{S1})$$

where I_{+} is the total signal of pixels in the depot (sum of all pixels above a threshold limit) and $I_{+,0.9}$ is the total intensity in the top 10% most intensive pixels within depot (in other words, the intensity of top 90 percentile of all pixels, whose intensity is above the threshold limit). Simply put, this index calculate what percentage of signal is localized within the 10% most intensive pixels of the depot.

Lastly, from photoacoustic imaging, we were able to detect the fifth parameter, **depot volume** (V_{IM}). In this parameter, we evaluated the volume of tissue where Cy7 signal was still detectable and expressed it in mm^3. All raw data as a function of time are shown in **Table S25** to **Table S79** and **Figure S64** to **Figure S108**.

S5.2. Phases of signal

Based on the variation of *in vivo* signal intensity of intermuscular depots (I_{IM}) as a function of time, we proposed a pharmacokinetics model with three (partly overlapping) phases **1a**, **2a**, and **3a** (*section S5.2.1*). Similarly, polymer pharmacokinetics in secondary (liver and kidney) depots can be characterized by fluorescence signal in these organs (I_{KID} in kidney and I_{LIV} in liver) in four phases (**Phase 1b**, **2b**, **3b**, and **4b**, see *section S5.2.2*).

S5.2.1. Signal on right side of mice

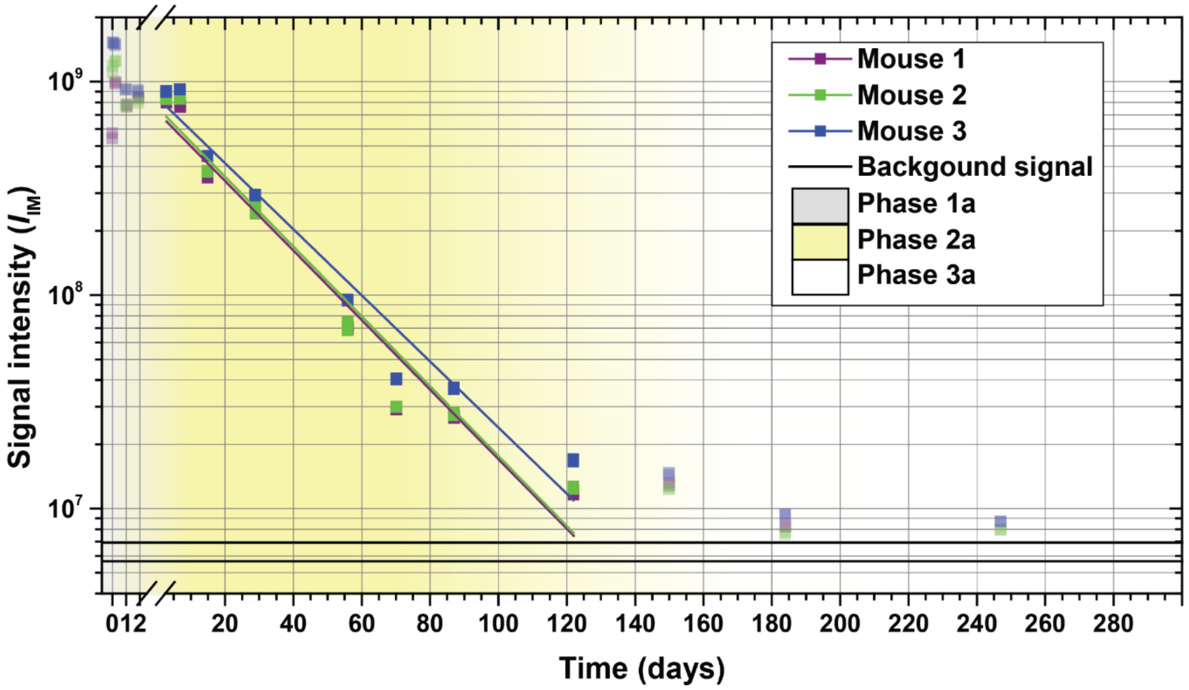

Figure S4. Signal (I_{IM}) of polymer **P1** as a function of time with noticeable three phases (**1a**, **2a**, and **3a**). The differences in fluorescence intensity between mice 1, 2 and 3 may be ascribed to the depth and shape of individual IM depots. Data in **Phase 2a** were fitted with an exponential function.

Phase 1a: **Depot maturation phase.** Depot maturation begins immediately after polymer administration and lasts for 2 to 4 days. During this phase, S_{IM} and V_{IM} grow due to polymer diffusion through the extracellular matrix, but polymer aggregation decreases the diffusion rate. Nearby cells take up a portion (*A*) of this polymer (as established in previous our study[21]), terminating depot expansion at $t_{S,\text{max}}$ because the polymers can no longer migrate freely through the tissues. The intracellular polymer fraction then enters **Phase 2a** - polymer dissolution, whereas the remaining polymer fraction (1 – *A*) is released into the bloodstream and delivered into secondary depots in kidneys/ liver and then eliminated (see *chapter S5.2.2*).

Paradoxically, in some mice, the signal increases (mouse 1 in **Figure S4**). When polymer is initially administered deep into mice's thigh, but polymer migrates through muscle *via* diffusion and thus moves closer to mice's surface (decreasing the shielding factor). As a result, in these mice, during **Phase 1a**, we observe signal increase. Nevertheless, in most mice, the initial elimination of polymer was stronger effect than the initial polymer diffusion, therefore, in this phase, we observed signal decrease in most mice.

Phase 2a: **Slow dissolution phase.** The slow dissolution phase begins 2-4 days after polymer injection and lasts up to several months. During this phase, cells slowly release polymers into the bloodstream, and I_{IM} decreases as a function of time, following first-order kinetics (**Table 2**). Additionally, S_{IM} and V_{IM} remain nearly constant (the polymer no longer diffuses through the tissues) and slowly decrease with the decrease in polymer concentration.

Phase 3a: **Terminal phase.** During the terminal phase, the signal as a function of time remains constant and equal to background noise because the polymers have been eliminated.

S5.2.2. Signal on left side of mice

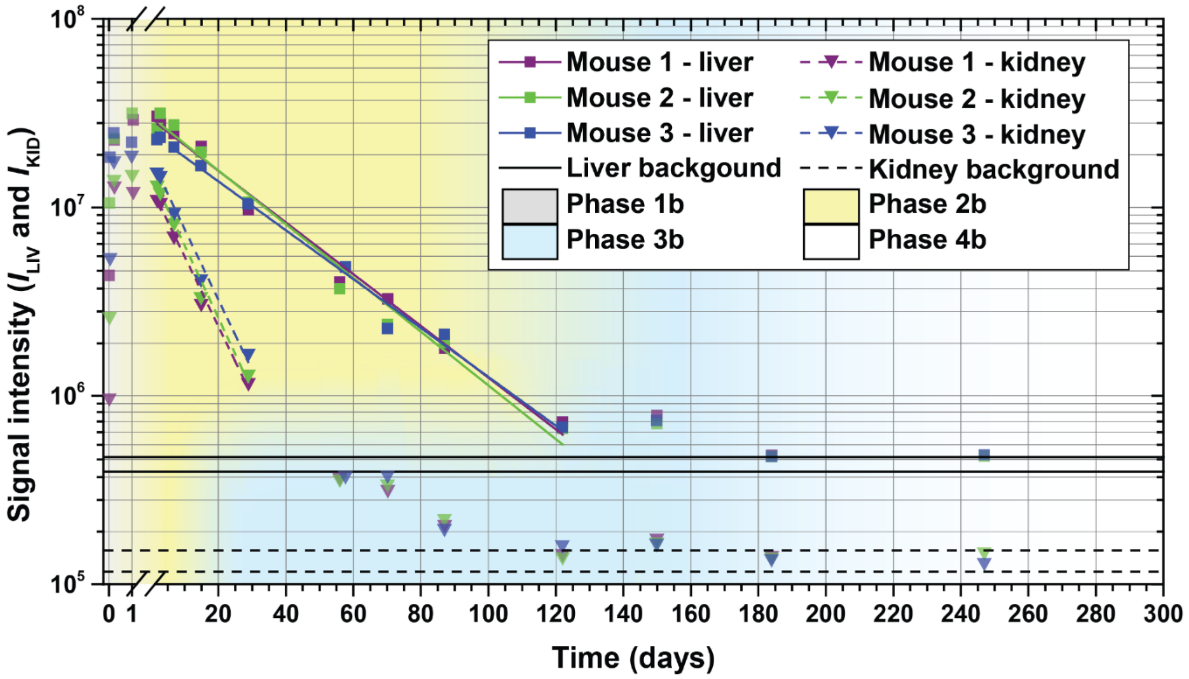

Figure S5. Signal of polymer **P1** as a function of time with noticeable four phases (**1b**, **2b**, **3b**, and **4b**). The differences in fluorescence intensity between mice 1, 2 and 3 may be ascribed to the depth and shape of individual IM depots. Data in **Phase 2b** were fitted with an exponential function.

Phase 1b: **Secondary depot formation phase.** This phase begins immediately after the polymer administration and lasts for *ca*. 2 days. During this phase, the secondary fluorescence signal grows, because the polymer is quickly released from the primary intramuscular depot into the bloodstream, from which the polymer is absorbed by the kidneys and liver.

Phase 2b: **Secondary depot dissolution phase.** This phase begins *ca*. 2 days after the administration and lasts for a few weeks (variable, depends on the amount of polymer in secondary depots). During this phase, the polymer is localized intracellularly in kidney and liver areas and the signal quickly decays following first-order kinetics with half-lives of *ca*. 8 to 45 days for kidney and 14 to 22 days for liver (**Table 2**).

Phase 3b: **Translucence phase.** This phase begins *ca*. 2 to 3 weeks after the administration and usually lasts weeks up to months. During this phase, the kidney and liver depots are nearly depleted, but residual IM signal (most noticeable in **F1** and **F2**, **Figure S62** and **Figure S63**) is still detectable within the entire left side of the mice. In other words, signal during this phase can be attributed the IM depot, not kidneys or liver.

Phase 4b: **Terminal phase.** During this phase, intramuscular, kidney and liver depots are depleted, and the signal is equal to background. This phase begins several months after the polymer administration (we observed this phase only in **P1**, **P2**, **E1**, **E2**).

S5.3. Evaluation of extensive parameters

These parameters (I_{IM}, S_{IM}, V_{IM}, I_{KID}, and I_{LIV}) provide the information about the quantity of the polymer. Therefore, their value as a function of time provides a direct information about the polymer depot dissolution kinetics. All data were linearized and fitted with a series of linear functions to avoid data overfitting.[27]

S5.3.1. Signal intensity in primary depot (I_{IM})

First, we measured the fluorescence intensity on the left side of the mouse (see *section S4.5*). Subsequently, we manually selected a selection of the image (rectangle with diameters 300 by 200 pixels) that contained the depot and surrounding tissues (examples are shown in **Figure S62**). Finally, we calculated the sum of all pixels with values higher than threshold value (200, *see section S5.4*) within the ROI, which provided us the I_{IM} value.

Data from polymer dissolution **Phase 2a** (see *section S5.2.1*) were described using **Equation 2a**:

$$I_{\mathrm{IM}} = I_{0,\mathrm{IM}} \cdot e^{-k_{I,\mathrm{IM}} \cdot t} \qquad (\mathbf{S2a})$$

where I_0 is the initial signal after the administration (signal amplitude), $k_{I,\mathrm{IM}}$ is the corresponding dissolution kinetics and t is the time past administration. **Equation S2a** was expressed in its linearized form **Equation S2b**:

$$\ln I_{\mathrm{IM}} = -k_{I,\mathrm{IM}} \cdot t + \ln I_{0,\mathrm{IM}} \qquad (\mathbf{S2b})$$

Therefore, we used natural logarithms of signal as a function of time was fitted with a linear function **Equation S2c**:

$$\ln I_{\mathrm{IM}} = a_{I,\mathrm{IM}} + b_{I,\mathrm{IM}} \cdot t \qquad (\mathbf{S2c})$$

where $a_{I,\mathrm{IM}}$ is the intercept of the linear fit and $b_{I,\mathrm{IM}}$ is the slope of the linear fit. Finally, we calculated k and I_0 with parameters from linear fit using **Equations S2d** and **S2e**:

$$k_{I,\mathrm{IM}} = -\, b_{I,\mathrm{IM}} \qquad (\mathbf{S2d})$$

$$I_{0,\mathrm{IM}} = e^{a_{I,\mathrm{IM}}} \qquad (\mathbf{S2e})$$

First, we linearized the data (*i.e.*, we calculated the natural logarithm of each value). Then, we disregarded the data measured at time 0, 4, 24 (and sometimes 48) hours; additionally, we disregarded the data from late phases of fitting (*i.e.*, those values that no longer followed the linear function, see **Figure S4**). Subsequently, we fitted the data using Equation S3c. Finally, the biological half-lives ($t_{1/2}$) were calculated with **Equation S9**.

S5.3.2. Primary depot size (S_{IM})

First, we measured the fluorescence intensity on the left side of the mouse (see *section S4.5*). Subsequently, we manually selected a selection of the image (rectangle with diameters 300 by 200 pixels) that contained the depot and surrounding tissues (examples are shown in **Figure S62**). Finally, we calculated the number of pixels within the FOV, whose value was higher than the threshold value (200, see *section S5.4*), which yielded the S_{IM} in pixels.

Data from polymer dissolution **Phase 2a** (see *section S5.2.1*) was described using **Equation 3a**:

$$S_{\mathrm{IM}} = S_{0,\mathrm{IM}} \cdot e^{-k_{S,\mathrm{IM}} \cdot t} \qquad (\mathbf{S3a})$$

where $S_{0,\mathrm{IM}}$ is the initial signal after the administration, $k_{S,\mathrm{IM}}$ is the corresponding dissolution kinetics, and t is the time past administration. **Equation S3a** was expressed in its linearized form **Equation S3b**:

$$\ln S_{\mathrm{IM}} = -k_{S,\mathrm{IM}} \cdot t + \ln S_{0,\mathrm{IM}} \qquad (\mathbf{S3b})$$

Therefore, the used natural logarithms of area as a function of time were fitted with linear function **Equation S3c**:

$$\ln S_{\mathrm{IM}} = a_{S,\mathrm{IM}} + b_{S,\mathrm{IM}} \cdot t \qquad (\mathbf{S3c})$$

where $a_{S,\mathrm{IM}}$ is the intercept of the linear fit and $b_{S,\mathrm{IM}}$ is the slope of the linear fit. Finally, we calculated k and I_0 with linear fit parameters using **Equations S3d** and **S3e**:

$$k_{S,\mathrm{IM}} = -\, b_{S,\mathrm{IM}} \qquad (\mathbf{S3d})$$
$$S_{0,\mathrm{IM}} = e^{a_{S,\mathrm{IM}}} \qquad (\mathbf{S3e})$$

First, we linearized the data (*i.e.*, we calculated the natural logarithm of each value). Then, we disregarded the data measured at time 0, 4, 24 (and sometimes 48) hours; additionally, we disregarded the data from late phases of fitting (*i.e.*, those values that no longer followed the linear function). Subsequently, we fitted the data using Equation S4c. Finally, the biological half-lives ($t_{1/2}$) were calculated with **Equation S9**.

S5.3.3. Depot volume (V_{IM})

These data originated from occasional photoacoustics measurements of the site of administration of the polymer (*section S4.3*).

Data from polymer dissolution **Phase 2a** (see *section S5.2.1*) were described using **Equation 4a**:

$$V_{\mathrm{IM}} = V_{0,\mathrm{IM}} \cdot e^{k_{V,\mathrm{IM}} \cdot t} \qquad (\mathbf{S4a})$$

where $V_{0,\mathrm{IM}}$ is the initial signal after the administration, $V_{\mathrm{noise,IM}}$ is noise values determined from control groups (see *section S5.4*; equal to 0.00 ± 0.00 mm^3), k is the corresponding dissolution kinetics and, t is the time past administration. **Equation S4a** was expressed in its linearized form **Equation S4b**:

$$\ln V_{\mathrm{IM}} = -k_{V,\mathrm{IM}} \cdot t + \ln V_{0,\mathrm{IM}} \qquad (\mathbf{S4b})$$

Therefore, we used natural logarithms of volume as a function of time as fitted with a linear function **Equation S4c**:

$$\ln V_{\mathrm{IM}} = a_{V,\mathrm{IM}} + b_{V,\mathrm{IM}} \cdot t \qquad (\mathbf{S4c})$$

where $a_{V,\mathrm{IM}}$ is the intercept of the linear fit and $b_{V,\mathrm{IM}}$ is the slope of the linear fit. Finally, we calculated k and I_0 with linear fit parameters using **Equations S4d** and **S4e**:

$$k_{V,\mathrm{IM}} = -\, b_{V,\mathrm{IM}} \qquad (\mathbf{S4d})$$
$$V_{0,\mathrm{IM}} = e^{a_{V,\mathrm{IM}}} \qquad (\mathbf{S4e})$$

First, we linearized the data (*i.e.*, we calculated the natural logarithm of each value). Then, we disregarded the data measured at time 0, 4, 24 (and sometimes 48) hours; additionally, we disregarded the data from late phases of fitting (*i.e.*, those values that no longer followed the linear function). Subsequently, we fitted the data using Equation S5c. Finally, the biological half-lives ($t_{1/2}$) were calculated with **Equation S9**.

S5.3.4. Signal intensity in primary depot (I_{KID})

First, we measured the fluorescence intensity on the right side of the mouse (see *section S4.5*). Subsequently, we manually selected an ellipsoid-shaped are in the kidney region (in all cases, this area contained 1264 pixels, which was experimentally determined to be sufficient to cover the kidney in all mouse, but the area was small enough not to include signal from "delocalized signal" from abdomen, see fluorescence signal distribution in **Figure S62** and **Figure S63**). Finally, we calculated the sum of all pixel values within the FOV, which yielded I_{KID} in pixels.

Data from polymer dissolution **Phase 2b** (see *section S5.2.2*) was described using **Equation 5a**:

$$I_{\text{KID}} = I_{0,\text{KID}} \cdot e^{-k_{I,\text{KID}} \cdot t} \qquad (\mathbf{S5a})$$

where I_0 is the initial signal after the administration (signal amplitude), k is the corresponding dissolution kinetics, and t is the time past administration. **Equation S5a** was expressed in its linearized form **Equation S5b**:

$$\ln I_{\text{KID}} = -k_{I,\text{KID}} \cdot t + \ln I_{0,\text{KID}} \qquad (\mathbf{S5b})$$

Therefore, we used natural logarithms of signal as a function of time was fitted with linear function **Equation S5c**:

$$\ln I_{I,\text{KID}} = a_{I,\text{KID}} + b_{I,\text{KID}} \cdot t \qquad (\mathbf{S5c})$$

where $a_{I,\text{KID}}$ is the intercept of the linear fit and $b_{I,\text{KID}}$ is the slope of the linear fit. Finally, we calculated k and I_0 using **Equations S5d** and **S5e**:

$$k_{I,\text{KID}} = -\, b \qquad (\mathbf{S5d})$$

$$I_{0,\text{KID}} = e^{a} \qquad (\mathbf{S5e})$$

First, we linearized the data (*i.e.*, we calculated the natural logarithm of each value). Then, we disregarded the data measured at time 0, 4, 24 (and sometimes 48) hours; additionally, we disregarded the data from late phases of fitting (*i.e.*, those values that no longer followed the linear function, see **Figure S5**). Subsequently, we fitted the data using Equation S6c. Finally, the biological half-lives ($t_{1/2}$) were calculated using **Equation S9**.

S5.3.5. Signal intensity in primary depot (I_{LIV})

First, we measured the fluorescence intensity on the right side of the mouse (see *section S4.5*). Subsequently, we manually selected an ellipsoid-shaped are in the liver region (in all cases, this area contained 3922 pixels, which was experimentally determined to be sufficient to cover the liver in all mouse, but the area was small enough not to include signal from "delocalized signal" from abdomen, see fluorescence signal distribution in **Figure S62** and **Figure S63**). Finally, calculated the sum of all pixel values within the FOV, which yielded in I_{LIV} in pixels.

Data from polymer dissolution **Phase 2b** (see *section S5.2.2*) can be described using **Equation 6a**:

$$I_{\mathrm{LIV}} = I_{0,\mathrm{LIV}} \cdot e^{-k_{I,\mathrm{LIV}} \cdot t} \quad (\mathbf{S6a})$$

where I_0 is the initial signal after the administration (signal amplitude), k is the corresponding dissolution kinetics, and t is the time past administration. **Equation S6a** were expressed in its linearized form **Equation S6b**:

$$\ln I_{\mathrm{LIV}} = -k_{I,\mathrm{LIV}} \cdot t + \ln I_{0,\mathrm{LIV}} \quad (\mathbf{S6b})$$

Therefore, we used natural logarithms of signal as a function of time was fitted with linear function **Equation S6c**:

$$\ln I_{\mathrm{LIV}} = a_{I,\mathrm{LIV}} + b_{I,\mathrm{LIV}} \cdot t \quad (\mathbf{S6c})$$

where $a_{I,\mathrm{LIV}}$ is the intercept of the linear fit and $b_{I,\mathrm{LIV}}$ is the slope of the linear fit. Finally, we calculated k and I_0 using **Equations S6d** and **S6e**:

$$k_{I,\mathrm{LIV}} = -\, b_{I,\mathrm{LIV}} \quad (\mathbf{S6d})$$
$$I_{0,\mathrm{LIV}} = e^{a_{I,\mathrm{LIV}}} \quad (\mathbf{S6e})$$

First, we linearized the data (*i.e.*, we calculated the natural logarithm of each value). Then, we disregarded the data measured at time 0, 4, 24 (and sometimes 48) hours; additionally, we disregarded the data from late phases of fitting (*i.e.*, those values that no longer followed the linear function, see **Figure S5**). Subsequently, we fitted the data using Equation S3c. Finally, the biological half-lives ($t_{1/2}$) were calculated using **Equation S9**.

S5.4. Determination of auxiliary parameters

S5.4.1. Noise threshold

We derived the threshold value for noise from the signal values in **DMSO group**. The mean ± SD signal in **DMSO group** was 100.18 ± 26.17, the lowest value was 39.05, the highest value was 203.41, the lowest quartile (25%) was 87.30, the middle quartile (50%) was 98.56, and the upper quartile (75%) was 112.32. With these values in mind, we arbitrarily chose to set a threshold for noise to 200, which was sufficiently high to filter the background signal noise, but low enough not to interfere with fluorescence polymer depot (whose values ranged from 200 to 70,000).

S5.4.2. Additional auxiliary parameters

We determined values of auxiliary parameters ($I_{\mathrm{noise,IM}}$, $S_{\mathrm{noise,IM}}$, $V_{\mathrm{noise,IM}}$, $I_{\mathrm{noise,KID}}$, and $I_{\mathrm{noise,LIV}}$), which enable the assessment of the background signal (noise). Their values were determined as mean values of all mice in control groups (**DMSO group** and **saline group**). Their values (mean ± SD) were:

$$I_{\mathrm{noise,IM}} = 6290766 \pm 621150\ \mathrm{a.u.}\ \text{(for fluorescence imaging)}$$
$$I_{\mathrm{noise,IM,PH}} = 0.0620 \pm 0.0651\ \mathrm{a.u.}\ \text{(for photoacoustics)}$$
$$S_{\mathrm{noise,IM}} = 6607 \pm 1715\ \text{pixels}$$
$$V_{\mathrm{noise,IM}} = 0.00 \pm 0.00\ \mathrm{mm}^3$$
$$I_{\mathrm{noise,KID}} = 134015 \pm 17300\ \mathrm{a.u.}$$
$$I_{\mathrm{noise,LIV}} = 434784 \pm 39161\ \mathrm{a.u.}$$

S5.5. Biological half-lives and statistical evaluations

We performed all statistical analyses (Dixon's Q-test, Grubbs' test, correlation matrices) using build-in functions in OriginPro 2018 (64-bit, SR1, b9.5.1.159; OriginLab, Northampton, MA, USA).

S5.5.1. Dissolution kinetics

Fitted $k_{i,T}$ and $\ln I_{0,i,T}$ values of Mouse i (i = 1, 2, and 3), in tissue depot T (T = intramuscular (IM), kidney (KID), liver (LIV)) were tested for outliers by Dixon's Q-test and Grubbs' test, for each polymer, in each tissue depot T (intramuscular (IM), kidney (KID), liver (LIV)), in range of i = (1,3), with statistical significance 0.95. We omit outlier values hereafter.

S5.5.2. Dissolution kinetics

To obtain representative values for dissolution kinetics, we calculated $\bar{k}_T$, means of $k_{i,T}$ of Mouse i, in i = 1, 2, and 3, in tissue depot T, T = intramuscular (IM), kidney (KID), liver (LIV); weighted by the number of fitted points, and corresponding (pooled) standard deviations $s_{k,T}$ using **Equations S7** and **S8** respectively.

$$\bar{k}_T = \frac{\sum N_{i,T} \cdot k_{i,T}}{\sum N_{i,T}} \quad (\mathbf{S7})$$

where $N_{i,T}$ are the number of fitted points; and $k_{i,T}$ are the dissolution kinetics observed in Mouse i, in i = 1, 2, 3, in tissue depot T = intramuscular (IM), kidney (KID), liver (LIV).

$$s_{\bar{k},T} = \sqrt{\frac{\sum (N_{i,T} - 1) \cdot s_{k,i,T}^2}{\sum (N_{i,T} - 1)}} \quad (\mathbf{S8})$$

where $N_{i,T}$ are the number of fitted points and $s_{k,i,T}$ are standard deviations of $k_{i,T}$ values observed in Mouse i, in i = 1, 2, 3, in tissue depot T = intramuscular (IM), kidney (KID), liver (LIV). The results of $\bar{k}_T$ and $s_{\bar{k},T}$ are shown in *section S14*. Lastly, the mean biological half-lives ($\bar{t}_{1/2,T}$) were calculated by **Equation S9**:

$$\bar{t}_{1/2,T} = \frac{\ln 2}{\bar{k}_T} \quad (\mathbf{S9})$$

Standard deviations of $\bar{t}_{1/2,T}$ ($\sigma_{\bar{t}_{1/2,T}}$) were estimated by propagation of uncertainty formula for $f(x) = a \cdot A^b$ (**Equation S10)**, where A are the expectation values and a and b are constants:

$$\sigma_{\bar{t}_{1/2,T}} = \left| \frac{\bar{t}_{1/2,T} \cdot (-1) \cdot s_{k,T}}{\bar{k}_T} \right| \quad (\mathbf{S10})$$

Similarly, we evaluated the values of the fitted intercept $\ln I_{0,i,T}$ by calculating their N-weighted means $\ln \bar{I}_{0,T}$ and pooled standard deviations $s_{\ln \bar{I},T}$ by **Equations S7** and **S8** respectively.

We calculated mean initial fluorescence signal amplitudes ($\bar{I}_{0,T}$) using **Equations S2e**, **3e**, **4e**, **5e**, and **6e** (*section S14*).

Standard deviations of $\bar{I}_{0,T}$ ($\sigma_{\bar{I}_{0,T}}$) were estimated by propagation of uncertainty formula for $f(x) = ae^{bA}$ (**Equation S11)**, where A are the expectation values and a and b are constants:[28,29]

$$\sigma_{\bar{I}_{0,T}} = |\bar{I}_{0,T}||s_{\ln \bar{I},T}| \qquad \textbf{(S11)}$$

S5.5.3. Kidney and liver accumulation factors (f_{KID} and f_{LIV})

We defined reduced tissue depot initial signal amplitudes using **Equation S12:**

$$\bar{I}'_{0,T} = \bar{I}_{0,T} - I_{\text{noise},T} \qquad \textbf{(S12)}$$

where $\bar{I}_{0,T}$ are the mean initial fluorescence signal amplitudes and $\bar{I}_{0,T}$ are background (noise) levels in tissue depot T (T = kidney (KID), liver (LIV), and intramuscular (IM)), see *section S5.4.2.*

We defined kidney and liver accumulation factors (f_{KID} and f_{LIV}) using **Equation S13** and **S14:**

$$f_{\text{KID}} = \frac{\bar{I}'_{0,\text{KID}}}{\bar{I}'_{0,\text{IM}}} \qquad \textbf{(S13)}$$

$$f_{\text{LIV}} = \frac{\bar{I}'_{0,\text{LIV}}}{\bar{I}'_{0,\text{IM}}} \qquad \textbf{(S14)}$$

Additionally, we calculated the polymer relative affinities to the kidneys and liver ("selectivity") for **each mouse** as a ratio of $f_{\text{LIV}}/f_{\text{KID}}$, as defined in **Equation S15:**

$$f_{\text{LIV/KID}} = \frac{\bar{I}'_{0,\text{LIV}}}{\bar{I}'_{0,\text{KID}}} = \frac{f_{\text{LIV}}}{f_{\text{KID}}} \qquad \textbf{(S15)}$$

Standard deviations of $\bar{I}'_{0,T}$, f_{KID}, f_{LIV} and $f_{\text{LIV/KID}}$ were estimated by propagation of uncertainty formulas for $f(x) = A - B$ and $f(x) = A/B$ (**Equations S16 and S17)**, where A and B are the expectation values:[28,29]

$$\sigma_{\bar{I}'_{0,T}} = \sqrt{s^2_{\bar{I}_{0,T}} + s^2_{\text{noise},T} - 2 \cdot s_{\bar{I}_{0,T}} \cdot s_{I_{\text{noise},T}}} \qquad \textbf{(S16)}$$

$$\sigma_{f(\frac{T_A}{T_B})} = \sqrt{\left(\frac{\sigma_{\bar{I}'_{0,T_A}}}{\bar{I}'_{0,T_A}}\right)^2 + \left(\frac{\sigma_{\bar{I}'_{0,T_B}}}{\bar{I}'_{0,T_B}}\right)^2 - \frac{2 \cdot \sigma_{\bar{I}'_{0,T_A}} \cdot \sigma_{\bar{I}'_{0,T_B}}}{(\bar{I}'_{0,T_A}) \cdot (\bar{I}'_{0,T_B})}} \qquad \textbf{(S17)}$$

where T_A and T_B are tissue depots: intramuscular (IM), kidney (KID), liver (LIV) and $f(\frac{T_A}{T_B})$ accumulation factors of their respective combinations as per **Equations S13**, **S14** and **S15**.

S5.6. Evaluation of depot density (K_{10})

We calculated distribution index (K_{10}) to describe the distribution of signal within the polymer depot. This parameter provides the percentage of signal localized in the top 10% most active pixels (90th percentile) of the depot. This parameter is always smaller or equal to 100% (uneven depot distribution) but higher or equal to 10% (for those depots where all pixels have equal signal values). The use of this parameter is demonstrated in **Figure S6**: the depot in **A** is concentrated and localized only in a relatively small spot (and K_{10} is relatively high), whereas the depot in **B** is more diffuse and spread over a large area of the mice (and K_{10} is relatively low). K_{10} data is available in *section S12.1.4.*

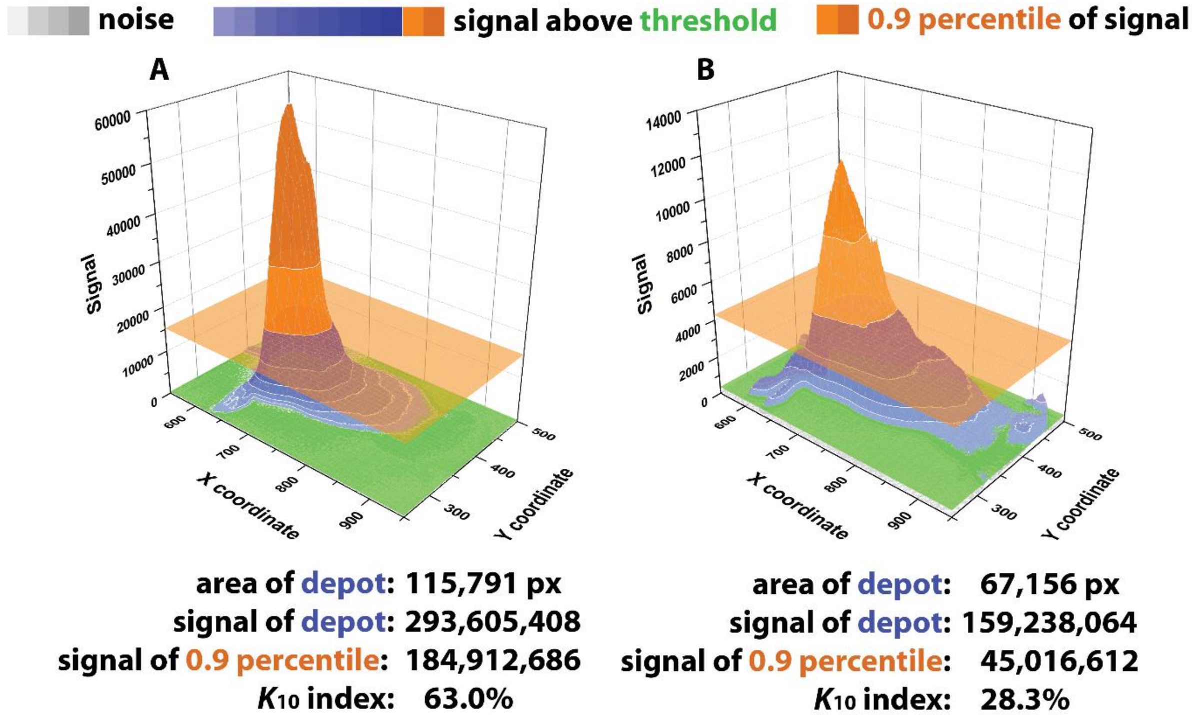

Figure S6. Two examples of spatial distribution (X and Y coordinate) of fluorescence intensity (Z coordinate) in mice with two different depot densities (**pNIPAM** in **A**; **pDFEA** in **B**); highest signal intensity corresponds to the site of administration (thigh muscle). Pixels with intensities below **threshold** (**green plane**) are shown in gray and were not used in calculation of depot densities (they were regarded as noise). Pixels with intensities above **threshold** were shown in **blue** (intensity percentile 0.0 to 0.9) or **orange** (intensity percentile 0.9 to 1.0). Subsequently, we calculated (a) number of pixels with intensities above the threshold (**area of depot**) and (b) sum of intensity in all pixels with intensities above the threshold (**signal of depot**). Afterwards, we calculated the sum of signals in top-10% most intensive signals (**signal of 0.9 percentile**). Finally, calculated K_{10} index as a ratio of [**signal of 0.9 percentile**]/[**signal of depot**].

To compare densities of IM depots of various polymers, we compared the K_{10} indexes of these depots in various timepoints after the polymer administration (**Figure S7**). Note, that for first 3 days after administration, K_{10} describes the spatial biodistribution of both intracellular and extracellular polymer. However, by day 7, the diffusion stopped and all polymer signal originates from polymers internalized in cells, and thus K_{10} index described the density of intracellular depots. Noteworthily, K_{10} in all depots decreased as a function of time after day 7 (**Figure S7**), because the areas with highest polymer concentrations released the polymer most quickly (first-order kinetics of release), effectively decreasing the differences in polymer concentration throughout the depot. Arbitrarily, depots with K_{10} above 50% were considered dense, while those with K_{10} below 50% were considered diffuse. These data show that **I1**, **I2**, **E1**, **E2** and **Cy7** formed dense depots (highest being in **E2**), while **F1**, **F2**, **P1** and **P2** formed diffuse depots (most diffuse being in **F1** and **F2**).

In most cases, increasing molar mass increased depot densities, which can be ascribed to decrease of T_{CP} of these polymers and thus more extensive polymer aggregation, which in turn decreases polymer diffusion and causes higher densities of polymer depots.

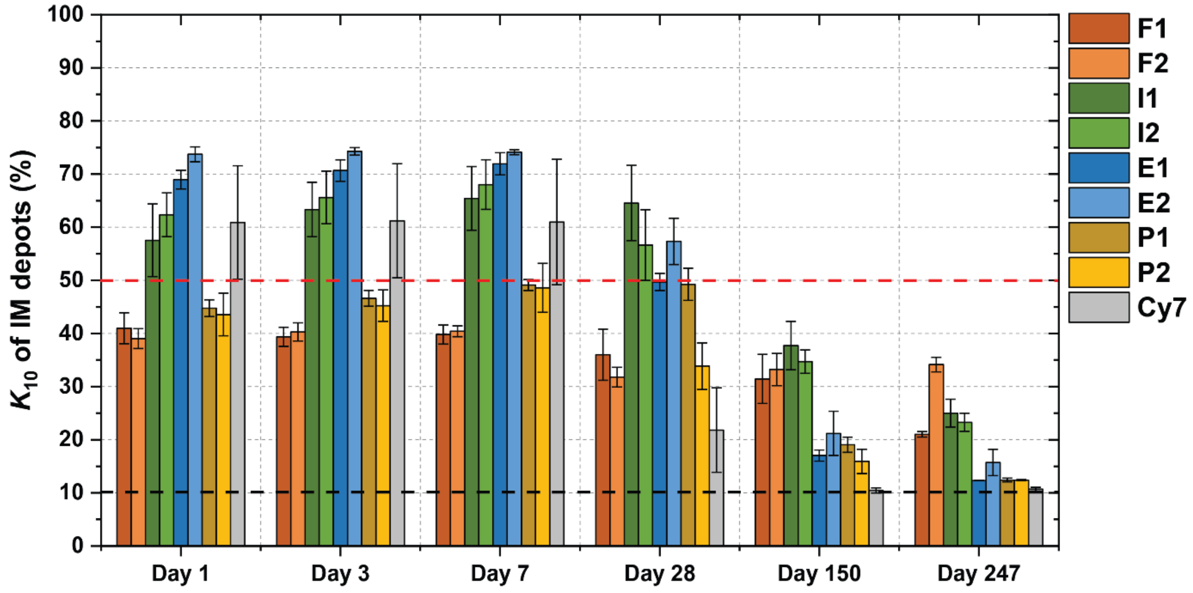

Figure S7. K_{10} of muscle depots as a function of time at selected timepoints; data expressed as mean ± SD. **Red line** represents arbitrary threshold of 50% for dense ($K_{10} \geq 50\%$) or diffuse depots ($K_{10} < 50\%$); **black line** represents lowest possible K_{10} (10 %). K_{10} data is available in *section S12.1.4*.

In **DMSO** and **saline groups**, K_{10} ($K_{\mathrm{background,IM}}$) was equal to 10.2719 ± 0.3215 % (mean ± SD).

S5.7. Data processing

All data were processed in Microsoft Office 365 Pro Plus 16.0 (Microsoft, Redmond, USA), OriginPro 8.6.0 (OriginLab Corporation, Northampton, USA) and R 4.0.2 (RStudio, Boston, USA). The schemes and structures were drawn in ChemDraw Professional 16.0.1.4 (77) (Perkin Elmer Informatics, Inc., Waltham, USA). The graphics were processed in Adobe Illustrator CS6 16.0.0 (Adobe Systems Inc., San Jose, USA).

For initial data processing and analysis, Python 3.8 and Jupyter notebook[30] were used together with the following packages: Matplotlib v3.4,[31] NumPy v1.22,[32] pandas v1.2.4,[33] h5py v3.3,[34] SciPy v1.7.3,[35] Statmodels v0.13,[36] seaborn v11.2,[37] and Pillow.Image v8.3.1.[38] Python code together with details about the data processing and analysis are available at https://github.com/jankaWIS/code2pharmacokinetics.

S6. Histopathological examination

S6.1. Method

At the end of the long-term experiment (day 202 past administration), one mouse from each group has been euthanized *via* cervical dislocation (in mild isoflurane anesthesia). Their organs were visually inspected for abnormalities and samples of their liver, kidneys, hearts, injected thigh muscle and contralateral thigh muscle were collected within minutes after the sacrifice. They were fixed using 4% formaldehyde solution and stored in dark in a refrigerator (4 °C) until processed further.

The samples were washed in PBS and a routine histological examination was performed. Samples were embedding in paraffin and sectioned for 4 or 7 μm histological sections using Leica microtome RM2245 (Leica Biosystems, Wetzlar, Germany). Sections were stained using hematoxylin and eosin; representative sections were also stained using Van Gieson's stain. The staining was performed according to distributors guidelines (for staining details see **Table S5** and **Table S6**); Canada balsam was used for slide mounting. Finally, we observed the microscope slides using microscopy.

An overview of histopathological findings can be found in **Table S93**, examples of micrographs are shown in **Figure S110** and **Figure S111**.

Table S5. The report detailed description of hematoxylin & eosin staining protocol. Asterisk (*****) indicates that a solution from staining kit (H&E Fast Staining Kit Art. No. 9194) was used without.

**Hematoxylin & Eosin Staining Protocol**		
step	compound/procedure (concentration %)	approximate time
1	Xylene (100%)	5 min
2	Xylene (100%)	5 min
3	Ethanol (100%)	2 min
4	Ethanol (100%)	2 min
5	Ethanol (95%)	2 min
6	Distilled water wash	2 min
7	**Hematoxylin (< 0.5%)***	3 min
8	Distilled water wash	30 s
9	Hydrochloric acid in ethanol (0.5%)	2 s
10	Running water washing	1 min
11	Ethanol (95%)	1 min
12	**Eosin (1%)***	90 s
13	Ethanol (95%)	1 min
14	Ethanol (95%)	1 min
15	Ethanol (100%)	1 min
16	Acetone (100%)	5 min
17	Xylene (100%)	5 min
18	Xylene (100%)	5 min
19	Mounting: Canada balsam	N/A

Table S6. The report detailed description of Van Gieson' staining protocol. Asterisk (*) indicates that a solution from staining kit (Van Gieson's Trichrome Staining Kit 9193.1) was used without.

Van Gieson's staining protocol		
step	compound/procedure (concentration %)	approximate time
1	Xylene (100%)	5 min
2	Xylene (100%)	5 min
3	Ethanol (100%)	2 min
4	Ethanol (96%)	2 min
5	Ethanol (80%)	2 min
6	Ethanol (70%)	2 min
7	**Solution A+B (1:1)***	5 min
8	Distilled water wash	2 min
9	Hydrochloric acid in ethanol (0.5%)	2 s
10	Running water washing	10 min
11	**Van Gieson solution***	2 min
12	Ethanol (70%)	2 s
13	Ethanol (80%)	2 s
14	Ethanol (96%)	2 s
15	Ethanol (95%)	2 s
16	Ethanol (100%)	2 s
17	Xylene (100%)	5 min
18	Xylene (100%)	5 min
19	Mounting: Canada balsam	N/A

S7. Physiological model

S7.1. Presumptions

Our model was built based on data in our study and in previous studies[21,39–47] and it is based on five assumptions:

1) Every phase transition between two compartments follows a first-order kinetics.
2) The intensity of the fluorescence signal is linearly proportional to the amount of polymer.
3) The intensity of the fluorescence signal is affected only by the polymer concentration and the depth of the depot beneath the animal's skin (due to shielding). The transition of the polymer from one compartment to another (*e.g.* from extracellular to intracellular space) does not increase nor decrease the fluorescence signal.
4) Auto-fluorescence of the mice flesh is constant and much lower than fluorescence of dyes.
5) The kinetics follows the suggested multiple-compartment model as suggested in **Figure S8**.

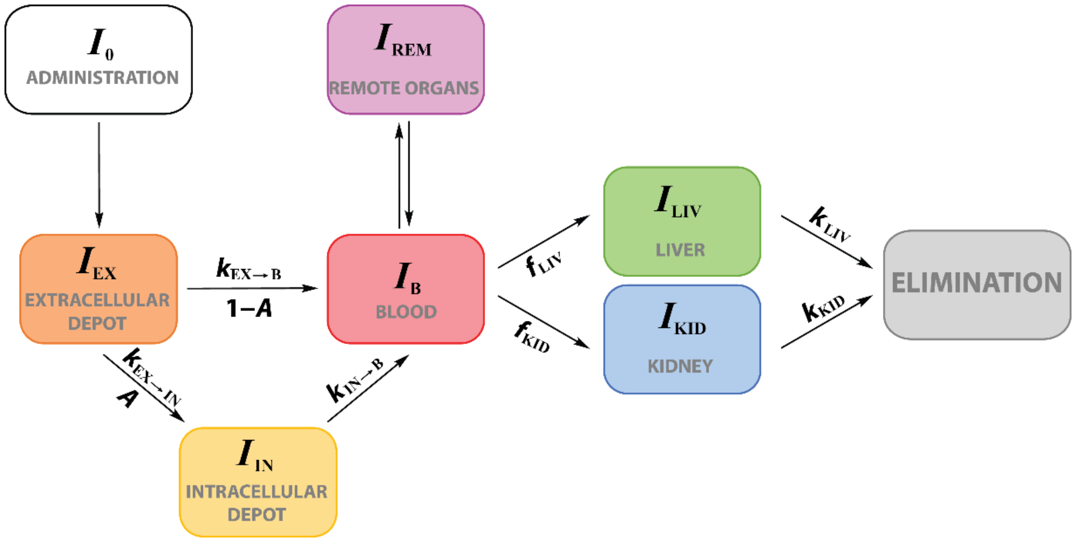

Figure S8. Scheme of proposed pharmacokinetics model

S7.2. Model description

In the beginning of the experiment, amount of polymer (I_0) is administered into extracellular space, from where it is parallelly absorbed by cells (with kinetics determined by $k_{\mathrm{EX}\rightarrow\mathrm{IN}}$) cleared into bloodstream or lymphatic system (with kinetics determined by $k_{\mathrm{EX}\rightarrow\mathrm{B}}$). Thus, **Equations S18a** and **S18b** can be written:

$$\frac{\mathrm{d}I_{\mathrm{EX}}}{\mathrm{d}t} = -k_{\mathrm{EX}\rightarrow\mathrm{B}} - k_{\mathrm{EX}\rightarrow\mathrm{IN}} \qquad \textbf{(S18a)}$$

$$I_{\mathrm{EX}} = I_0 \cdot \mathrm{e}^{-(k_{\mathrm{EX}\rightarrow\mathrm{B}} + k_{\mathrm{EX}\rightarrow\mathrm{IN}})t} \qquad \textbf{(S18b)}$$

Where t is time. Additionally, as polymer cellular uptake ($k_{\mathrm{EX}\rightarrow\mathrm{IN}}$) and elimination into bloodstream ($k_{\mathrm{EX}\rightarrow\mathrm{B}}$) are parallel (competing) processes, the fraction of the polymer that enters intracellular depot (A) can be expressed with **Equation S19**:

$$A = \frac{k_{\mathrm{EX}\rightarrow\mathrm{IN}}}{k_{\mathrm{EX}\rightarrow\mathrm{IN}} + k_{\mathrm{EX}\rightarrow\mathrm{B}}} \qquad \textbf{(S19)}$$

Conversely, the fraction of polymer that enters bloodstream is equal to 1 – A.

The amount of polymer in intracellular depot (I_{IN}) at quickly increases due to absorption of polymer ($k_{\mathrm{EX\rightarrow IN}}$) and then slowly decreases (phase of slow exocytosis). The exocytosis and endocytosis of polymer by cells are an equilibrium process, but a portion of polymer is slowly cleared from extracellular depot into bloodstream, shifting the equilibrium towards polymer eliminations. Because the exocytosis is much slower than endocytosis, the concentration of polymer in extracellular matrix during later phase is low. In previous article,[1] we demonstrated that polymers in low polymer concentrations bind to local proteins increasing their hydrophilicity and T_{CP}. As a result, the rate of absorption in the latter phase is **not** equal to the rate of polymer absorption immediately after administration ($k_{\mathrm{EX\rightarrow IN}}$). Nevertheless, the dissolution in intracellular depot can be characterized with a single constant ($k_{\mathrm{IN\rightarrow B}}$) in **Equations S20**:

$$I_{\mathrm{IN}} = I_0 A(\mathrm{e}^{-k_{\mathrm{IN\rightarrow B}}t} - \mathrm{e}^{-k_{\mathrm{EX\rightarrow IN}}t}) = I_0 \frac{k_{\mathrm{EX\rightarrow IN}}}{k_{\mathrm{EX\rightarrow IN}} + k_{\mathrm{IN\rightarrow B}}}(\mathrm{e}^{-k_{\mathrm{IN\rightarrow B}}t} - \mathrm{e}^{-k_{\mathrm{EX\rightarrow IN}}t}) \quad \textbf{(S20)}$$

A portion of polymer (amount equal to $I_0 \cdot (1 - A)$) enters the bloodstream, from where polymers form secondary depots in remote organs, namely in liver and kidneys (and possibly other organs). The initial release of polymer from IM depot ($k_{\mathrm{EX\rightarrow B}}$) is a predominant source of polymer in these organs, the amount of polymer released from intracellular depot ($k_{\mathrm{IN\rightarrow B}}$) is negligible (because $k_{\mathrm{IN\rightarrow B}} \ll k_{\mathrm{EX\rightarrow B}}$) and thus will not be considered in following model.

The intensity of the signal observed in each organ depends on the organs' ability to uptake this polymer, most likely proportional to the blood-flow rate per organ weight ratio. Nevertheless, the organs can be shielded by the surrounding tissue, organs situated in greater depth or shielded behind dense material (such as skull) may display decreased signal. Therefore, the empirical factors f_{KID} and f_{LIV} are functions of polymer accumulation in these organs, but also shielding factors. As a result, these factors cannot be used directly to calculate the amounts of polymers in each organ.

The amount of polymer in liver and kidney depot initially increases due to blood-borne polymer ($k_{\mathrm{EX\rightarrow B}}$), and then decreases due to polymer elimination from these organs (k_{KID} and k_{LIV}).

$$I_{\mathrm{KID}} = I_0 f_{\mathrm{KID}}(1 - A)(\mathrm{e}^{-k_{\mathrm{EX\rightarrow B}}t} - \mathrm{e}^{-k_{\mathrm{KID}}t}) \quad \textbf{(S21a)}$$
$$I_{\mathrm{LIV}} = I_0 f_{\mathrm{LIV}}(1 - A)(\mathrm{e}^{-k_{\mathrm{EX\rightarrow B}}t} - \mathrm{e}^{-k_{\mathrm{LIV}}t}) \quad \textbf{(S21b)}$$

S7.3. Applied model

Based on our proposed model and its formulas (*section S7.2*), we calculated a presumed biodistribution of polymer **P1** (as a model polymer) as a function of time (**Figure 5**). In this calculation, we used predominantly empirically determined kinetics constants (*section S5* and **Table S7**). However, note that some values (A, f_{KID}, and f_{LIV}) may be distorted due to variable levels of signal shielding in organs.

Values of some parameters ($k_{\mathrm{EX\rightarrow B}}$ and $k_{\mathrm{EX\rightarrow IN}}$) cannot be estimated from our data directly (other imaging techniques, such as ^{19}F MRI may be more useful[21]), however, edified guess can be made based on:

(a) $A = \frac{k_{\mathrm{EX\rightarrow IN}}}{k_{\mathrm{EX\rightarrow IN}} + k_{\mathrm{EX\rightarrow B}}}$

(b) intramuscular extracellular depot was depleted (*i.e.* less than 5% of polymer remained site of administration) within two days.

Table S7. List of parameters of pharmacokinetics of **P1** that were used in calculation of biodistribution model shown in **Figure 5** in main article. f_{KID} was multiplied by 2 because kidneys are pair organs.

Symbol	Value	Determination
I_0	1.000	arbitrarily chosen as 1.00 (model was normalized to its maxima)
A	0.75	this value was estimated from IM depot dissolution data (**Figure S70**)
$k_{\mathrm{EX}\rightarrow\mathrm{B}}$	0.4 d^{-1}	estimate based on **Equation S18b** and **S19** and *A*
$k_{\mathrm{EX}\rightarrow\mathrm{IN}}$	1.2 d^{-1}	estimate based on **Equation S18b** and **S19** and *A*
$k_{\mathrm{IN}\rightarrow\mathrm{B}}$	0.042 d^{-1}	determined from long-term **muscle** depot dissolution data (*section S5.3.1*)
k_{LIV}	0.087 d^{-1}	determined from **liver** depot dissolution data (*section S5.3.5*)
k_{KID}	0.031 d^{-1}	determined from **kidney** depot dissolution data (*section S5.3.4*)
$2f_{\mathrm{KID}}$	0.082	ratio of initial signals in **liver** and muscle depots (*section S5.5.3*)
f_{LIV}	0.057	ratio of initial signals in **liver** and muscle depots (*section S5.5.3*)

RESULTS & RAW DATA SECTION

S8. Polymer characterization

S8.1. Polymer characterization – NMR spectra of polymers

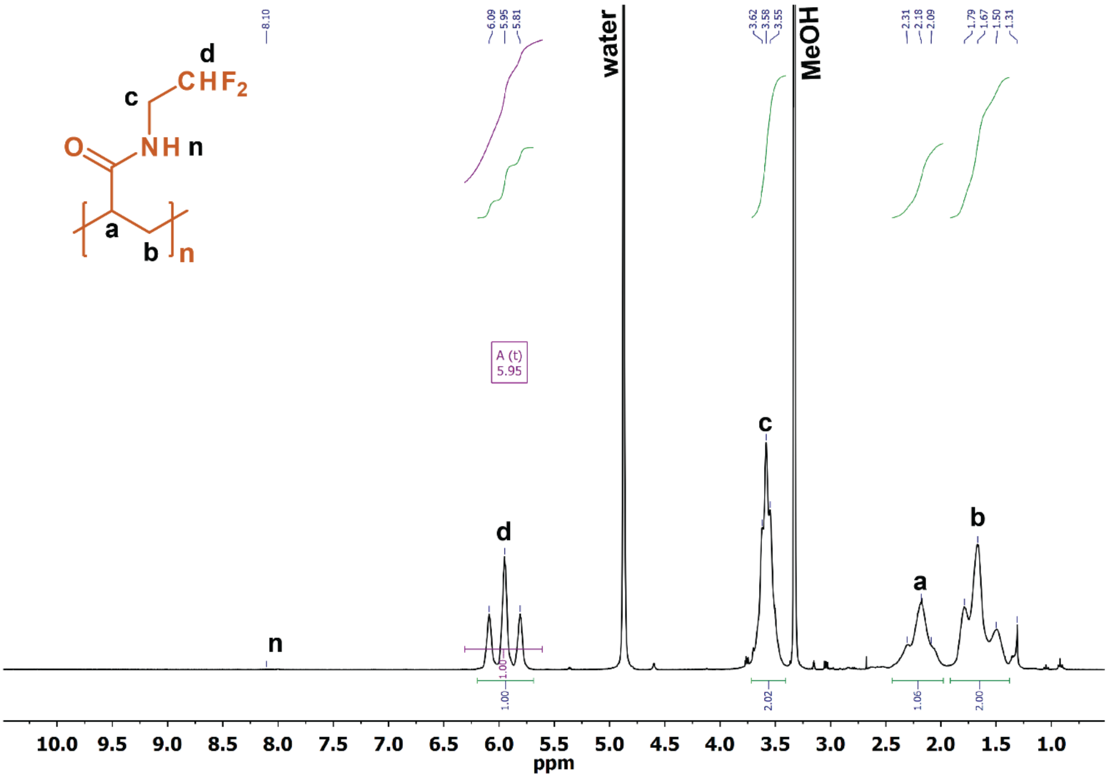

Figure S9. ^{1}H NMR spectrum of **F1** in MeOH before conjugation with fluorescent label

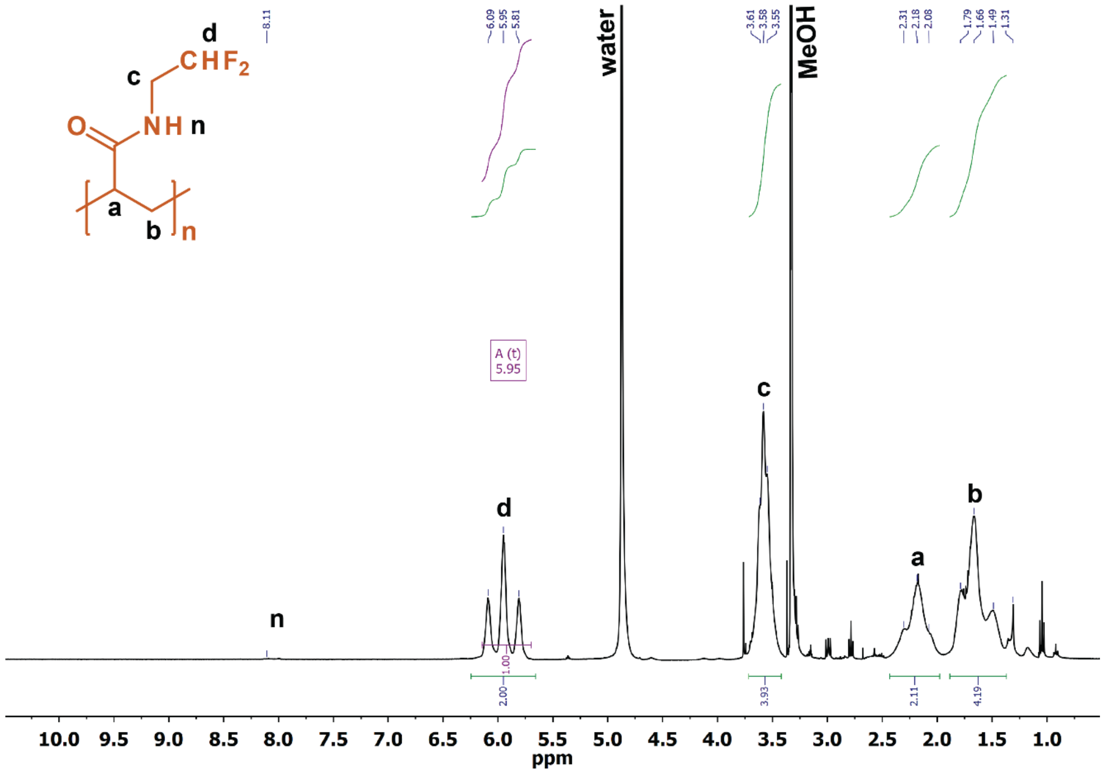

Figure S10. ^{1}H NMR spectrum of **F2** in MeOH before conjugation with fluorescent label

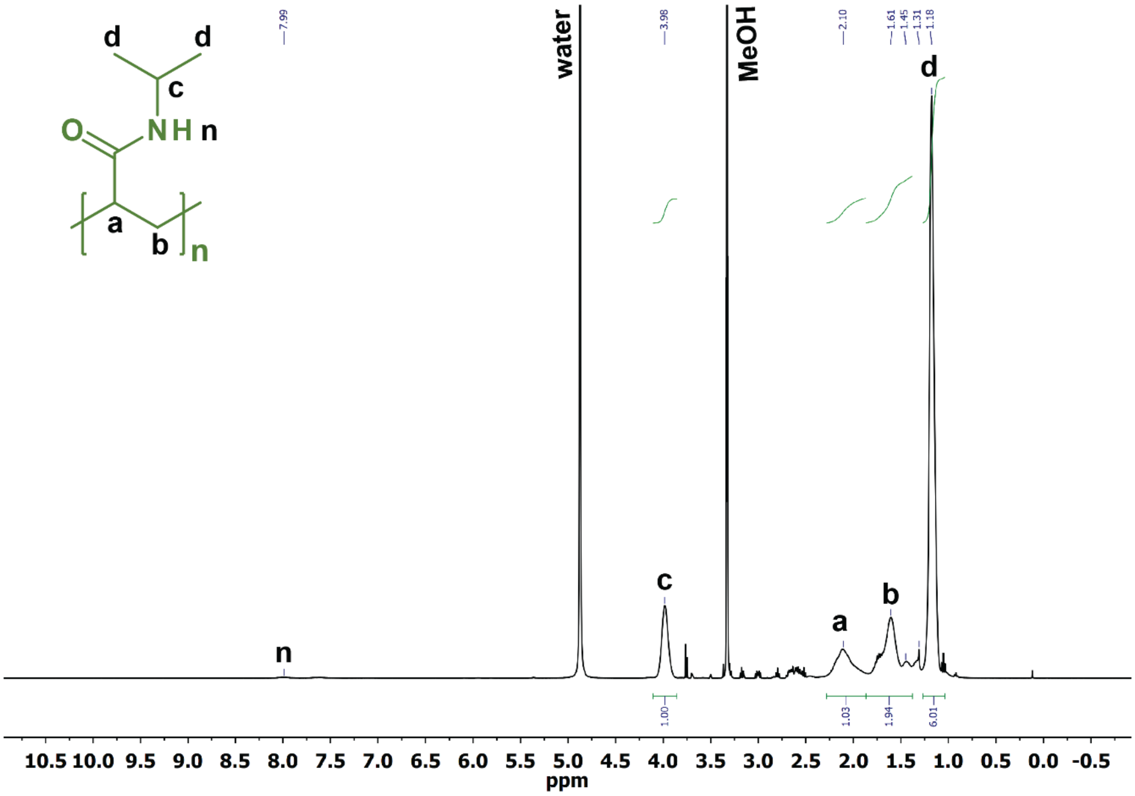

Figure S11. ^{1}H NMR spectrum of **I1** in MeOH before conjugation with fluorescent label

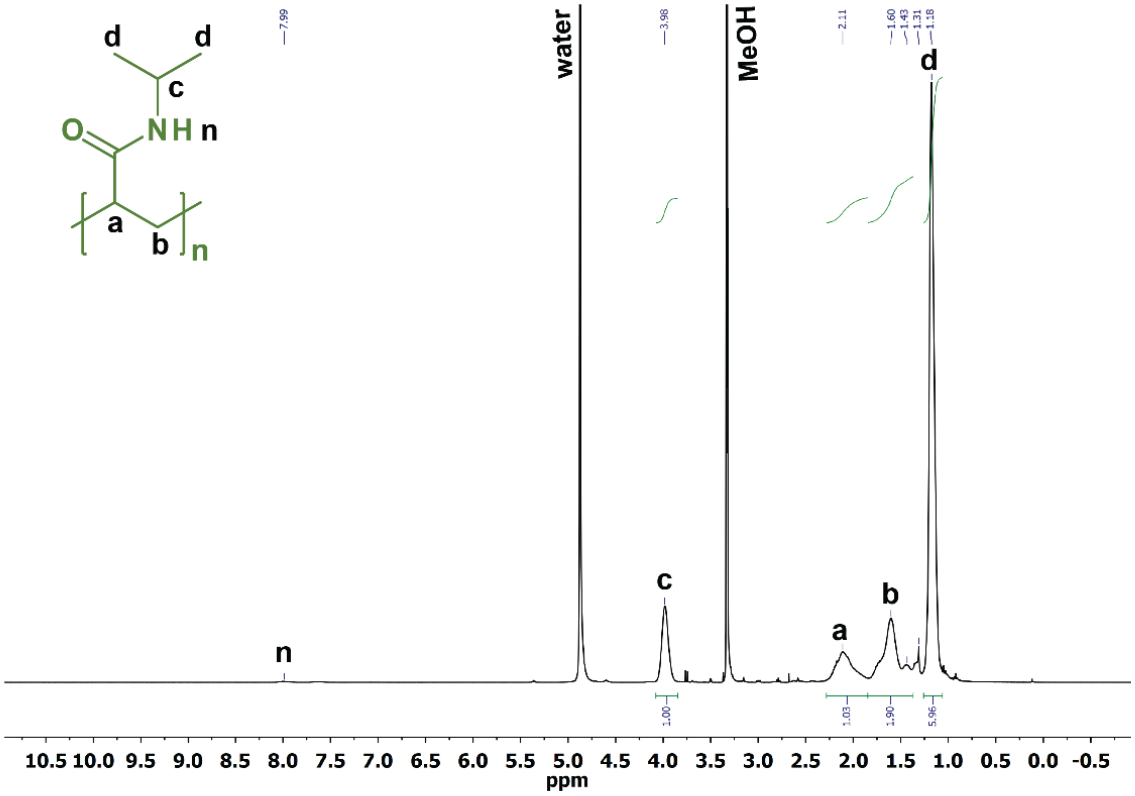

Figure S12. ^{1}H NMR spectrum of **I2** in MeOH before conjugation with fluorescent label

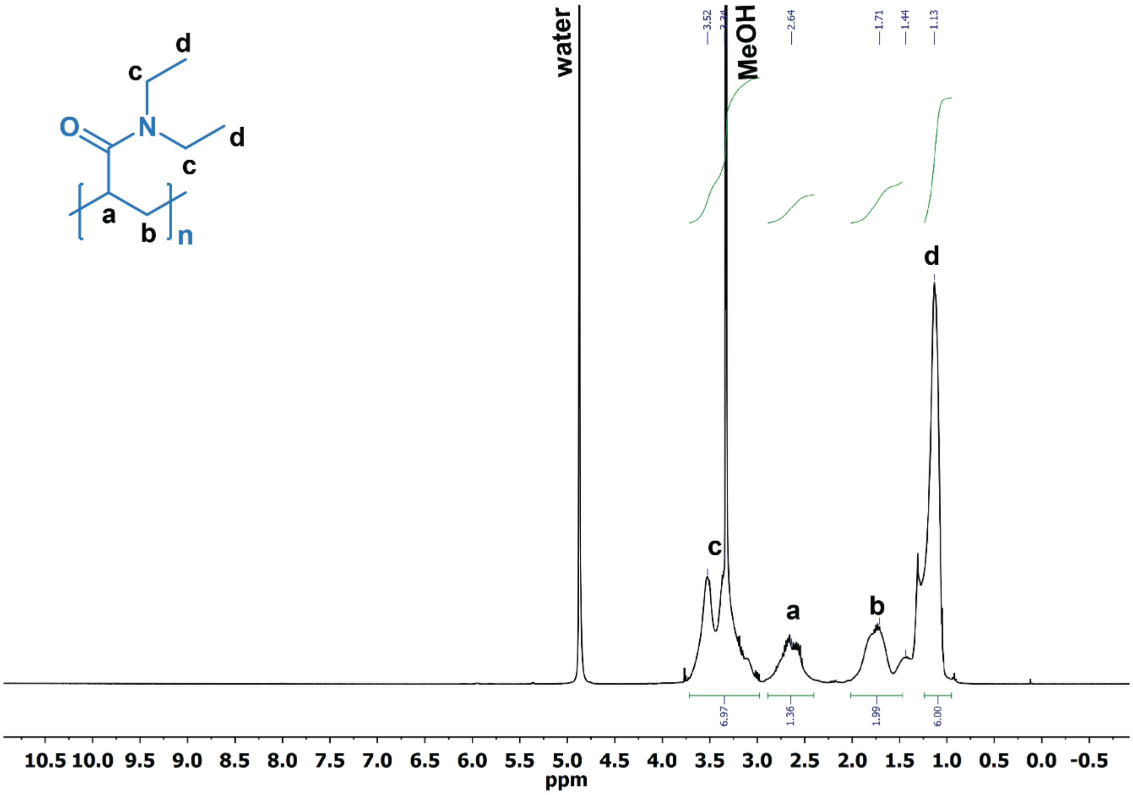

Figure S13. ^{1}H NMR spectrum of **E1** in MeOH before conjugation with fluorescent label

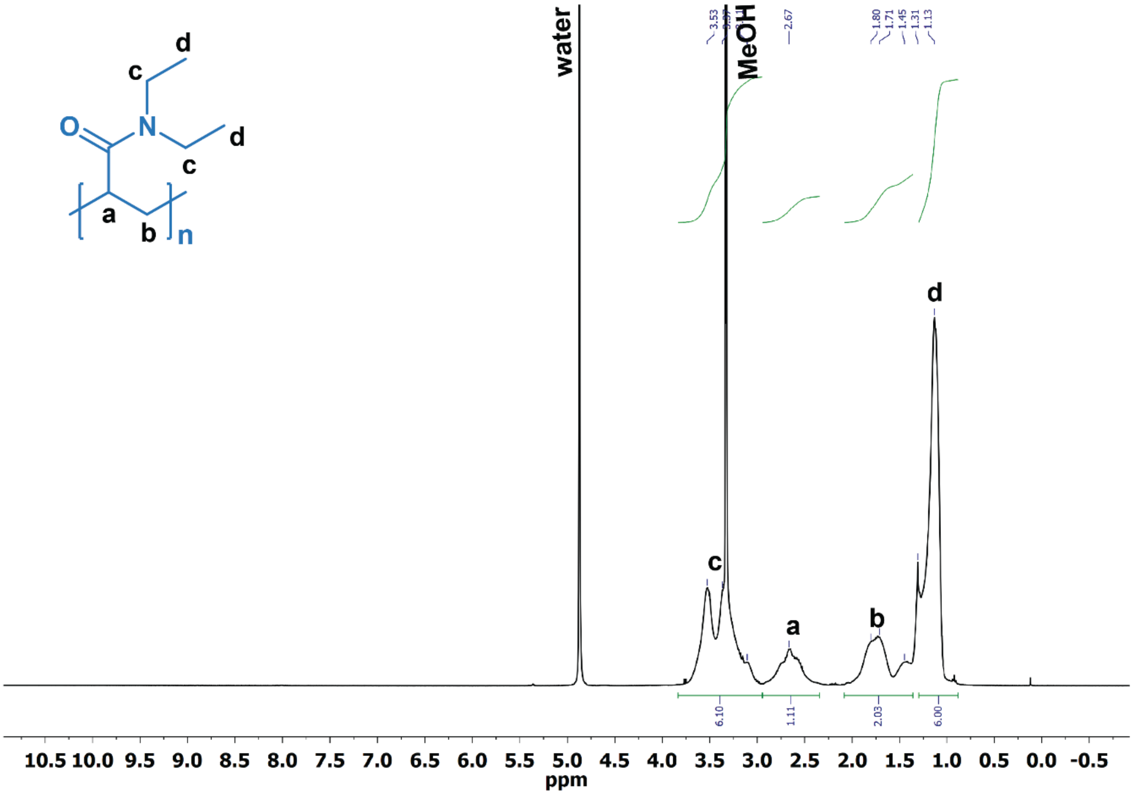

Figure S14. ^{1}H NMR spectrum of **E2** in MeOH before conjugation with fluorescent label

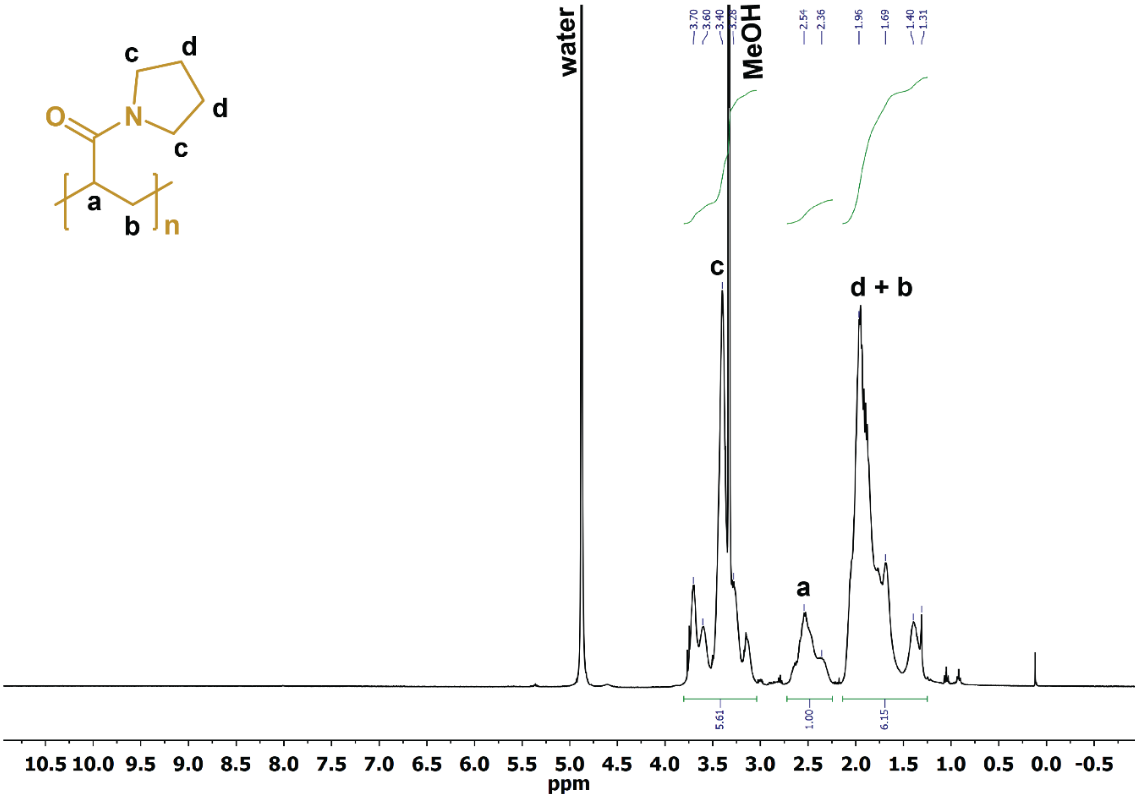

Figure S15. ^{1}H NMR spectrum of **P1** in MeOH before conjugation with fluorescent label

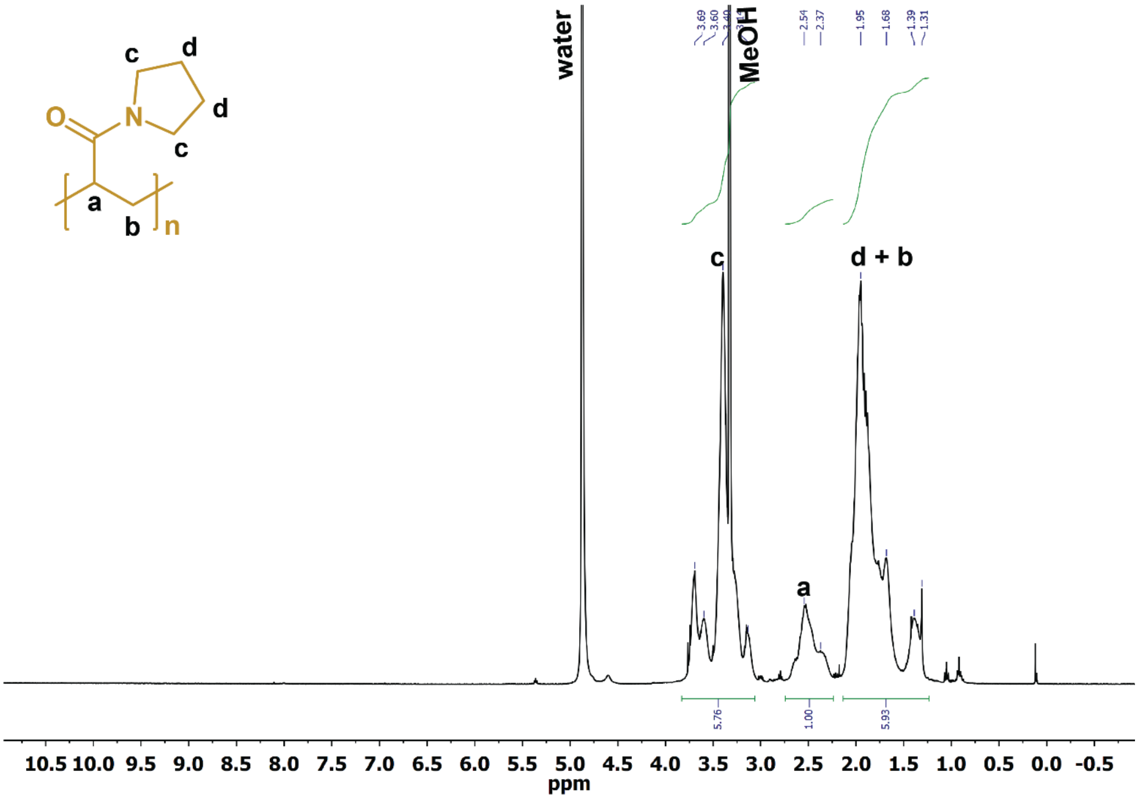

Figure S16. ^{1}H NMR spectrum of **P2** in MeOH before conjugation with fluorescent label

S8.2. Polymer characterization – size exclusion chromatography (SEC)

Subsequently, we used size exclusion chromatography to measure the masses (M_w and M_n) and polydispersity index $Đ_M$ (M_w/M_n); the results were shown in **Table S8**, SEC traces are shown in **Figure S17** to **Figure S20**. For details, see our previous article.[1]

Table S8. Molar masses (M_w and M_n), dispersity index $Đ_M$ (M_w/M_n), and T_{CP} in water and in FBS, as described in our previous article.[1]

Polymer		M_w* (kg/mol)	M_n* (kg/mol)	$Đ_M$* (M_w/ M_n)	$T_{CP, H2O}$⁺ (°C)	$T_{CP, FBS}$⁺ (°C)
pDFEA	**F1**	26.2	24.2	1.08	27.1 ± 0.6	22.6 ± 0.1
	**F2**	36.2	35.1	1.03	34.0 ± 0.9	30.1 ± 0.2
pNIPAM	**I1**	20.2	19.6	1.03	30.0 ± 0.3	25.7 ± 0.1
	**I2**	31.6	30.8	1.03	31.4 ± 0.2	24.4 ± 0.1
pDEA	**E1**	22.3	21.2	1.06	29.3 ± 0.0	24.4 ± 0.1
	**E2**	34.7	31.7	1.09	30.2 ± 0.1	25.8 ± 0.2
pAP	**P1**	19.6	17.6	1.11	58.1 ± 0.1	56.7 ± 0.3
	**P2**	36.0	32.9	1.09	56.8 ± 0.4	48.6 ± 0.1

*Determined by size exclusion chromatography. ⁺Determined by turbidimetry at c_{pol} = 10 mg/mL.

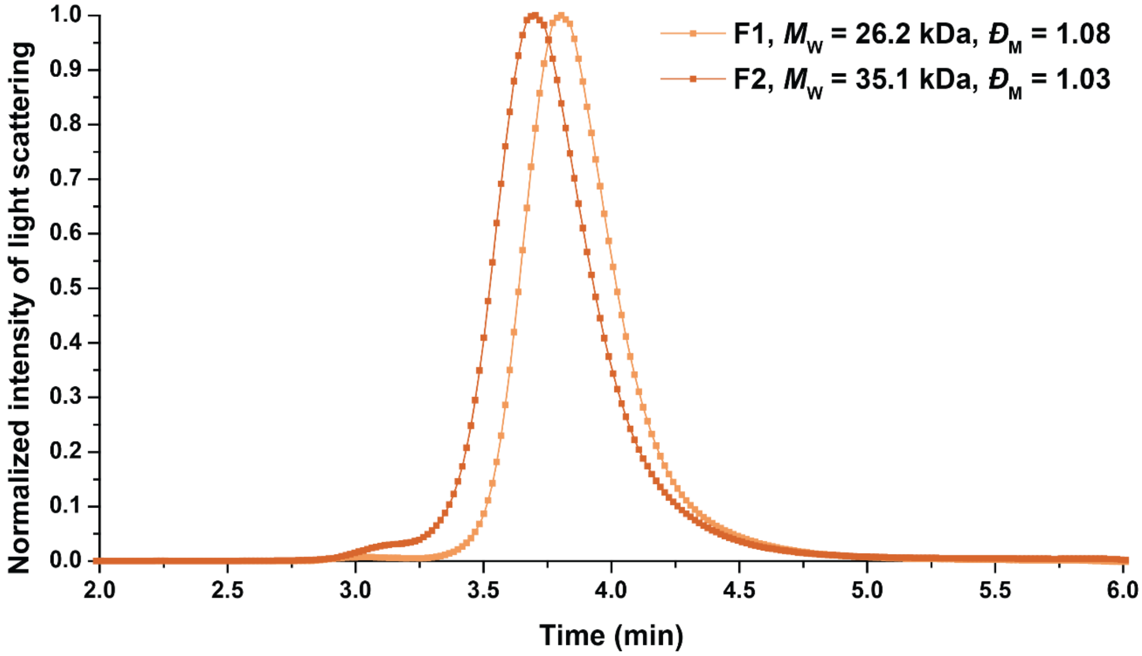

Figure S17. Size exclusion chromatograms of **pDFEA** polymers; see our previous article for details.[1] These data were subsequently used to determine the molar masses M_w, M_n, and dispersity ($Đ_M$) of the polymers. Note, that these SEC were measured before conjugating the polymers with fluorescent label.

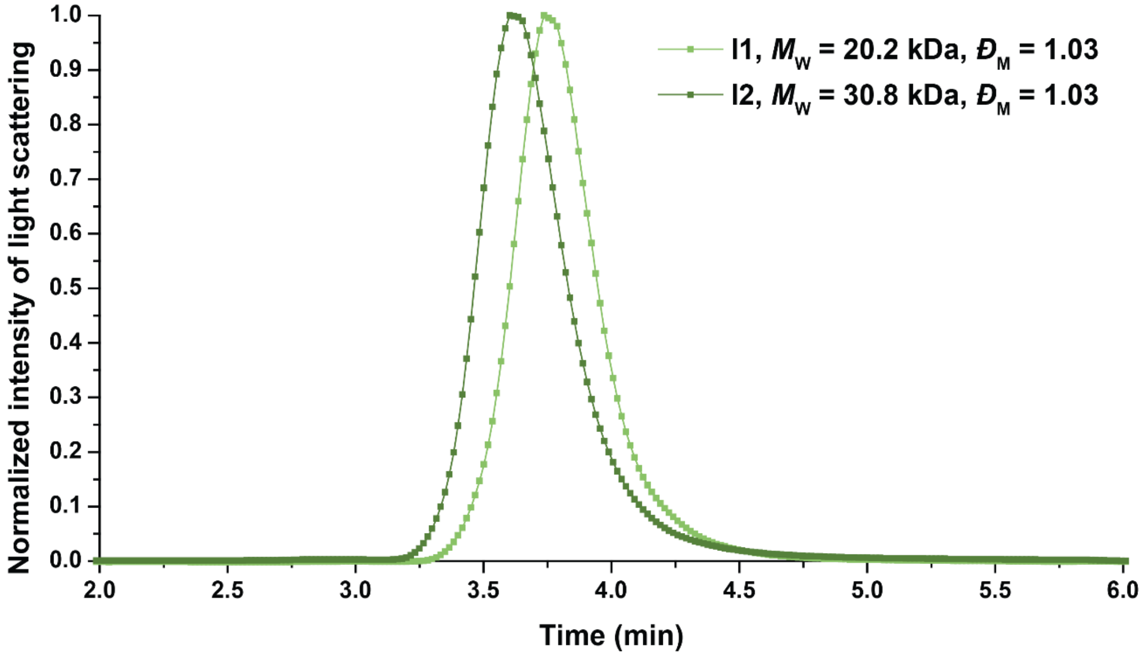

Figure S18. Size exclusion chromatograms of **pNIPAM** polymers; see our previous article for details.[1] These data were subsequently used to determine the molar masses M_w, M_n, and dispersity ($Đ_M$) of the polymers. Note, that these SEC were measured before conjugating the polymers with fluorescent label.

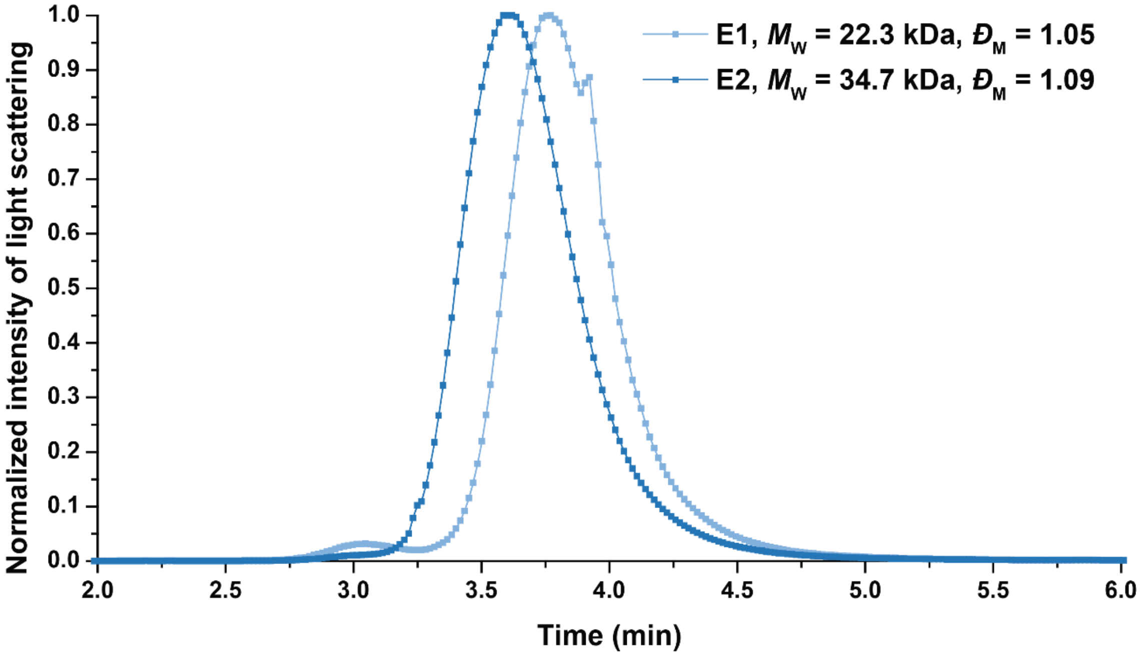

Figure S19. Size exclusion chromatograms of **pDEA** polymers; see our previous article for details.[1] These data were subsequently used to determine the molar masses M_w, M_n, and dispersity ($Đ_M$) of the polymers. Note, that these SEC were measured before conjugating the polymers with fluorescent label.

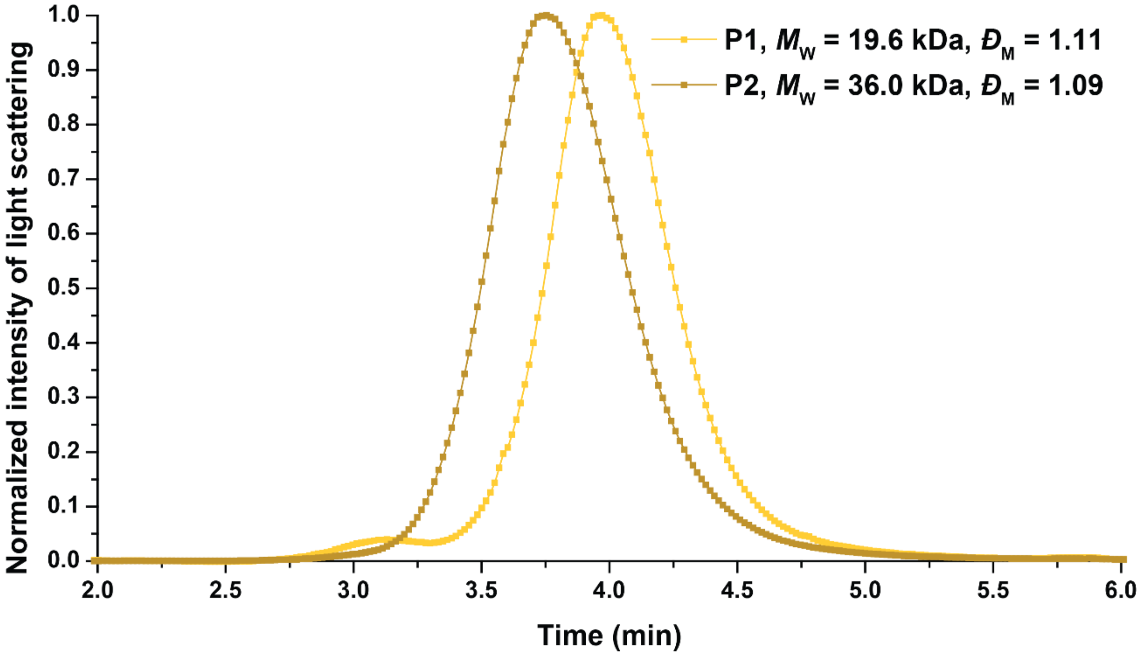

Figure S20. Size exclusion chromatograms of **pAP** polymers; see our previous article for details.[1] These data were subsequently used to determine the molar masses M_w, M_n, and dispersity ($Đ_M$) of the polymers. Note, that these SEC were measured before conjugating the polymers with fluorescent label.

S8.3. Differential scanning calorimetry – results

We measured the thermal properties of polymers, because glass-point transition can affect polymers' dissolution rates.

First, we measured the of pure (neat, bulk) polymers. This property is straightforward to quantify, however, its information is relatively limited because water (as well as other compounds, such as proteins) can act as plasticizers[9–11] and thus lower the T_g *in vivo*. Nevertheless, DSC data revealed that while bulk polymer **E** had significantly lower T_g than the remaining polymers (**Table S9**), which indicated that **E** may be more less prone to vitrify than the remaining polymers.

To investigate the thermal properties of polymers in more relevant environment, we studied the vitrification of phase-separated polymers in aqueous environment, following the method proposed by Van Durme (2004) on **pNIPAM** polymer.[9] In this study, authors demonstrated that polymer vitrification induces a change (a decrease) of polymer heat capacity, thereby altering the slope of heat flow as a function of temperature. Regrettably, this method does not provide a specific T_g values of samples as it provides only whether the T_g is above samples' T_{CP} (an so we observe a change of slope) or the T_g is below the samples' T_{CP} (an so we observe no change of slope). Our **pNIPAM** samples exhibited very similar behavior to that shown in Van Durme study, corroborating that our **pNIPAM** samples vitrify above T_{CP} (*ca*. 35 °C, close to body temperature). We observed similar behavior in **pDFEA** (at *ca*. 35 °C) and **pAP** (*ca*. 50 °C) indicating that both these polymers vitrify under given conditions. Noteworthily, **pAP** phase separates only above 50 °C, therefore at body temperature it remains soluble and non-aggregates. On the other hand, the thermal behavior of phase separated **pDFEA** was different from that of the remaining polymers: its aggregation peak was very broad (the peak broadness was identical even with lower the heating rate; data not shown) and the slopes before and after the peak were very similar. This led us to a conclusion that the phase separation "aggregation" of **pDFEA** is followed by slow processes (possibly arrangement of polymer chains), that, furthermore, show no sign of vitrification.

Thus, these findings support our suggestion that polymers **pDFEA** and **pNIPAM** both phase-separate and vitrify at body temperature, while polymer **pDFEA** phase separates but does not vitrify and neither **pAP** phase separates, nor vitrifies. Nevertheless, we acknowledge that these hypotheses should be confirmed with a broader study of various polymers.

S8.3.1. Glass point temperature (T_g) of pure (bulk) materials

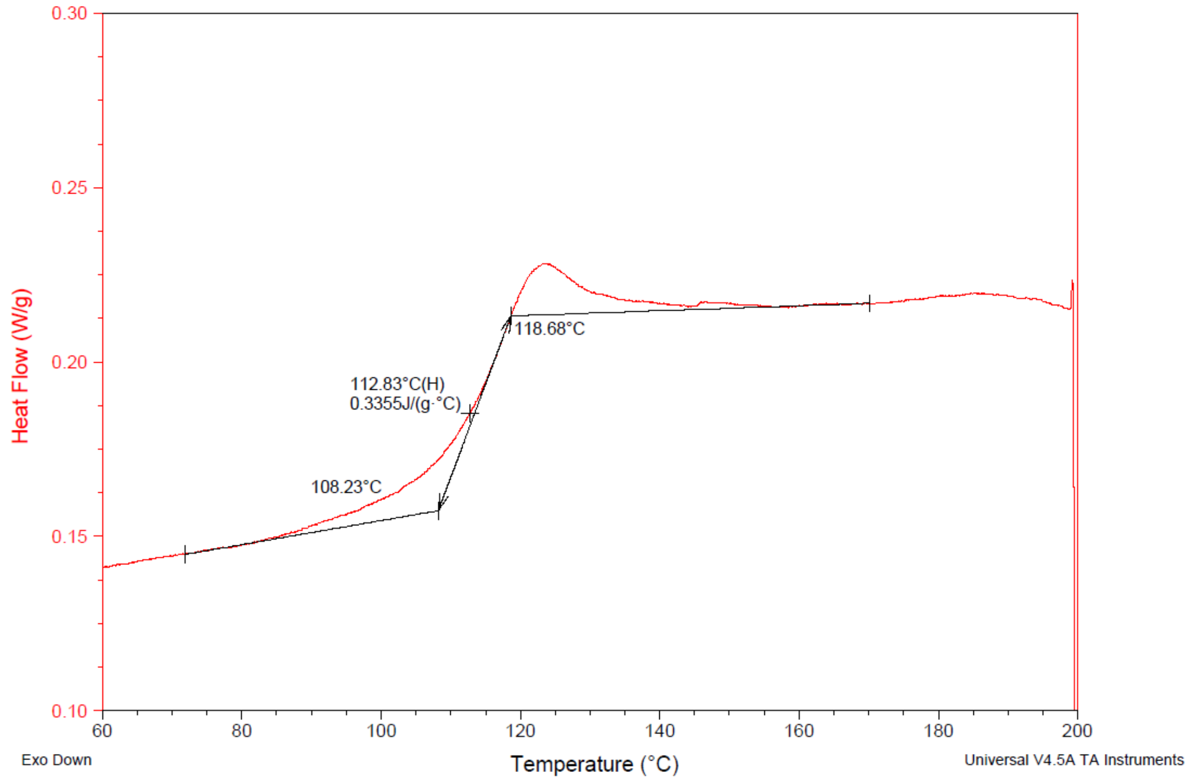

Figure S21. Glass point measurement of pure polymer **F2**; glass point was *ca*. 113 °C.

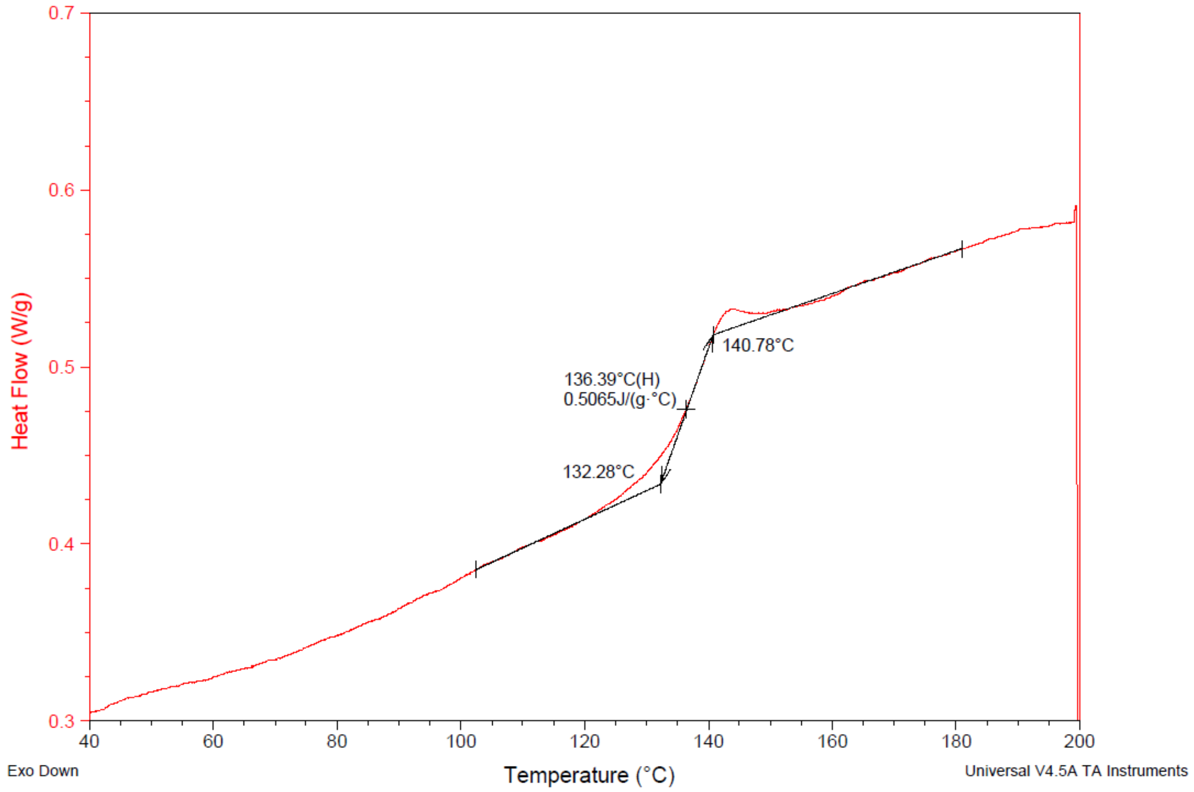

Figure S22. Glass point measurement of pure polymer **I2**; glass point was *ca*. 136 °C.

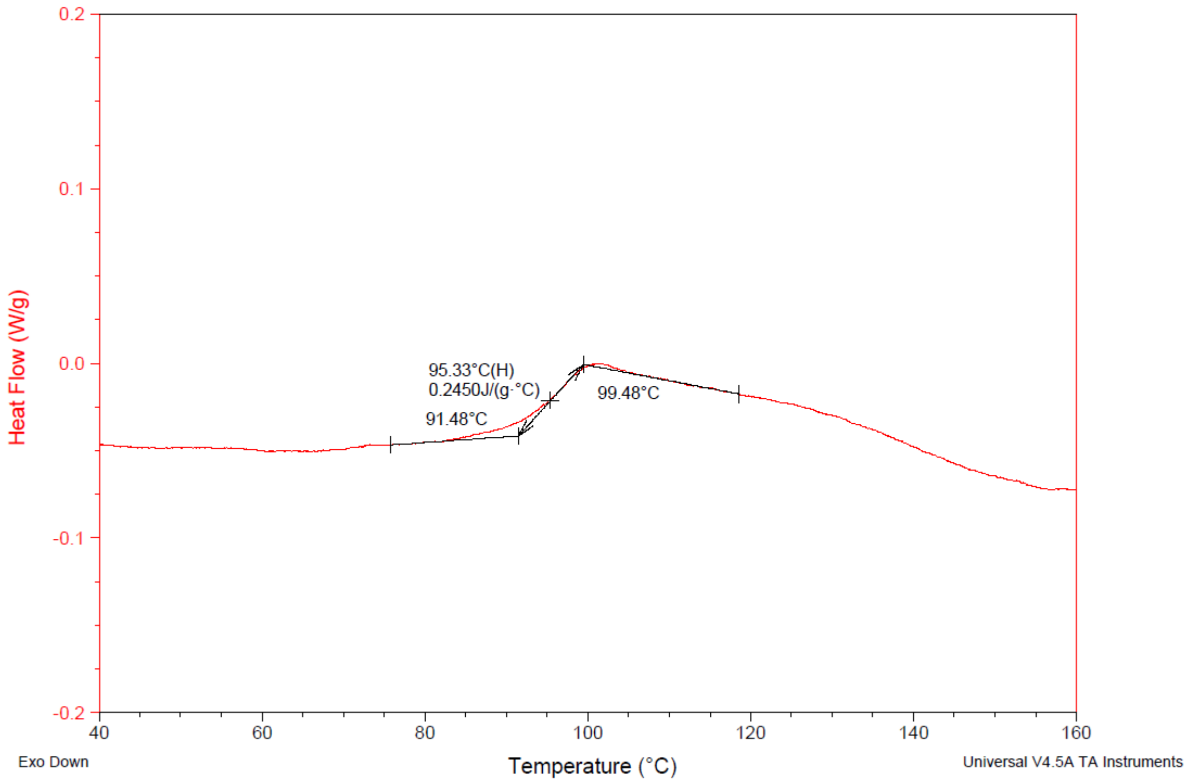

Figure S23. Glass point measurement of pure polymer **E2**; glass point was *ca*. 95 °C.

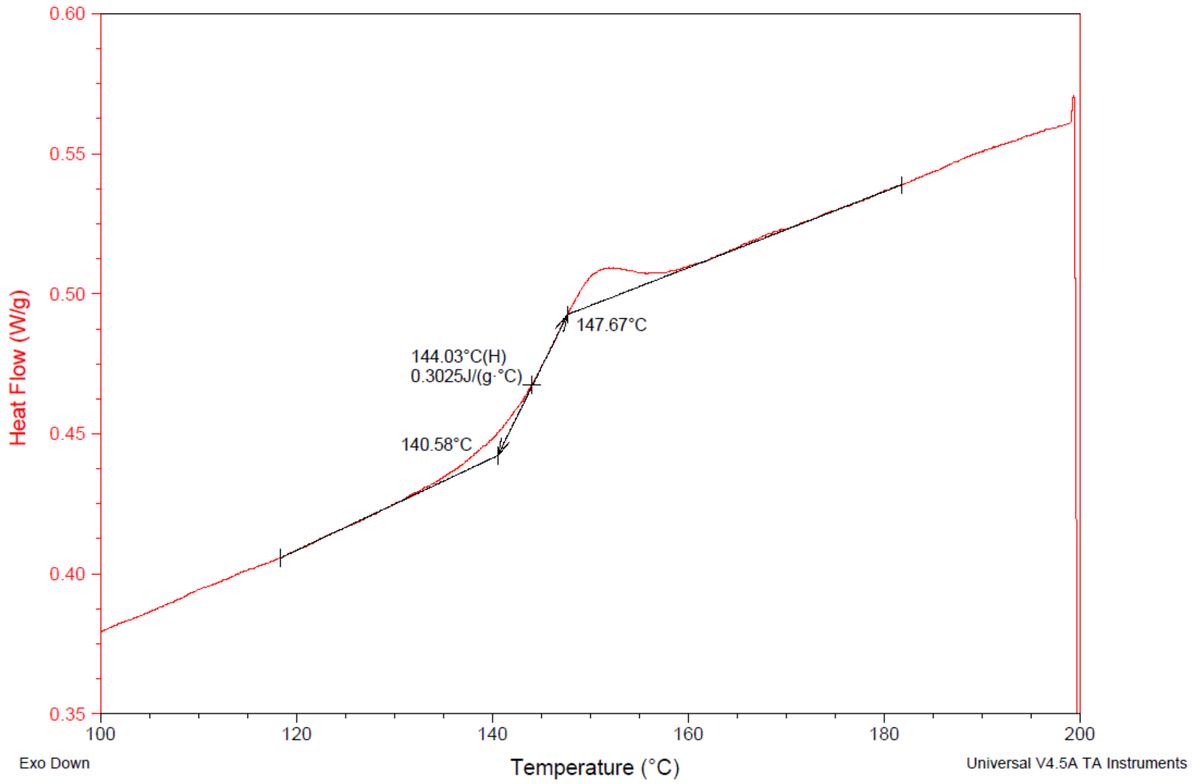

Figure S24. Glass point measurement of pure polymer **P2**; glass point was *ca*. 144 °C.

Table S9. Glass point temperatures (T_g) of pure (bulk) polymers and enthalpy of their glass-point transitions (H_{Tg}).

**Polymer**		T_g (°C)	H_{Tg} (J/(g·°C))
**pDFEA**	**F2**	113	0.336
**pNIPAM**	**I2**	136	0.506
**pDEA**	**E2**	95	0.245
**pAP**	**P2**	144	0.303

S8.3.2. Cloud point temperature (T_{CP}) of aqueous solutions of polymers

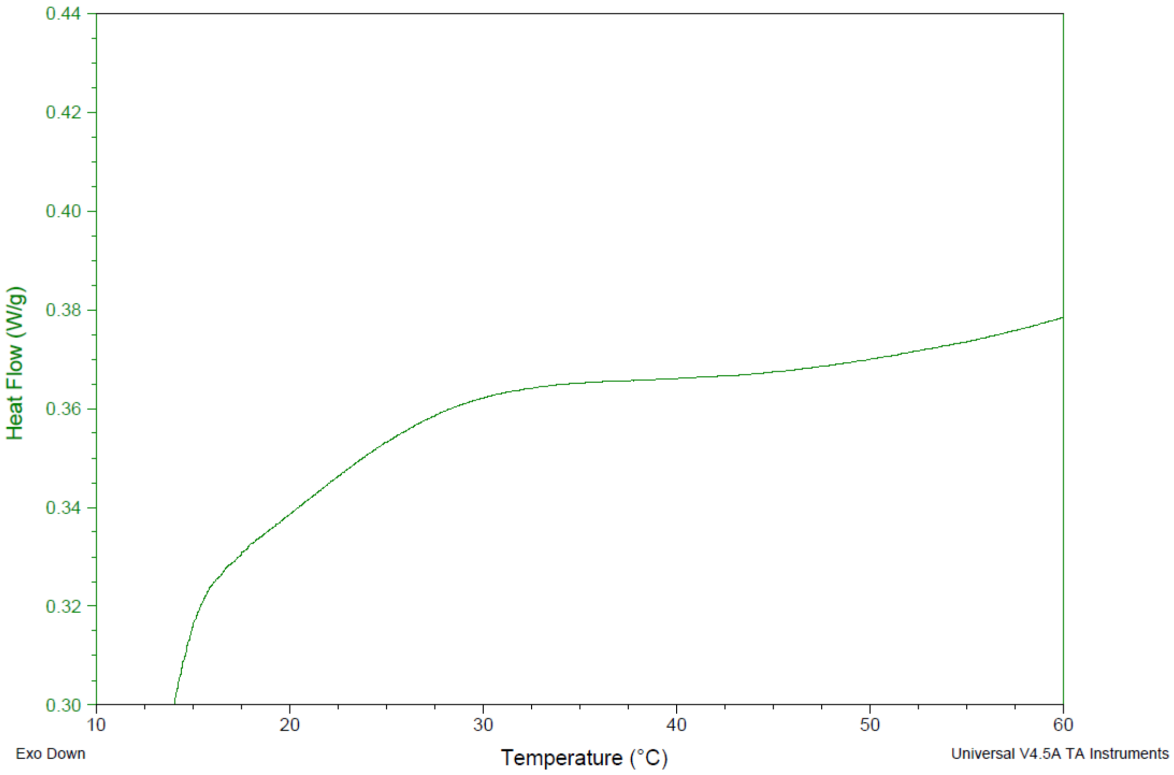

Figure S25. DSC measurement of 20% aqueous solution of polymer **F2**.

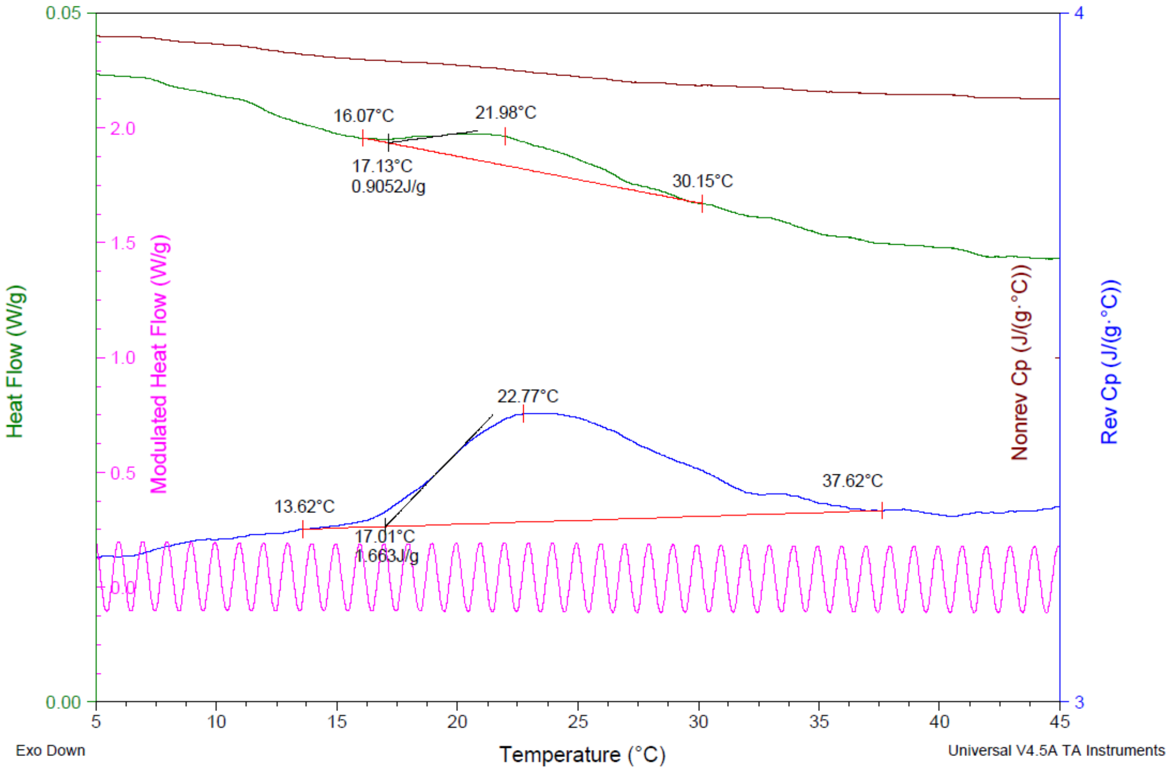

Figure S26. DSC measurement of 20% aqueous solution of polymer **F2**.

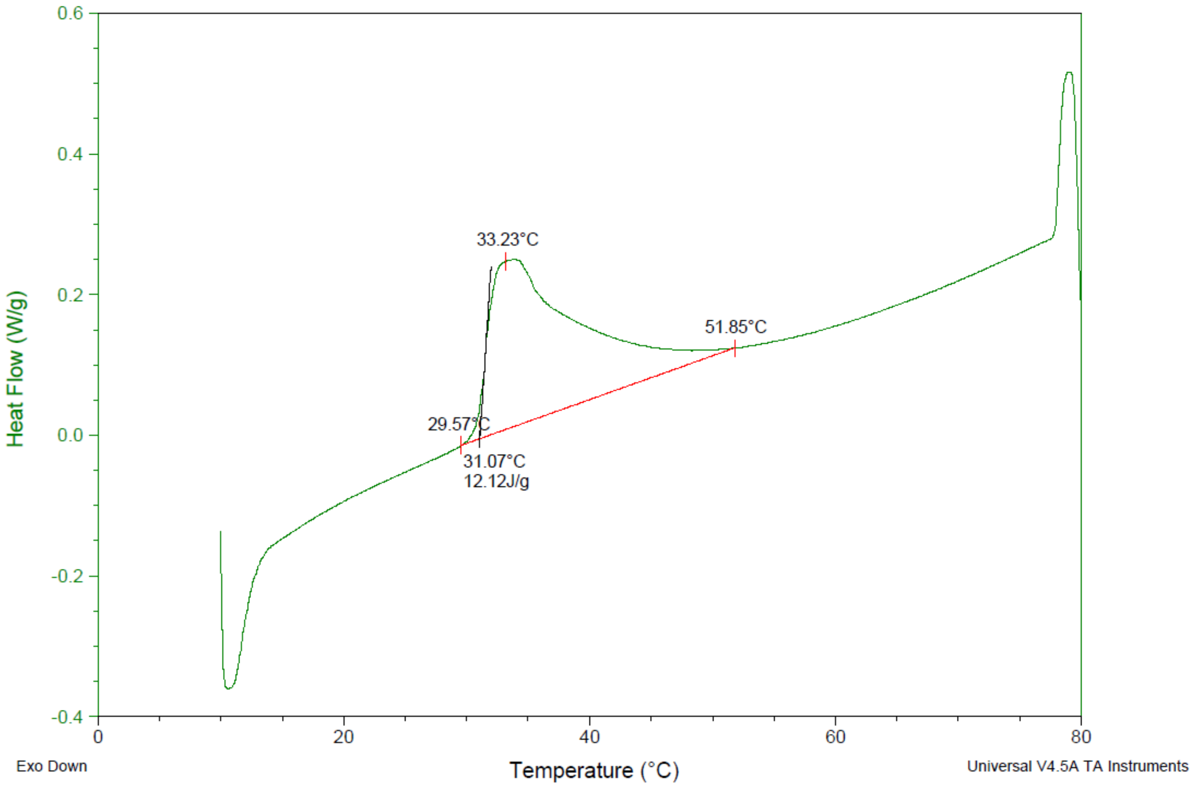

Figure S27. DSC measurement of 20% aqueous solution of polymer **I2**.

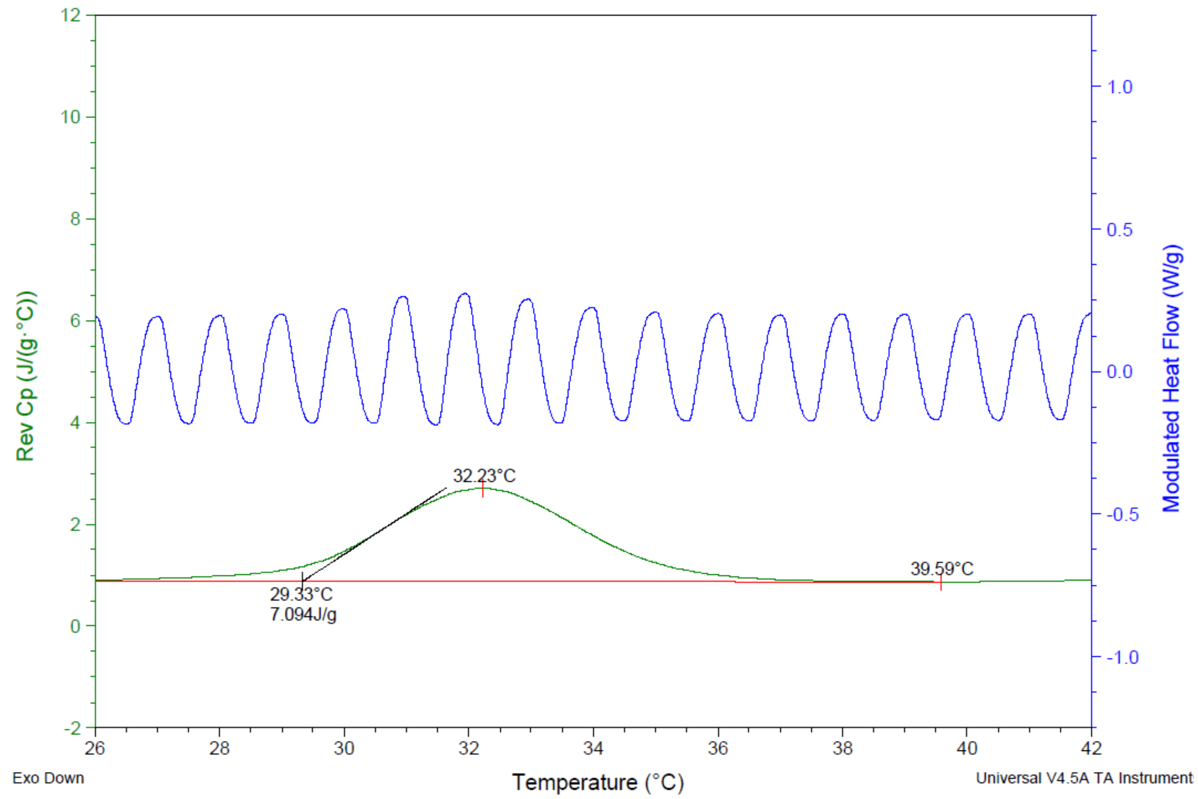

Figure S28. DSC measurement of 20% aqueous solution of polymer **I2**.

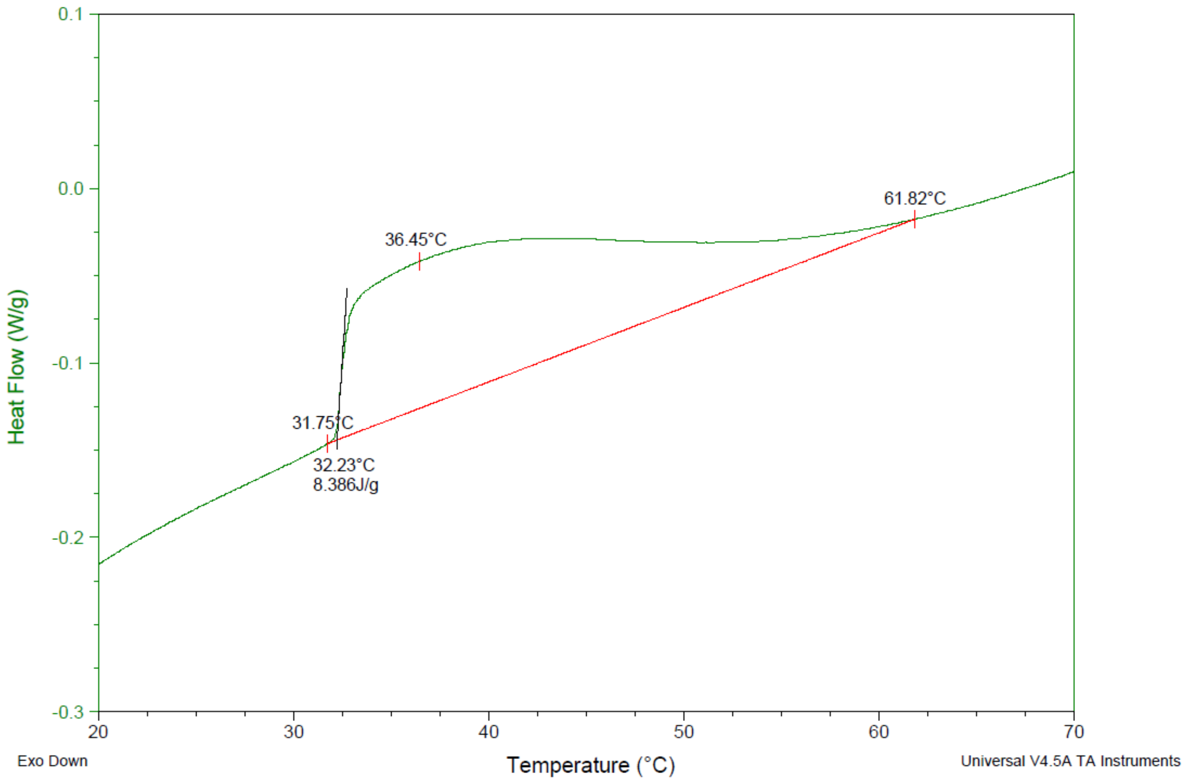

Figure S29. DSC measurement of 20% aqueous solution of polymer **E2**.

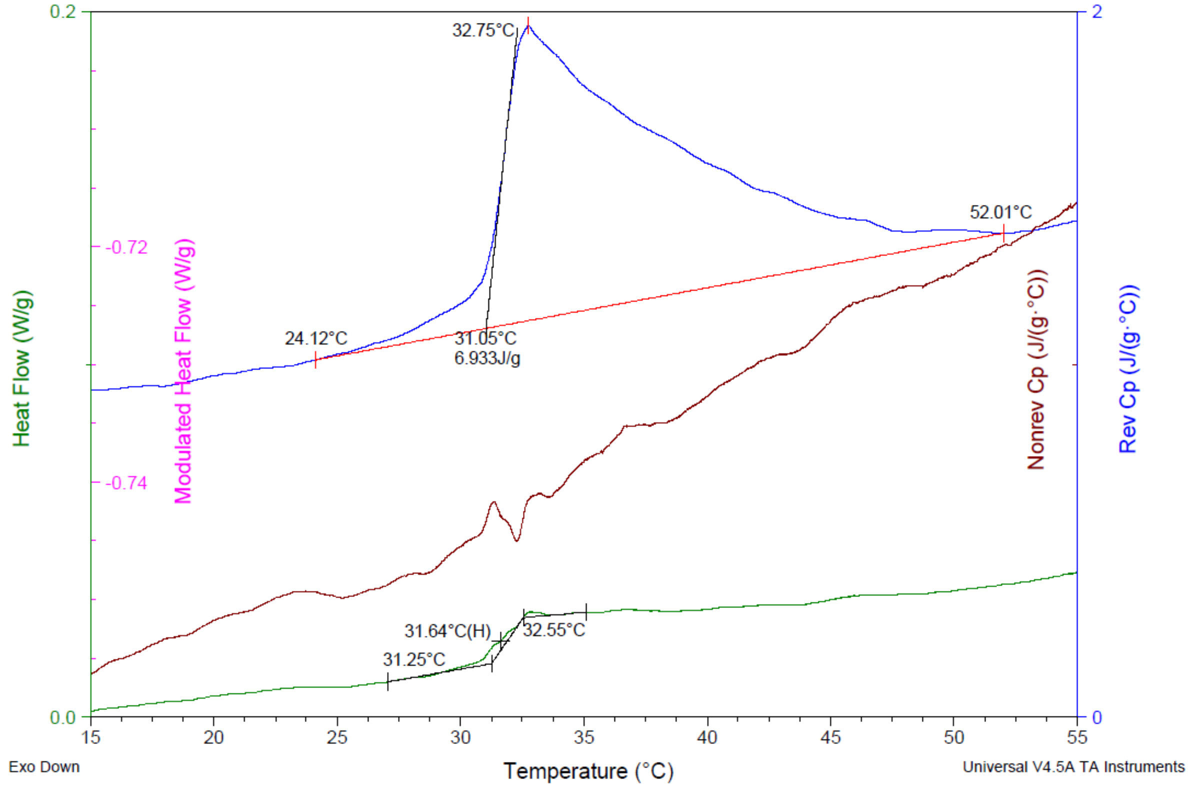

Figure S30. DSC measurement of 20% aqueous solution of polymer **E2**.

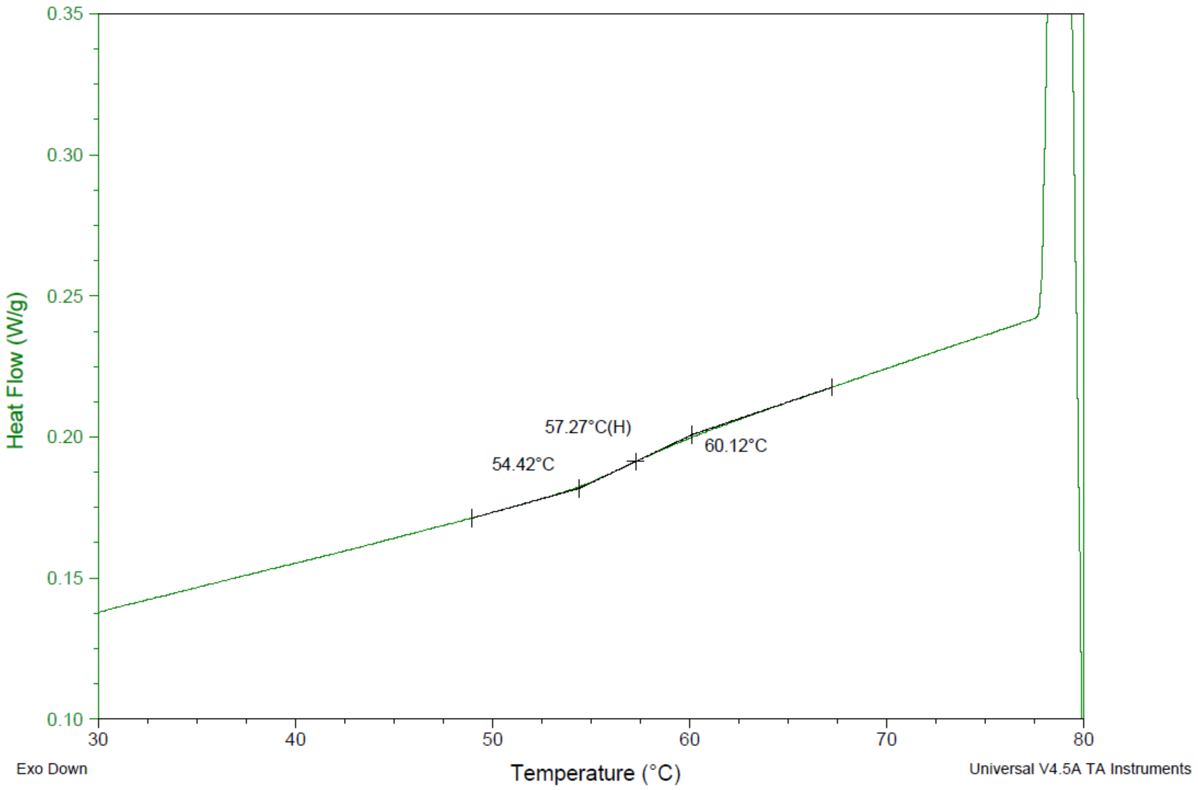

Figure S31. DSC point measurement of 20% aqueous solution of polymer **P2**.

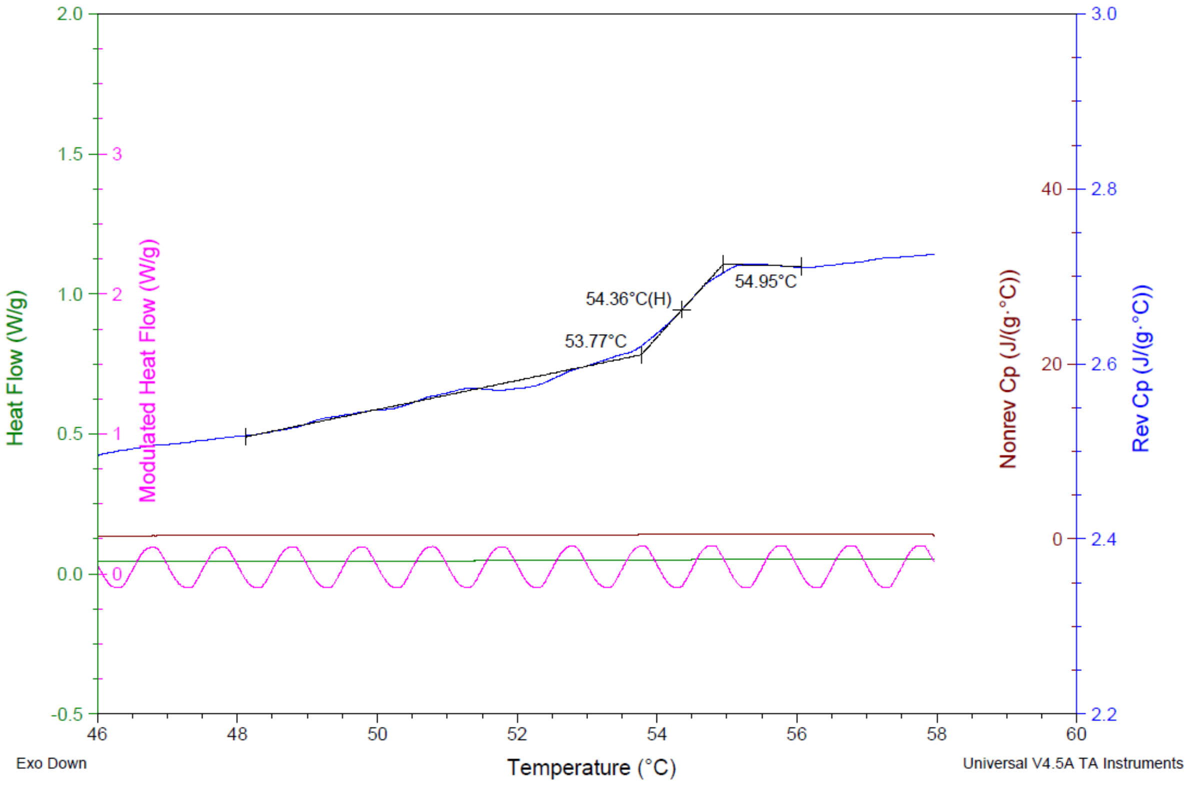

Figure S32. DSC point measurement of 20% aqueous solution of polymer **P2**.

Table S10. Cloud point temperature (T_{CP}) and aggregation heat ($\Delta H_{agg,\ neat}$) of 20 *wt*. % aqueous solutions of polymers

**Polymer**	T_{CP} (°C)	$\Delta H_{agg,\ neat}$* (J/g)
**pDFEA**	17	8
**pNIPAM**	29	36
**pDEA**	31	35
**pAP**	54	2

*ΔH_{agg} - calculated aggregation heat per gram of pure polymer

S8.4. Purity of Cy7 labelled polymers & Cy7 quantification

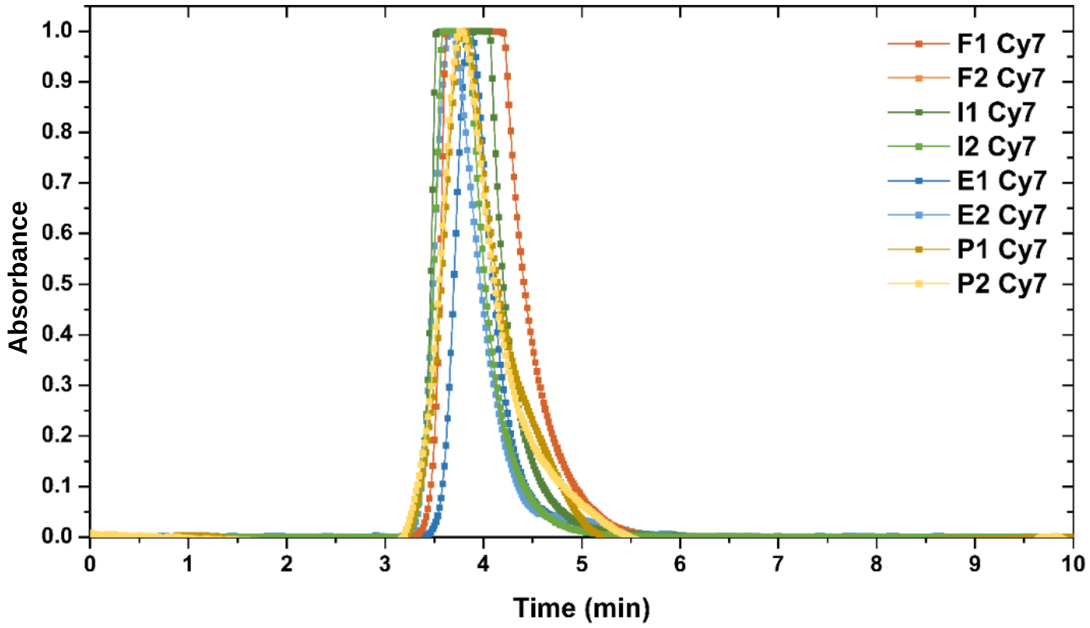

Figure S33. Size exclusion chromatograms (SEC traces) of Cy7-labelled polymers; normalized absorbance at 665 nm as a function of time. The absorbance of polymers in most cases exceeded the detection maximum of the detector. The concentration of low-molecular Cy7 (non-covalently bound) was in most cases below the limit of detection.

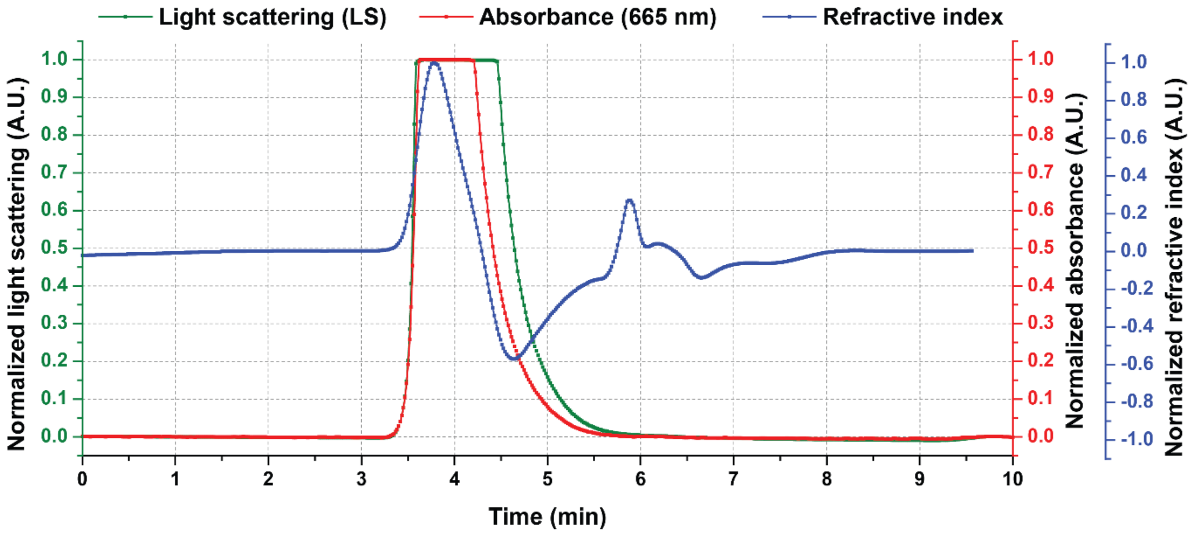

Figure S34. Size exclusion chromatogram of Cy7-labelled **F1**. Absorbance and light-scattering surpassed the detection maxima of detectors.

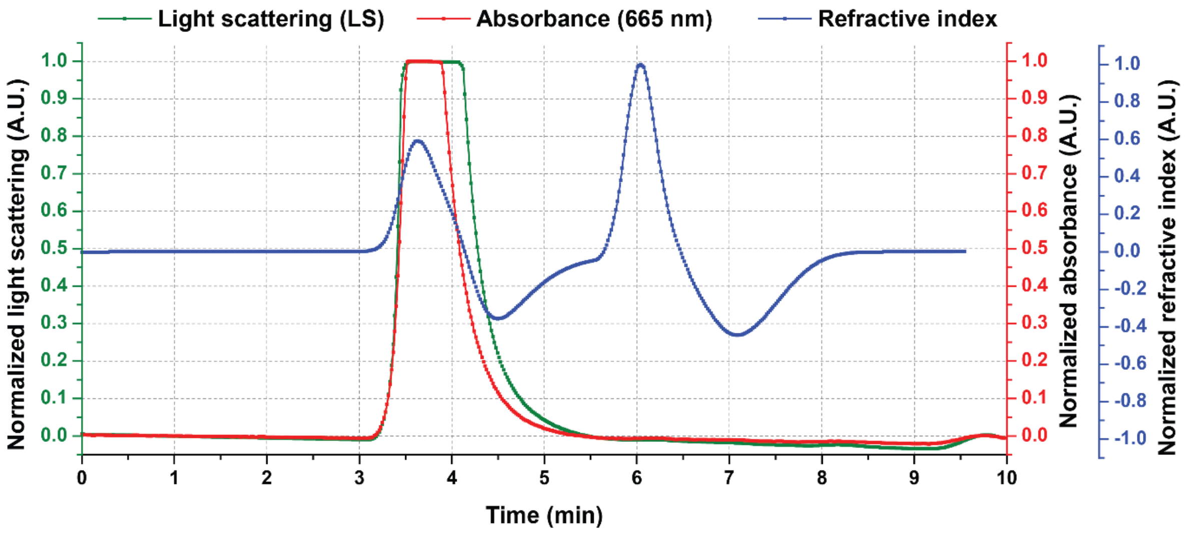

Figure S35. Size exclusion chromatogram of Cy7-labelled **F2**. Absorbance and light-scattering surpassed the detection maxima of detectors.

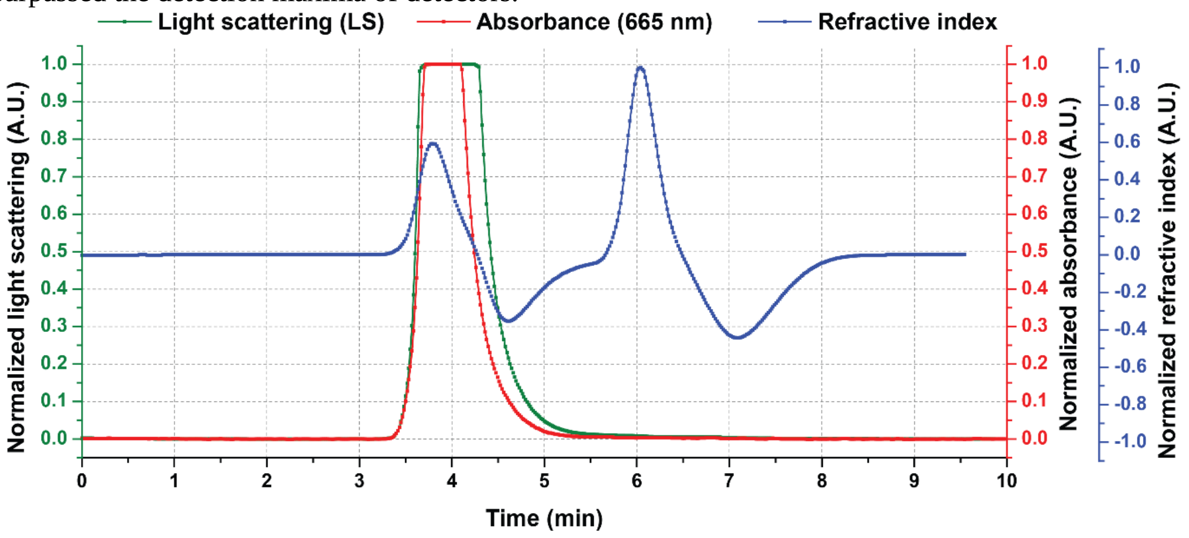

Figure S36. Size exclusion chromatogram of Cy7-labelled **I1**. Absorbance and light-scattering surpassed the detection maxima of detectors.

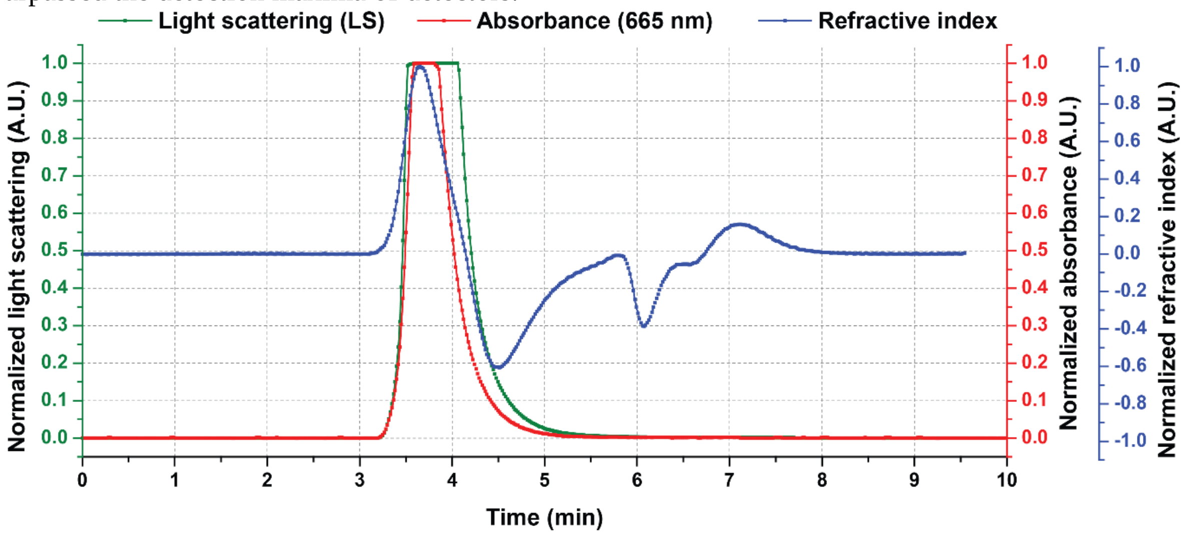

Figure S37. Size exclusion chromatogram of Cy7-labelled **I2**. Absorbance and light-scattering surpassed the detection maxima of detectors.

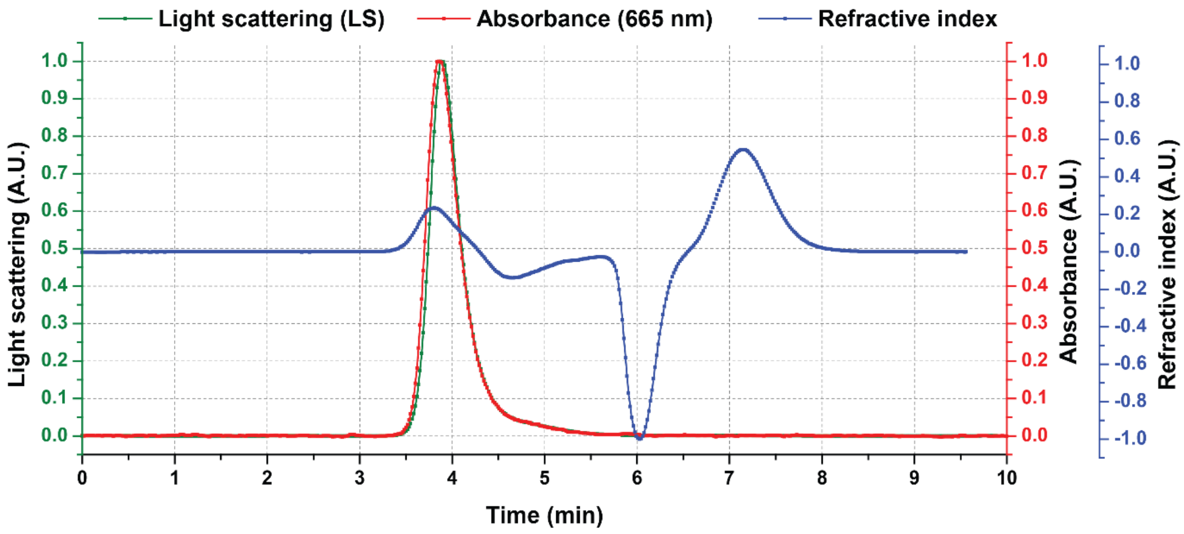

Figure S38. Size exclusion chromatogram of Cy7-labelled **E1**

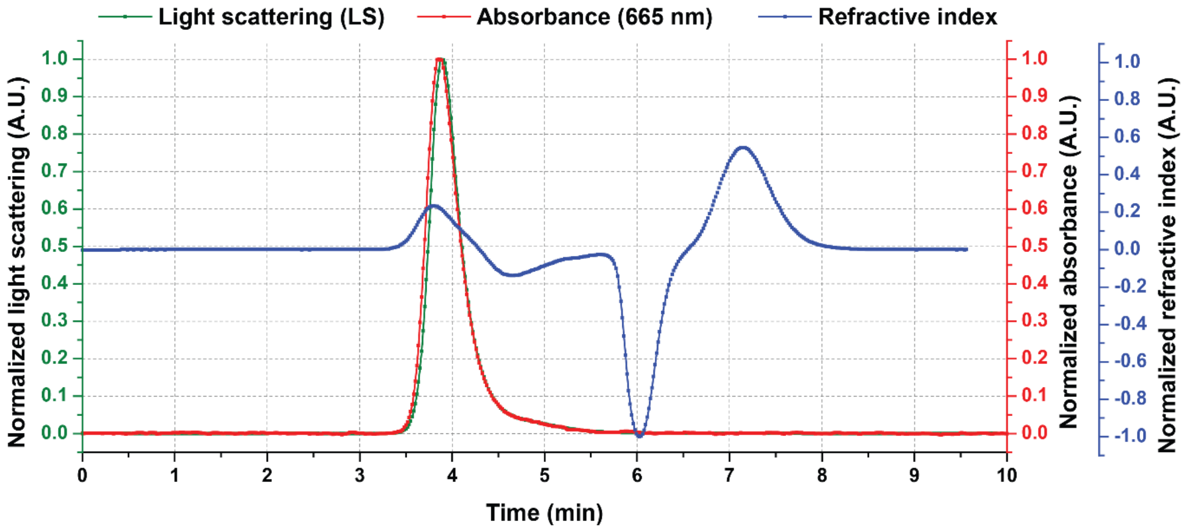

Figure S39. Size exclusion chromatogram of Cy7-labelled **E2**

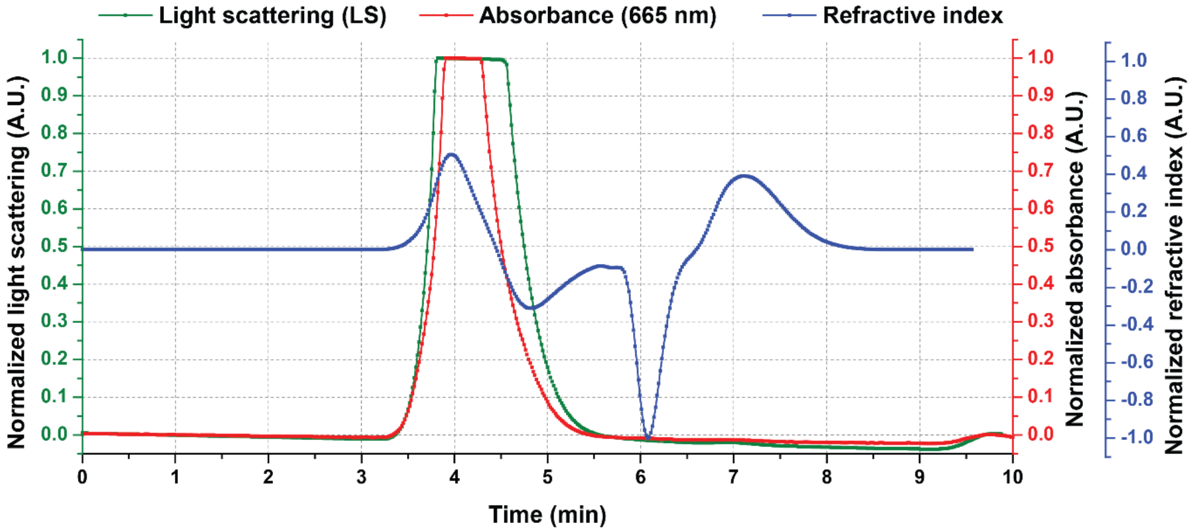

Figure S40. Size exclusion chromatogram of Cy7-labelled **P1**. Absorbance and light-scattering surpassed the detection maxima of detectors.

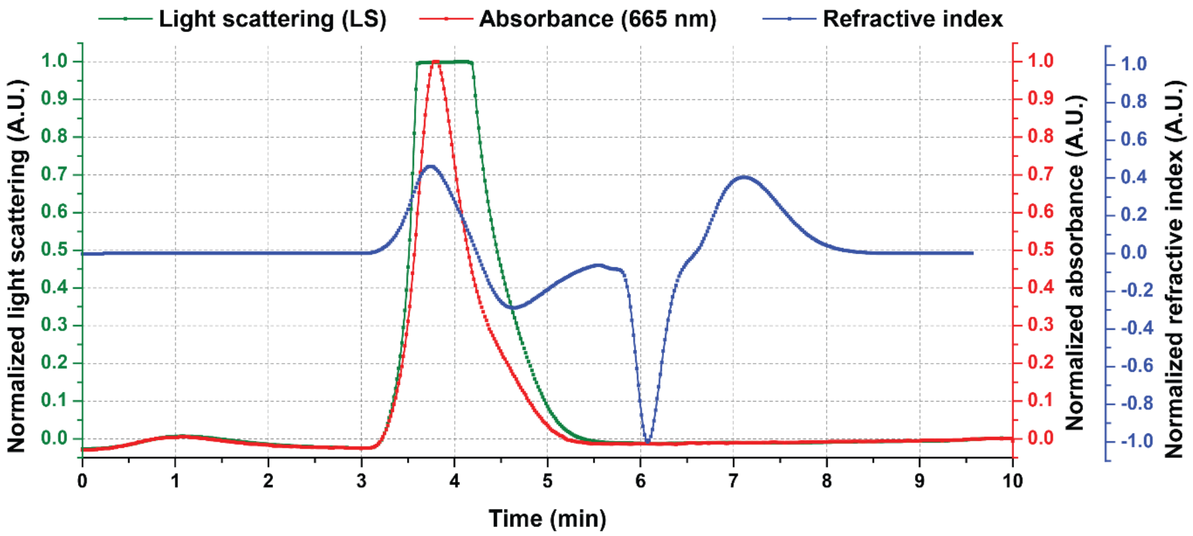

Figure S41. Size exclusion chromatogram of Cy7-labelled **P2**. Light-scattering surpassed the detection maximum of detectors.

Table S11. Molar amount of Cy7 (n_{Cy7}) and ratio of n_{Cy7}/n_{pol} were calculated from spectrophotometry. The polymer purity was determined as a ratio of polymer peak and other peaks integrals in SEC and HPLC; presented as adjusted mean ± mid-range at 95% confidence interval.

**Polymer**		**purity** (%)	n_{Cy7} (μmol/mg)	n_{Cy7}/n_{pol}
**pDFEA**	**F1**	≥ 99.7	24.5 ± 0.9	0.59 ± 0.02
	**F2**	≥ 99.3	13.6 ± 0.7	0.48 ± 0.03
**pNIPAM**	**I1**	≥ 99.6	13.4 ± 0.7	0.26 ± 0.01
	**I2**	≥ 99.5	11.8 ± 0.5	0.36 ± 0.03
**pDEA**	**E1**	≥ 97.3	3.1 ± 0.4	0.06 ± 0.01
	**E2**	≥ 98.4	4.5 ± 0.4	0.14 ± 0.02
**pAP**	**P1**	≥ 99.7	14.3 ± 0.7	0.25 ± 0.01
	**P2**	≥ 98.4	8.6 ± 0.6	0.27 ± 0.02

Note: During the GPC experiments, we injected quite a large amount of polymer into the GPC loop, so that we could detect even trace amounts of non-bound Cy7-amine label. In most samples, the polymer-peak was so intensive that the detector overflowed, which artificially decreased the total integral of the polymer peak (by flattening it).

We calculated the polymer purity as a ratio of integral of polymer-peak and the total integral of all spectra. Even though the polymer peak was artificially decreased in most polymers (due to detector overflow), all polymers were sufficiently pure (all were more than 98% pure, most polymers were more than 99% pure). However, the real purity of all polymers was probably substantially higher that the reported ones.

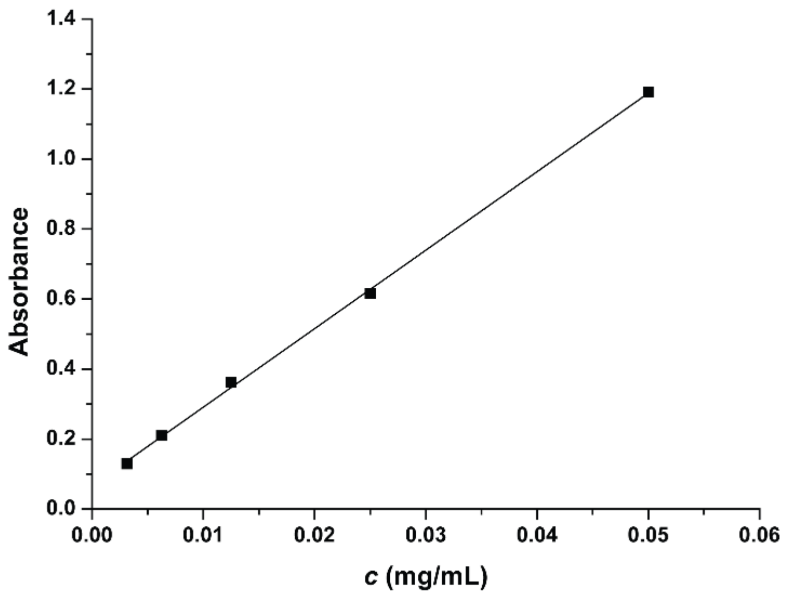

Figure S42. Calibration curve; the absorbance of Cy7-amine as a function concentration (λ = 775 nm) in MeOH, given by the formula $A = 0.0668 \pm 0.0084 + c \cdot (22.429 \pm 0.323)$; $R^2 = 0.9997$.

S8.5. Purity of Dy505 labelled polymers & Dy505 quantification

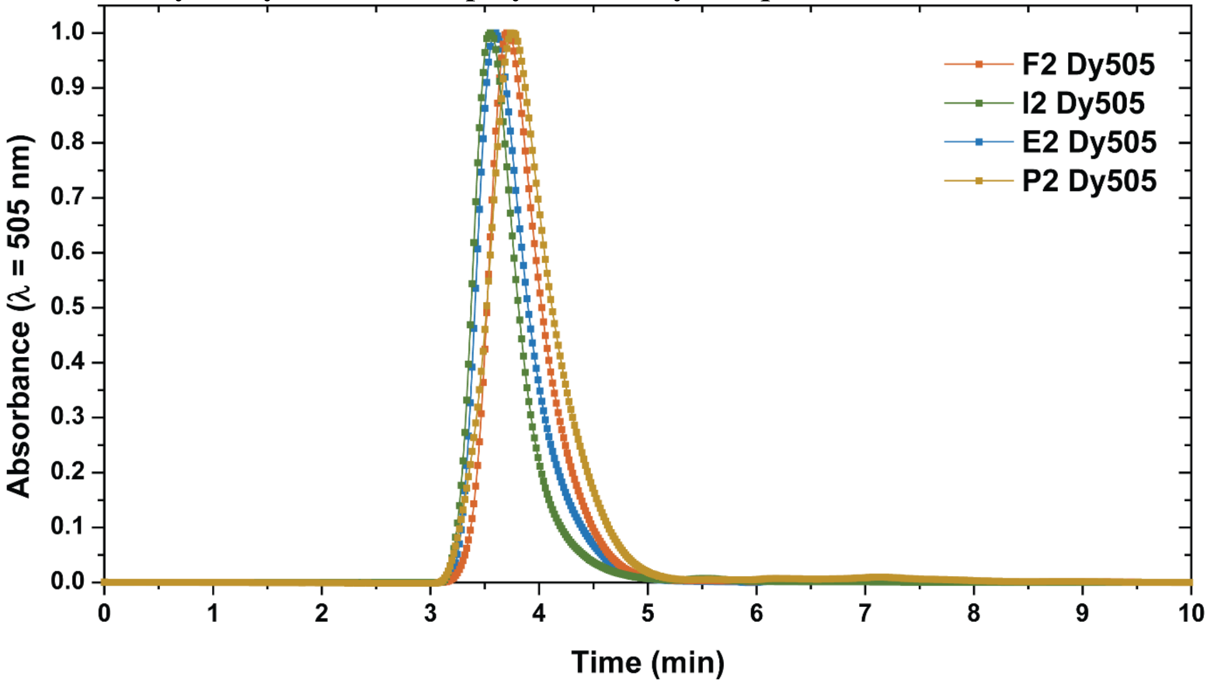

Figure S43. Size exclusion chromatograms (SEC traces) of Dy505-labelled polymers; normalized absorbance at 505 nm as a function of time. The absorbance of polymers did not exceed the detection maximum of the detector. The concentration of low-molecular Dy505 (non-covalently bound) was in most cases below the limit of detection; its concentration was ≥ 0.5%).

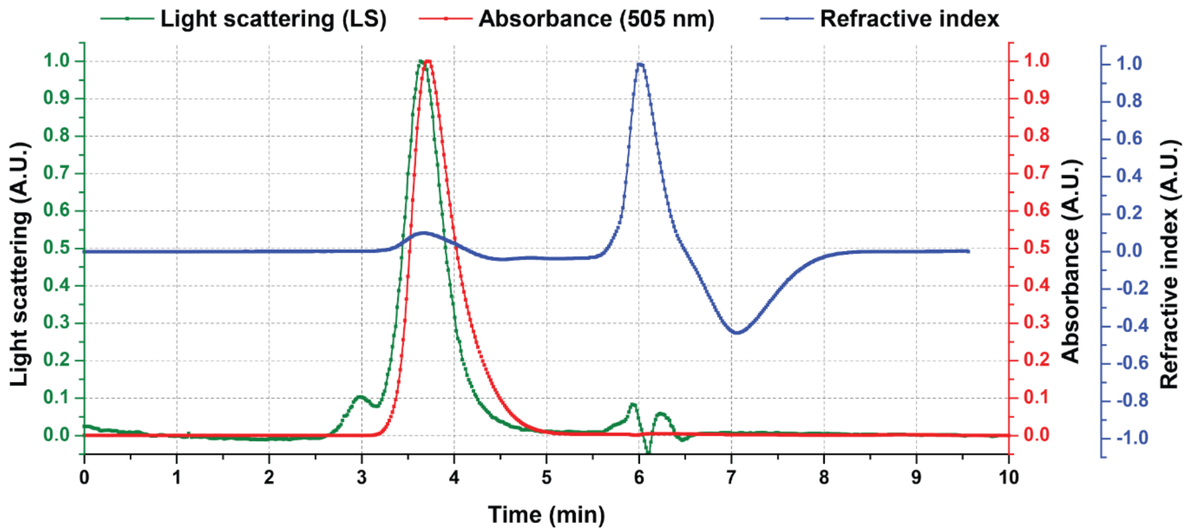

Figure S44. Size exclusion chromatogram of Dy505-labelled **F2**. Light-scattering surpassed the detection maximum of detectors.

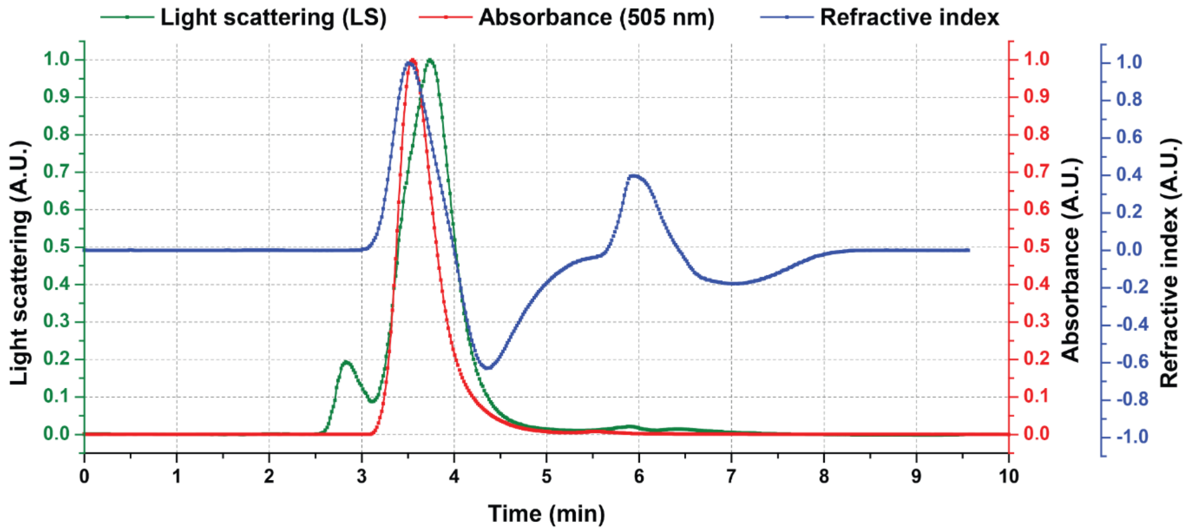

Figure S45. Size exclusion chromatogram of Dy505-labelled **I2**. Light-scattering surpassed the detection maximum of detectors.

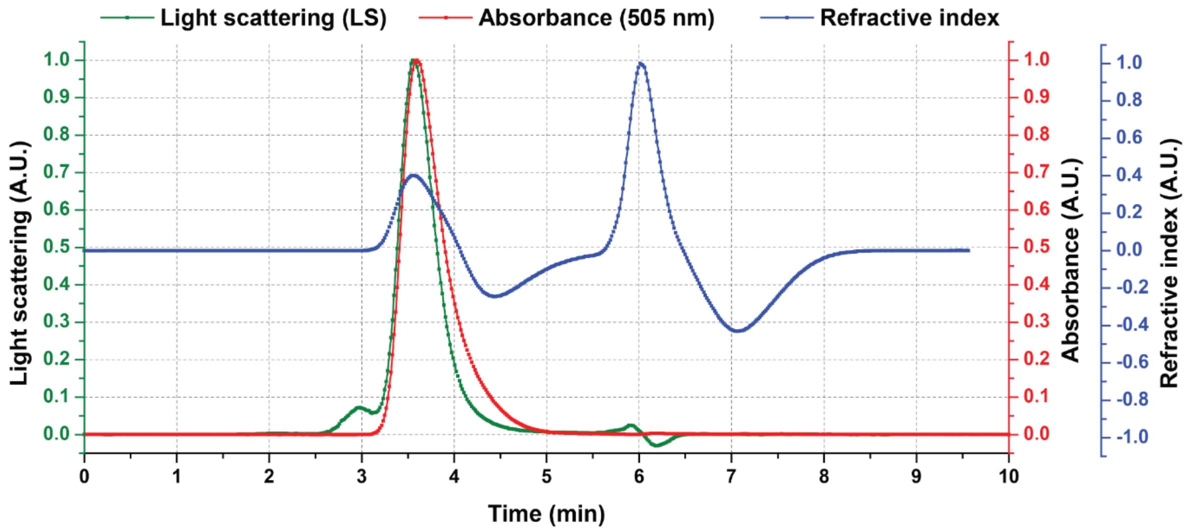

Figure S46. Size exclusion chromatogram of Dy505-labelled **E2**. Light-scattering surpassed the detection maximum of detectors.

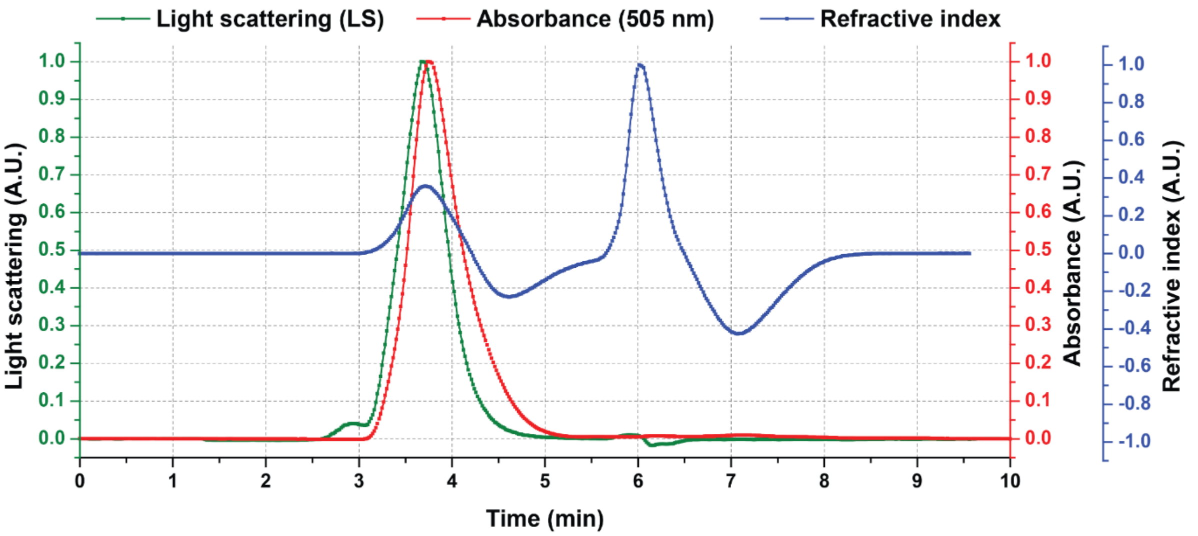

Figure S47. Size exclusion chromatogram of Dy505-labelled **P2**. Light-scattering surpassed the detection maximum of detectors.

Table S12. Molar amount of Dy505 (n_{Dy505}) and ratio of n_{Dy505}/n_{pol} were calculated from spectrophotometry. The polymer purity (PP) was determined as a ratio of polymer peak and other peaks integrals in SEC and HPLC.

**Polymer**		**purity** (%)	n_{DY505} (μmol/mg)	n_{DY505}/n_{pol}
**pDFEA**	**F2**	≥ 99.9	5.1 ± 0.5	0.30 ± 0.03
**pNIPAM**	**I2**	≥ 99.4	2.9 ± 0.3	0.15 ± 0.01
**pDEA**	**E2**	≥ 99.9	1.6 ± 0.4	0.09 ± 0.02
**pAP**	**P2**	≥ 99.9	4.9 ± 0.5	0.26 ± 0.03

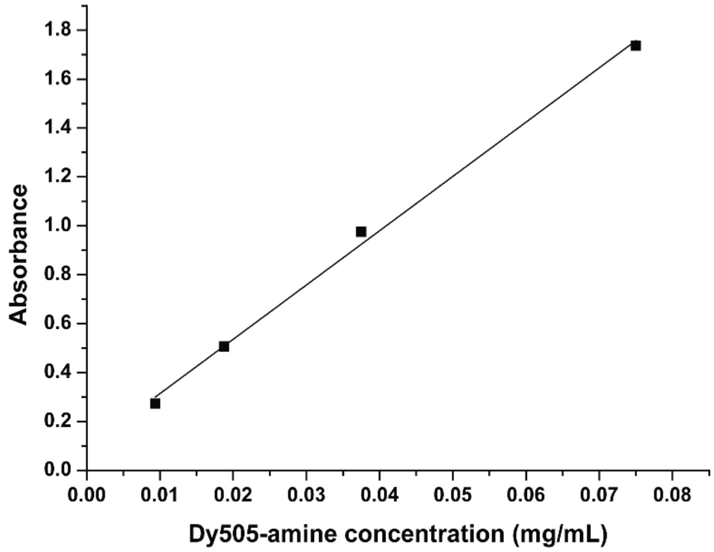

Figure S48. Calibration curve; the absorbance of Dy505-amine as a function of concentration (λ = 505 nm) in MeOH, given by the formula $A = 0.0917 \pm 0.0371 + c{\cdot}(22.207 \pm 0.859)$; $R^2 = 0.9985$.

S8.5.1. Fluorescence characteristics of Cy7-labelled polymers

Emission spectra of fluorescent polymers were measured for all four polymers, **F2**, **I2**, **E2**, **P2**, below and above the T_{CP} (at 0 ± 5 °C and 60 ± 5 °C). Except the fluorinated polymer **F2**, which did not become turbid, all polymers were a clear blue-green solutions at 0 °C and became highly turbid at 60 °C. This process was almost instantaneous after reaching T_{CP} and could be repeated multiple times without any observed change in absorption or emission spectrum. The change in turbidity is demonstrated in **Figure S49** for polymer **I2**. The baseline is increased due to uneven light scattering on the precipitated polymer. Other polymers behave similarly.

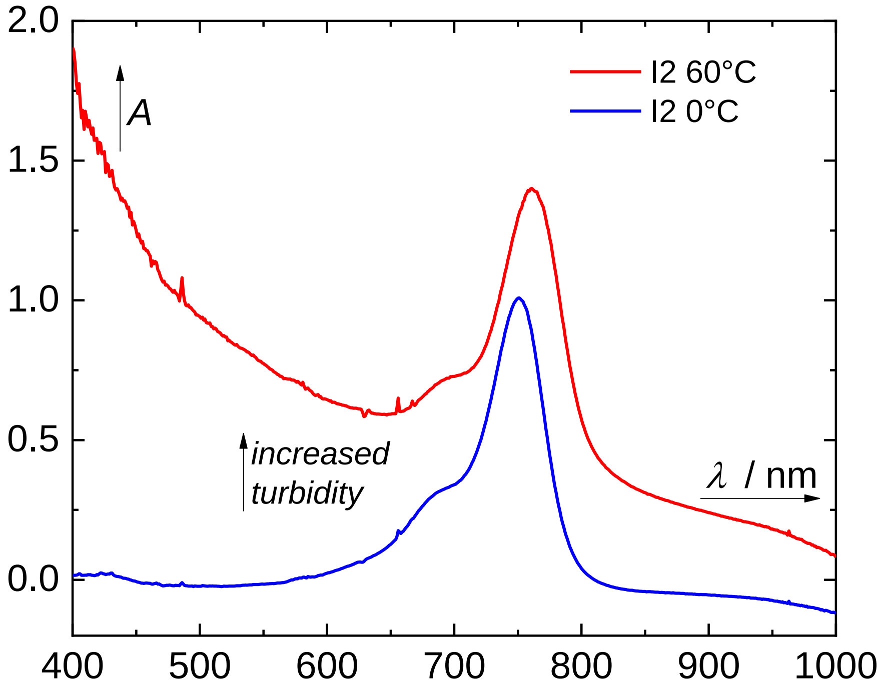

Figure S49. Absorption spectra of an aqueous solution of **I2** (c_{pol} = 1.25 mg/mL) at 0 °C (blue line) and 60 °C (red line).

The attachment of Cy7 at polymers led to a bathochromic shift of their emission from 766 nm for free dye to 780 nm for all polymers. No variation of the emission maxima between different polymers was found (**Figure S50**).

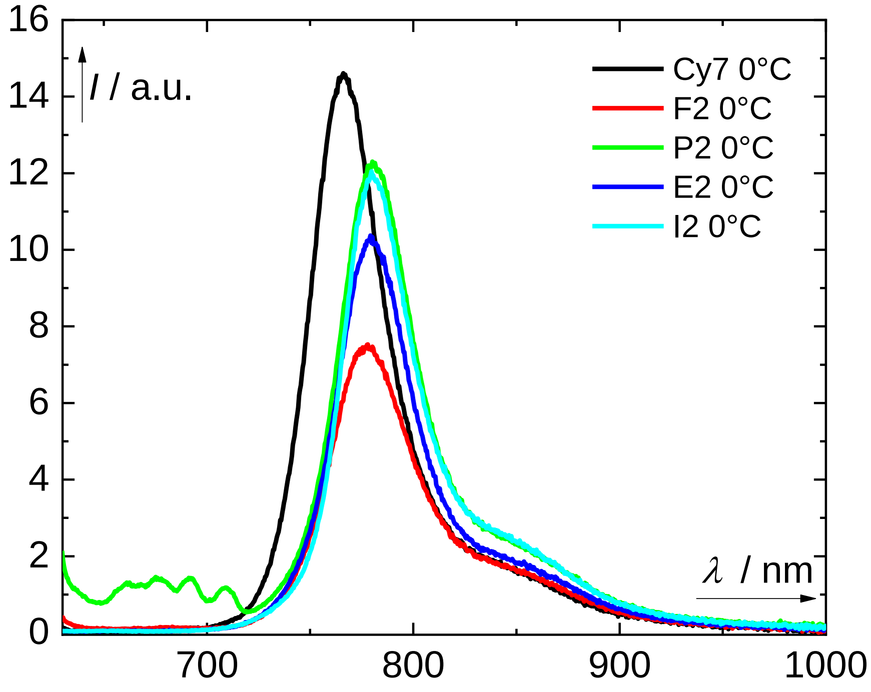

Figure S50. Emission spectra of an aqueous solution of Cy7-labelled polymers (coloured lines, c_{pol} = 1.25 mg/mL) and Cy7 dye (black line) excited at 620 nm at 0 °C.

Heating of polymer solutions to 60°C led (with the exception for **F2**, whose precipitation was not observed under experimental conditions described above) to the increase of turbidity of the solutions. This decreases the sample transparency and increases the scattering of the excitation light. In all cases the emission signal was lower at 60°C than at 0°C due to the lower amount of excitation light efficiently addressing the observed emissive molecules (**Figure S51** to **Figure S53**). Moreover, the precipitation induced aggregation of Cy7 dye as observed by emission signal broadening and further bathochromic shift by ≈ 20 nm. Furthermore, we observed an artifact caused by Raman scattering in turbid samples at ≈ 650 nm. Note that the intensity of the Raman signal relative to the emission maximum is different for different polymers, which can be attributed to different levels of precipitation (caused by different T_{CP}'s) of polymers.

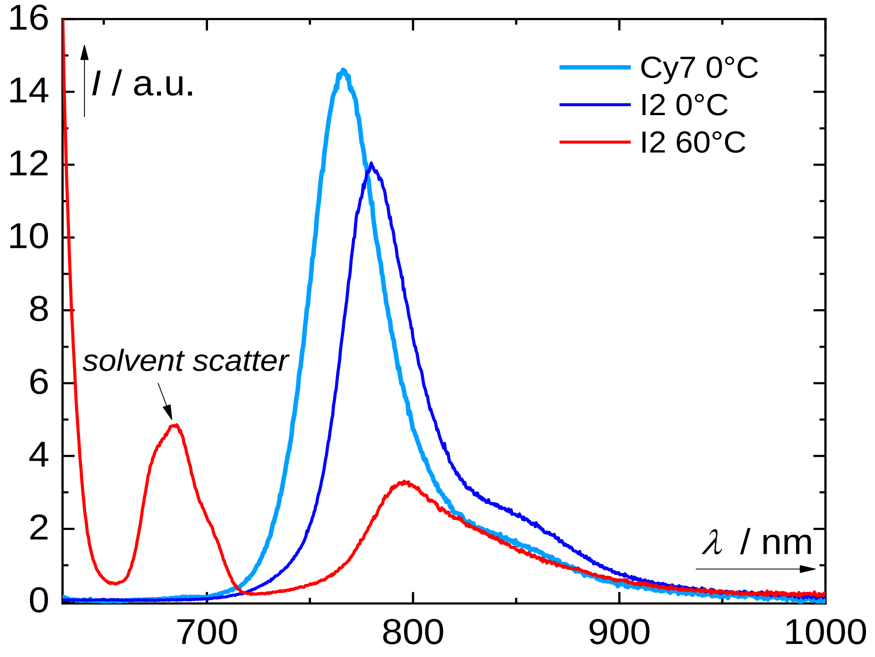

Figure S51. Emission spectra (λ_{exc} = 620 nm) of an aqueous solution of **I2** (c_{pol} = 1.25 mg/mL) at 0°C (navy blue line) and at 60°C (orange line), respectively, and of Cy7 dye (light blue line) at 0°C. The Raman scatter signal is labelled by an arrow.

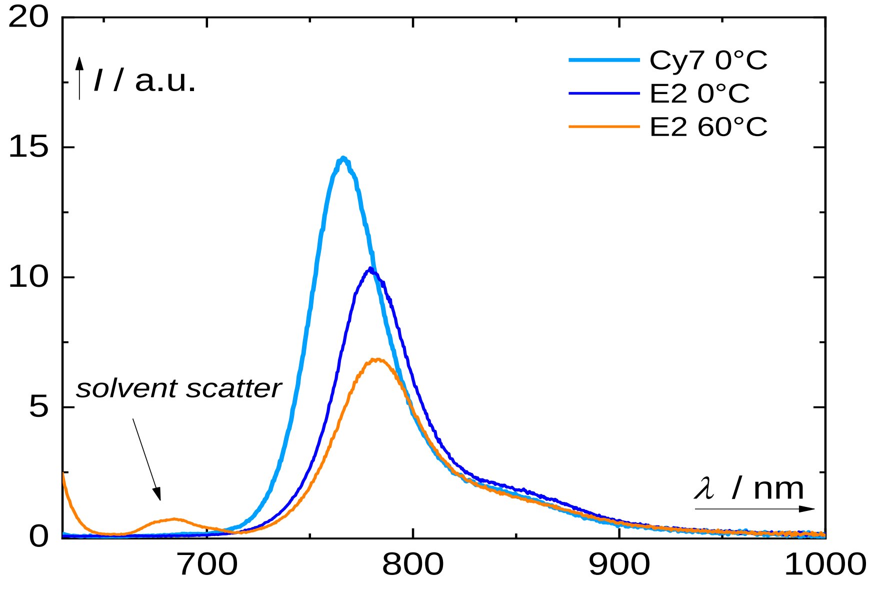

Figure S52. Emission spectra (λ_{exc} = 620 nm) of an aqueous solution of **E2** (c_{pol} = 1.25 mg/mL) at 0 °C (navy blue line) and at 60 °C (orange line), respectively, and of Cy7 dye (light blue line) at 0 °C. The Raman scatter signal is labelled by an arrow.

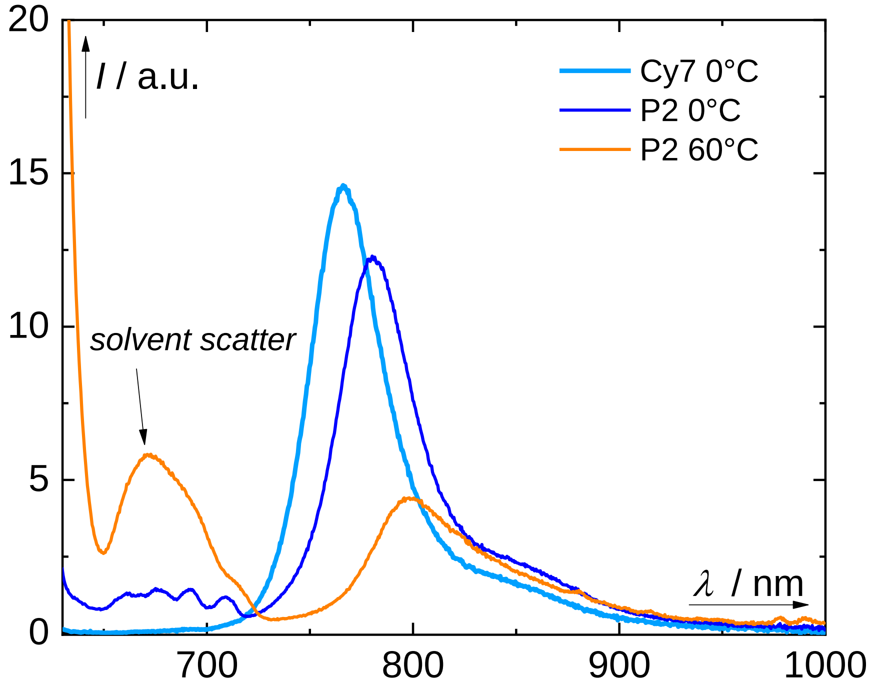

Figure S53. Emission spectra (λ_{exc} = 620 nm) of an aqueous solution of **P2** (c_{pol} = 1.25 mg/mL) at 0°C (navy blue line) and at 60°C (orange line), respectively, and of Cy7 dye (light blue line) at 0°C. The Raman scatter signal is labelled by an arrow.

To prove that the additional peak at ≈ 650 nm is caused by Raman scattering, we measured excitation emission matrices for clear solutions and their turbid counterparts. While at 0 °C the clear solution has a single cross peak corresponding to the emission of monomeric Cy7 (**Figure S54**), turbid solutions at 60 °C have very intense Rayleigh scattering (diagonal grey trace) and significant Raman scattering (parallel less intense diagonal trace in **Figure S55**). Moreover, the mission cross peak is red shifted by ≈ 20 nm, which can be attributed to Cy7 aggregation.

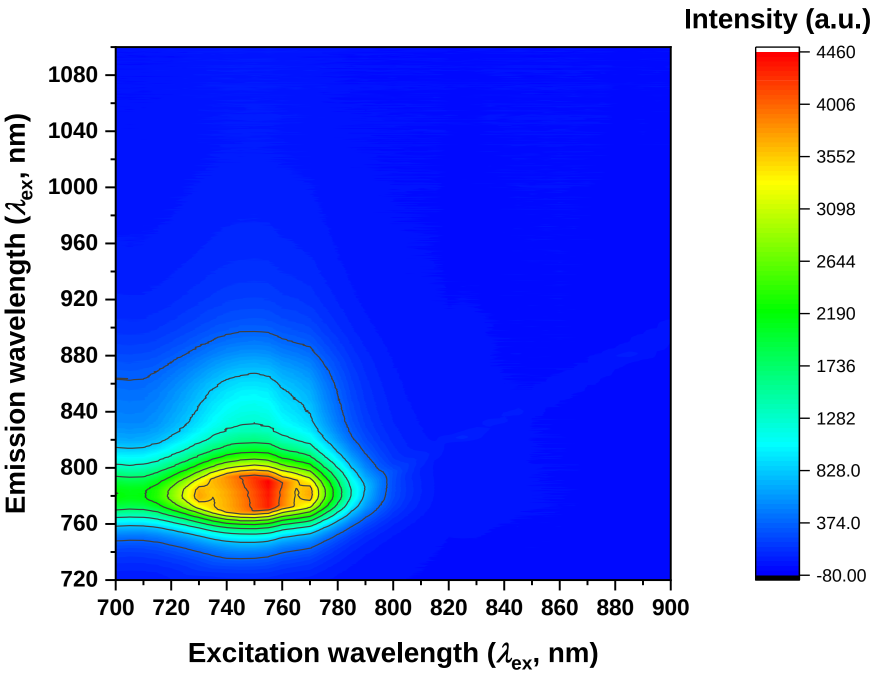

Figure S54. Excitation-emission matrix of an aqueous solution of I2 (c_{pol} = 1.25 mg/mL) at 0 °C.

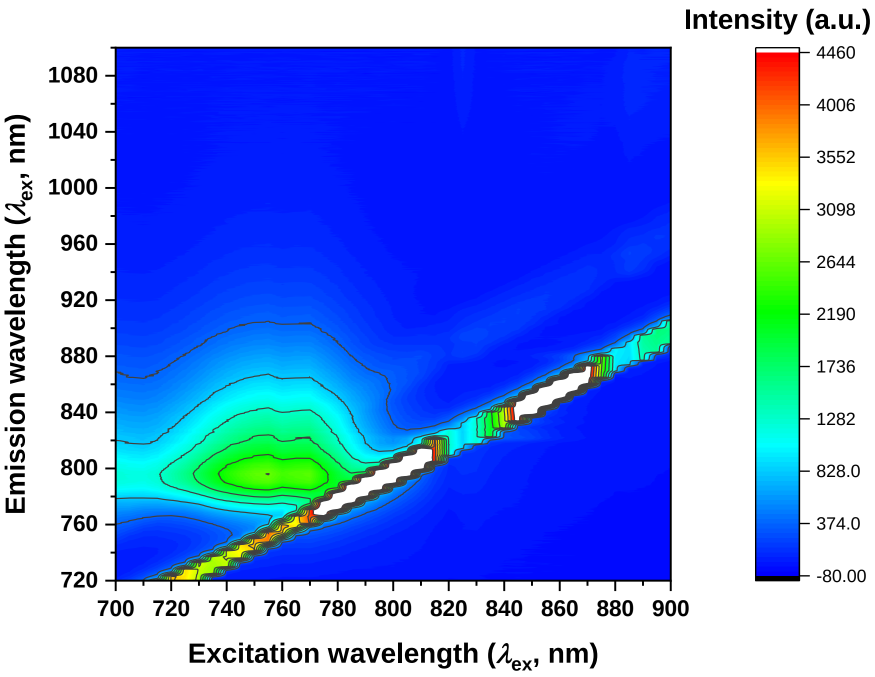

Figure S55. Excitation-emission matrix of an aqueous solution of I2 (c_{pol} = 1.25 mg/mL) at 60 °C.

S9. In vitro cellular assays

S9.1. *In vitro* cytotoxicity

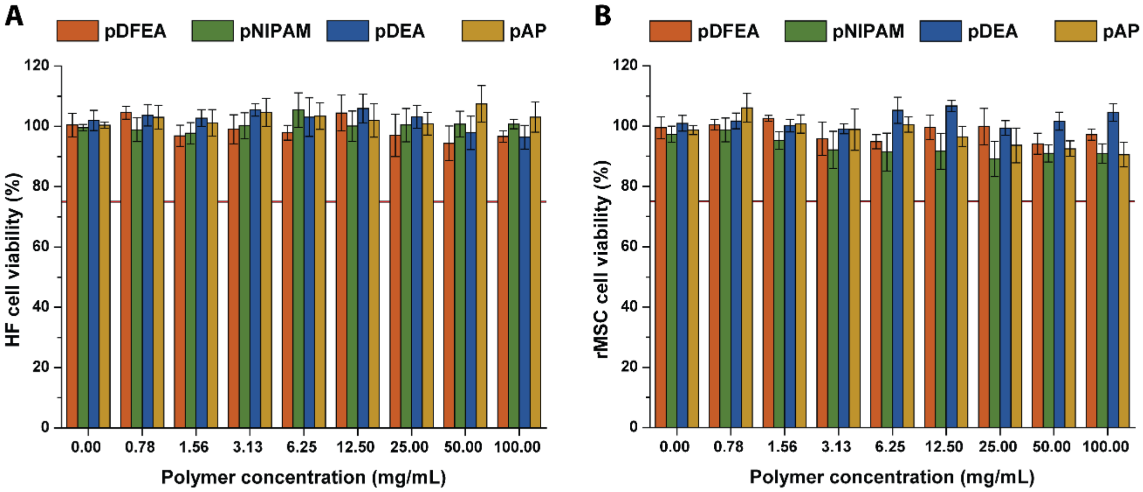

Figure S56. Viability of human fibroblast (HF; **A**) and rat mesenchymal stem cell (rMSC, **B**) as a function of polymers (**F2**, **I2**, **E2**, and **P2**) concentration. All cells were incubated for 72 hours with study polymers; the cell viability was determined using resazurin assay.

Table S13. Viability of HF cells as a function of polymer concentration

Concentration (mg/L)	pDFEA		pNIPAM		pDEA		pAP	
	mean	SD	mean	SD	mean	SD	mean	SD
0.00	100.446	3.96236	99.6124	1.02335	101.976	3.35739	100.388	1.02335
0.78	104.546	2.16713	98.8913	3.93237	103.63	3.52454	103.034	3.90041
1.56	96.8882	3.50198	97.7312	3.5587	102.75	2.81624	101.197	4.44088
3.13	99.0572	4.82572	100.229	4.30347	105.49	2.08225	104.629	4.62259
6.25	97.9052	2.44935	105.436	5.70748	103.076	6.42972	103.421	4.37469
12.5	104.434	5.95435	100.114	4.9981	105.963	4.75932	101.969	5.52273
25.0	97.0525	7.03572	100.464	5.51507	103.129	3.74153	100.891	3.7199
50.0	94.3835	5.71729	100.744	4.26966	97.8854	5.54755	107.445	6.05601
100.0	96.6315	1.91016	100.766	1.52336	96.4386	3.92773	103.091	4.98764

Table S14. Viability of rMSC cells as a function of polymer concentration

Concentration (mg/L)	pDFEA		pNIPAM		pDEA		pAP	
	mean	SD	mean	SD	mean	SD	mean	SD
0.00	99.5038	3.57921	97.3594	2.64435	100.955	2.64536	98.6734	1.51999
0.78	100.481	1.70028	98.7237	3.92493	101.732	2.60926	106.084	4.77298
1.56	102.627	0.98052	95.2236	2.97813	100.139	2.04965	100.728	3.0438
3.13	95.8757	5.5147	92.1281	6.16903	99.1009	1.60872	98.9022	6.91182
6.25	94.8396	2.35177	91.4383	6.27113	105.246	4.33826	100.529	2.53273
12.5	99.6425	4.0598	91.6709	5.91869	106.687	1.82313	96.5014	3.33582
25.0	99.8719	6.0296	89.1057	5.86252	99.3902	2.43849	93.637	5.75627
50.0	94.1514	3.46544	90.9449	2.84823	101.606	3.01111	92.5258	2.55563
100.0	97.1525	1.80033	90.9005	3.25455	104.533	2.89534	90.5595	4.08814

Table S15. ANOVA of F2 toxicity in HF (comparison of control group and polymer-treated cells)

Dunnett's Multiple Comparison Test	Mean Diff.	q	Significant? P < 0.05?	Summary	95% CI of diff
NT vs 0.78μg/ml	− 4.100	0.574	No	ns	− 25.06 to 16.86
NT vs 1.56 μg/ml	3.560	0.499	No	ns	− 17.40 to 24.52
NT vs 3.13 μg/ml	1.390	0.195	No	ns	− 19.57 to 22.35
NT vs 6.25 μg/ml	2.543	0.356	No	ns	− 18.42 to 23.50
NT vs 12.50 μg/ml	− 3.987	0.558	No	ns	− 24.95 to 16.97
NT vs 25 μg/ml	3.393	0.475	No	ns	− 17.57 to 24.35
NT vs 50 μg/ml	6.063	0.849	No	ns	− 14.90 to 27.02
NT vs 100 μg/ml	3.813	0.534	No	ns	− 17.15 to 24.77

Table S16. ANOVA of I2 toxicity in HF (comparison of control group and polymer-treated cells)

Dunnett's Multiple Comparison Test	Mean Diff.	q	Significant? P < 0.05?	Summary	95% CI of diff
NT vs 0.78 ug/ml	0.720	0.207	No	ns	− 9.218 to 10.66
NT vs 1.56 ug/ml	1.883	0.542	No	ns	− 8.055 to 11.82
NT vs 3.13 ug/ml	− 0.615	0.177	No	ns	− 10.55 to 9.323
NT vs 6.25 ug/ml	− 5.822	1.676	No	ns	− 15.76 to 4.115
NT vs 12.50 ug/ml	− 0.501	0.134	No	ns	− 11.24 to 10.23
NT vs 25.00 ug/ml	− 0.850	0.245	No	ns	− 10.79 to 9.088
NT vs 50.00 μg/ml	− 1.131	0.301	No	ns	− 11.87 to 9.603
NT vs 100.00 μg/ml	− 1.157	0.308	No	ns	− 11.89 to 9.577

Table S17. ANOVA of E2 toxicity in HF (comparison of control group and polymer-treated cells)

Dunnett's Multiple Comparison Test	Mean Diff.	q	Significant? P < 0.05?	Summary	95% CI of diff
NT vs 0.78μg/ml	− 1.633	0.267	No	ns	− 19.58 to 16.32
NT vs 1.56 μg/ml	− 0.767	0.125	No	ns	− 18.72 to 17.18
NT vs 3.13 μg/ml	− 3.500	0.572	No	ns	− 21.45 to 14.45
NT vs 6.25 μg/ml	− 1.067	0.174	No	ns	− 19.02 to 16.88
NT vs 12.50 μg/ml	− 3.933	0.643	No	ns	− 21.88 to 14.02
NT vs 25 μg/ml	− 1.133	0.185	No	ns	− 19.08 to 16.82
NT vs 50 μg/ml	4.100	0.670	No	ns	− 13.85 to 22.05
NT vs 100 μg/ml	5.567	0.910	No	ns	− 12.38 to 23.52

Table S18. ANOVA of P2 toxicity in HF (comparison of control group and polymer-treated cells)

Dunnett's Multiple Comparison Test	Mean Diff.	q	Significant? P < 0.05?	Summary	95% CI of diff
NT vs 0.78 ug/ml	− 2.648	0.715	No	ns	− 13.21 to 7.92
NT vs 1.56 ug/ml	− 0.808	0.218	No	ns	− 11.37 to 9.76
NT vs 3.13 ug/ml	− 4.243	1.146	No	ns	− 14.81 to 6.32
NT vs 6.25 ug/ml	− 3.033	0.758	No	ns	− 14.44 to 8.38
NT vs 12.50 ug/ml	− 1.582	0.427	No	ns	− 12.15 to 8.98
NT vs 25.00 ug/ml	− 0.505	0.136	No	ns	− 11.07 to 10.06
NT vs 50.00 μg/ml	− 7.055	1.905	No	ns	− 17.62 to 3.51
NT vs 100.00 μg/ml	− 2.702	0.676	No	ns	− 14.11 to 8.71

Table S19. ANOVA of F2 toxicity in rMSC (comparison of control group and polymer-treated cells)

Dunnett's Multiple Comparison Test	Mean Diff.	q	Significant? P < 0.05?	Summary	95% CI of diff
NT vs 0.78 µg/ml	− 0.960	0.153	No	ns	− 19.41 to 17.49
NT vs 1.56 µg/ml	− 3.107	0.494	No	ns	− 21.56 to 15.35
NT vs 3.13 µg/ml	3.637	0.579	No	ns	− 14.82 to 22.09
NT vs 6.25 µg/ml	4.680	0.744	No	ns	− 13.77 to 23.13
NT vs 12.5µg/ml	− 0.1233	0.020	No	ns	− 18.58 to 18.33
NT vs 25 µg/ml	− 0.350	0.056	No	ns	− 18.80 to 18.10
NT vs 50 µg/ml	5.380	0.856	No	ns	− 13.07 to 23.83
NT vs 100 µg/ml	2.373	0.378	No	ns	− 16.08 to 20.83

Table S20. ANOVA of I2 toxicity in rMSC (comparison of control group and polymer-treated cells)

Dunnett's Multiple Comparison Test	Mean Diff.	q	Significant? P < 0.05?	Summary	95% CI of diff
NT vs 0.78 µg/ml	− 1.362	0.300	No	ns	− 19.63 to 4.80
NT vs 1.56 µg/ml	2.135	0.470	No	ns	− 14.28 to 10.16
NT vs 3.13 µg/ml	5.230	1.151	No	ns	− 12.45 to 11.99
NT vs 6.25 µg/ml	5.923	1.303	No	ns	− 14.08 to 10.36
NT vs 12.5µg/ml	5.690	1.252	No	ns	− 10.05 to 14.39
NT vs 25 µg/ml	8.255	1.816	No	ns	− 7.18 to 17.25
NT vs 50 µg/ml	6.415	1.414	No	ns	− 6.07 to 18.37
NT vs 100 µg/ml	6.458	1.421	No	ns	− 4.10 to 20.34

Table S21. ANOVA of E2 toxicity in rMSC (comparison of control group and polymer-treated cells)

Dunnett's Multiple Comparison Test	Mean Diff.	q	Significant? P < 0.05?	Summary	95% CI of diff
NT vs 0.78µg/ml	− 0.767	0.080	No	ns	− 29.07 to 27.54
NT vs 1.56 µg/ml	0.833	0.086	No	ns	− 27.47 to 29.14
NT vs 3.13 µg/ml	1.867	0.194	No	ns	− 26.44 to 30.17
NT vs 6.25 µg/ml	− 4.267	0.442	No	ns	− 32.57 to 24.04
NT vs 12.50 µg/ml	− 5.700	0.591	No	ns	− 34.01 to 22.61
NT vs 25 µg/ml	1.567	0.163	No	ns	− 26.74 to 29.87
NT vs 50 µg/ml	− 0.633	0.066	No	ns	− 28.94 to 27.67
NT vs 100 µg/ml	− 3.567	0.370	No	ns	− 31.87 to 24.74

Table S22. ANOVA of P2 toxicity in rMSC (comparison of control group and polymer-treated cells)

Dunnett's Multiple Comparison Test	Mean Diff.	q	Significant? P < 0.05?	Summary	95% CI of diff
NT vs 0.78 µg/ml	− 7.410	1.780	No	ns	− 19.63 to 4.81
NT vs 1.56 µg/ml	− 2.057	0.494	No	ns	− 14.28 to 10.16
NT vs 3.13 µg/ml	− 0.227	0.054	No	ns	− 12.45 to 11.99
NT vs 6.25 µg/ml	− 1.857	0.446	No	ns	− 14.08 to 14.36
NT vs 12.5µg/ml	2.170	0.521	No	ns	− 10.05 to 14.39
NT vs 25 µg/ml	5.033	1.209	No	ns	− 7.19 to 17.25
NT vs 50 µg/ml	6.147	1.476	No	ns	6.07 to 18.37
NT vs 100 µg/ml	8.117	1.950	No	ns	− 4.10 to 20.34

S9.2. Cellular uptake

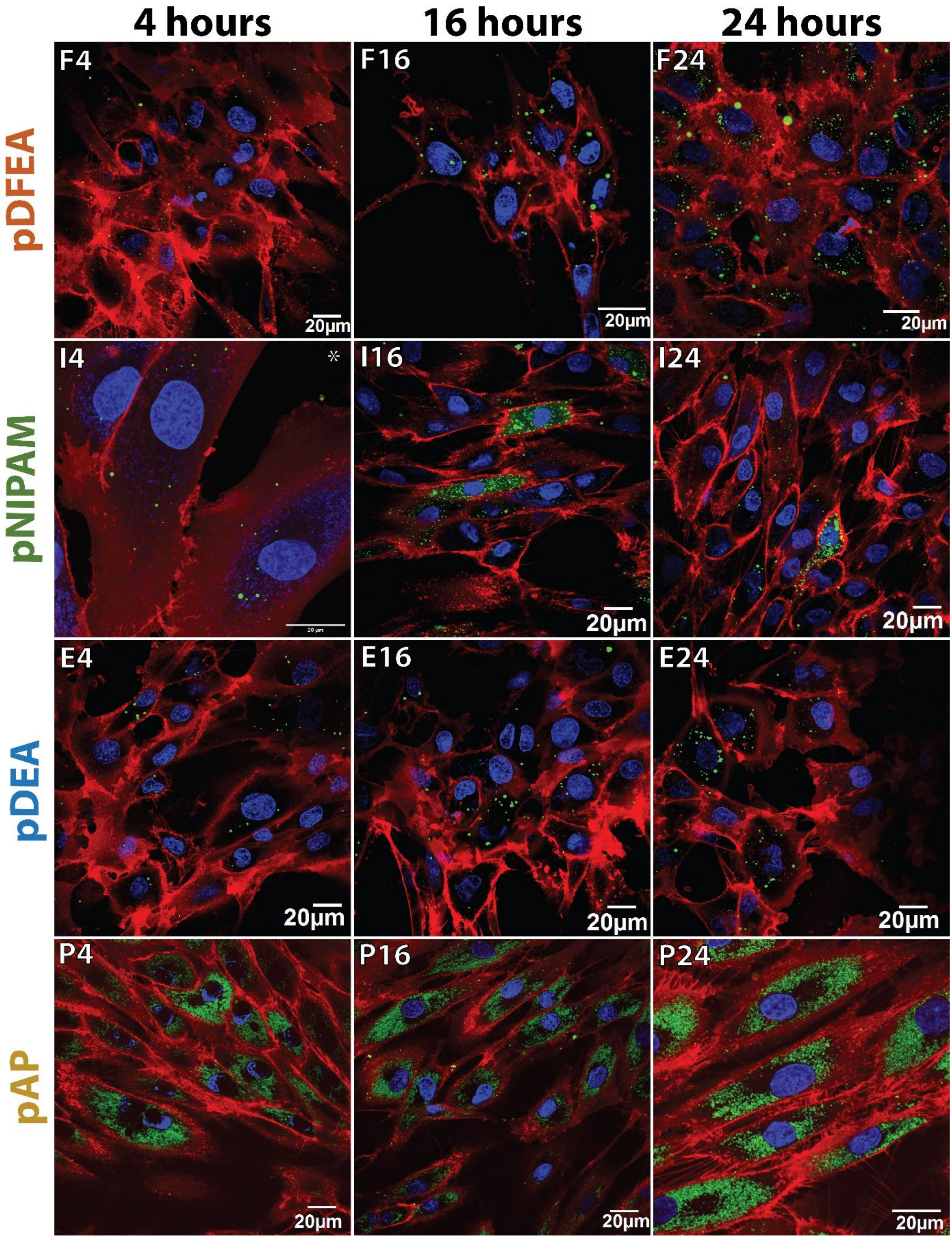

Figure S57. Cellular uptake of Dy505-labelled polymers into HF cells as a function of time. Polymer is shown in **green** (Dy505), cellular membranes in **red** (CellMask™ Deep red), and nucleus in **blue** (Hoechst 33342). Asterisk (*) indicates images acquires with different microscope.

S9.3. Cellular uptake assay – polymer-lysosome colocalization

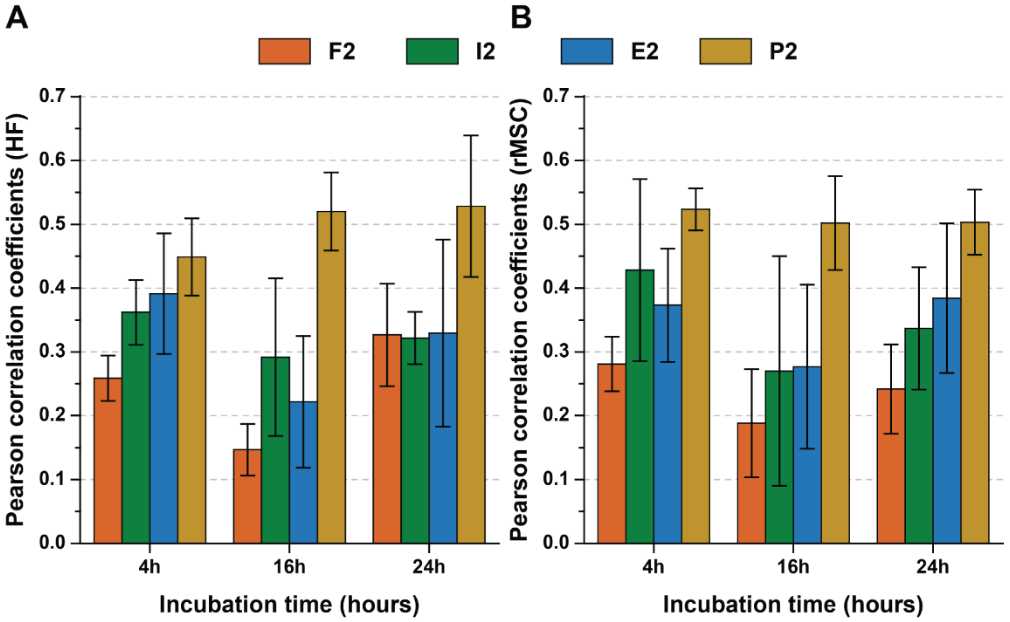

Figure S58. Pearson correlation coefficient of Dy505-labelled polymers (**F2**, **I2**, **E2**, and **P2**) and Lysotracker as a function of time in (HF; **A**) and rat mesenchymal stem cell (rMSC, **B**)

Table S23. Pearson correlation coefficient of polymers and Lysotracker in HF as a function of time

time (h)	F2		I2		E2		P2	
	mean	SD	mean	SD	mean	SD	mean	SD
4	0.259	0.036	0.362	0.051	0.391	0.094	0.449	0.060
16	0.147	0.040	0.292	0.124	0.222	0.103	0.520	0.061
24	0.327	0.080	0.322	0.041	0.330	0.147	0.528	0.111

Table S24. Pearson correlation coefficient of polymers and Lysotracker in rMSC as a function of time

time (h)	F2		I2		E2		P2	
	mean	SD	mean	SD	mean	SD	mean	SD
4	0.281	0.043	0.428	0.143	0.373	0.089	0.523	0.033
16	0.188	0.085	0.270	0.180	0.277	0.129	0.502	0.074
24	0.242	0.070	0.337	0.096	0.384	0.117	0.503	0.051

S10. *Ex vivo* experiments – results overview

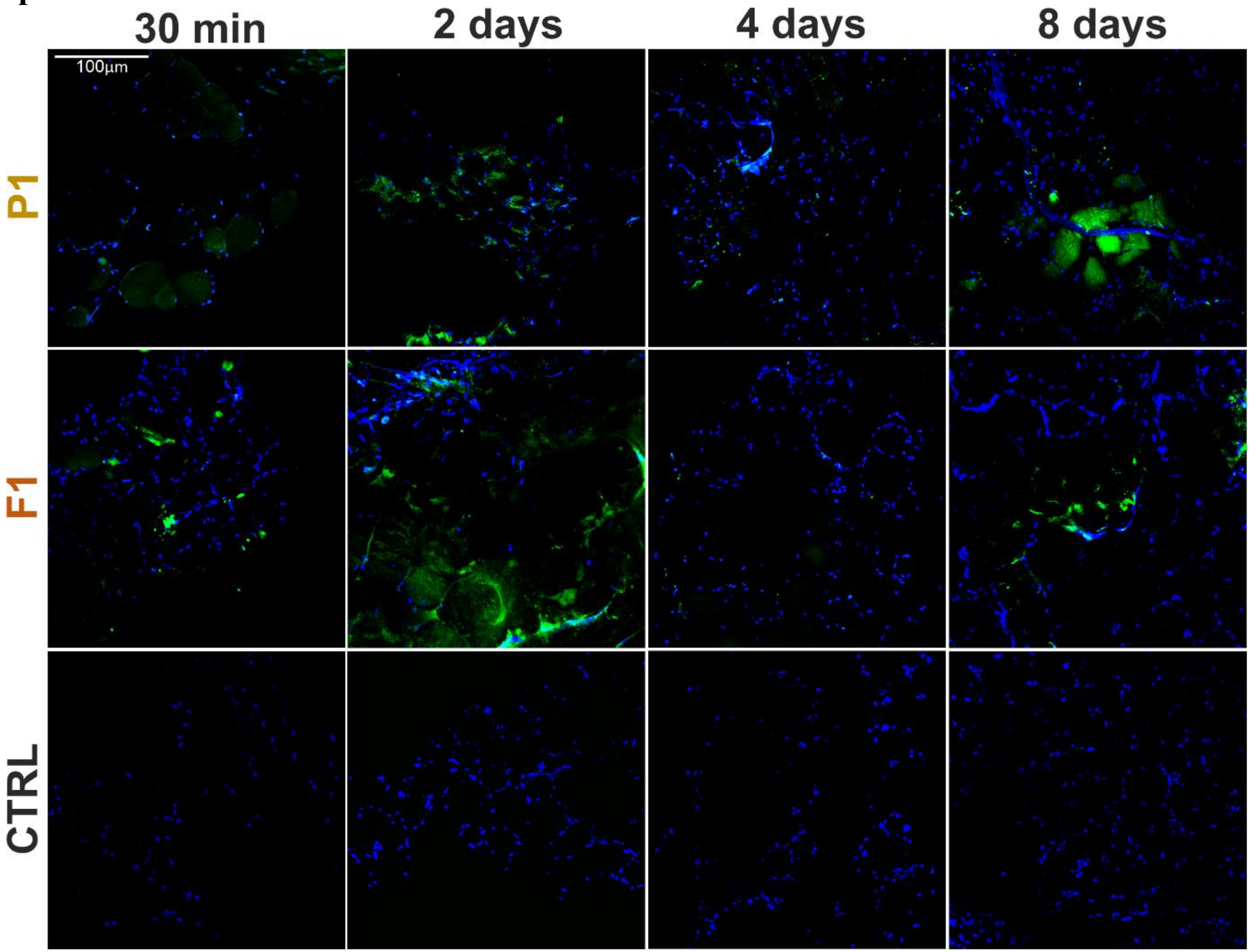

Figure S59. *Ex vivo* micrograms of muscles of **P1**, **F1** or **DMSO mice (CTRL)** at various timepoints; cell nuclei were shown in **blue** (Hoechst 33258) and polymer (Cy7-labelled) shown in **green**.

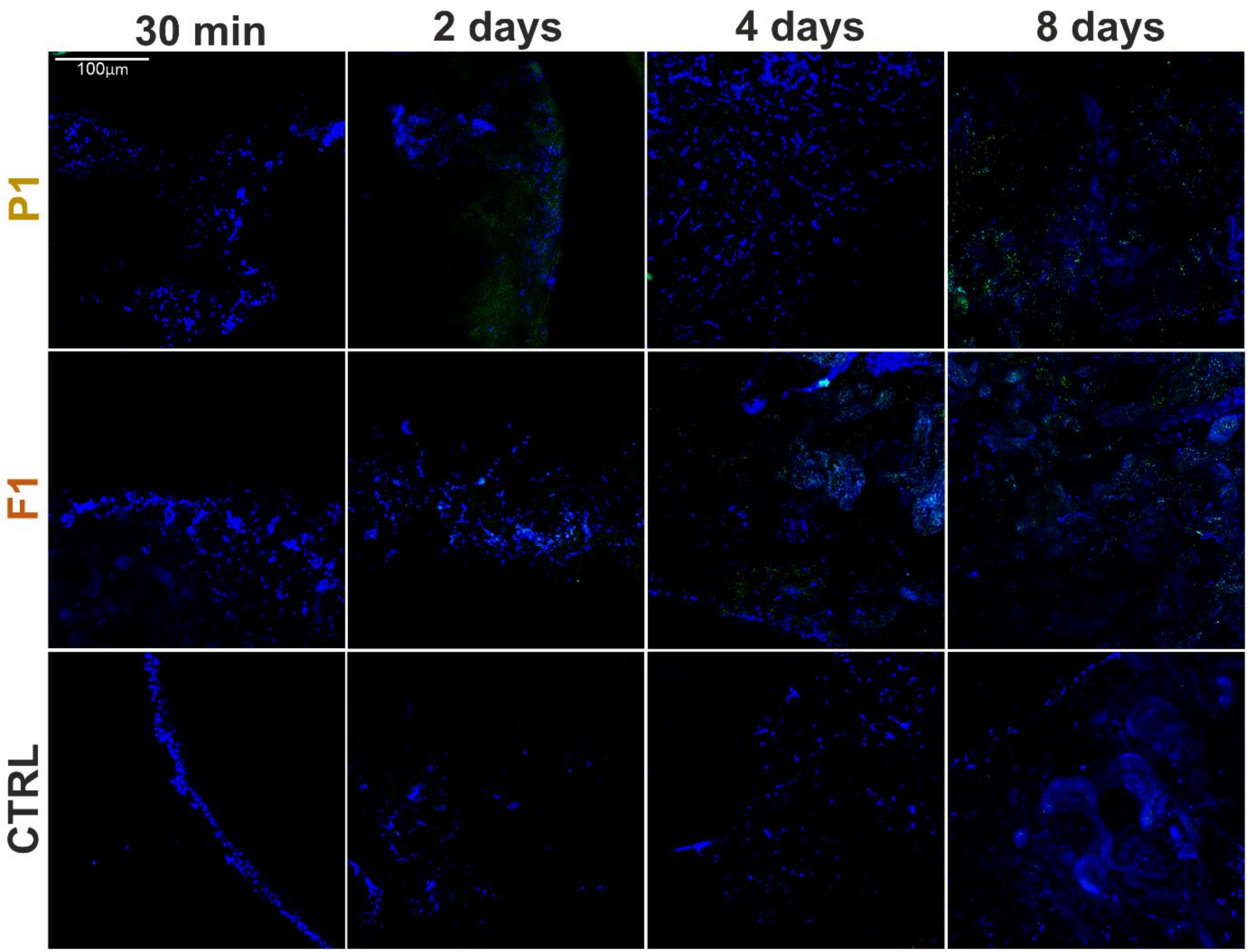

Figure S60. *Ex vivo* micrograms of kidneys of **P1**, **F1** or **DMSO mice (CTRL)** at various timepoints; cell nuclei were shown in **blue** (Hoechst 33258) and polymer (Cy7-labelled) shown in **green**.

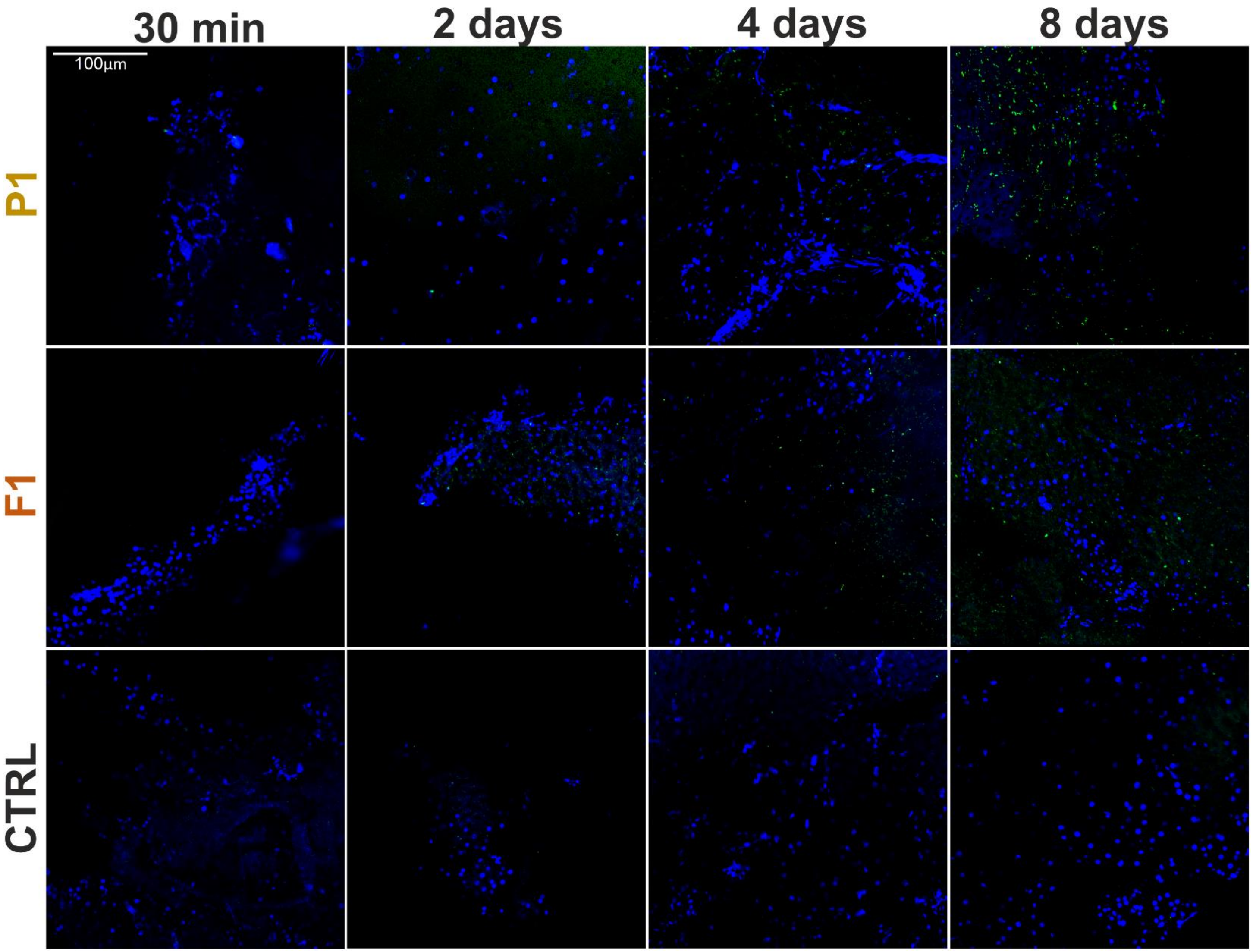

Figure S61. *Ex vivo* micrograms of liver of **P1**, **F1** or **DMSO mice (CTRL)** at various timepoints; cell nuclei were shown in **blue** (Hoechst 33258) and polymer (Cy7-labelled) shown in **green**.

S11. Polymer biodistribution

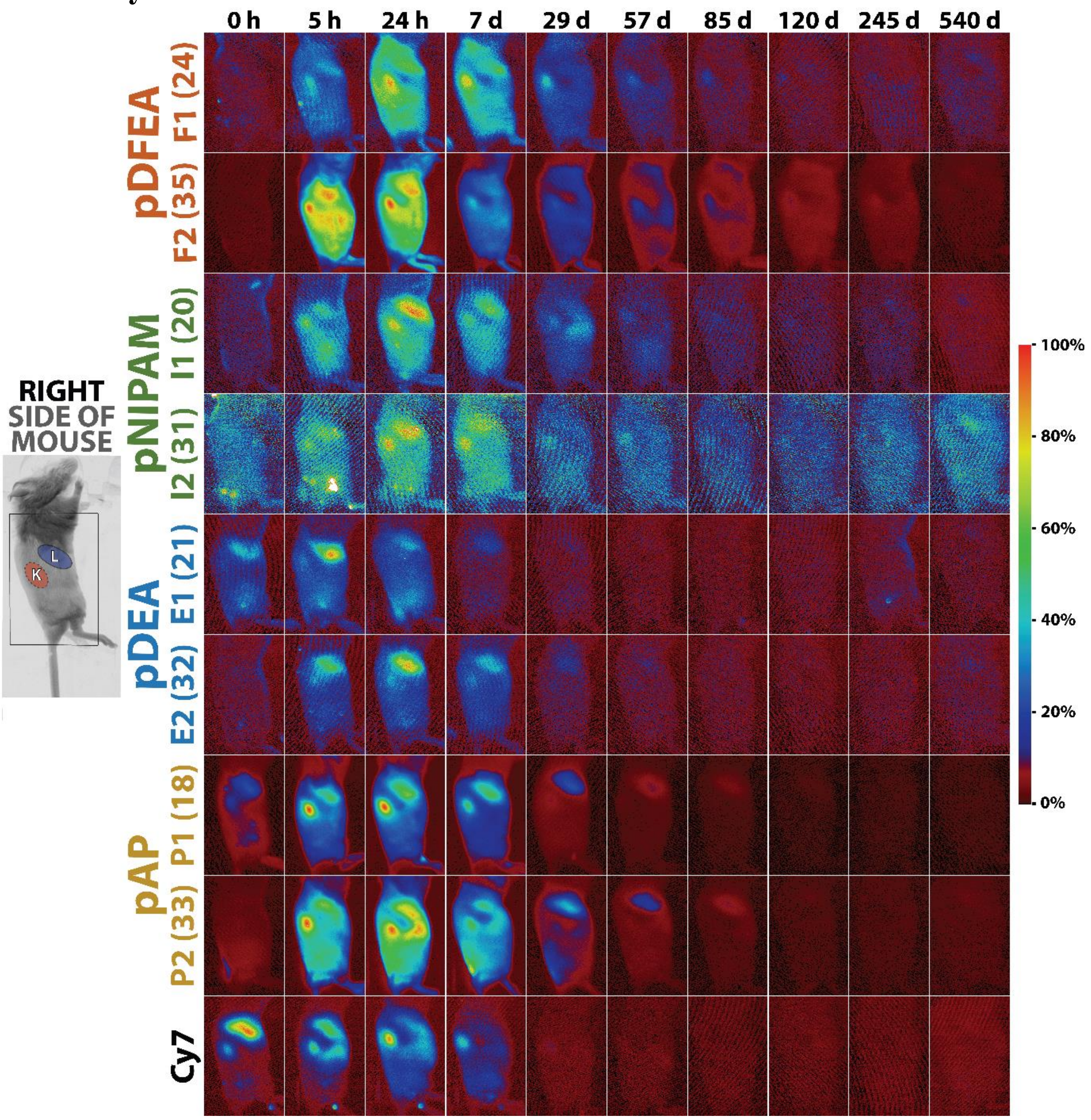

Figure S62. Biodistribution of polymers **F1** (mouse 1), **F2** (mouse 3), **I1** (mouse 2), **I2** (mouse 3), **E1** (mouse 2), **E2** (mouse 2), **P1** (mouse 2), **P2** (mouse 1), and **Cy7** (mouse 2) on the right side of mice (kidney and liver) in various timepoints. Note, that the intensity of each polymer is normalized to its maximum and shown as **relative intensity of that maxima** (see the scalebar on the left). The mouse **I2** at time 5 hours contains a small, localized hotspot of signal, which is caused by a urine droplet on its fur. Identical plot, but normalized to maximum signal of all polymers, is depicted in **Figure S63**.

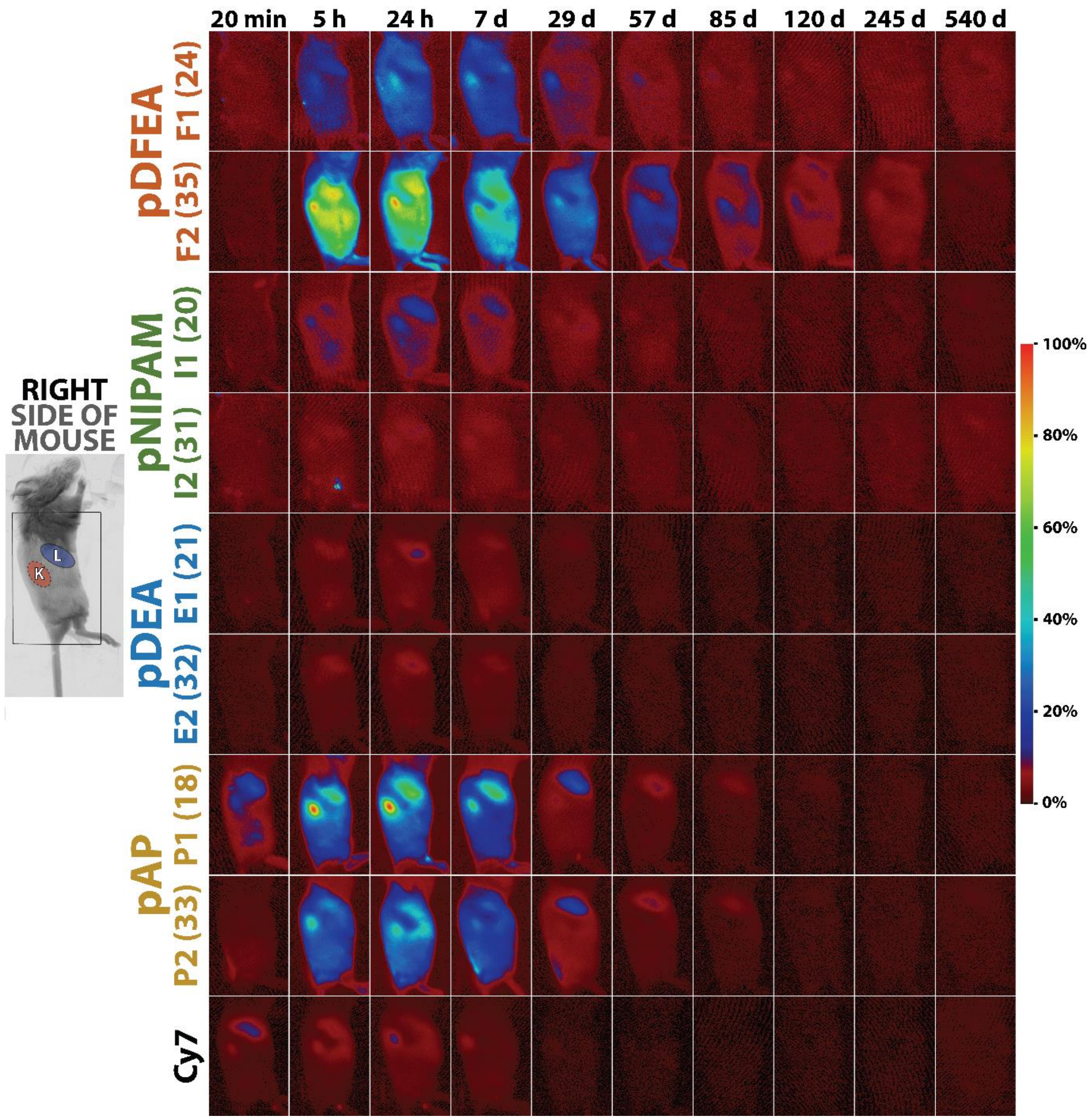

Figure S63. Biodistribution of polymers **F1** (mouse 1), **F2** (mouse 3), **I1** (mouse 2), **I2** (mouse 3), **E1** (mouse 2), **E2** (mouse 2), **P1** (mouse 2), **P2** (mouse 1), and **Cy7** (mouse 2) on the right side of mice (kidney and liver) in various timepoints. Note, that the intensity of each polymer is normalized to **maximum signal of all polymers** and shown as relative intensity of that maxima (see the scalebar on the left). The mouse **I2** at time 5 hours contains a small, localized hotspot of signal, which is caused by a urine droplet on its fur. Identical plot, but normalized to relative intensity of maxima of each individual polymer, is depicted in **Figure S62**.

S12. Polymer biodistribution & dissolution kinetics

S12.1. Polymer pharmacokinetics - raw data

S12.1.1. Intramuscular depot dissolution data – signal intensity (I_{IM})

Table S25. Signal of polymer **F1** (arbitrary units) in M1, M2, and M3 as a function of time.

Mouse M1		Mouse M2		Mouse M3	
**Time (d)**	**Signal (*I*)**	**Time (d)**	**Signal (*I*)**	**Time (d)**	**Signal (*I*)**
0.01244213	25957626	0.014189815	83917072	0.009328704	190026320
0.01443287	27678254	0.016458333	95241720	0.011180556	211972400
0.244375	55298468	0.22849537	180030944	0.223391204	917531200
0.246261574	57023088	0.230335648	186992192	0.225486111	929452736
1.03962963	57310884	0.98337963	158462768	1.046111111	468554048
1.042141204	58292248	0.985891204	159184672	1.048275463	483069440
1.991134259	54674204	1.934884259	142036848	2.044594907	364427168
1.993634259	54652988	1.937384259	143845200	2.049201389	372674976
2.9325	51736156	2.87625	126799968	2.975231481	304267744
2.935949074	52796108	2.879699074	126081080	2.977430556	323473472
6.96431713	48973724	6.90806713	110869032	7.158125	237465072
6.966770833	47924988	6.910520833	113124816	7.160231481	246224592
14.96072917	30340692	14.90447917	79407608	14.94936343	153623600
14.96314815	30407310	14.90689815	79212048	14.95398148	155853696
28.99543981	26498982	28.93918981	67894992	28.87645833	125645232
28.99820602	26304410	28.94195602	68481384	28.87976852	130312640
56.11059028	22579914	56.05434028	49932912	56.97891204	95228152
56.11278935	22397344	56.05653935	49627600	56.98115741	97688368
70.25825231	17387302	70.20200231	35650212	70.17987269	76206808
70.26082176	18512988	70.20457176	37326084	70.18262731	77406224
86.11630787	19576386	86.06005787	39666512	85.96809028	83188280
86.11840278	20445624	86.06215278	39213048	85.97021991	82849088
122.0740278	14566728	122.0177778	26173742	122.9824884	61837632
122.0763773	15499827	122.0201273	26304792	122.9846412	64596844
150.0428356	18596696	149.9865856	31037436	149.9269676	72756240
150.0453472	19771872	149.9890972	31204064	149.9317245	73245760
184.0263426	13905249	183.9700926	18930802	183.0573495	55404496
184.0284954	14709808	183.9722454	18987610	183.0620139	56394268
247.0727546	14286670	**Mouse euthanized on day 202**		247.017535	44202740
247.0747454	14082511			247.019525	42660968

Table S26. Signal of polymer **F2** (arbitrary units) in M1, M2, and M3 as a function of time.

**Mouse M1**		**Mouse M2**		**Mouse M3**	
**Time (d)**	**Signal (*I*)**	**Time (d)**	**Signal (*I*)**	**Time (d)**	**Signal (*I*)**
0.015069444	340801664	0.019097222	432331648	0.020185185	249138208
0.016851852	360192704	0.021354167	484502336	0.022141204	256096928
0.222488426	1010607040	0.225324074	927166912	0.229814815	893996992
0.224259259	1040832512	0.227835648	952418240	0.231770833	902395648
1.032222222	566592320	1.011388889	461609376	1.044039352	1088721280
1.034386574	557797120	1.013553241	470257024	1.046574074	1114516608
2.030706019	435868704	2.009872685	349224704	2.042719907	1023008832
2.0353125	438836480	2.014479167	358422560	2.043680556	962624384
2.961342593	358699328	2.940509259	315623136	5.341053241	559845760
2.963541667	358993920	2.942708333	316080768	5.343483796	580132672
7.144236111	274032608	7.123402778	238233792	6.082465278	514647200
7.146342593	276000864	7.125509259	240017648	6.085023148	527183936
14.93547454	176498080	14.9146412	151749776	15.16583333	90180040
14.94009259	173488352	14.91925926	150451968	15.16923611	91719056
28.86256944	120689656	28.84173611	115414824	29.27958333	22638288
28.86587963	124893384	28.8450463	114951728	29.28241898	23657810
56.96502315	97337648	56.94418981	83716128	57.31351852	9477001
56.96726852	98842584	56.94643519	81753256	57.31608796	9553615
70.1659838	68082416	70.14515046	57006752	69.45393519	7223520
70.16873843	64409948	70.14790509	56039388	69.45649306	7147964.5
85.95420139	74639640	85.93336806	70337024	85.18834491	7205311.5
85.95633102	75313120	85.93549769	70893984	85.19056713	7212894.5
122.9685995	55345720	122.9477662	55457960	121.0998843	6631809
122.9707523	56400520	122.949919	56959040	121.1071065	6629061
149.9130787	69572720	149.8922454	56260208	149.2655208	7189367
149.9178356	71378416	149.8970023	59530416	N/A	N/A
183.0434606	53679368	183.0226273	45180368	183.2483102	6047537.5
183.048125	54457724	183.0272917	45244128	183.2510417	6045141
**Mouse euthanized on day 202**		247.054838	37204344	246.2715972	6762824
		247.0611921	38386484	246.2734259	6915175

Table S27. Signal of polymer **I1** (arbitrary units) in M1, M2, and M3 as a function of time.

**Mouse M1**		**Mouse M2**		**Mouse M3**	
**Time (d)**	**Signal (*I*)**	**Time (d)**	**Signal (*I*)**	**Time (d)**	**Signal (*I*)**
0.025300926	143215184	0.015717593	100533424	0.011967593	185751584
0.028946759	157976912	0.017708333	107178376	0.014131944	206442960
0.220983796	314254496	0.218935185	262110992	0.221793981	578975168
0.222905093	334119488	0.225069444	262991856	0.223634259	572804736
1.023333333	365639040	1.021909722	307025952	1.029490741	473039520
1.025451389	363775104	1.024351852	287814880	1.031886574	488074880
2.03162037	375665280	2.001064815	357782560	1.980231481	501322752
2.034016204	407481344	2.003460648	358416160	1.982627315	497877120
3.030578704	369666496	3.000023148	306527552	2.979189815	487928608
3.032696759	363574368	3.002141204	311701792	2.98130787	486874784
7.209097222	332760992	7.178541667	283894816	7.157708333	489258592
7.211400463	344146336	7.180844907	275613984	7.160011574	507128928
14.99456019	254090608	14.96400463	196642400	14.9431713	369084992
14.99645833	257974640	14.96590278	201789872	14.94506944	359020704
28.92715278	225576672	28.89659722	165463408	28.87576389	296994368
28.9296412	226237040	28.89908565	170167504	28.87825231	286796192
57.0305787	118027248	57.00002315	107240568	56.97918981	176439264
57.03320602	119511968	57.00265046	110515272	56.98181713	177989696
70.18325231	60378156	70.15269676	55330684	70.13186343	99577728
70.1865625	57671532	70.15600694	56173284	70.13517361	98887832
86.01891204	67744384	85.98835648	64263008	85.96752315	119414680
86.02116898	66810436	85.99061343	65167016	85.96978009	121270856
123.0411343	34933100	123.0105787	32617866	123.1425463	60515440
123.0437616	35101848	123.013206	32920512	123.1445718	57876596
149.9797106	37748608	149.9491551	46381040	149.9283218	58223228
149.9831019	38395028	149.9525463	47018872	149.931713	58431320
183.1138542	23635082	183.0832986	30575534	183.0624653	41892872
183.1160995	23183960	183.085544	31067572	183.0647106	40838820
**Mouse euthanized on day 202**		247.1143866	21446996	247.0935532	29231516
		247.1165856	22018826	247.0957523	28087536

Table S28. Signal of polymer **I2** (arbitrary units) in M1, M2, and M3 as a function of time.

**Mouse M1**		**Mouse M2**		**Mouse M3**	
**Time (d)**	**Signal (*I*)**	**Time (d)**	**Signal (*I*)**	**Time (d)**	**Signal (*I*)**
0.031747685	64980644	0.017430556	95429576	0.013449074	146998080
0.034502315	65359336	0.019305556	98016072	0.015416667	163632464
0.241689815	129580912	0.268726852	143738672	0.25130787	290064928
0.243599537	125293256	0.270798611	141951504	0.253483796	285110784
1.072581019	153879632	1.030219907	172766448	0.998275463	248504688
1.074884259	155293424	1.032523148	146757856	1.000578704	252873728
2.034224537	145943216	1.991863426	149075792	1.959918981	246223552
2.036574074	150655584	1.994212963	156525296	1.962268519	246515776
3.025543981	162225056	2.98318287	173917408	2.951238426	276376192
3.02806713	161331136	2.985706019	174999568	2.953761574	272259552
7.054305556	151508192	7.011944444	166975968	6.98	270575200
7.056574074	151414800	7.014212963	163225456	6.982268519	282449280
15.04621528	111417232	15.00385417	134195320	14.97190972	213444576
15.04922454	114923768	15.00686343	123403440	14.97491898	221140224
29.18171296	83223152	29.13935185	134171840	29.10740741	200459808
29.184375	83471440	29.14201389	135673456	29.11006944	203175040
56.17017361	57604160	56.1278125	87441560	56.09586806	123944208
56.17436343	59953452	56.13200231	91131552	56.10005787	132561240
70.34950231	36916708	70.3071412	71433264	70.27519676	88837824
70.35253472	36585616	70.31017361	71241936	70.27822917	89002552
86.21047454	35865064	86.16811343	63293132	86.13616898	77865904
86.21262731	38984768	86.1702662	64812976	86.13832176	76235568
122.1678819	27067800	122.1255208	38993496	122.0935764	43877104
122.1702546	26993084	122.1278935	41979688	122.0959491	44690776
150.1361458	30947912	150.0937847	44202452	150.0618403	40706808
150.1383565	29800912	150.0959954	40369788	150.0640509	42042604
184.122338	20706556	184.0799769	30810938	184.0480324	26594326
184.1271875	22337158	184.0848264	29311992	184.0528819	27136488
247.1662153	15794463	**Mouse euthanized on day 202**		247.0919097	22556408
247.1682523	15867012			247.0939468	21136026

Table S29. Signal of polymer **E1** (arbitrary units) in M1, M2, and M3 as a function of time.

**Mouse M1**		**Mouse M2**		**Mouse M3**	
**Time (d)**	**Signal (*I*)**	**Time (d)**	**Signal (*I*)**	**Time (d)**	**Signal (*I*)**
0.010578704	349893824	0.014953704	213338816	0.010578704	385364000
0.012777778	374041152	0.016851852	230850496	0.012777778	398958432
0.224386574	812435584	0.226712963	554180288	0.224386574	663331584
0.226481481	820444992	0.229027778	556256512	0.226481481	637924800
1.034456019	993825024	1.02056713	791030784	1.034456019	751210048
1.036493056	1001030336	1.022604167	782379904	1.036493056	830054976
2.0315625	895042304	2.017673611	794146880	2.0315625	755950720
2.034050926	886513216	2.020162037	779969472	2.034050926	727374144
2.909398148	933505472	2.895509259	806103232	2.909398148	832193984
2.911377315	966904576	2.897488426	769901952	2.911377315	770896192
7.092604167	853865472	7.078715278	675032768	7.092604167	718428416
7.094976852	862389888	7.081087963	683998592	7.094976852	720446144
14.88707176	372094912	14.87318287	312967648	14.88707176	290271744
14.88952546	366239072	14.87563657	322863520	14.88952546	291939232
28.83755787	143084432	28.82366898	136204880	28.83755787	119196896
28.84013889	143699888	28.82625	139448352	28.84013889	119538968
56.96059028	60306888	56.94670139	59222100	56.96059028	49660044
56.96258102	60331508	56.94869213	59782416	56.96258102	51486552
70.22501157	29836512	70.21112269	25991218	70.22501157	20193040
70.2371412	28887714	70.22325231	27270022	70.2371412	19681956
85.99033565	23145100	85.97644676	23523188	85.99033565	19460690
85.99273148	23994628	85.97884259	23264746	85.99273148	19901046
122.9238657	13297826	122.9099769	11979414	122.9238657	11484194
122.9260069	13767590	122.9121181	12213414	122.9260069	11743833
149.8670949	13515006	149.853206	11695518	149.8670949	10984505
149.8699306	13491124	149.8560417	11771038	149.8699306	11044230
182.9947801	9747064	182.9808912	8476650	182.9947801	8081430
182.9980903	9722101	182.9842014	8377644.5	182.9980903	8181074
**Mouse euthanized on day 202**		247.9501	8206125	**Mouse euthanized on day 202**	
		247.95214	8110692.5		

Table S30. Signal of polymer **E2** (arbitrary units) in M1, M2, and M3 as a function of time.

**Mouse M1**		**Mouse M2**		**Mouse M3**	
**Time (d)**	**Signal (*I*)**	**Time (d)**	**Signal (*I*)**	**Time (d)**	**Signal (*I*)**
0.013229167	301036928	0.0234375	530360960	0.034965278	1040781120
0.015289352	333977120	0.025266204	585007680	0.035914352	1134969984
0.232534722	783102528	0.255555556	984005888	0.244444444	1383676288
0.236168981	762129984	0.25625	937138048	0.245138889	1402174080
1.077766204	916360000	1.065266204	977078272	1.054155093	1201652352
1.080115741	941062848	1.067615741	982585408	1.05650463	1099477120
2.060162037	877461824	2.047662037	904439616	2.036550926	1150306304
2.062291667	915463168	2.049791667	925602240	2.038680556	1113714688
3.050717593	897370560	3.038217593	899897984	3.027106481	1147380736
3.053425926	907556032	3.040925926	899787136	3.029814815	1129490560
7.078657407	859903296	7.066157407	823148096	7.055046296	1020280384
7.081180556	849146368	7.068680556	801668992	7.057569444	1003737664
15.1159375	441248512	15.1034375	334301344	15.09232639	542637888
15.11893519	458211296	15.10643519	318406528	15.09532407	531138400
29.1940162	202405696	29.1815162	111768968	29.17040509	236762640
29.19615741	202295024	29.18365741	117829000	29.1725463	242656576
56.19212963	85412640	56.17962963	57373848	56.16851852	132766680
56.19475694	85152488	56.18225694	58112296	56.17114583	136595856
70.36564815	32225534	70.35314815	25834826	70.34203704	66545620
70.36997685	31738984	70.35747685	27948976	70.34636574	67578024
86.18175926	26620734	86.16925926	28709312	86.15814815	44876404
86.18390046	27662128	86.17140046	23295102	86.16028935	46440088
122.1954282	12260033	122.1829282	10976330	122.1718171	21630480
122.1973843	12444951	122.1848843	11196361	122.1737731	21785470
150.1613657	13454042	150.1488657	12086440	150.1377546	20669304
150.1636574	13612628	150.1511574	12482812	150.1400463	20436808
184.1498843	8987673	184.1373843	9090823	184.1262731	13206468
184.1524421	9125518	184.1399421	8874702	184.128831	13784577
**Mouse euthanized on day 202**		247.181713	8870843	247.1706019	12297370
		247.1842477	9060523	247.1731366	12252568

Table S31. Signal of polymer **P1** (arbitrary units) in M1, M2, and M3 as a function of time.

**Mouse M1**		**Mouse M2**		**Mouse M3**	
**Time (d)**	**Signal (*I*)**	**Time (d)**	**Signal (*I*)**	**Time (d)**	**Signal (*I*)**
0.011805556	541907712	0.028703704	1189102464	0.054027778	1518658688
0.01400463	573348032	0.029409722	1102175872	0.054965278	1528223232
0.235023148	978684928	0.218668981	1239490048	0.213414352	1479864832
0.236944444	997913728	0.220844907	1253599104	0.215532407	1505358592
1.057268519	778135424	1.007962963	759122688	0.98712963	922165952
1.059409722	765108480	1.010104167	784636480	0.989270833	915996544
1.933009259	839902656	1.883703704	796168832	1.86287037	915749888
1.935358796	845786112	1.886053241	826664512	1.865219907	897590208
2.873622685	798048384	2.82431713	850207616	2.803483796	894316224
2.875810185	832522688	2.82650463	827293248	2.805671296	904605952
6.906076389	759480896	6.856770833	869866752	6.8359375	919083904
6.908587963	790780288	6.859282407	829965568	6.838449074	922125888
14.91810185	354724064	14.8687963	381863200	14.84796296	449003488
14.92077546	357256224	14.87146991	377061152	14.85063657	446863584
28.94070602	240464624	28.89140046	240332336	28.87056713	292171328
28.94372685	242689472	28.8944213	260080704	28.87358796	296096992
56.02479167	68969880	55.97548611	68411088	55.95465278	95341712
56.02895833	71227056	55.97965278	74781896	55.95881944	93912840
70.20241898	29158662	70.15311343	29850816	70.13228009	40726092
70.20532407	29043734	70.15601852	29985302	70.13518519	40087312
87.04762731	26560022	86.99832176	27248680	86.97748843	36865884
87.05053241	27099616	87.00122685	28009340	86.98039352	36240772
122.0149884	11601124	121.9656829	12662829	121.9448495	16604948
122.0171181	12088216	121.9678125	12348102	121.9469792	17050712
149.9853588	13161666	149.9360532	12301834	149.9152199	14412654
149.9875231	12822057	149.9382176	12519457	149.9173843	14711841
183.9723264	8264244.5	183.9230208	7745936	183.9021875	9383052
183.9745139	8199316	183.9252083	7550210.5	183.904375	9300700
**Mouse euthanized on day 202**		246.96334	7954653	246.9425116	8659109
		246.96572	7941937.5	246.9448843	8720371

Table S32. Signal of polymer **P2** (arbitrary units) in M1, M2, and M3 as a function of time.

**Mouse M1**		**Mouse M2**		**Mouse M3**	
**Time (d)**	**Signal (*I*)**	**Time (d)**	**Signal (*I*)**	**Time (d)**	**Signal (*I*)**
0.013541667	690910976	0.025300926	143215184	0.015717593	100533424
0.015277778	709256064	0.028946759	157976912	0.017708333	107178376
0.228171296	1107142016	0.220983796	314254496	0.218935185	262110992
0.230138889	1086851072	0.222905093	334119488	0.225069444	262991856
0.980740741	827865792	1.023333333	365639040	1.021909722	307025952
0.983032407	800673984	1.025451389	363775104	1.024351852	287814880
1.994756944	779236032	2.03162037	375665280	2.001064815	357782560
1.997118056	815622464	2.034016204	407481344	2.003460648	358416160
2.911747685	820543488	3.030578704	369666496	3.000023148	306527552
2.913831019	828951872	3.032696759	363574368	3.002141204	311701792
7.091759259	726586560	7.209097222	332760992	7.178541667	283894816
7.096597222	771928128	7.211400463	344146336	7.180844907	275613984
14.88825231	338430208	14.99456019	254090608	14.96400463	196642400
14.89138889	337745152	14.99645833	257974640	14.96590278	201789872
28.83503472	121491024	28.92715278	225576672	28.89659722	165463408
28.83890046	125942592	28.9296412	226237040	28.89908565	170167504
56.92354167	47281468	57.0305787	118027248	57.00002315	107240568
56.92631944	45585820	57.03320602	119511968	57.00265046	110515272
70.22130787	23272072	70.18325231	60378156	70.15269676	55330684
70.22390046	22495332	70.1865625	57671532	70.15600694	56173284
85.91983796	18149982	86.01891204	67744384	85.98835648	64263008
85.92288194	18537340	86.02116898	66810436	85.99061343	65167016
122.9235301	11041209	123.0411343	34933100	123.0105787	32617866
122.9256481	10648515	123.0437616	35101848	123.013206	32920512
149.864919	10273381	149.9797106	37748608	149.9491551	46381040
149.8678009	10230828	149.9831019	38395028	149.9525463	47018872
182.9984028	7822257	183.1138542	23635082	183.0832986	30575534
183.000706	7765702	183.1160995	23183960	183.085544	31067572
247.9684375	8519921	**Mouse euthanized on day 202**		247.1143866	21446996
247.970463	8231383.5			247.1165856	22018826

Table S33. Signal of **Cy7-amine** (arbitrary units) in M1, M2, and M3 as a function of time.

**Mouse M1**		**Mouse M2**		**Mouse M3**	
**Time (d)**	**Signal (*I*)**	**Time (d)**	**Signal (*I*)**	**Time (d)**	**Signal (*I*)**
0.020185185	249138208	0.020960648	772553856	0.016712963	143618816
0.022141204	256096928	0.023159722	790062016	0.018668981	137589648
0.229814815	893996992	0.227604167	1148956288	0.226342593	128547328
0.231770833	902395648	0.229560185	1089529600	0.228298611	123893464
1.044039352	1088721280	1.016956019	1365878784	1.04056713	122648904
1.046574074	1114516608	1.019490741	1273627136	1.043101852	122605280
2.042719907	1023008832	2.015636574	1222285056	2.039247685	91812512
2.043680556	962624384	2.016597222	1185499008	2.040208333	88040456
5.341053241	559845760	5.313969907	768923264	5.337581019	54088240
5.343483796	580132672	5.316400463	771988544	5.340011574	51116496
6.082465278	514647200	6.055381944	708882304	6.078993056	48302700
6.085023148	527183936	6.057939815	712067200	6.081550926	54251028
15.16583333	90180040	15.13875	124695352	15.16236111	14094676
15.16923611	91719056	15.14215278	126557568	15.16576389	13814838
29.27958333	22638288	29.2525	31964962	29.27611111	6996515.5
29.28241898	23657810	29.25533565	32630328	29.27894676	7180365.5
57.31351852	9477001	57.28643519	11327991	57.3100463	5378527
57.31608796	9553615	57.28900463	11397725	57.31261574	5415778.5
69.45393519	7223520	69.42685185	7498180	69.45046296	5214868.5
69.45649306	7147964.5	69.42940972	7524230.5	69.45302083	5150796.5
85.18834491	7205311.5	85.16126157	7386399	85.18487269	5693890.5
85.19056713	7212894.5	85.1634838	7345141	85.18709491	5473707.5
121.0998843	6631809	121.0728009	6357946	121.096412	6146471
121.1071065	6629061	121.0800231	6407366.5	121.1036343	6012256
149.2655208	7189367	149.2384375	6994151.5	149.2620486	6777861
183.2483102	6047537.5	183.2212269	5633007	183.244838	5927238.5
183.2510417	6045141	183.2239583	5708342	183.2475694	5722120
246.2715972	6762824	246.2445139	6277955.5	**Mouse euthanized on day 202**	
246.2734259	6915175	246.2463426	6378515		

S12.1.2. Intramuscular depot dissolution data – depot area (S_{IM})

Table S34. Area of polymer depot **F1** (pixels) in M1, M2, and M3 as a function of time.

Mouse M1		Mouse M2		Mouse M3	
**Time (d)**	**Area (*S*)**	**Time (d)**	**Area (*S*)**	**Time (d)**	**Area (*S*)**
0.01244213	21986	0.018391204	30631	0.014189815	29533
0.01443287	23345	0.020810185	32661	0.016458333	32576
0.244375	34337	0.231111111	43003	0.22849537	42995
0.246261574	34769	0.232951389	42728	0.230335648	44169
1.03962963	44760	1.002824074	47276	0.98337963	48541
1.042141204	46699	1.005335648	47063	0.985891204	48770
1.991134259	45848	1.954328704	46660	1.934884259	46909
1.993634259	45961	1.956828704	46175	1.937384259	47616
2.9325	43115	2.895694444	45195	2.87625	44953
2.935949074	43130	2.899143519	45044	2.879699074	44252
6.96431713	43876	6.927511574	44488	6.90806713	45488
6.966770833	43139	6.929965278	44218	6.910520833	46680
14.96072917	37946	14.92392361	41580	14.90447917	41708
14.96314815	38054	14.92634259	41588	14.90689815	41960
28.99543981	34194	28.95863426	41015	28.93918981	40533
28.99820602	33736	28.96140046	40637	28.94195602	39861
56.11059028	30172	56.07378472	39276	56.05434028	35280
56.11278935	30567	56.0759838	40012	56.05653935	36339
70.25825231	24927	70.22144676	38032	70.20200231	31036
70.26082176	25293	70.2240162	37375	70.20457176	32232
86.11630787	24010	86.07950231	36015	86.06005787	30942
86.11840278	24642	86.08159722	36867	86.06215278	30816
122.0740278	20713	122.0372222	32050	122.0177778	28089
122.0763773	21604	122.0395718	32190	122.0201273	28274
150.0428356	26631	150.0060301	35845	149.9865856	32122
150.0453472	27008	150.0085417	36197	149.9890972	32131
184.0263426	20550	183.989537	29622	183.9700926	20865
184.0284954	20925	183.9916898	30615	183.9722454	21011
247.0727546	21906	**Mouse euthanized on day 202**		247.017535	21607
247.0747454	21545			247.019525	21908

Table S35. Area of polymer depot F2 (pixels) in M1, M2, and M3 as a function of time.

Mouse M1		Mouse M2		Mouse M3	
**Time (d)**	**Area (*S*)**	**Time (d)**	**Area (*S*)**	**Time (d)**	**Area (*S*)**
0.009328704	44812	0.015069444	53873	0.019097222	55417
0.011180556	47281	0.016851852	54555	0.021354167	56857
0.223391204	59808	0.222488426	59992	0.225324074	59990
0.225486111	59876	0.224259259	59994	0.227835648	59985
1.046111111	59983	1.032222222	59999	1.011388889	59956
1.048275463	59972	1.034386574	59998	1.013553241	59949
2.044594907	59891	2.030706019	59985	2.009872685	59784
2.049201389	59878	2.0353125	59968	2.014479167	59851
2.975231481	59414	2.961342593	59769	2.940509259	59462
2.977430556	59569	2.963541667	59842	2.942708333	59582
7.158125	57343	7.144236111	58855	7.123402778	57680
7.160231481	57738	7.146342593	58912	7.125509259	57918
14.94936343	52379	14.93547454	55091	14.9146412	50006
14.95398148	52436	14.94009259	54062	14.91925926	52209
28.87645833	49667	28.86256944	49529	28.84173611	49880
28.87976852	49630	28.86587963	49909	28.8450463	48964
56.97891204	46551	56.96502315	46326	56.94418981	46609
56.98115741	46588	56.96726852	46918	56.94643519	44610
70.17987269	46590	70.1659838	42555	70.14515046	42060
70.18262731	45897	70.16873843	43120	70.14790509	42071
85.96809028	44319	85.95420139	43916	85.93336806	43804
85.97021991	44501	85.95633102	44366	85.93549769	44205
122.9824884	45239	122.9685995	44807	122.9477662	44341
122.9846412	45203	122.9707523	45181	122.949919	44898
149.9269676	46606	149.9130787	45432	149.8922454	43744
149.9317245	46386	149.9178356	45332	149.8970023	45440
183.0573495	41725	183.0434606	42278	183.0226273	41023
183.0620139	43131	183.048125	43332	183.0272917	42060
247.0895602	40096	**Mouse euthanized on day 202**		247.054838	37703
247.0959144	40812			247.0611921	38134

Table S36. Area of polymer depot **I1** (pixels) in M1, M2, and M3 as a function of time.

**Mouse M1**		**Mouse M2**		**Mouse M3**	
**Time (d)**	**Area (*S*)**	**Time (d)**	**Area (*S*)**	**Time (d)**	**Area (*S*)**
0.025300926	39418	0.015717593	34911	0.011967593	42543
0.028946759	40851	0.017708333	36607	0.014131944	44329
0.220983796	52584	0.218935185	49301	0.221793981	58428
0.222905093	53827	0.225069444	49463	0.223634259	58567
1.023333333	55637	1.021909722	51274	1.029490741	57035
1.025451389	55559	1.024351852	49870	1.031886574	57373
2.03162037	59689	2.001064815	59744	1.980231481	59948
2.034016204	59810	2.003460648	59891	1.982627315	59949
3.030578704	59730	3.000023148	59791	2.979189815	59896
3.032696759	59758	3.002141204	59767	2.98130787	59956
7.209097222	59356	7.178541667	59439	7.157708333	59903
7.211400463	59380	7.180844907	59391	7.160011574	59883
14.99456019	57188	14.96400463	57330	14.9431713	58496
14.99645833	57405	14.96590278	57753	14.94506944	58422
28.92715278	56294	28.89659722	55559	28.87576389	56660
28.9296412	56520	28.89908565	56601	28.87825231	56746
57.0305787	45908	57.00002315	48352	56.97918981	49275
57.03320602	45891	57.00265046	48189	56.98181713	49504
70.18325231	38695	70.15269676	38590	70.13186343	40824
70.1865625	37993	70.15600694	38051	70.13517361	41462
86.01891204	37145	85.98835648	39686	85.96752315	41574
86.02116898	36765	85.99061343	39014	85.96978009	41666
123.0411343	26867	123.0105787	28486	123.1425463	30344
123.0437616	26998	123.013206	28275	123.1445718	28778
149.9797106	32197	149.9491551	36388	149.9283218	34608
149.9831019	33321	149.9525463	37215	149.931713	34558
183.1138542	25125	183.0832986	29348	183.0624653	27538
183.1160995	25393	183.085544	29788	183.0647106	24650
**Mouse euthanized on day 202**		247.1143866	25602	247.0935532	21685
		247.1165856	25829	247.0957523	20011

Table S37. Area of polymer depot I2 (pixels) in M1, M2, and M3 as a function of time.

**Mouse M1**		**Mouse M2**		**Mouse M3**	
**Time (d)**	**Area (*S*)**	**Time (d)**	**Area (*S*)**	**Time (d)**	**Area (*S*)**
0.031747685	41921	0.017430556	39168	0.013449074	39713
0.034502315	41460	0.019305556	39240	0.015416667	41306
0.241689815	38460	0.268726852	39930	0.25130787	48923
0.243599537	38844	0.270798611	40182	0.253483796	48601
1.072581019	50756	1.030219907	52168	0.998275463	53799
1.074884259	51107	1.032523148	52261	1.000578704	52557
2.034224537	53665	1.991863426	53533	1.959918981	52622
2.036574074	53857	1.994212963	54569	1.962268519	54149
3.025543981	52511	2.98318287	53306	2.951238426	54415
3.02806713	52333	2.985706019	53946	2.953761574	54722
7.054305556	53443	7.011944444	54968	6.98	54957
7.056574074	52642	7.014212963	53812	6.982268519	55753
15.04621528	49337	15.00385417	49187	14.97190972	50977
15.04922454	50174	15.00686343	50593	14.97491898	50615
29.18171296	41253	29.13935185	44526	29.10740741	46092
29.184375	42298	29.14201389	44777	29.11006944	46196
56.17017361	36610	56.1278125	35599	56.09586806	37824
56.17436343	35595	56.13200231	37814	56.10005787	38821
70.34950231	30726	70.3071412	32397	70.27519676	29301
70.35253472	32872	70.31017361	33129	70.27822917	30388
86.21047454	28280	86.16811343	29518	86.13616898	27880
86.21262731	29215	86.1702662	30305	86.13832176	27313
122.1678819	25798	122.1255208	23019	122.0935764	24387
122.1702546	26085	122.1278935	24850	122.0959491	25206
150.1361458	30915	150.0937847	28497	150.0618403	25720
150.1383565	31940	150.0959954	28336	150.0640509	28681
184.122338	22633	184.0799769	21341	184.0480324	18499
184.1271875	24037	184.0848264	20787	184.0528819	18906
247.1662153	21989	**Mouse euthanized on day 202**		247.0919097	19748
247.1682523	22131			247.0939468	18393

Table S38. Area of polymer depot **E1** (pixels) in M1, M2, and M3 as a function of time.

Mouse M1		Mouse M2		Mouse M3	
**Time (d)**	**Area (*S*)**	**Time (d)**	**Area (*S*)**	**Time (d)**	**Area (*S*)**
0.010578704	53881	0.014953704	46093	0.010578704	53565
0.012777778	54588	0.016851852	47769	0.012777778	55083
0.224386574	59254	0.226712963	57821	0.224386574	59167
0.226481481	59474	0.229027778	57696	0.226481481	58497
1.034456019	60000	1.02056713	60000	1.034456019	60000
1.036493056	60000	1.022604167	60000	1.036493056	59999
2.0315625	60000	2.017673611	60000	2.0315625	60000
2.034050926	60000	2.020162037	60000	2.034050926	60000
2.909398148	60000	2.895509259	60000	2.909398148	60000
2.911377315	60000	2.897488426	60000	2.911377315	60000
7.092604167	60000	7.078715278	60000	7.092604167	59999
7.094976852	60000	7.081087963	60000	7.094976852	59999
14.88707176	59058	14.87318287	59181	14.88707176	58438
14.88952546	59203	14.87563657	59534	14.88952546	58820
28.83755787	48938	28.82366898	47425	28.83755787	45465
28.84013889	48988	28.82625	47691	28.84013889	45269
56.96059028	39051	56.94670139	35274	56.96059028	34686
56.96258102	38941	56.94869213	36339	56.96258102	35432
70.22501157	31814	70.21112269	25781	70.22501157	25997
70.2371412	31488	70.22325231	26134	70.2371412	25837
85.99033565	27271	85.97644676	25079	85.99033565	24092
85.99273148	28183	85.97884259	25338	85.99273148	25417
122.9238657	21318	122.9099769	18255	122.9238657	19035
122.9260069	21409	122.9121181	18657	122.9260069	19032
149.8670949	23193	149.853206	19649	149.8670949	19581
149.8699306	23334	149.8560417	19743	149.8699306	19741
182.9947801	17537	182.9808912	14231	182.9947801	12223
182.9980903	17786	182.9842014	13995	182.9980903	12849
**Mouse euthanized on day 202**		247.9501	14264	**Mouse euthanized on day 202**	
		247.95214	13749		

Table S39. Area of polymer depot **E2** (pixels) in M1, M2, and M3 as a function of time.

**Mouse M1**		**Mouse M2**		**Mouse M3**	
**Time (d)**	**Area (*S*)**	**Time (d)**	**Area (*S*)**	**Time (d)**	**Area (*S*)**
0.013229167	52475	0.0234375	56711	0.034965278	59871
0.015289352	53038	0.025266204	56832	0.035914352	59933
0.232534722	59036	0.255555556	59984	0.244444444	60000
0.236168981	59115	0.25625	59965	0.245138889	59999
1.077766204	59990	1.065266204	59998	1.054155093	60000
1.080115741	59998	1.067615741	60000	1.05650463	60000
2.060162037	59996	2.047662037	59999	2.036550926	59999
2.062291667	59993	2.049791667	59998	2.038680556	60000
3.050717593	59992	3.038217593	59996	3.027106481	59999
3.053425926	59989	3.040925926	59994	3.029814815	60000
7.078657407	59986	7.066157407	59991	7.055046296	59996
7.081180556	59993	7.068680556	59993	7.057569444	59999
15.1159375	59910	15.1034375	59912	15.09232639	59932
15.11893519	59854	15.10643519	59838	15.09532407	59940
29.1940162	50270	29.1815162	48011	29.17040509	50930
29.19615741	50616	29.18365741	50115	29.1725463	51977
56.19212963	40768	56.17962963	39979	56.16851852	45182
56.19475694	41147	56.18225694	40604	56.17114583	45389
70.36564815	29611	70.35314815	29901	70.34203704	36120
70.36997685	29745	70.35747685	31836	70.34636574	36776
86.18175926	27875	86.16925926	28094	86.15814815	33307
86.18390046	28365	86.17140046	28633	86.16028935	33787
122.1954282	18791	122.1829282	17127	122.1718171	26173
122.1973843	19201	122.1848843	18149	122.1737731	26918
150.1613657	24459	150.1488657	22210	150.1377546	29986
150.1636574	24274	150.1511574	22770	150.1400463	28006
184.1498843	15229	184.1373843	15768	184.1262731	18713
184.1524421	15842	184.1399421	15332	184.128831	20192
**Mouse euthanized on day 202**		247.181713	16068	247.1706019	19604
		247.1842477	16699	247.1731366	19696

Table S40. Area of polymer depot **P1** (pixels) in M1, M2, and M3 as a function of time.

Mouse M1		Mouse M2		Mouse M3	
**Time (d)**	**Area (*S*)**	**Time (d)**	**Area (*S*)**	**Time (d)**	**Area (*S*)**
0.011805556	58487	0.028703704	59831	0.054027778	59965
0.01400463	58300	0.029409722	59823	0.054965278	59973
0.235023148	59879	0.218668981	59997	0.213414352	59988
0.236944444	59944	0.220844907	60000	0.215532407	59984
1.057268519	60000	1.007962963	60000	0.98712963	60000
1.059409722	60000	1.010104167	60000	0.989270833	60000
1.933009259	60000	1.883703704	60000	1.86287037	60000
1.935358796	60000	1.886053241	60000	1.865219907	60000
2.873622685	60000	2.82431713	60000	2.803483796	60000
2.875810185	60000	2.82650463	60000	2.805671296	60000
6.906076389	60000	6.856770833	60000	6.8359375	60000
6.908587963	60000	6.859282407	60000	6.838449074	60000
14.91810185	59753	14.8687963	59918	14.84796296	59927
14.92077546	59848	14.87146991	59960	14.85063657	59903
28.94070602	56065	28.89140046	56394	28.87056713	57539
28.94372685	56792	28.8944213	57677	28.87358796	57389
56.02479167	41898	55.97548611	40990	55.95465278	43137
56.02895833	41946	55.97965278	42083	55.95881944	43931
70.20241898	34881	70.15311343	35250	70.13228009	36917
70.20532407	34498	70.15601852	34338	70.13518519	36670
87.04762731	33470	86.99832176	32611	86.97748843	35739
87.05053241	34345	87.00122685	33917	86.98039352	35624
122.0149884	19915	121.9656829	22948	121.9448495	24931
122.0171181	20691	121.9678125	22948	121.9469792	26426
149.9853588	24691	149.9360532	23337	149.9152199	22835
149.9875231	23549	149.9382176	23518	149.9173843	23591
183.9723264	14496	183.9230208	13156	183.9021875	14532
183.9745139	14103	183.9252083	12519	183.904375	13991
**Mouse euthanized on day 202**		246.96334	13582	246.9425116	13806
		246.96572	13338	246.9448843	13125

Table S41. Area of polymer depot **P2** (pixels) in M1, M2, and M3 as a function of time.

**Mouse M1**		**Mouse M2**		**Mouse M3**	
**Time (d)**	**Area (*S*)**	**Time (d)**	**Area (*S*)**	**Time (d)**	**Area (*S*)**
0.013541667	59414	0.025300926	59001	0.015717593	59545
0.015277778	59155	0.028946759	59575	0.017708333	59492
0.228171296	59813	0.220983796	59994	0.218935185	59992
0.230138889	59849	0.222905093	60000	0.225069444	59994
0.980740741	60000	1.023333333	60000	1.021909722	60000
0.983032407	60000	1.025451389	60000	1.024351852	60000
1.994756944	60000	2.03162037	60000	2.001064815	60000
1.997118056	60000	2.034016204	60000	2.003460648	60000
2.911747685	60000	3.030578704	60000	3.000023148	60000
2.913831019	60000	3.032696759	60000	3.002141204	60000
7.091759259	60000	7.209097222	60000	7.178541667	60000
7.096597222	60000	7.211400463	59980	7.180844907	60000
14.88825231	59864	14.99456019	59977	14.96400463	59820
14.89138889	59844	14.99645833	53455	14.96590278	59866
28.83503472	52094	28.92715278	53801	28.89659722	50206
28.83890046	52051	28.9296412	42993	28.89908565	51356
56.92354167	44059	57.0305787	42826	57.00002315	38734
56.92631944	42791	57.03320602	37635	57.00265046	38512
70.22130787	37075	70.18325231	38061	70.15269676	34515
70.22390046	38105	70.1865625	34983	70.15600694	35352
85.91983796	34665	86.01891204	35391	85.98835648	29056
85.92288194	33938	86.02116898	28025	85.99061343	30118
122.9235301	22872	123.0411343	28233	123.0105787	20596
122.9256481	24174	123.0437616	26577	123.013206	19005
149.864919	23849	149.9797106	26092	149.9491551	19072
149.8678009	23707	149.9831019	17548	149.9525463	18146
182.9984028	15203	183.1138542	18115	183.0832986	11884
183.000706	15087	183.1160995	16092	183.085544	11425
247.9684375	16727	**Mouse euthanized on day 202**		247.1143866	14367
247.970463	16988			247.1165856	12749

Table S42. Area of polymer depot **Cy7-amine** (pixels) in M1, M2, and M3 as a function of time.

**Mouse M1**		**Mouse M2**		**Mouse M3**	
**Time (d)**	**Area (*S*)**	**Time (d)**	**Area (*S*)**	**Time (d)**	**Area (*S*)**
0.020185185	49046	0.020960648	59564	0.016712963	34924
0.022141204	49728	0.023159722	59560	0.018668981	35314
0.229814815	59891	0.227604167	59846	0.226342593	38527
0.231770833	59934	0.229560185	59745	0.228298611	37988
1.044039352	60000	1.016956019	59997	1.04056713	59832
1.046574074	60000	1.019490741	59996	1.043101852	59839
2.042719907	59993	2.015636574	59998	2.039247685	59581
2.043680556	59993	2.016597222	59993	2.040208333	59261
5.341053241	59988	5.313969907	59987	5.337581019	58035
5.343483796	59991	5.316400463	59998	5.340011574	57266
6.082465278	59986	6.055381944	59997	6.078993056	57394
6.085023148	59988	6.057939815	59992	6.081550926	57355
15.16583333	45550	15.13875	44704	15.16236111	27430
15.16923611	45841	15.14215278	43737	15.16576389	26241
29.27958333	28226	29.2525	25411	29.27611111	8349
29.28241898	29518	29.25533565	25213	29.27894676	8818
57.31351852	16755	57.28643519	15170	57.3100463	4097
57.31608796	16682	57.28900463	15195	57.31261574	3860
69.45393519	12151	69.42685185	10702	69.45046296	3176
69.45649306	11770	69.42940972	10439	69.45302083	2936
85.18834491	11471	85.16126157	10530	85.18487269	3863
85.19056713	11396	85.1634838	10438	85.18709491	3182
121.0998843	8749	121.0728009	7325	121.096412	4069
121.1071065	8806	121.0800231	7957	121.1036343	4247
149.2655208	10536	149.2384375	9758	149.2620486	6282
183.2483102	7817	183.2212269	5834	183.244838	4723
183.2510417	7791	183.2239583	6017	183.2475694	3812
246.2715972	9833	246.2445139	7111	**Mouse euthanized on day 202**	
246.2734259	10399	246.2463426	7303		

S12.1.3. Intramuscular depot dissolution data – depot volume (V_{IM})

Table S43. Volume and signal of intramuscular depot of **F1** in M1, M2, M3 and mean values as a function of time, determined by photoacoustics.

Time past administr.	Mouse M1		Mouse M2		Mouse M3		Mean ± SD	
	Volume (V, mm^3)	Signal (I, a.u.)	Volume (V, mm^3)	Signal (I, a.u.)	Volume (V, mm^3)	Signal (I, a.u.)	Volume (V, mm^3)	Signal (I, a.u.)
**before**	0.000	0.030	0.000	0.036	0.000	0.052	0 ± 0	0.039 ± 0.009
**5 min**	23.489	0.728	50.019	1.010	144.053	2.534	73 ± 52	1.424 ± 0.793
**7 days**	57.353	1.237	94.538	1.160	94.282	1.627	82 ± 17	1.341 ± 0.204
**14 days**	56.218	1.423	71.128	1.297	77.258	1.767	68 ± 9	1.496 ± 0.199
**30 days**	18.111	1.162	37.326	1.186	35.745	1.437	30 ± 9	1.262 ± 0.124
**60 days**	34.773	1.832	71.519	1.563	42.080	1.074	49 ± 16	1.490 ± 0.314
**90 days**	36.793	1.401	55.147	1.057	38.310	0.830	43 ± 8	1.096 ± 0.235
**120 days**	86.649	0.663	121.984	0.685	51.749	0.327	87 ± 29	0.558 ± 0.164
**150 days**	50.461	0.843	56.761	0.751	13.789	0.400	40 ± 19	0.665 ± 0.191
**180 days**	67.906	0.590	95.079	0.550	8.407	0.273	57 ± 36	0.471 ± 0.141
**240 days**	53.686	0.623	**euthanized**		1.075	0.222	27 ± 26	0.423 ± 0.201

Table S44. Volume and signal of intramuscular depot of **F2** in M1, M2, M3 and mean values as a function of time, determined by photoacoustics.

Time past administr.	Mouse M1		Mouse M2		Mouse M3		Mean ± SD	
	Volume (V, mm^3)	Signal (I, a.u.)	Volume (V, mm^3)	Signal (I, a.u.)	Volume (V, mm^3)	Signal (I, a.u.)	Volume (V, mm^3)	Signal (I, a.u.)
**before**	0.000	0.027	0.000	0.030	0.000	0.047	0 ± 0	0.035 ± 0.009
**5 min**	52.991	1.022	31.697	1.090	77.428	1.489	54 ± 19	1.200 ± 0.206
**7 days**	14.691	0.428	0.000	0.380	0.000	0.485	5 ± 7	0.431 ± 0.043
**14 days**	0.000	0.234	0.000	0.255	0.000	0.354	0 ± 0	0.281 ± 0.052
**30 days**	0.000	0.309	0.000	0.224	0.000	0.160	0 ± 0	0.231 ± 0.061
**60 days**	0.000	0.118	0.000	0.096	0.000	0.140	0 ± 0	0.118 ± 0.018
**90 days**	0.000	0.097	0.000	0.119	0.000	0.138	0 ± 0	0.118 ± 0.017
**120 days**	0.000	0.058	0.000	0.093	0.000	0.047	0 ± 0	0.066 ± 0.020
**150 days**	0.000	0.043	0.000	0.072	0.000	0.040	0 ± 0	0.052 ± 0.014
**180 days**	0.000	0.030	0.000	0.033	0.000	0.030	0 ± 0	0.031 ± 0.001
**240 days**	0.000	0.028	**euthanized**		0.000	0.025	0 ± 0	0.027 ± 0.002

Table S45. Volume and signal of intramuscular depot of **I1** in M1, M2, M3 and mean values as a function of time, determined by photoacoustics.

Time past administr.	Mouse M1		Mouse M2		Mouse M3		Mean ± SD	
	Volume (V, mm^3)	Signal (I, a.u.)	Volume (V, mm^3)	Signal (I, a.u.)	Volume (V, mm^3)	Signal (I, a.u.)	Volume (V, mm^3)	Signal (I, a.u.)
**before**	0.000	0.000	0.000	0.000	0.000	0.029	0 ± 0	0.010 ± 0.014
**5 min**	28.448	0.508	100.298	0.985	76.112	1.206	68 ± 30	0.900 ± 0.291
**7 days**	71.794	1.200	63.395	0.680	54.026	1.824	63 ± 7	1.235 ± 0.468
**14 days**	127.613	0.540	75.855	0.430	166.285	0.610	123 ± 37	0.527 ± 0.074
**30 days**	19.106	0.703	21.936	0.594	35.301	1.091	25 ± 7	0.796 ± 0.213
**60 days**	14.633	0.849	24.131	0.815	33.618	0.820	24 ± 8	0.828 ± 0.015
**90 days**	12.172	0.792	21.375	0.751	34.224	0.819	23 ± 9	0.787 ± 0.028
**120 days**	6.928	0.026	33.628	0.420	42.287	0.299	28 ± 15	0.248 ± 0.165
**150 days**	4.543	0.349	5.452	0.258	14.090	0.529	8 ± 4	0.379 ± 0.113
**180 days**	3.306	0.150	8.614	0.270	9.493	0.270	7 ± 3	0.230 ± 0.057
**240 days**	**euthanized**		1.268	0.175	4.629	0.161	3 ± 2	0.168 ± 0.007

Table S46. Volume and signal of intramuscular depot of **I2** in M1, M2, M3 and mean values as a function of time, determined by photoacoustics.

Time past administr.	Mouse M1 Volume (V, mm^3)	Mouse M1 Signal (I, a.u.)	Mouse M2 Volume (V, mm^3)	Mouse M2 Signal (I, a.u.)	Mouse M3 Volume (V, mm^3)	Mouse M3 Signal (I, a.u.)	Mean ± SD Volume (V, mm^3)	Mean ± SD Signal (I, a.u.)
**before**	0.000	0.043	0.000	0.050	0.000	0.051	0 ± 0	0.048 ± 0.004
**5 min**	140.892	1.446	83.393	1.470	148.729	1.150	124 ± 29	1.355 ± 0.146
**7 days**	58.271	1.032	71.864	1.578	101.323	1.498	77 ± 18	1.369 ± 0.241
**14 days**	58.650	1.678	76.839	1.196	64.912	1.832	67 ± 8	1.569 ± 0.271
**30 days**	24.314	0.937	31.393	1.597	47.539	0.946	34 ± 10	1.16 ± 0.309
**60 days**	70.652	0.872	45.321	0.947	80.598	0.704	66 ± 15	0.841 ± 0.102
**90 days**	56.743	0.987	63.299	0.849	56.094	0.674	59 ± 3	0.837 ± 0.128
**120 days**	78.572	0.748	62.400	0.451	23.328	0.541	55 ± 23	0.58 ± 0.124
**150 days**	43.811	0.663	31.576	0.519	14.445	0.680	30 ± 12	0.621 ± 0.072
**180 days**	55.393	0.471	29.370	0.311	14.322	0.307	33 ± 17	0.363 ± 0.076
**240 days**	33.354	0.435	**euthanized**		12.374	0.415	23 ± 10	0.425 ± 0.01

Table S47. Volume and signal of intramuscular depot of **E1** in M1, M2, M3 and mean values as a function of time, determined by photoacoustics.

Time past administr.	Mouse M1 Volume (V, mm^3)	Mouse M1 Signal (I, a.u.)	Mouse M2 Volume (V, mm^3)	Mouse M2 Signal (I, a.u.)	Mouse M3 Volume (V, mm^3)	Mouse M3 Signal (I, a.u.)	Mean ± SD Volume (V, mm^3)	Mean ± SD Signal (I, a.u.)
**before**	0.000	0.000	0.000	0.046	0.000	0.037	0 ± 0	0.028 ± 0.02
**5 min**	113.581	2.329	125.675	0.675	105.423	1.263	115 ± 8	1.422 ± 0.685
**7 days**	10.406	0.807	9.800	0.370	11.738	0.592	11 ± 1	0.59 ± 0.178
**14 days**	6.887	0.410	28.874	0.480	7.411	0.200	14 ± 10	0.363 ± 0.119
**30 days**	0.000	0.026	16.639	0.249	0.000	0.054	6 ± 8	0.11 ± 0.099
**60 days**	0.000	0.024	0.000	0.250	0.000	0.028	0 ± 0	0.101 ± 0.106
**90 days**	0.000	0.014	0.000	0.028	0.000	0.020	0 ± 0	0.021 ± 0.006
**120 days**	0.000	0.020	0.000	0.051	0.000	0.030	0 ± 0	0.034 ± 0.013
**150 days**	0.000	0.023	0.000	0.019	0.000	0.019	0 ± 0	0.02 ± 0.002
**180 days**	0.000	0.024	0.000	0.021	0.000	0.021	0 ± 0	0.022 ± 0.001
**240 days**	**euthanized**		0.000	0.022	**euthanized**		0 ± 0	0.022 ± 0

Table S48. Volume and signal of intramuscular depot of **E2** in M1, M2, M3 and mean values as a function of time, determined by photoacoustics.

Time past administr.	Mouse M1 Volume (V, mm^3)	Mouse M1 Signal (I, a.u.)	Mouse M2 Volume (V, mm^3)	Mouse M2 Signal (I, a.u.)	Mouse M3 Volume (V, mm^3)	Mouse M3 Signal (I, a.u.)	Mean ± SD Volume (V, mm^3)	Mean ± SD Signal (I, a.u.)
**before**	0.000	0.074	0.000	0.030	0.000	0.000	0 ± 0	0.035 ± 0.03
**5 min**	148.245	2.095	54.849	2.696	66.660	1.805	90 ± 42	2.199 ± 0.371
**7 days**	20.024	0.580	0.000	0.074	19.782	0.659	13 ± 9	0.438 ± 0.259
**14 days**	14.240	0.554	7.251	0.308	6.166	0.414	9 ± 4	0.425 ± 0.101
**30 days**	0.000	0.014	0.000	0.037	0.000	0.024	0 ± 0	0.025 ± 0.009
**60 days**	0.000	0.030	0.000	0.032	0.000	0.026	0 ± 0	0.029 ± 0.002
**90 days**	0.000	0.033	0.000	0.022	0.000	0.015	0 ± 0	0.023 ± 0.007
**120 days**	0.000	0.021	0.000	0.022	0.000	0.027	0 ± 0	0.023 ± 0.003
**150 days**	0.000	0.023	0.000	0.019	0.000	0.030	0 ± 0	0.024 ± 0.005
**180 days**	0.000	0.021	0.000	0.017	0.000	0.020	0 ± 0	0.019 ± 0.002
**240 days**	**euthanized**		0.000	0.023	0.000	0.025	0 ± 0	0.024 ± 0.001

Table S49. Volume and signal of intramuscular depot of **P1** in M1, M2, M3 and mean values as a function of time, determined by photoacoustics.

Time past administr.	Mouse M1 Volume (V, mm^3)	Mouse M1 Signal (I, a.u.)	Mouse M2 Volume (V, mm^3)	Mouse M2 Signal (I, a.u.)	Mouse M3 Volume (V, mm^3)	Mouse M3 Signal (I, a.u.)	Mean ± SD Volume (V, mm^3)	Mean ± SD Signal (I, a.u.)
**before**	0.000	0.046	0.000	0.031	0.000	0.040	0 ± 0	0.039 ± 0.006
**5 min**	65.055	0.316	17.677	0.350	44.287	0.305	42 ± 19	0.324 ± 0.019
**7 days**	13.130	0.467	0.000	0.040	35.071	0.339	16 ± 14	0.282 ± 0.179
**14 days**	6.482	0.493	0.000	0.064	0.000	0.067	2 ± 3	0.208 ± 0.202
**30 days**	0.000	0.021	0.000	0.028	0.000	0.015	0 ± 0	0.021 ± 0.005
**60 days**	0.000	0.030	0.000	0.026	0.000	0.027	0 ± 0	0.028 ± 0.002
**90 days**	0.000	0.026	0.000	0.024	0.000	0.020	0 ± 0	0.023 ± 0.002
**120 days**	0.000	0.024	0.000	0.037	0.000	0.027	0 ± 0	0.029 ± 0.006
**150 days**	0.000	0.022	0.000	0.024	0.000	0.026	0 ± 0	0.024 ± 0.002
**180 days**	0.000	0.017	0.000	0.016	0.000	0.034	0 ± 0	0.022 ± 0.008
**240 days**	**euthanized**		0.000	0.024	0.000	0.028	0 ± 0	0.026 ± 0.002

Table S50. Volume and signal of intramuscular depot of **P2** in M1, M2, M3 and mean values as a function of time, determined by photoacoustics.

Time past administr.	Mouse M1 Volume (V, mm^3)	Mouse M1 Signal (I, a.u.)	Mouse M2 Volume (V, mm^3)	Mouse M2 Signal (I, a.u.)	Mouse M3 Volume (V, mm^3)	Mouse M3 Signal (I, a.u.)	Mean ± SD Volume (V, mm^3)	Mean ± SD Signal (I, a.u.)
**before**	0.000	0.050	0.000	0.053	0.000	0.044	0 ± 0	0.049 ± 0.004
**5 min**	19.199	0.210	87.629	0.835	44.159	0.414	50 ± 28	0.486 ± 0.26
**7 days**	0.000	0.054	0.000	0.050	0.000	0.066	0 ± 0	0.057 ± 0.007
**14 days**	0.000	0.115	0.000	0.045	0.000	0.049	0 ± 0	0.07 ± 0.032
**30 days**	0.000	0.028	0.000	0.028	0.000	0.019	0 ± 0	0.025 ± 0.004
**60 days**	0.000	0.024	0.000	0.031	0.000	0.020	0 ± 0	0.025 ± 0.005
**90 days**	0.000	0.011	0.000	0.016	0.000	0.024	0 ± 0	0.017 ± 0.005
**120 days**	0.000	0.023	0.000	0.022	0.000	0.027	0 ± 0	0.024 ± 0.002
**150 days**	0.000	0.023	0.000	0.045	0.000	0.021	0 ± 0	0.03 ± 0.011
**180 days**	0.000	0.023	0.000	0.020	0.000	0.022	0 ± 0	0.022 ± 0.001
**240 days**	0.000	0.022	0.000	0.025	0.000	0.033	0 ± 0	0.027 ± 0.005

Table S51. Volume and signal of intramuscular depot of **Cy7-amine** in M1, M2, M3 and mean values as a function of time, determined by photoacoustics.

Time past administr.	Mouse M1 Volume (V, mm^3)	Mouse M1 Signal (I, a.u.)	Mouse M2 Volume (V, mm^3)	Mouse M2 Signal (I, a.u.)	Mouse M3 Volume (V, mm^3)	Mouse M3 Signal (I, a.u.)	Mean ± SD Volume (V, mm^3)	Mean ± SD Signal (I, a.u.)
**before**	0.000	0.040	0.000	0.068	0.000	0.058	0 ± 0	0.055 ± 0.012
**5 min**	55.489	0.179	137.507	0.809	136.829	0.406	110 ± 39	0.465 ± 0.261
**7 days**	0.000	0.061	0.000	0.049	0.000	0.042	0 ± 0	0.051 ± 0.008
**14 days**	0.000	0.025	0.000	0.045	0.000	0.033	0 ± 0	0.034 ± 0.008
**30 days**	0.000	0.014	0.000	0.020	0.000	0.040	0 ± 0	0.025 ± 0.011
**60 days**	0.000	0.030	0.000	0.049	0.000	0.024	0 ± 0	0.034 ± 0.011
**90 days**	0.000	0.036	0.000	0.030	0.000	0.033	0 ± 0	0.033 ± 0.002
**120 days**	N/A	N/A	N/A	N/A	N/A	N/A	N/A	N/A
**150 days**	0.000	0.022	0.000	0.026	0.000	0.018	0 ± 0	0.022 ± 0.003
**180 days**	0.000	0.021	0.000	0.017	0.000	0.021	0 ± 0	0.02 ± 0.002
**240 days**	0.000	0.024	0.000	0.030	**euthanized**		0 ± 0	0.027 ± 0.003

Table S52. Volume and signal of intramuscular depot of **DMSO mice** in M1, M2, M3, M4, M5 and M6 and mean values as a function of time, determined by photoacoustics.

**Time past administr.**	**Mouse M1**		**Mouse M2**		**Mouse M3**		**Mouse M4**		**Mouse M5**		**Mouse M6**		**Mean ± SD**	
	**Volume (V, mm^3)**	**Signal (I, a.u.)**	**Volume (V, mm^3)**	**Signal (I, a.u.)**	**Volume (V, mm^3)**	**Signal (I, a.u.)**	**Volume (V, mm^3)**	**Signal (I, a.u.)**	**Volume (V, mm^3)**	**Signal (I, a.u.)**	**Volume (V, mm^3)**	**Signal (I, a.u.)**	**Volume (V, mm^3)**	**Signal (I, a.u.)**
**before**	0.000	0.000	0.000	0.000	0.000	0.000	0.000	0.045	0.000	0.035	0.000	0.099	0 ± 0	0.03 ± 0.036
**5 min**	0.000	0.055	0.000	0.042	0.000	0.036	0.000	0.037	0.000	0.036	0.000	0.070	0 ± 0	0.046 ± 0.013
**7 days**	0.000	0.064	0.000	0.033	0.000	0.064	0.000	-	0.000	-	0.000	-	0 ± 0	0.054 ± 0.015
**14 days**	0.000	0.047	0.000	0.027	0.000	0.070	0.000	-	0.000	-	0.000	-	0 ± 0	0.048 ± 0.018
**30 days**	0.000	0.030	0.000	0.043	0.000	0.026	0.000	-	0.000	-	0.000	-	0 ± 0	0.033 ± 0.007
**60 days**	0.000	0.026	0.000	0.026	0.000	0.023	0.000	-	0.000	-	0.000	-	0 ± 0	0.025 ± 0.001
**90 days**	N/A	N/A	N/A	N/A	N/A	N/A	N/A	N/A	N/A	N/A	N/A	N/A	0 ± 0	N/A
**120 days**	0.000	0.016	0.000	0.018	0.000	0.019	0.000	-	0.000	-	0.000	-	0 ± 0	0.018 ± 0.001
**150 days**	0.000	0.032	0.000	0.024	0.000	0.039	0.000	-	0.000	-	0.000	-	0 ± 0	0.032 ± 0.006
**180 days**	0.000	0.034	0.000	0.016	0.000	0.020	0.000	-	0.000	-	0.000	-	0 ± 0	0.023 ± 0.008
**240 days**	N/A	N/A	N/A	N/A	N/A	N/A	N/A	N/A	N/A	N/A	N/A	N/A	0 ± 0	N/A

S12.1.4. Intramuscular depot dissolution data – depot distribution index (K_{10})

Table S53. K_{10} of intramuscular depot **F1** in M1, M2, and M3 as a function of time.

Mouse M1		Mouse M2		Mouse M3	
**Time (d)**	**K_{10} (%)**	**Time (d)**	**K_{10} (%)**	**Time (d)**	**K_{10} (%)**
0.01244213	26.8726778	0.018391204	41.18494511	0.014189815	39.20550823
0.01443287	28.35560083	0.020810185	43.89081478	0.016458333	42.8205061
0.244375	37.68959522	0.231111111	43.43250275	0.22849537	45.92130184
0.246261574	38.30986023	0.232951389	43.27404022	0.230335648	47.07471848
1.03962963	37.14161158	1.002824074	41.64226055	0.98337963	43.77813816
1.042141204	37.10666418	1.005335648	41.65099144	0.985891204	44.51037884
1.991134259	37.24989176	1.954328704	41.04254723	1.934884259	41.44934177
1.993634259	36.97295427	1.956828704	40.37489414	1.937384259	41.1259985
2.9325	36.85702562	2.895694444	41.03655338	2.87625	40.647645
2.935949074	36.85572863	2.899143519	40.05917549	2.879699074	40.69068909
6.96431713	37.26462603	6.927511574	41.36669159	6.90806713	40.82199574
6.966770833	37.27998495	6.929965278	41.17435455	6.910520833	40.95180988
14.96072917	30.35702229	14.92392361	35.59995651	14.90447917	38.16788435
14.96314815	30.54667473	14.92634259	35.5592823	14.90689815	37.96817541
28.99543981	29.62453842	28.95863426	37.34452248	28.93918981	41.28763199
28.99820602	29.5769763	28.96140046	37.05945969	28.94195602	41.13304138
56.11059028	28.1144166	56.07378472	39.43067074	56.05434028	37.99412012
56.11278935	28.13137293	56.0759838	39.79222536	56.05653935	38.67030144
70.25825231	23.59794617	70.22144676	34.61190224	70.20200231	33.16933155
70.26082176	24.60082769	70.2240162	35.1966691	70.20457176	32.96123266
86.11630787	24.77472782	86.07950231	36.93135977	86.06005787	33.75956774
86.11840278	25.46661854	86.08159722	37.33627081	86.06215278	33.81848335
122.0740278	20.75895548	122.0372222	32.46591091	122.0177778	28.84082794
122.0763773	21.86129332	122.0395718	32.53840446	122.0201273	29.0325141
150.0428356	24.79904413	150.0060301	36.23910904	149.9865856	32.53377438
150.0453472	25.80955267	150.0085417	36.62391424	149.9890972	32.65138626
184.0263426	20.1850462	183.989537	30.40871859	183.9700926	22.88978577
184.0284954	21.10004425	183.9916898	31.43069744	183.9722454	22.99913406
247.0727546	20.57924509	**Mouse euthanized on day 202**		247.017535	21.49395227
247.0747454	20.43531179			247.019525	21.59399033

Table S54. K_{10} of intramuscular depot **F2** in M1, M2, and M3 as a function of time.

Mouse M1		Mouse M2		Mouse M3	
**Time (d)**	**K_{10} (%)**	**Time (d)**	**K_{10} (%)**	**Time (d)**	**K_{10} (%)**
0.009328704	61.20753765	0.015069444	63.69200706	0.019097222	78.56770039
0.011180556	64.38688278	0.016851852	65.03332615	0.021354167	80.52357674
0.223391204	52.87550449	0.222488426	49.64683056	0.225324074	57.7944231
0.225486111	53.42493057	0.224259259	48.56740952	0.227835648	58.55394363
1.046111111	40.69138527	1.032222222	36.18466139	1.011388889	40.74400425
1.048275463	40.33146381	1.034386574	36.76021814	1.013553241	39.48740005
2.044594907	40.79825401	2.030706019	36.28196478	2.009872685	42.58202076
2.049201389	40.72062969	2.0353125	36.11035109	2.014479167	40.9963274
2.975231481	42.51257896	2.961342593	37.86313772	2.940509259	40.93015671
2.977430556	41.26957417	2.963541667	38.06865931	2.942708333	41.05137348
7.158125	41.28688812	7.144236111	39.08530712	7.123402778	40.78309059
7.160231481	41.26701832	7.146342593	38.88101816	7.125509259	41.22936249
14.94936343	34.86147642	14.93547454	31.95285082	14.9146412	34.32411432
14.95398148	35.48719406	14.94009259	32.7845645	14.91925926	35.14904022
28.87645833	33.74176741	28.86256944	29.02635098	28.84173611	32.13870525
28.87976852	33.29665422	28.86587963	29.49113846	28.8450463	32.99599171
56.97891204	34.81773615	56.96502315	29.57134008	56.94418981	33.85895491
56.98115741	34.91708279	56.96726852	30.0477767	56.94643519	31.96609735
70.17987269	35.75553179	70.1659838	28.35220575	70.14515046	30.51304817
70.18262731	35.44240713	70.16873843	28.36313963	70.14790509	31.20911121
85.96809028	35.1436305	85.95420139	30.55122614	85.93336806	33.32297325
85.97021991	35.26675701	85.95633102	30.3960824	85.93549769	32.53214598
122.9824884	34.7649169	122.9685995	29.24993515	122.9477662	34.73234892
122.9846412	35.50416231	122.9707523	29.11916494	122.949919	35.81006765
149.9269676	36.23334885	149.9130787	28.96141291	149.8922454	33.74037743
149.9317245	36.37972116	149.9178356	29.27288294	149.8970023	34.76267576
183.0573495	32.29159355	183.0434606	27.70041943	183.0226273	33.26639891
183.0620139	33.06677341	183.048125	28.38421106	183.0272917	33.15604687
247.0895602	32.83375978	**Mouse euthanized on day 202**		247.054838	35.29402494
247.0959144	32.75918245			247.0611921	35.73839664

Table S55. K_{10} of intramuscular depot **I1** in M1, M2, and M3 as a function of time.

**Mouse M1**		**Mouse M2**		**Mouse M3**	
**Time (d)**	**K_{10} (%)**	**Time (d)**	**K_{10} (%)**	**Time (d)**	**K_{10} (%)**
0.025300926	53.52656841	0.015717593	45.38583755	0.011967593	58.79834652
0.028946759	55.58012486	0.017708333	47.37661839	0.014131944	61.48067951
0.220983796	65.19915104	0.218935185	50.87183952	0.221793981	70.26882172
0.222905093	65.2680254	0.225069444	50.97001076	0.223634259	70.76332092
1.023333333	62.57708549	1.021909722	48.49153042	1.029490741	62.2584343
1.025451389	61.44411564	1.024351852	47.32029915	1.031886574	63.10013294
2.03162037	68.06720257	2.001064815	54.04950619	1.980231481	65.09875774
2.034016204	65.66627979	2.003460648	53.71743202	1.982627315	64.99735355
3.030578704	67.31894493	3.000023148	56.38544083	2.979189815	66.61964893
3.032696759	67.39109039	3.002141204	55.84373474	2.98130787	66.43420696
7.209097222	70.88998318	7.178541667	57.07721233	7.157708333	69.40911293
7.211400463	69.98738766	7.180844907	56.9717741	7.160011574	68.16349506
14.99456019	67.10698605	14.96400463	51.95254803	14.9431713	71.53903008
14.99645833	66.77252769	14.96590278	52.77850628	14.94506944	71.89601898
28.92715278	67.0558548	28.89659722	54.83841896	28.87576389	71.48068905
28.9296412	67.20385075	28.89908565	54.89408016	28.87825231	71.80417061
57.0305787	53.66932869	57.00002315	45.31137466	56.97918981	62.99276829
57.03320602	53.13952446	57.00265046	45.71066856	56.98181713	62.9919529
70.18325231	44.13968563	70.15269676	36.39799356	70.13186343	50.58806896
70.1865625	43.73343945	70.15600694	35.33570051	70.13517361	50.79758167
86.01891204	43.03536415	85.98835648	38.1221652	85.96752315	54.35452938
86.02116898	42.4072361	85.99061343	37.77703047	85.96978009	54.04569626
123.0411343	30.84260941	123.0105787	28.0652833	123.1425463	39.30188179
123.0437616	30.9304595	123.013206	28.121171	123.1445718	37.57745504
149.9797106	35.23328304	149.9491551	33.46003771	149.9283218	44.00274754
149.9831019	35.43140411	149.9525463	34.06987906	149.931713	44.1349268
183.1138542	26.95334435	183.0832986	26.89240217	183.0624653	36.10779285
183.1160995	26.61162138	183.085544	27.39491463	183.0647106	33.03804874
**Mouse euthanized on day 202**		247.1143866	22.47302532	247.0935532	28.38831425
		247.1165856	22.38470554	247.0957523	26.76533699

Table S56. K_{10} of intramuscular depot **I2** in M1, M2, and M3 as a function of time.

**Mouse M1**		**Mouse M2**		**Mouse M3**	
**Time (d)**	**K_{10} (%)**	**Time (d)**	**K_{10} (%)**	**Time (d)**	**K_{10} (%)**
0.031747685	47.27107525	0.017430556	53.22714806	0.013449074	55.35090923
0.034502315	47.19826698	0.019305556	53.82368565	0.015416667	57.64804363
0.241689815	47.4237299	0.268726852	51.38897419	0.25130787	67.47525215
0.243599537	48.68989468	0.270798611	51.77260399	0.253483796	66.5090704
1.072581019	58.10711861	1.030219907	61.47353172	0.998275463	68.04877758
1.074884259	57.28171825	1.032523148	61.83485508	1.000578704	67.31711388
2.034224537	61.3572073	1.991863426	64.43262577	1.959918981	68.1035471
2.036574074	62.37831593	1.994212963	65.53878307	1.962268519	71.07526302
3.025543981	59.40956593	2.98318287	66.08181477	2.951238426	71.05207443
3.02806713	59.23021317	2.985706019	65.9899044	2.953761574	71.75316334
7.054305556	62.99690247	7.011944444	68.00973415	6.98	73.89389515
7.056574074	62.20145226	7.014212963	66.95975304	6.982268519	74.10359383
15.04621528	56.75542355	15.00385417	62.99746037	14.97190972	68.7835741
15.04922454	57.0945549	15.00686343	64.04411793	14.97491898	68.87373447
29.18171296	47.07068443	29.13935185	58.89345646	29.10740741	63.12410831
29.184375	48.01712513	29.14201389	59.39592838	29.11006944	63.35748196
56.17017361	42.70896435	56.1278125	47.69444466	56.09586806	50.73353767
56.17436343	40.93784809	56.13200231	49.83736992	56.10005787	52.20602036
70.34950231	33.60166788	70.3071412	44.21423912	70.27519676	40.3275156
70.35253472	35.24022102	70.31017361	44.74163055	70.27822917	41.74227238
86.21047454	30.58245897	86.16811343	39.52711821	86.13616898	38.56207848
86.21262731	32.02438831	86.1702662	40.3565979	86.13832176	38.00915956
122.1678819	28.73831272	122.1255208	31.39577389	122.0935764	33.08196783
122.1702546	28.48896742	122.1278935	33.34175348	122.0959491	33.80390167
150.1361458	32.5173831	150.0937847	36.90011501	150.0618403	33.92902136
150.1383565	31.38617516	150.0959954	36.59102678	150.0640509	36.81541681
184.122338	24.89826202	184.0799769	28.35809469	184.0480324	25.14397383
184.1271875	25.61661482	184.0848264	27.96979189	184.0528819	25.43708563
247.1662153	21.69829369	**Mouse euthanized on day 202**		247.0919097	25.71174622
247.1682523	21.67046309			247.0939468	24.03290033

Table S57. K_{10} of intramuscular depot E1 in M1, M2, and M3 as a function of time.

Mouse M1		Mouse M2		Mouse M3	
**Time (d)**	**K_{10} (%)**	**Time (d)**	**K_{10} (%)**	**Time (d)**	**K_{10} (%)**
0.010578704	70.16759396	0.014953704	59.90602493	0.010578704	74.20728207
0.012777778	71.69400215	0.016851852	62.45993137	0.012777778	75.41339874
0.224386574	74.86395836	0.226712963	70.74977875	0.224386574	77.04542637
0.226481481	75.11527061	0.229027778	71.30753517	0.226481481	76.73229694
1.034456019	68.42784882	1.02056713	66.66847706	1.034456019	71.38268948
1.036493056	68.14965725	1.022604167	67.80355453	1.036493056	71.26377583
2.0315625	69.86287117	2.017673611	68.462677	2.0315625	73.63532543
2.034050926	69.01287079	2.020162037	68.97542953	2.034050926	73.25318813
2.909398148	71.01266384	2.895509259	68.3769989	2.909398148	73.15312862
2.911377315	70.94273567	2.897488426	67.74929523	2.911377315	72.77268887
7.092604167	70.85490227	7.078715278	69.97133255	7.092604167	74.74411488
7.094976852	70.83232403	7.081087963	70.23526669	7.094976852	74.92685795
14.88707176	62.521348	14.87318287	66.87488079	14.88707176	66.37235165
14.88952546	62.59772778	14.87563657	66.65092468	14.88952546	66.55648708
28.83755787	49.21293736	28.82366898	51.77596569	28.83755787	47.77413368
28.84013889	49.3305397	28.82625	51.86123848	28.84013889	48.12348366
56.96059028	36.37798548	56.94670139	36.67481422	56.96059028	34.64813471
56.96258102	36.28078461	56.94869213	37.14383125	56.96258102	34.81078625
70.22501157	27.53257275	70.21112269	27.24304438	70.22501157	23.63057137
70.2371412	27.29725122	70.22325231	27.2568512	70.2371412	23.13568115
85.99033565	24.74638224	85.97644676	25.50751686	85.99033565	22.87440777
85.99273148	25.41387796	85.97884259	25.4065609	85.99273148	23.45389605
122.9238657	18.54905367	122.9099769	17.57837653	122.9238657	16.83810711
122.9260069	18.77820969	122.9121181	17.83705592	122.9260069	16.99130297
149.8670949	18.27029824	149.853206	16.99391961	149.8670949	15.64857364
149.8699306	18.31479669	149.8560417	17.11950541	149.8699306	15.8328414
182.9947801	14.43744659	182.9808912	13.06482315	182.9947801	12.12352514
182.9980903	14.51545119	182.9842014	13.09864163	182.9980903	12.35575795
**Mouse euthanized on day 202**		247.9501042	12.31953979	**Mouse euthanized on day 202**	
		247.9521412	12.29150057		

Table S58. K_{10} of intramuscular depot **E2** in M1, M2, and M3 as a function of time.

Mouse M1		Mouse M2		Mouse M3	
**Time (d)**	**K_{10} (%)**	**Time (d)**	**K_{10} (%)**	**Time (d)**	**K_{10} (%)**
0.013229167	74.54263687	0.0234375	84.04162407	0.034965278	83.86631966
0.015289352	75.49448967	0.025266204	83.7520504	0.035914352	83.93517494
0.232534722	79.69047546	0.255555556	75.83781242	0.244444444	75.15275478
0.236168981	79.15197372	0.25625	78.06889057	0.245138889	74.97038364
1.077766204	73.04368973	1.065266204	74.14980412	1.054155093	73.92457008
1.080115741	71.16875648	1.067615741	74.28820133	1.05650463	75.72948933
2.060162037	72.97492981	2.047662037	72.93012619	2.036550926	73.81106377
2.062291667	72.65646935	2.049791667	72.59642601	2.038680556	74.73073483
3.050717593	74.62489128	3.038217593	75.0114584	3.027106481	73.28539371
3.053425926	75.12140274	3.040925926	73.90045166	3.029814815	73.6635828
7.078657407	75.02110958	7.066157407	74.0094471	7.055046296	74.15560246
7.081180556	73.57285976	7.068680556	73.74749184	7.057569444	74.15856838
15.1159375	72.50425816	15.1034375	67.05339909	15.09232639	76.37132645
15.11893519	72.52802372	15.10643519	68.76356602	15.09532407	75.97222805
29.1940162	61.7694521	29.1815162	51.53525829	29.17040509	58.39915276
29.19615741	62.02697277	29.18365741	51.38659477	29.1725463	58.78214359
56.19212963	46.95324898	56.17962963	39.68759775	56.16851852	51.45999432
56.19475694	47.6113224	56.18225694	39.82174635	56.17114583	51.67498112
70.36564815	30.66850901	70.35314815	27.99679279	70.34203704	40.21193027
70.36997685	31.0080719	70.35747685	27.30756283	70.34636574	39.83471394
86.18175926	28.76884937	86.16925926	29.93571997	86.15814815	36.54090405
86.18390046	29.66383457	86.17140046	26.45620823	86.16028935	37.0004344
122.1954282	18.18537831	122.1829282	16.42047405	122.1718171	26.50117397
122.1973843	18.42770457	122.1848843	16.57151341	122.1737731	26.84882402
150.1613657	19.05723095	150.1488657	17.33098269	150.1377546	27.15617657
150.1636574	19.25817251	150.1511574	17.48161316	150.1400463	26.81695461
184.1498843	14.10856485	184.1373843	13.9554286	184.1262731	19.42554593
184.1524421	14.23628688	184.1399421	13.79044533	184.128831	20.15944481
**Mouse euthanized on day 202**		247.181713	13.17429185	247.1706019	18.19272399
		247.1842477	13.31602931	247.1731366	18.16027522

Table S59. K_{10} of intramuscular depot **P1** in M1, M2, and M3 as a function of time.

**Mouse M1**		**Mouse M2**		**Mouse M3**	
**Time (d)**	**K_{10} (%)**	**Time (d)**	**K_{10} (%)**	**Time (d)**	**K_{10} (%)**
0.011805556	77.01307774	0.028703704	75.52524567	0.054027778	71.94939613
0.01400463	77.06967354	0.029409722	75.36076546	0.054965278	72.08173275
0.235023148	57.00844288	0.218668981	54.32581425	0.213414352	62.80666351
0.236944444	56.78850651	0.220844907	54.54392433	0.215532407	61.67158604
1.057268519	43.42270374	1.007962963	44.32179928	0.98712963	47.05705166
1.059409722	43.87934685	1.010104167	43.11275959	0.989270833	46.74778938
1.933009259	44.23954964	1.883703704	44.22008038	1.86287037	47.04092503
1.935358796	44.11792755	1.886053241	43.20636272	1.865219907	49.0935564
2.873622685	46.51180267	2.82431713	45.86519241	2.803483796	48.97141933
2.875810185	45.34715652	2.82650463	44.88253117	2.805671296	48.19950581
6.906076389	48.78259182	6.856770833	48.16100597	6.8359375	50.66718578
6.908587963	48.03927898	6.859282407	48.66230011	6.838449074	50.35177231
14.91810185	44.94231701	14.8687963	45.50329685	14.84796296	48.75303268
14.92077546	44.52620029	14.87146991	43.92827034	14.85063657	50.05172253
28.94070602	49.74843025	28.89140046	45.18783092	28.87056713	52.62085915
28.94372685	50.44732094	28.8944213	45.17012596	28.87358796	52.1885252
56.02479167	35.4938674	55.97548611	32.3109889	55.95465278	39.25337076
56.02895833	36.11939907	55.97965278	32.92398691	55.95881944	40.40024281
70.20241898	26.76591396	70.15311343	24.1306138	70.13228009	31.10733032
70.20532407	26.63106918	70.15601852	24.53996658	70.13518519	31.20112181
87.04762731	27.91104317	86.99832176	24.82721567	86.97748843	31.44637823
87.05053241	27.71194696	87.00122685	25.48583269	86.98039352	32.31193066
122.0149884	17.53047705	121.9656829	18.11751723	121.9448495	23.46608639
122.0171181	18.0128479	121.9678125	17.53821135	121.9469792	24.08448458
149.9853588	18.67975235	149.9360532	17.44545698	149.9152199	20.80200434
149.9875231	18.31422806	149.9382176	17.89865494	149.9173843	21.20006561
183.9723264	13.12685013	183.9230208	12.45522141	183.9021875	14.4643414
183.9745139	13.05195689	183.9252083	12.18260884	183.904375	14.30350065
**Mouse euthanized on day 202**		246.96334	12.08222389	246.9425116	12.7798903
		246.96572	12.046628	246.9448843	12.78747201

Table S60. K_{10} of intramuscular depot **P2** in M1, M2, and M3 as a function of time.

Mouse M1		Mouse M2		Mouse M3	
**Time (d)**	**K_{10} (%)**	**Time (d)**	**K_{10} (%)**	**Time (d)**	**K_{10} (%)**
0.014803241	70.11885166	0.014409722	78.64177704	0.013541667	74.13828373
0.016574074	70.22106647	0.016076389	79.01288986	0.015277778	73.90015602
0.22724537	45.28098106	0.229861111	54.51322556	0.228171296	55.76928139
0.22912037	45.00407219	N/A	N/A	0.230138889	56.10991478
1.009907407	39.39031839	0.996018519	43.14018726	0.980740741	47.06068993
1.012199074	39.01876211	0.998310185	42.56523609	0.983032407	50.2063942
2.023923611	41.02406979	2.010034722	42.80132771	1.994756944	49.9673748
2.026284722	40.02824783	2.012395833	42.69516468	1.997118056	49.57762241
2.940914352	41.89181805	2.927025463	45.31376839	2.911747685	48.19779873
2.942997685	41.13101006	2.929108796	45.63067913	2.913831019	49.17175293
7.120925926	44.51135635	7.107037037	47.83801556	7.091759259	56.59598827
7.125763889	43.34206581	7.111875	46.73212051	7.096597222	52.52193928
14.91741898	33.45655441	14.90353009	41.71349525	14.88825231	46.16266727
14.92055556	33.94436836	14.90666667	41.77215576	14.89138889	46.85276031
28.86420139	27.69568205	28.8503125	36.65035248	28.83503472	36.78811312
28.86806713	27.6496911	28.85417824	36.91958427	28.83890046	37.4068141
56.95270833	23.09523106	56.93881944	32.18802691	56.92354167	30.7267642
56.95548611	22.81862974	56.94159722	32.11993694	56.92631944	29.30087805
70.25047454	19.44815159	70.23658565	26.86000109	70.22130787	24.89225149
70.25306713	19.61765051	70.23917824	27.19754934	70.22390046	24.34253454
85.94900463	18.22146297	85.93511574	25.54430723	85.91983796	21.99650764
85.95204861	17.96967745	85.93815972	25.66896677	85.92288194	22.00166464
122.9526968	14.27323937	122.9388079	20.38910866	122.9235301	16.01086855
122.9548148	14.7177875	122.9409259	20.50328016	122.9256481	15.67570806
149.8940856	14.07155752	149.8801968	19.15093422	149.864919	14.59581137
149.8969676	14.19748187	149.8830787	19.11655426	149.8678009	14.4760716
183.0275694	12.24226952	183.0136806	14.09292698	182.9984028	11.52735233
183.0298727	12.26159096	183.0159838	14.04686928	183.000706	11.42374635
247.9976042	12.28992462	247.9837153	12.50985742	247.1143866	12.50985742
247.9996296	12.34635592	247.9857407	12.37498522	247.1165856	12.37498521

Table S61. K_{10} of intramuscular depot **Cy7-amine** in M1, M2, and M3 as a function of time.

**Mouse M1**		**Mouse M2**		**Mouse M3**	
**Time (d)**	**K_{10} (%)**	**Time (d)**	**K_{10} (%)**	**Time (d)**	**K_{10} (%)**
0.020185185	54.81778145	0.020960648	80.47237396	0.016712963	49.30695534
0.022141204	55.67899227	0.023159722	80.19345284	0.018668981	50.13559818
0.229814815	50.67507267	0.227604167	78.33385944	0.226342593	51.14891529
0.231770833	51.33954048	0.229560185	78.37739944	0.228298611	50.92093468
1.044039352	50.54820061	1.016956019	75.12274265	1.04056713	55.50796509
1.046574074	51.11907005	1.019490741	76.15283489	1.043101852	56.95086956
2.042719907	54.81807709	2.015636574	76.16026402	2.039247685	51.47031784
2.043680556	54.62744713	2.016597222	76.48131371	2.040208333	53.69921684
5.341053241	61.84679985	5.313969907	75.3407383	5.337581019	45.69670677
5.343483796	61.88730717	5.316400463	75.58095932	5.340011574	45.69946766
6.082465278	61.25618935	6.055381944	75.21213531	6.078993056	44.40709591
6.085023148	61.35564804	6.057939815	75.14201164	6.081550926	48.39803696
15.16583333	43.06127071	15.13875	51.52392387	15.16236111	19.36259031
15.16923611	43.41520786	15.14215278	50.73991776	15.16576389	19.35985208
29.27958333	25.31124592	29.2525	28.76128435	29.27611111	10.63814282
29.28241898	26.49544477	29.25533565	28.88325691	29.27894676	10.75151205
57.31351852	14.8352766	57.28643519	17.06216455	57.3100463	10
57.31608796	14.8721087	57.28900463	17.19299316	57.31261574	10
69.45393519	12.01293111	69.42685185	12.81664968	69.45046296	10
69.45649306	12.05651641	69.42940972	12.77327657	69.45302083	10
85.18834491	11.72004104	85.16126157	12.32740641	85.18487269	10
85.19056713	11.70438886	85.1634838	12.28160143	85.18709491	10
121.0998843	10.74967027	121.0728009	10.41583419	121.096412	10
121.1071065	10.74272037	121.0800231	10.59435129	121.1036343	10
149.2655208	11.16504908	149.2384375	11.02242351	149.2620486	10.07001042
183.2483102	10.53223014	183.2212269	10	183.244838	10
183.2510417	10.53081512	183.2239583	10.00510454	183.2475694	10
246.2715972	11.00295186	246.2445139	10.29013991	**Mouse euthanized on day 202**	
246.2734259	11.11291409	246.2463426	10.33914089		

S12.1.5. Kidney depot dissolution

Table S62. Signal in kidney (I_{KID}) of **F1** in mice M1, M2, and M3 as a function of time; background noise ($I_{KID,noise}$) was not subtracted.

Mouse M1		Mouse M2		Mouse M3	
**Time (d)**	I_{KID}	**Time (d)**	I_{KID}	**Time (d)**	I_{KID}
0.0104051	140607	0.0141204	152184	0.0099421	177858
0.2415972	283035	0.2291898	751235	0.226412	702060
1.0376273	527829	1.0008218	1296815	0.9813773	1528959
1.9889699	578853	1.9521644	1493353	1.9327199	1636223
2.9303009	539879	2.8934954	1393575	2.8740509	1478962
6.9588773	564345	6.9220718	1320925	6.9026273	1365467
14.956123	488474	14.919317	1068146	14.899873	913830
28.989468	408851	28.952662	922242	28.933218	698560
56.107627	271704	56.070822	612046	56.051377	386420
70.255405	225390	70.2186	567272	70.199155	300975
86.114213	229936	86.077407	516275	86.057963	281473
122.07934	185691	122.04253	433669	122.02309	168004
150.03809	233922	150.00128	405694	149.98184	229288
184.03082	177028	183.99402	356771	183.97457	167246
247.07072	180310	**mice euthanized**		247.01447	168572

Table S63. Signal in kidney (I_{KID}) of **F2** in mice M1, M2, and M3 as a function of time; background noise ($I_{KID,noise}$) was not subtracted.

Mouse M1		Mouse M2		Mouse M3	
**Time (d)**	I_{KID}	**Time (d)**	I_{KID}	**Time (d)**	I_{KID}
0.0072222	174594	0.0132639	371880	0.0154282	236639
0.2294792	9335694	0.2290162	10446608	0.2321991	13427451
1.0433102	9473530	1.0294213	11012211	1.008588	12236199
2.042338	6083416	2.0284491	8297999	2.0076157	8397864
2.9727778	5440049	2.9588889	6501616	2.9380556	6852644
7.1558218	3964027	7.1419329	4482615	7.1210995	4793175
14.946852	2725815	14.932963	3230568	14.91213	3678377
28.874005	2224073	28.860116	2492229	28.839282	2836652
56.976782	1504187	56.962894	1745046	56.94206	1727869
70.177234	895692	70.163345	1294274	70.142512	1175588
85.965752	1144888	85.951863	1201754	85.93103	1244200
122.98825	805874	122.97436	859460	122.95353	689799
149.92468	722635	149.91079	827373	149.88995	876875
183.06418	732651	183.05029	696456	183.02946	772718
247.08731	553517	**mice euthanized**		247.05259	543759

Table S64. Signal in kidney (I_{KID}) of I1 in mice M1, M2, and M3 as a function of time; background noise ($I_{KID,noise}$) was not subtracted.

Mouse M1		Mouse M2		Mouse M3	
**Time (d)**	I_{KID}	**Time (d)**	I_{KID}	**Time (d)**	I_{KID}
0.0228704	174582	0.0127199	168234	0.0098843	162082
0.2268287	654314	0.2286921	825282	0.2277662	1158997
1.0209028	967292	1.0184491	1057355	1.0251852	2230053
2.0391319	789920	2.0085764	920021	1.9877431	1409503
3.0283912	818072	2.9978356	949231	2.9770023	1414261
7.2055903	632004	7.1750347	753787	7.1542014	1155934
14.958611	435215	14.928056	552702	14.907222	817122
28.923437	313797	28.892882	392190	28.872049	564596
57.028461	238133	56.997905	272512	56.977072	428769
70.180602	209338	70.150046	202615	70.129213	286789
86.01662	195594	85.986065	222094	85.965231	306981
123.03513	170887	123.00457	155192	122.98374	231054
149.97749	186510	149.94693	187125	149.9261	232693
183.11153	189582	183.08097	151269	183.06014	218194
**mice euthanized**		247.11235	149393	247.09152	156727

Table S65. Signal in kidney (I_{KID}) of I2 in mice M1, M2, and M3 as a function of time; background noise ($I_{KID,noise}$) was not subtracted.

Mouse M1		Mouse M2		Mouse M3	
**Time (d)**	I_{KID}	**Time (d)**	I_{KID}	**Time (d)**	I_{KID}
0.0269792	256734	0.0152662	215239	0.0109838	208492
0.2394097	336054	0.2665162	233466	0.2479745	313130
1.0701273	284463	1.0277662	248869	0.9958218	426937
2.0314005	334658	1.9890394	283743	1.9570949	332432
3.0210301	300113	2.978669	264874	2.9467245	369254
7.049294	288668	7.0069329	298509	6.9749884	403020
15.042836	217938	15.000475	230409	14.96853	289324
29.177245	168761	29.134884	158412	29.10294	190989
56.164213	141412	56.121852	153295	56.089907	204506
70.342859	181345	70.300498	148960	70.268553	155038
86.208356	128384	86.165995	126745	86.134051	138350
122.17288	126564	122.13052	128400	122.09858	122046
150.13257	184385	150.09021	153197	150.05826	163442
184.12027	132359	184.07791	140795	184.04596	138717
247.16368	147344	**mice euthanized**		247.08938	148064

Table S66. Signal in kidney (I_{KID}) of **E1** in mice M1, M2, and M3 as a function of time; background noise ($I_{KID,noise}$) was not subtracted.

Mouse M1		Mouse M2		Mouse M3	
**Time (d)**	I_{KID}	**Time (d)**	I_{KID}	**Time (d)**	I_{KID}
0.0088194	231056	0.0106481	170763	0.0242361	234118
0.230706	654252	0.2331597	464077	0.2343634	664690
1.0324074	867309	1.0185185	684085	1.0074074	965302
2.0293981	853133	2.0155093	697639	2.0043981	1017108
2.9071412	852855	2.8932523	600036	2.8821412	863006
7.0902662	770379	7.0763773	530087	7.0652662	706532
14.884132	493419	14.870243	389219	14.859132	482009
28.832639	263110	28.81875	263225	28.807639	299083
56.958368	209709	56.944479	183943	56.933368	211869
70.220336	192199	70.206447	171785	70.195336	154087
85.988194	166979	85.974306	143561	85.963194	157085
122.93053	164815	122.91664	135317	122.90553	145351
149.86485	170371	149.85096	164568	149.83985	156389
182.99272	160532	182.97883	150514	182.96772	147185
**mice euthanized**		247.94807	161642	247.93696	184511

Table S67. Signal in kidney (I_{KID}) of **E2** in mice M1, M2, and M3 as a function of time; background noise ($I_{KID,noise}$) was not subtracted.

Mouse M1		Mouse M2		Mouse M3	
**Time (d)**	I_{KID}	**Time (d)**	I_{KID}	**Time (d)**	I_{KID}
0.0107755	190411	0.0186227	204967	0.0289699	563617
0.2405556	520979	0.2502778	658155	0.2391667	1844345
1.0703241	650193	1.0578241	855340	1.046713	1619696
2.0537616	655039	2.0412616	838905	2.0301505	1524557
3.0454861	635364	3.0329861	839821	3.021875	1728425
7.0718403	544230	7.0593403	679063	7.0482292	1382414
15.059213	378778	15.046713	491517	15.035602	857847
29.19103	278968	29.17853	268868	29.167419	571543
56.187951	194778	56.175451	224259	56.16434	304158
70.363113	188068	70.350613	204730	70.339502	270328
86.179213	176015	86.166713	174824	86.155602	225716
122.20263	157250	122.19013	145149	122.17902	163427
150.15926	198200	150.14676	196540	150.13565	234525
184.14786	140852	184.13536	146878	184.12425	173839
**mice euthanized**		247.17652	164956	247.16541	162640

Table S68. Signal in kidney (I_{KID}) of **P1** in mice M1, M2, and M3 as a function of time; background noise ($I_{KID,noise}$) was not subtracted.

Mouse M1		Mouse M2		Mouse M3	
**Time (d)**	I_{KID}	**Time (d)**	I_{KID}	**Time (d)**	I_{KID}
0.0097569	975370	0.0230671	2640355	0.0492708	5410546
0.233206	13014232	0.2147338	14061702	0.2075	17663459
1.0550579	12160834	1.0057523	14957370	0.984919	18925499
1.9308681	11010278	1.8815625	13117528	1.8607292	15389542
2.8715509	10591136	2.8222454	12029803	2.801412	14768532
6.903669	7042913	6.8543634	8280628	6.8335301	9399785
14.91559	3092862	14.866285	3358774	14.845451	4171016
28.937905	1173648	28.8886	1300311	28.867766	1677314
56.019294	370395	55.969988	361849	57.721262	377389
70.198854	318915	70.149549	340618	70.128715	380432
87.04566	207300	86.996354	223799	86.975521	197814
122.02236	145593	121.97306	139997	121.95222	162104
149.98287	175664	149.93356	167297	149.91273	164532
183.96807	142300	183.91876	139550	183.89793	136855
**mice euthanized**		246.96124	148597	246.94041	130443

Table S69. Signal in kidney (I_{KID}) of **P2** in mice M1, M2, and M3 as a function of time; background noise ($I_{KID,noise}$) was not subtracted.

Mouse M1		Mouse M2		Mouse M3	
**Time (d)**	I_{KID}	**Time (d)**	I_{KID}	**Time (d)**	I_{KID}
0.0185185	4869209	0.011956	405484	0.0118287	419633
0.2336458	9357425	0.2349306	10566113	0.2329398	6430396
1.0074074	8824126	0.9935185	10925642	0.9782407	6761788
2.0216551	7743489	2.0077662	9880518	1.9924884	6565936
2.9389583	7155565	2.9250694	9451225	2.9097917	4745449
7.1175926	5269490	7.1037037	6484080	7.0884259	3523210
14.912407	2309205	14.898519	2593250	14.883241	2131593
28.861181	1012890	28.847292	1197029	28.832014	794262
56.95037	380054	56.936481	409446	56.921204	235214
70.247905	290191	70.234016	308434	70.218738	198606
85.946771	208568	85.932882	215629	85.917604	172312
122.96112	159649	122.94723	167212	122.93196	127107
149.89138	177204	149.87749	183203	149.86221	166912
183.02538	169229	183.01149	154400	182.99622	144602
247.99529	180402	247.9814	158705	247.96612	155558

Table S70. Signal in kidney (I_{KID}) of **Cy7-amine** in mice M1, M2, and M3 as a function of time; background noise ($I_{KID,noise}$) was not subtracted.

**Mouse M1**		**Mouse M2**		**Mouse M3**	
**Time (d)**	$\boldsymbol{I_{KID}}$	**Time (d)**	$\boldsymbol{I_{KID}}$	**Time (d)**	$\boldsymbol{I_{KID}}$
0.0159954	759198	0.0189583	1105527	0.0120486	190569
0.2273032	1540522	0.2215046	1617569	0.23625	270346
1.0378819	2593395	1.0107986	2836181	0.9927431	272497
2.034456	2809501	2.0073727	2778363	1.9893171	196957
5.3386343	1968813	5.3115509	1799942	5.2934954	162134
6.0773148	1620044	6.0502315	1583665	6.0321759	195032
15.14912	812809	15.122037	777080	15.103981	175837
29.277373	298488	29.250289	274184	29.232234	120917
57.318808	144898	57.291725	138817	57.273669	120235
69.451343	148272	69.424259	125962	69.406204	125074
85.185671	134817	85.158588	132420	85.140532	125805
121.10191	147363	121.07483	132317	121.05677	119350
149.26334	162954	149.23626	144686	149.21821	148556
183.25287	125037	183.22579	126389	183.20773	112239
246.26965	148398	246.24257	139840	**mice euthanized**	

S12.1.6. Liver depot dissolution

Table S71. Signal in liver (I_{LIV}) of **F1** in mice M1, M2, and M3 as a function of time; background noise ($I_{LIV,noise}$) was not subtracted.

**Mouse M1**		**Mouse M2**		**Mouse M3**	
**Time (d)**	I_{KID}	**Time (d)**	I_{KID}	**Time (d)**	I_{KID}
0.0104051	546744	0.0141204	519870	0.0099421	545654
0.2415972	938587	0.2291898	1936882	0.226412	1895034
1.0376273	1322228	1.0008218	2986313	0.9813773	3490099
1.9889699	1422161	1.9521644	2690267	1.9327199	3727214
2.9303009	1328725	2.8934954	2974617	2.8740509	3523695
6.9588773	1193374	6.9220718	2336079	6.9026273	2893091
14.956123	933402	14.919317	1755073	14.899873	1709741
28.989468	701057	28.952662	1305820	28.933218	1060022
56.107627	508701	56.070822	884998	56.051377	661182
70.255405	492857	70.2186	946348	70.199155	601181
86.114213	436861	86.077407	618102	86.057963	510609
122.07934	450603	122.04253	523681	122.02309	483648
150.03809	563492	150.00128	653672	149.98184	542391
184.03082	472866	183.99402	530252	183.97457	445315
247.07072	523975	**mice euthanized**		247.01447	459385

Table S72. Signal in liver (I_{LIV}) of **F2** in mice M1, M2, and M3 as a function of time; background noise ($I_{LIV,noise}$) was not subtracted.

**Mouse M1**		**Mouse M2**		**Mouse M3**	
**Time (d)**	I_{KID}	**Time (d)**	I_{KID}	**Time (d)**	I_{KID}
0.0072222	683205	0.0132639	1185106	0.0154282	647614
0.2294792	25924597	0.2290162	22391086	0.2321991	26782468
1.0433102	27059840	1.0294213	25291113	1.008588	23873387
2.042338	20318016	2.0284491	15747051	2.0076157	16237047
2.9727778	14972266	2.9588889	12951366	2.9380556	14170712
7.1558218	10264600	7.1419329	9777425	7.1210995	11673301
14.946852	7707284	14.932963	7505885	14.91213	7480938
28.874005	5418213	28.860116	4899305	28.839282	4796356
56.976782	3667380	56.962894	3202228	56.94206	2946065
70.177234	2756303	70.163345	2628670	70.142512	2665688
85.965752	2720780	85.951863	2655514	85.93103	2428158
122.98825	1911434	122.97436	2015907	122.95353	1753460
149.92468	2213760	149.91079	2113642	149.88995	1548796
183.06418	1996742	183.05029	1927905	183.02946	1420266
247.08731	1169541	**mice euthanized**		247.05259	865857

Table S73. Signal in liver (I_{LIV}) of **I1** in mice M1, M2, and M3 as a function of time; background noise ($I_{LIV,noise}$) was not subtracted.

Mouse M1		Mouse M2		Mouse M3	
**Time (d)**	I_{KID}	**Time (d)**	I_{KID}	**Time (d)**	I_{KID}
0.0228704	605263	0.0127199	583447	0.0098843	503956
0.2268287	861714	0.2286921	1749767	0.2277662	3305853
1.0209028	3034689	1.0184491	3711537	1.0251852	5249427
2.0391319	3496187	2.0085764	3746638	1.9877431	5923155
3.0283912	2791649	2.9978356	3426599	2.9770023	4369225
7.2055903	2454375	7.1750347	2447058	7.1542014	3237253
14.958611	1321432	14.928056	1879303	14.907222	2023334
28.923437	925373	28.892882	1057714	28.872049	1368948
57.028461	628963	56.997905	629580	56.977072	791368
70.180602	626426	70.150046	673650	70.129213	779333
86.01662	562726	85.986065	534382	85.965231	589737
123.03513	454564	123.00457	451922	122.98374	503701
149.97749	557413	149.94693	511200	149.9261	596588
183.11153	515975	183.08097	450605	183.06014	456352
**mice euthanized**		247.11235	424598	247.09152	501744

Table S74. Signal in liver (I_{LIV}) of **I2** in mice M1, M2, and M3 as a function of time; background noise ($I_{LIV,noise}$) was not subtracted.

Mouse M1		Mouse M2		Mouse M3	
**Time (d)**	I_{KID}	**Time (d)**	I_{KID}	**Time (d)**	I_{KID}
0.0269792	2146636	0.0152662	718801	0.0109838	550454
0.2394097	1673871	0.2665162	839941	0.2479745	897427
1.0701273	1536890	1.0277662	1041362	0.9958218	1275761
2.0314005	1476571	1.9890394	1113973	1.9570949	1047924
3.0210301	1213667	2.978669	1128624	2.9467245	1113561
7.049294	1099100	7.0069329	1068848	6.9749884	1106966
15.042836	779683	15.000475	635205	14.96853	695697
29.177245	532852	29.134884	488471	29.10294	423368
56.164213	464609	56.121852	410249	56.089907	441942
70.342859	558988	70.300498	500217	70.268553	442825
86.208356	404954	86.165995	354638	86.134051	354622
122.17288	429495	122.13052	390638	122.09858	364969
150.13257	588920	150.09021	476093	150.05826	505561
184.12027	423920	184.07791	392386	184.04596	395968
247.16368	516664	**mice euthanized**		247.08938	448248

Table S75. Signal in liver (I_{LIV}) of **E1** in mice M1, M2, and M3 as a function of time; background noise ($I_{LIV,noise}$) was not subtracted.

Mouse M1		Mouse M2		Mouse M3	
**Time (d)**	$\boldsymbol{I_{KID}}$	**Time (d)**	$\boldsymbol{I_{KID}}$	**Time (d)**	$\boldsymbol{I_{KID}}$
0.0088194	709489	0.0106481	663628	0.0242361	615860
0.230706	3896073	0.2331597	2571785	0.2343634	2571164
1.0324074	6754441	1.0185185	4213170	1.0074074	4694740
2.0293981	6511506	2.0155093	5067645	2.0043981	5585845
2.9071412	4461608	2.8932523	3387998	2.8821412	4036867
7.0902662	3970919	7.0763773	2270341	7.0652662	3066387
14.884132	2588567	14.870243	1781359	14.859132	2048853
28.832639	1732441	28.81875	1281599	28.807639	1216013
56.958368	1048350	56.944479	724404	56.933368	825155
70.220336	808177	70.206447	768677	70.195336	687854
85.988194	719029	85.974306	516619	85.963194	665020
122.93053	537104	122.91664	449870	122.90553	488739
149.86485	603874	149.85096	533936	149.83985	533812
182.99272	534605	182.97883	476650	182.96772	449832
**mice euthanized**		247.94807	529155	**mice euthanized**	

Table S76. Signal in liver (I_{LIV}) of **E2** in mice M1, M2, and M3 as a function of time; background noise ($I_{LIV,noise}$) was not subtracted.

Mouse M1		Mouse M2		Mouse M3	
**Time (d)**	$\boldsymbol{I_{KID}}$	**Time (d)**	$\boldsymbol{I_{KID}}$	**Time (d)**	$\boldsymbol{I_{KID}}$
0.0107755	675743	0.0186227	721474	0.0289699	1407104
0.2405556	2295482	0.2502778	3209422	0.2391667	6804665
1.0703241	5332299	1.0578241	4371456	1.046713	6763132
2.0537616	4357551	2.0412616	3707674	2.0301505	5154788
3.0454861	3649571	3.0329861	3386205	3.021875	5678261
7.0718403	2733737	7.0593403	2410657	7.0482292	4005079
15.059213	1787936	15.046713	1869142	15.035602	2626570
29.19103	1250540	29.17853	1063623	29.167419	1937897
56.187951	773755	56.175451	745070	56.16434	1081093
70.363113	842990	70.350613	612465	70.339502	839424
86.179213	623737	86.166713	587504	86.155602	693979
122.20263	514596	122.19013	496158	122.17902	493595
150.15926	686550	150.14676	573335	150.13565	683821
184.14786	549576	184.13536	481569	184.12425	503200
**mice euthanized**		247.17652	483094	247.16541	512790

Table S77. Signal in liver (I_{LIV}) of **P1** in mice M1, M2, and M3 as a function of time; background noise ($I_{LIV,noise}$) was not subtracted.

**Mouse M1**		**Mouse M2**		**Mouse M3**	
**Time (d)**	I_{KID}	**Time (d)**	I_{KID}	**Time (d)**	I_{KID}
0.0097569	4343090	0.0230671	10530011	0.0492708	18459198
0.233206	22724154	0.2147338	23183645	0.2075	24755274
1.0550579	29053621	1.0057523	31518972	0.984919	22161051
1.9308681	30349779	1.8815625	26214169	1.8607292	22850143
2.8715509	27755761	2.8222454	31484689	2.801412	23492066
6.903669	24608381	6.8543634	27267919	6.8335301	20862378
14.91559	20972629	14.866285	19610325	14.845451	16617783
28.937905	9694845	28.8886	10600817	28.867766	10442209
56.019294	4007619	55.969988	3696585	57.721262	4813217
70.198854	3268703	70.149549	2384911	70.128715	2273725
87.04566	1786513	86.996354	1915336	86.975521	2119627
122.02236	724488	121.97306	671041	121.95222	679419
149.98287	785931	149.93356	710348	149.91273	739216
183.96807	483620	183.91876	475551	183.89793	476981
**mice euthanized**		246.96124	478764	246.94041	486553

Table S78. Signal in liver (I_{LIV}) of **P2** in mice M1, M2, and M3 as a function of time; background noise ($I_{LIV,noise}$) was not subtracted.

**Mouse M1**		**Mouse M2**		**Mouse M3**	
**Time (d)**	I_{KID}	**Time (d)**	I_{KID}	**Time (d)**	I_{KID}
0.0185185	16951596	0.011956	1067817	0.0118287	1240169
0.2336458	25178003	0.2349306	21710094	0.2329398	18113212
1.0074074	27413258	0.9935185	26035510	0.9782407	14492414
2.0216551	25940177	2.0077662	24887849	1.9924884	19228263
2.9389583	23139652	2.9250694	21270234	2.9097917	16995117
7.1175926	20167641	7.1037037	14777905	7.0884259	13676820
14.912407	19005195	14.898519	18552210	14.883241	11384988
28.861181	10158146	28.847292	10359050	28.832014	5115424
56.95037	4712484	56.936481	4741142	56.921204	1197639
70.247905	3796961	70.234016	3011539	70.218738	1120823
85.946771	1687186	85.932882	2112927	85.917604	645876
122.96112	756839	122.94723	629318	122.93196	459830
149.89138	832871	149.87749	814810	149.86221	547701
183.02538	571712	183.01149	560872	182.99622	428278
247.99529	567815	247.9814	560756	247.96612	487727

Table S79. Signal in liver (I_{LIV}) of **Cy7-amine** in mice M1, M2, and M3 as a function of time; background noise ($I_{LIV,noise}$) was not subtracted.

**Mouse M1**		**Mouse M2**		**Mouse M3**	
**Time (d)**	I_{KID}	**Time (d)**	I_{KID}	**Time (d)**	I_{KID}
0.0159954	5633145	0.0189583	8733973	0.0120486	685800
0.2273032	8996903	0.2215046	5567478	0.23625	736535
1.0378819	5147795	1.0107986	3840217	0.9927431	1214758
2.034456	3510654	2.0073727	2730436	1.9893171	638235
5.3386343	1909354	5.3115509	1570430	5.2934954	473778
6.0773148	1906672	6.0502315	1585016	6.0321759	635415
15.14912	924514	15.122037	876153	15.103981	514631
29.277373	494184	29.250289	450163	29.232234	405859
57.318808	397704	57.291725	357914	57.273669	366035
69.451343	419371	69.424259	356736	69.406204	362610
85.185671	419034	85.158588	386815	85.140532	389590
121.10191	472273	121.07483	445370	121.05677	444212
149.26334	483773	149.23626	460625	149.21821	506331
183.25287	424758	183.22579	398604	183.20773	398342
246.26965	469745	246.24257	424098	**mice euthanized**	

S12.2. Polymer pharmacokinetics - fitting curves

S12.2.1. Intramuscular depot dissolution data – signal (I_{IM})

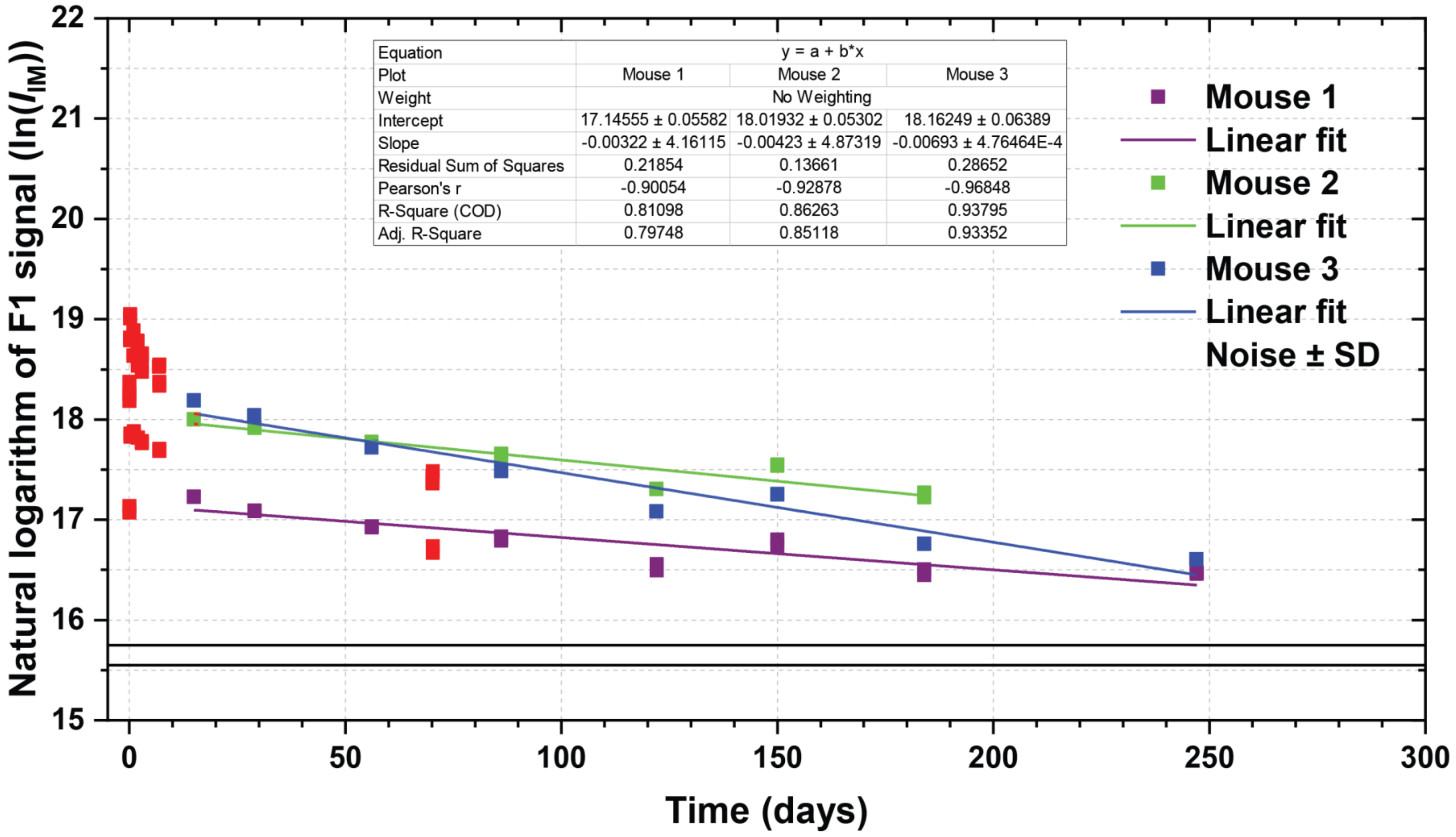

Figure S64. Signal of **F1** (arbitrary units) in mice M1, M2, and M3 as a function of time. **Phase 1a** and **Phase 3a** are marked in red.

 DOI: XXXX.YYYY/ZZZZ

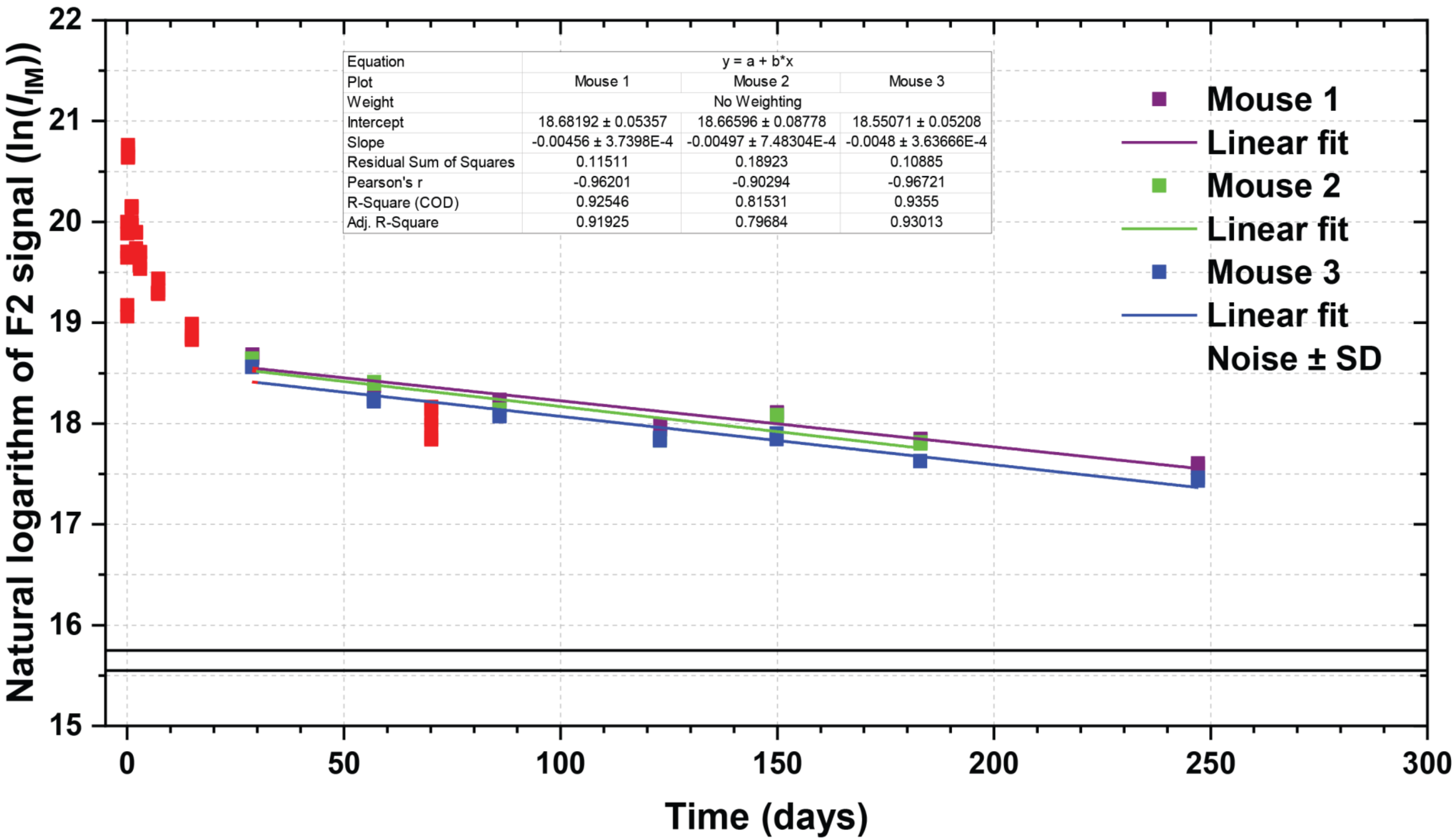

Equation	y = a + b*x		
Plot	Mouse 1	Mouse 2	Mouse 3
Weight	No Weighting		
Intercept	18.68192 ± 0.05357	18.66596 ± 0.08778	18.55071 ± 0.05208
Slope	-0.00456 ± 3.7398E-4	-0.00497 ± 7.48304E-4	-0.0048 ± 3.63666E-4
Residual Sum of Squares	0.11511	0.18923	0.10885
Pearson's r	-0.96201	-0.90294	-0.96721
R-Square (COD)	0.92546	0.81531	0.9355
Adj. R-Square	0.91925	0.79684	0.93013

Figure S65. Signal of F2 (arbitrary units) in mice M1, M2, and M3 as a function of time. **Phase 1a** and **Phase 3a** are marked in red.

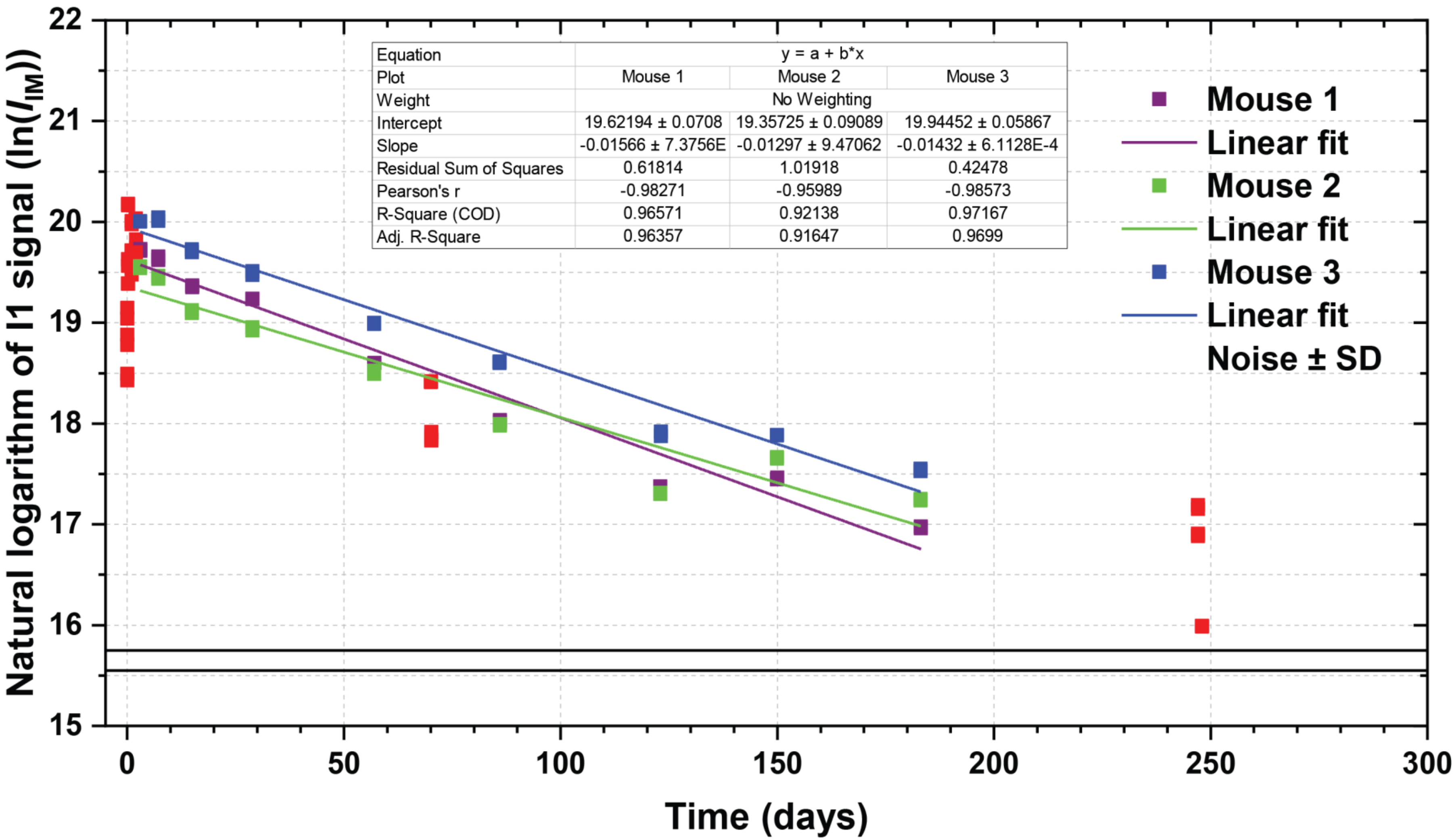

Figure S66. Signal of **I1** (arbitrary units) in mice M1, M2, and M3 as a function of time. **Phase 1a** and **Phase 3a** are marked in red.

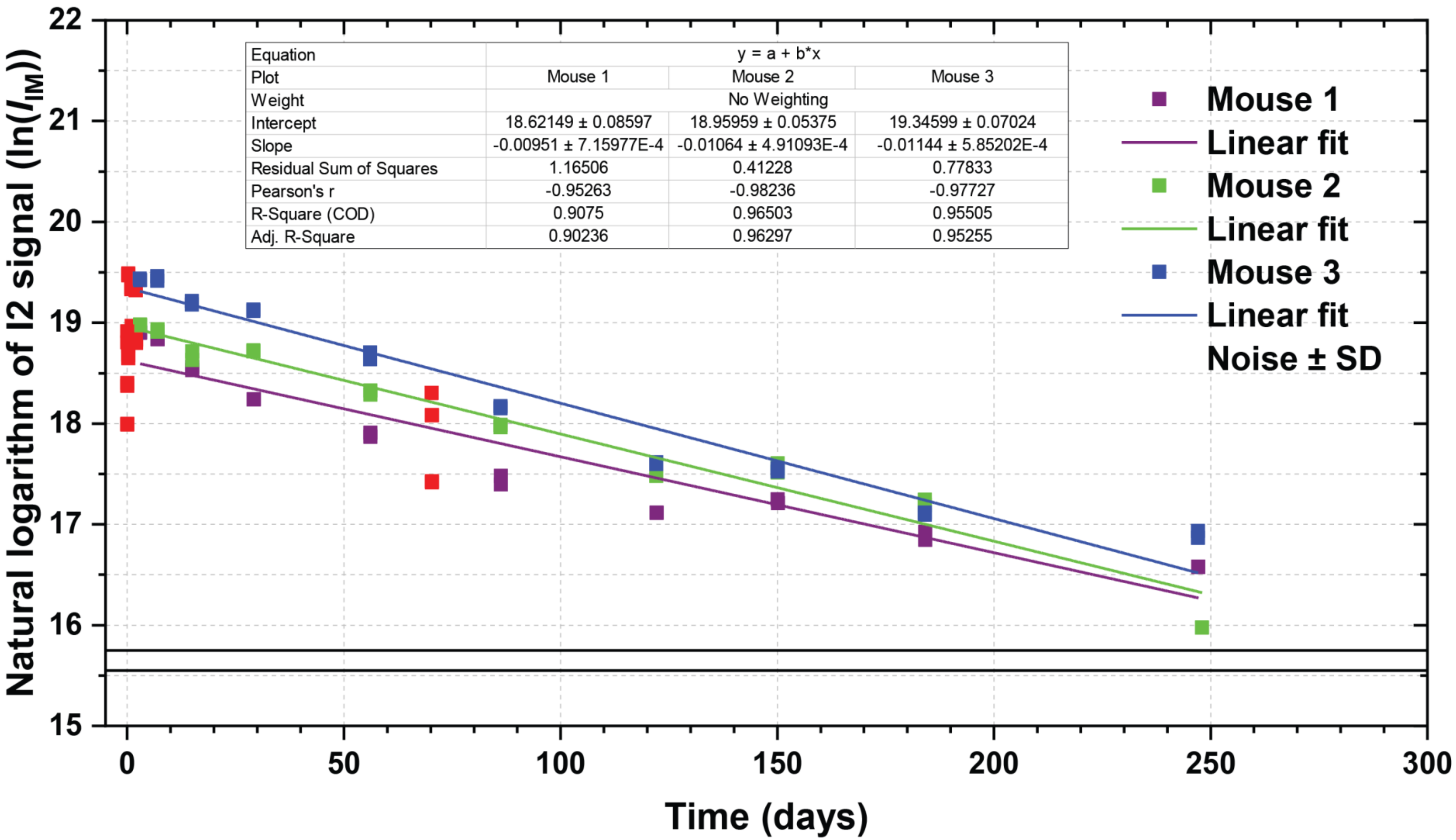

Figure S67. Signal of **I2** (arbitrary units) in mice M1, M2, and M3 as a function of time. **Phase 1a** and **Phase 3a** are marked in red.

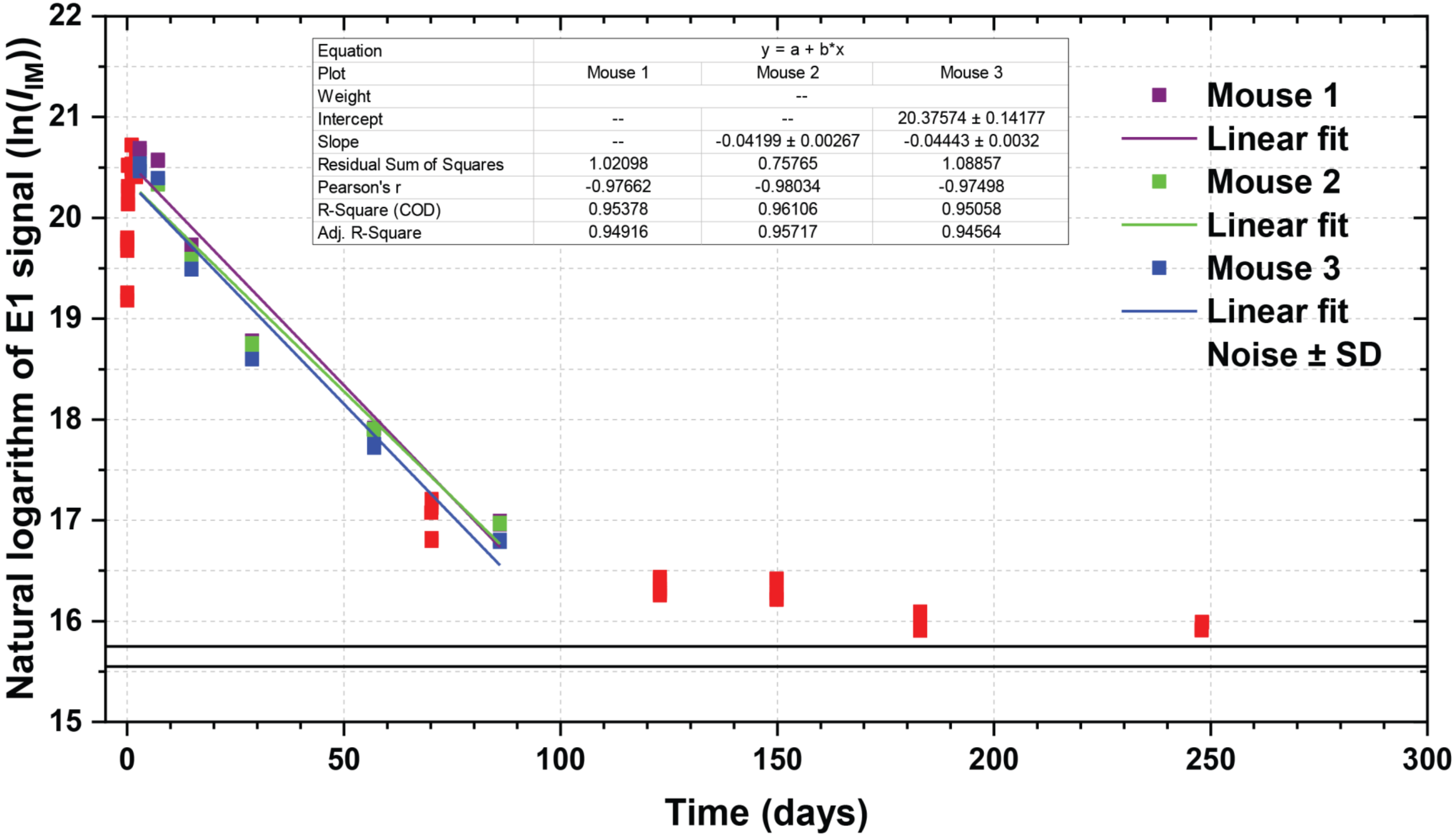

Figure S68. Signal of **E1** (arbitrary units) in mice M1, M2, and M3 as a function of time. **Phase 1a** and **Phase 3a** are marked in red.

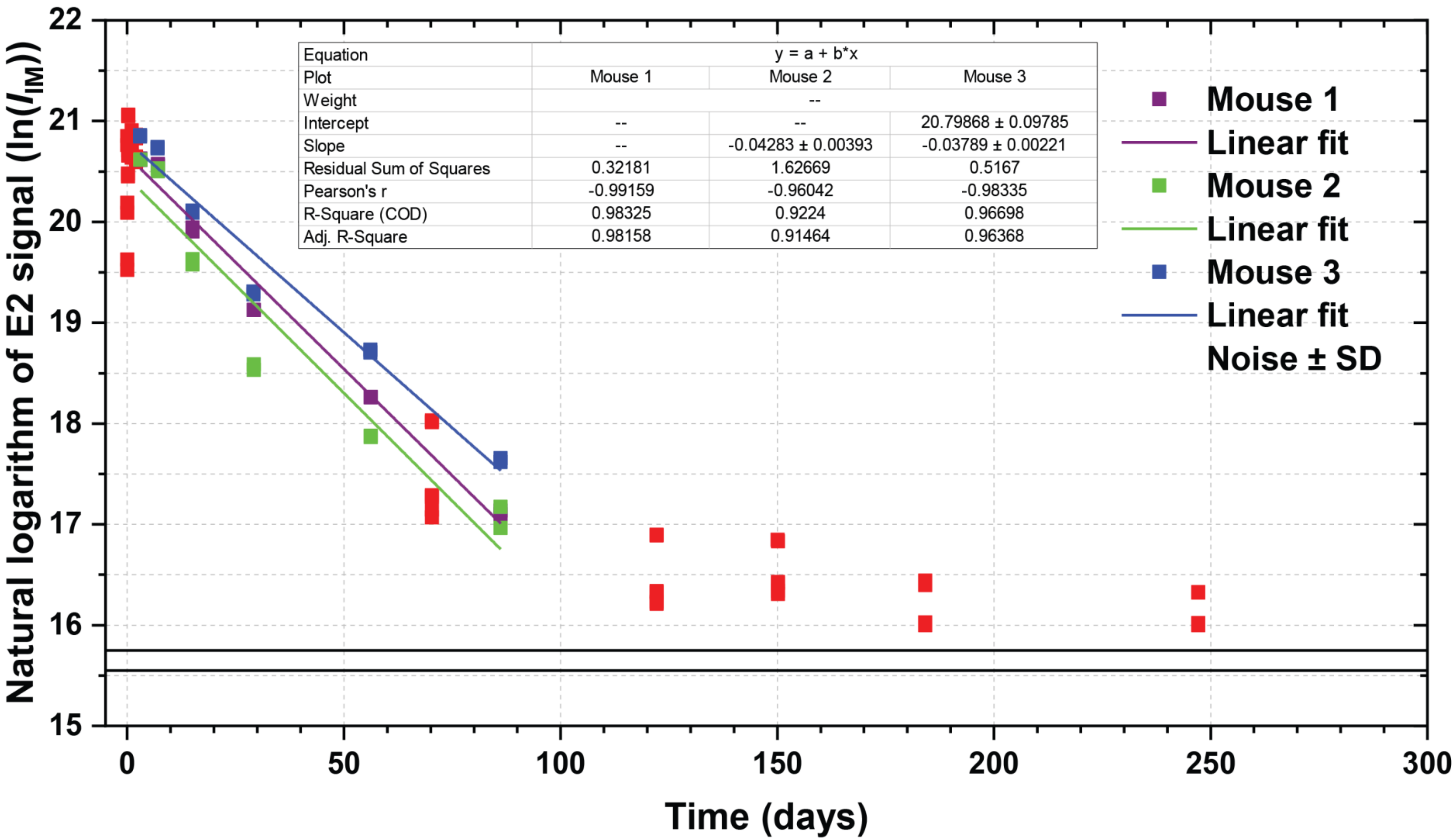

Figure S69. Signal of E2 (arbitrary units) in mice M1, M2, and M3 as a function of time. **Phase 1a** and **Phase 3a** are marked in red.

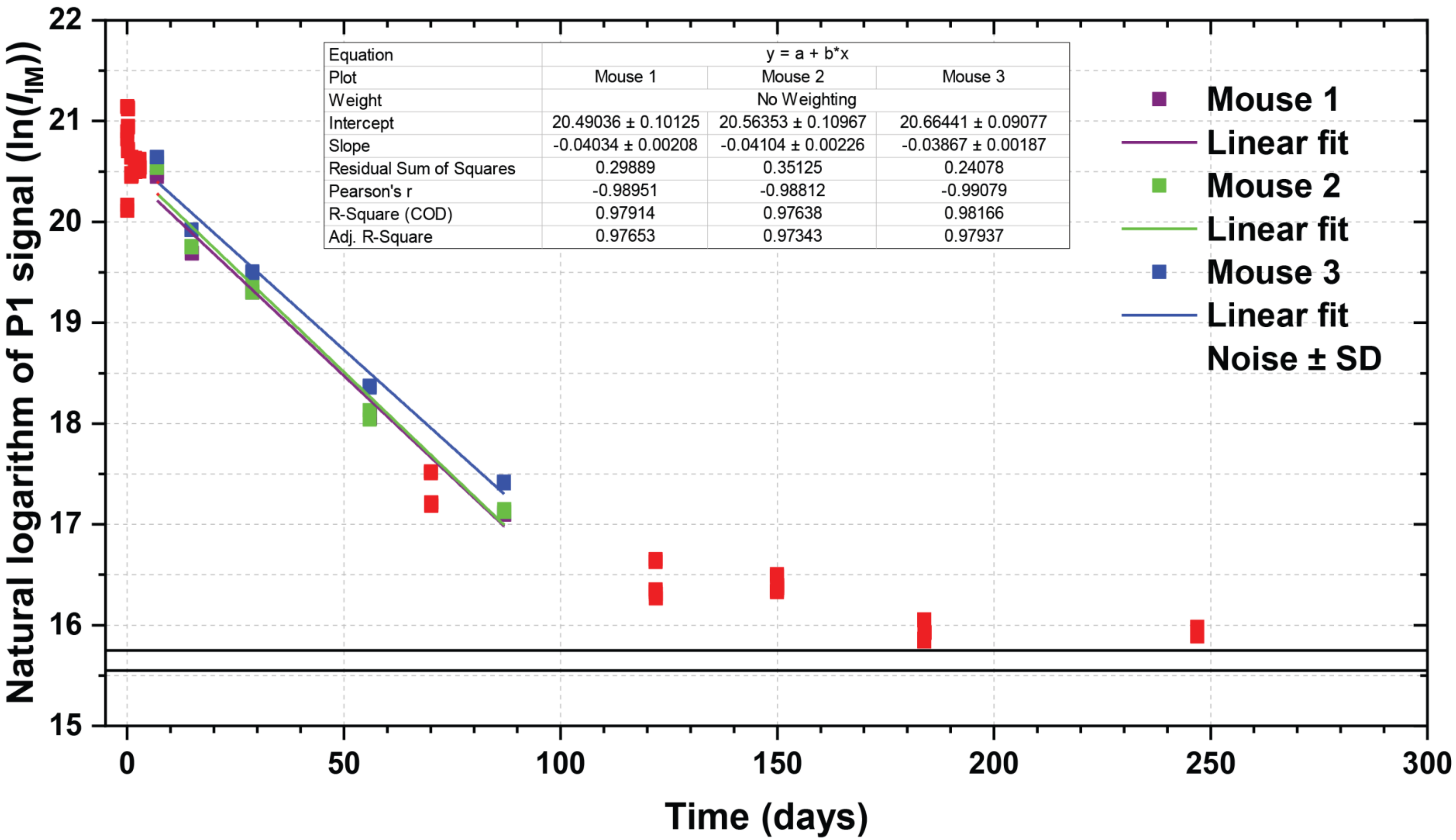

Figure S70. Signal of **P1** (arbitrary units) in mice M1, M2, and M3 as a function of time. **Phase 1a** and **Phase 3a** are marked in red.

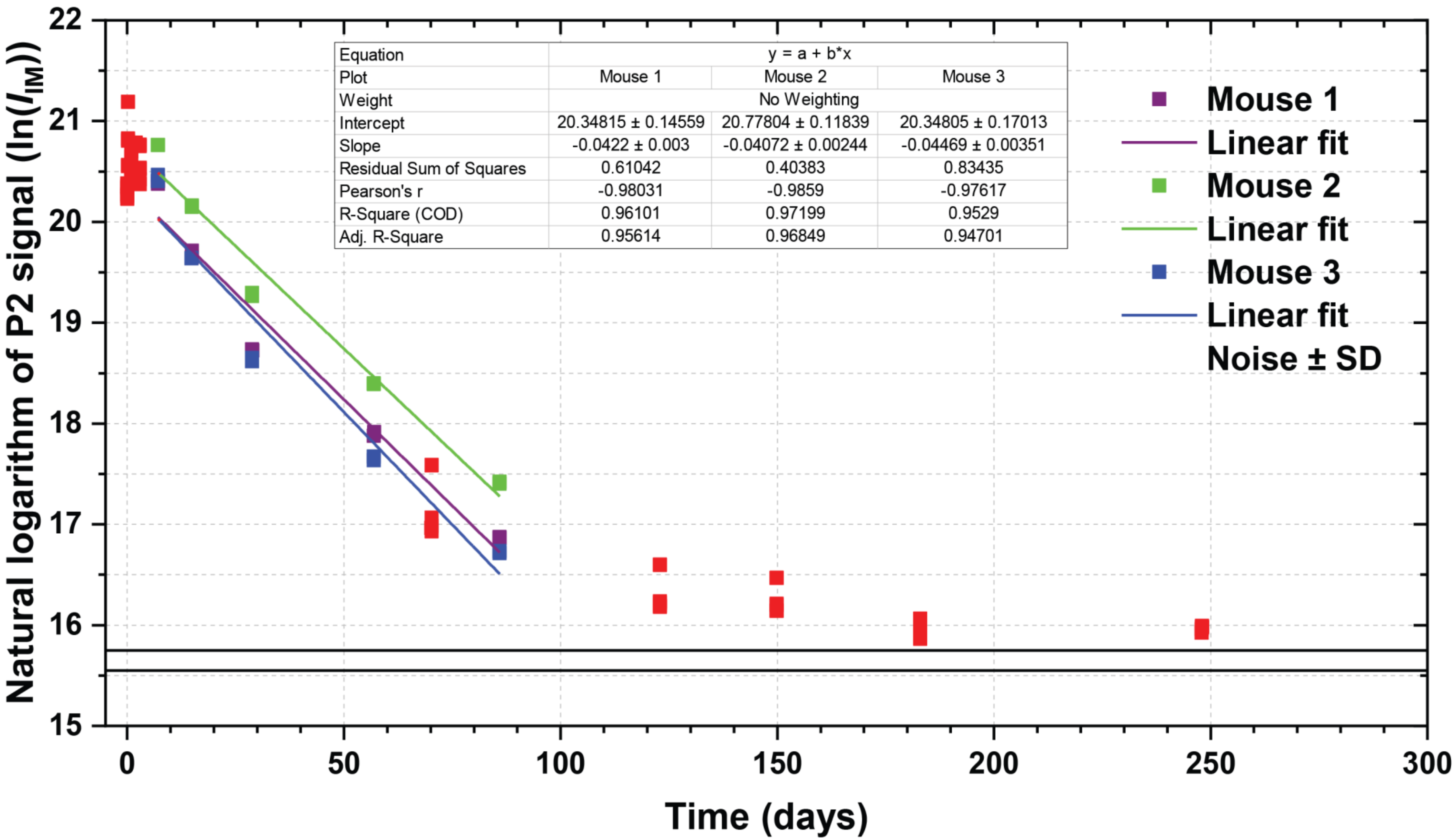

Figure S71. Signal of **P2** (arbitrary units) in mice M1, M2, and M3 as a function of time. **Phase 1a** and **Phase 3a** are marked in red.

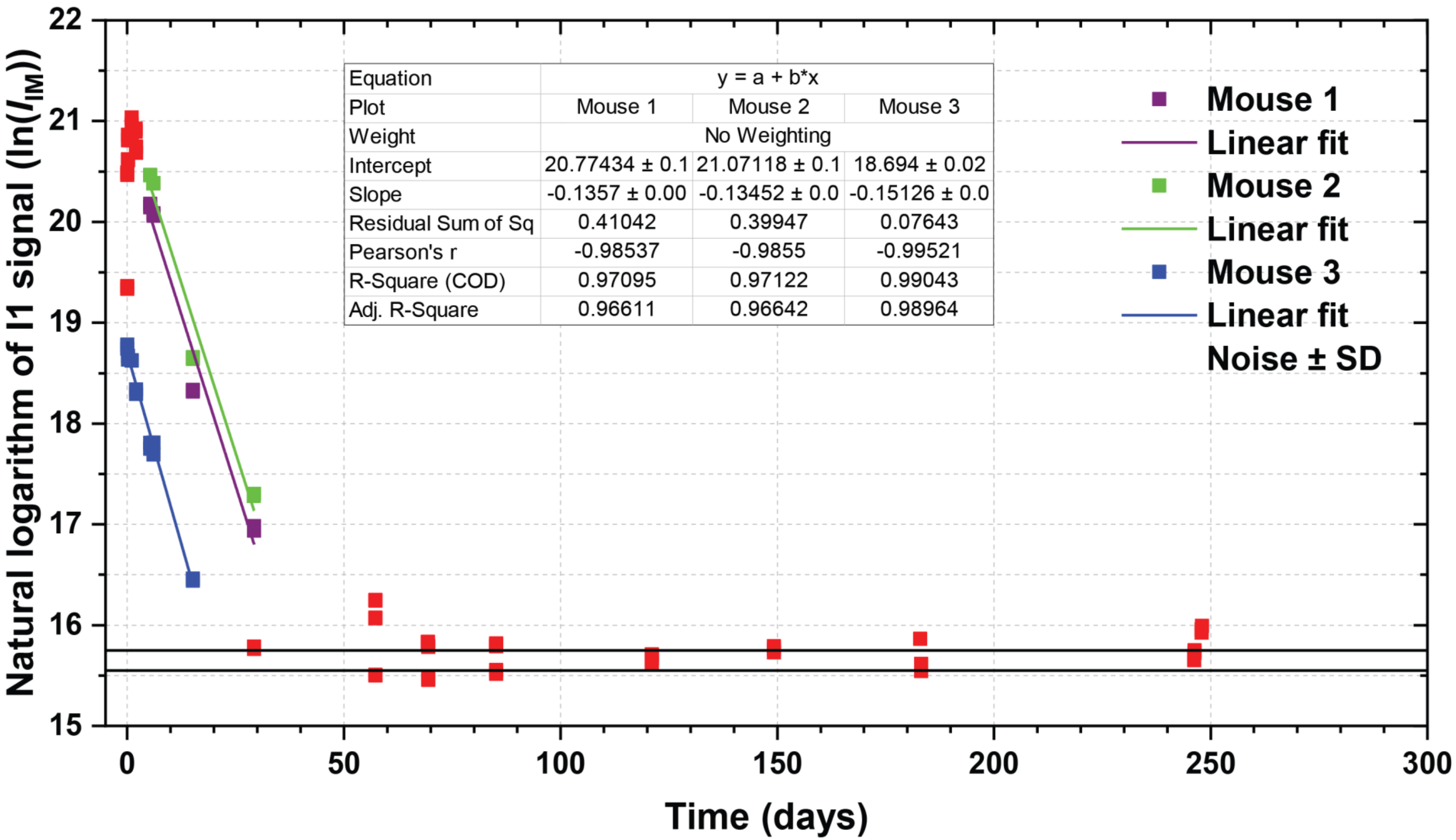

Figure S72. Signal of **Cy7-amine** (arbitrary units) in mice M1, M2, and M3 as a function of time. **Phase 1a** and **Phase 3a** are marked in red.

S12.2.2. Depot dissolution data – depot area (S_{IM})

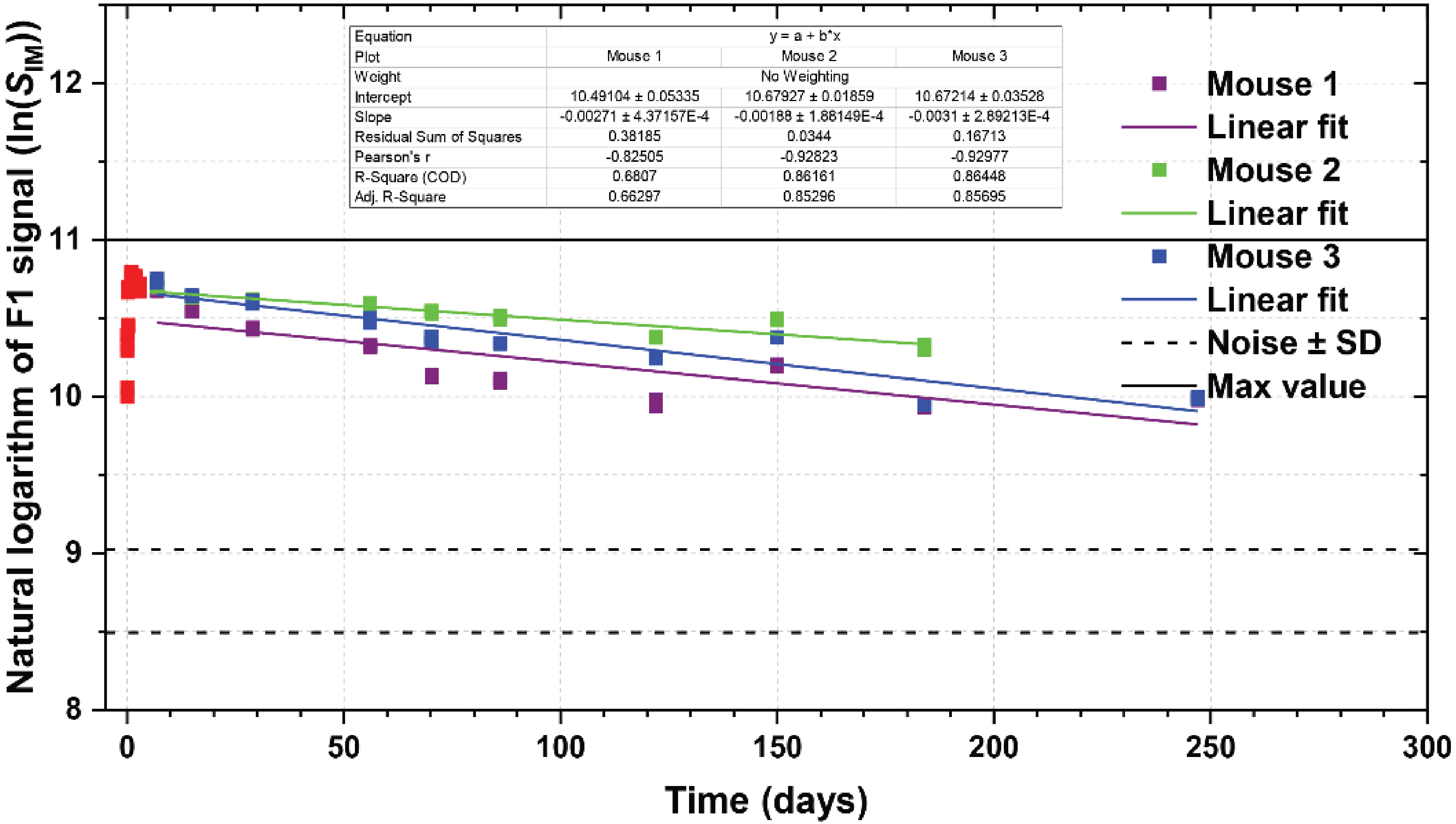

Figure S73. Depot size of **F1** (pixels) in mice M1, M2, and M3 as a function of time. **Phase 1a** and **Phase 3a** are marked in red.

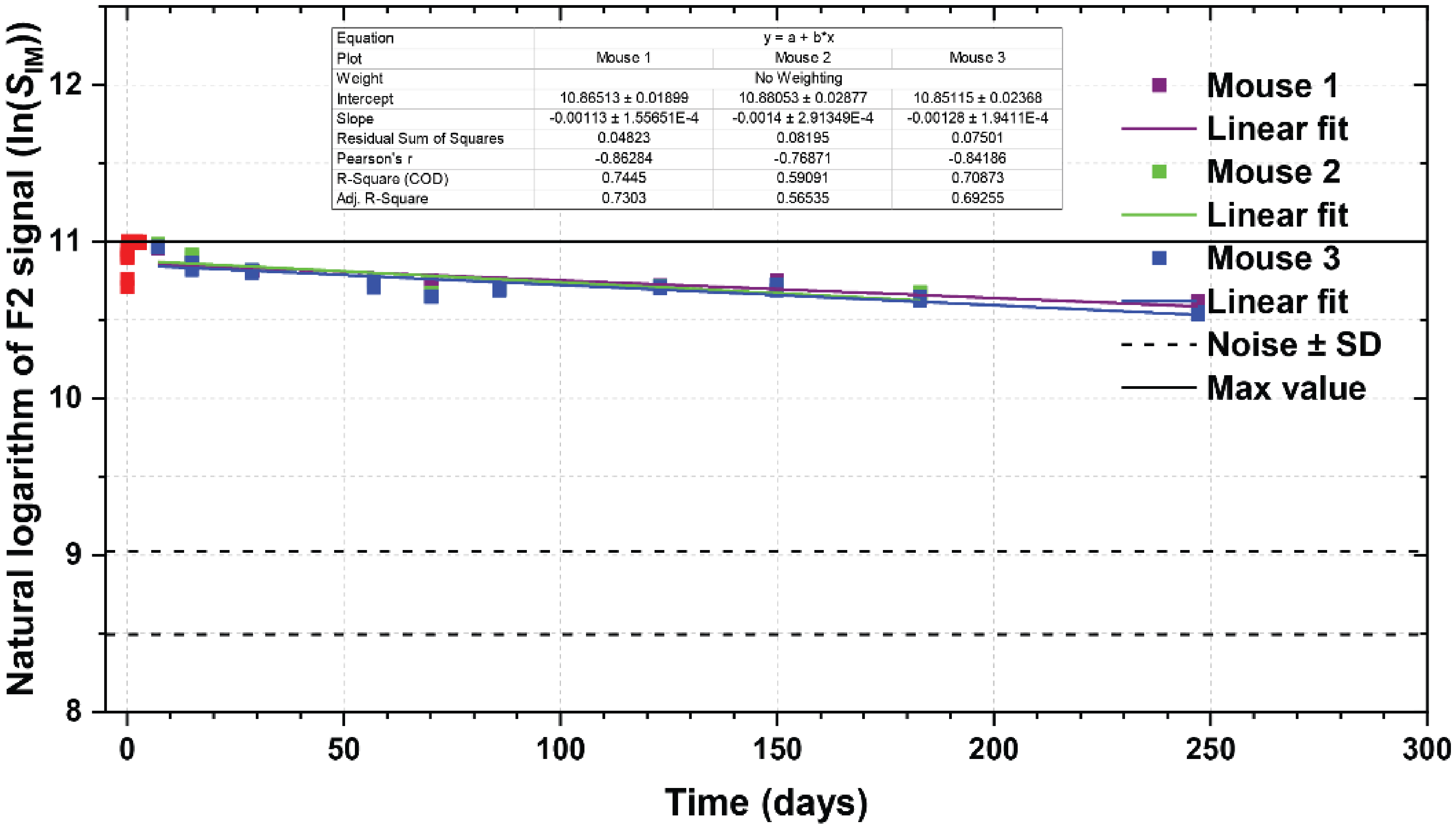

Figure S74. Depot size of F2 (pixels) in mice M1, M2, and M3 as a function of time. **Phase 1a** and **Phase 3a** are marked in red.

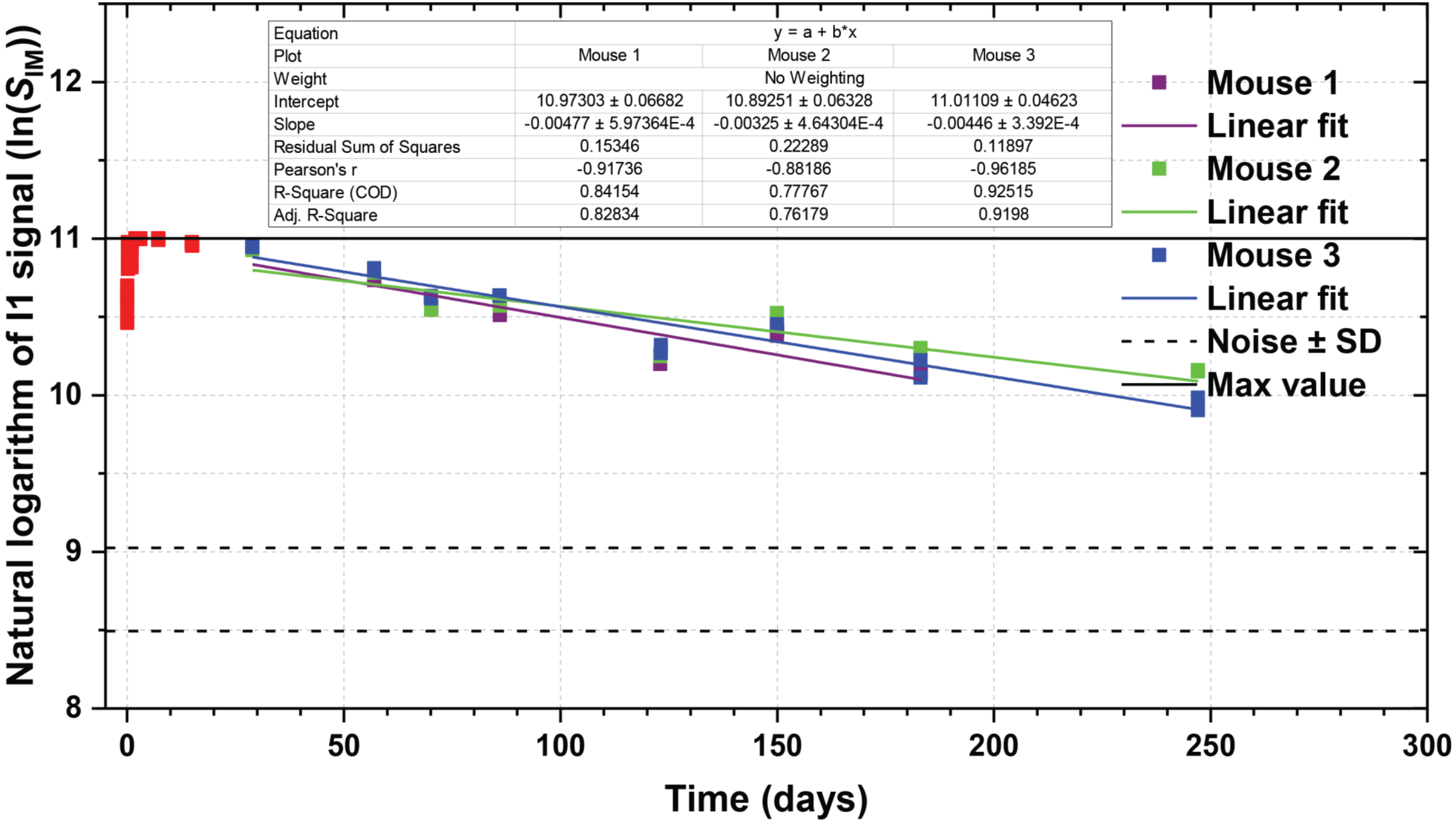

Figure S75. Depot size of **I1** (pixels) in mice M1, M2, and M3 as a function of time. **Phase 1a** and **Phase 3a** are marked in red.

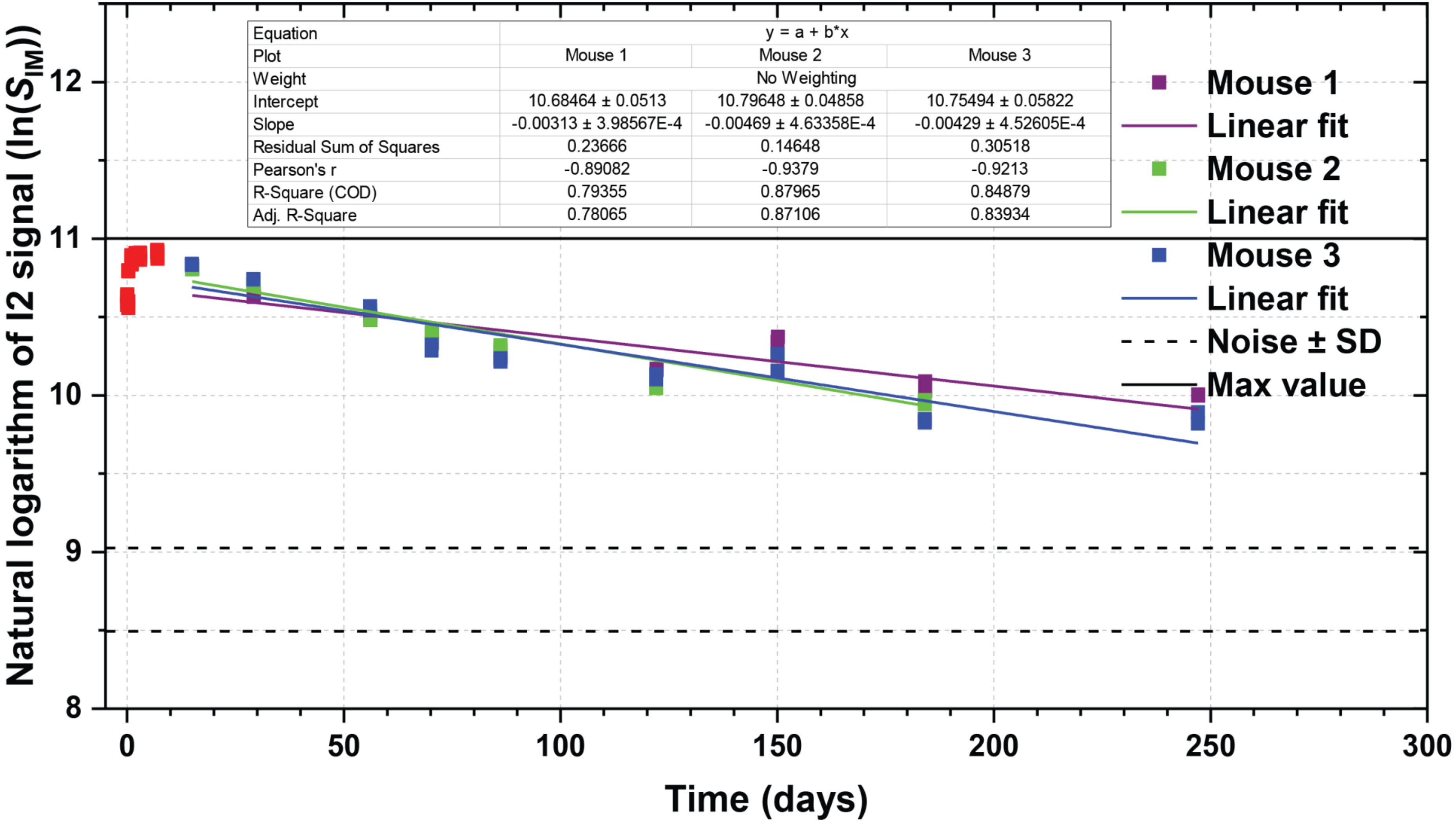

Figure S76. Depot size of **I2** (pixels) in mice M1, M2, and M3 as a function of time. **Phase 1a** and **Phase 3a** are marked in red.

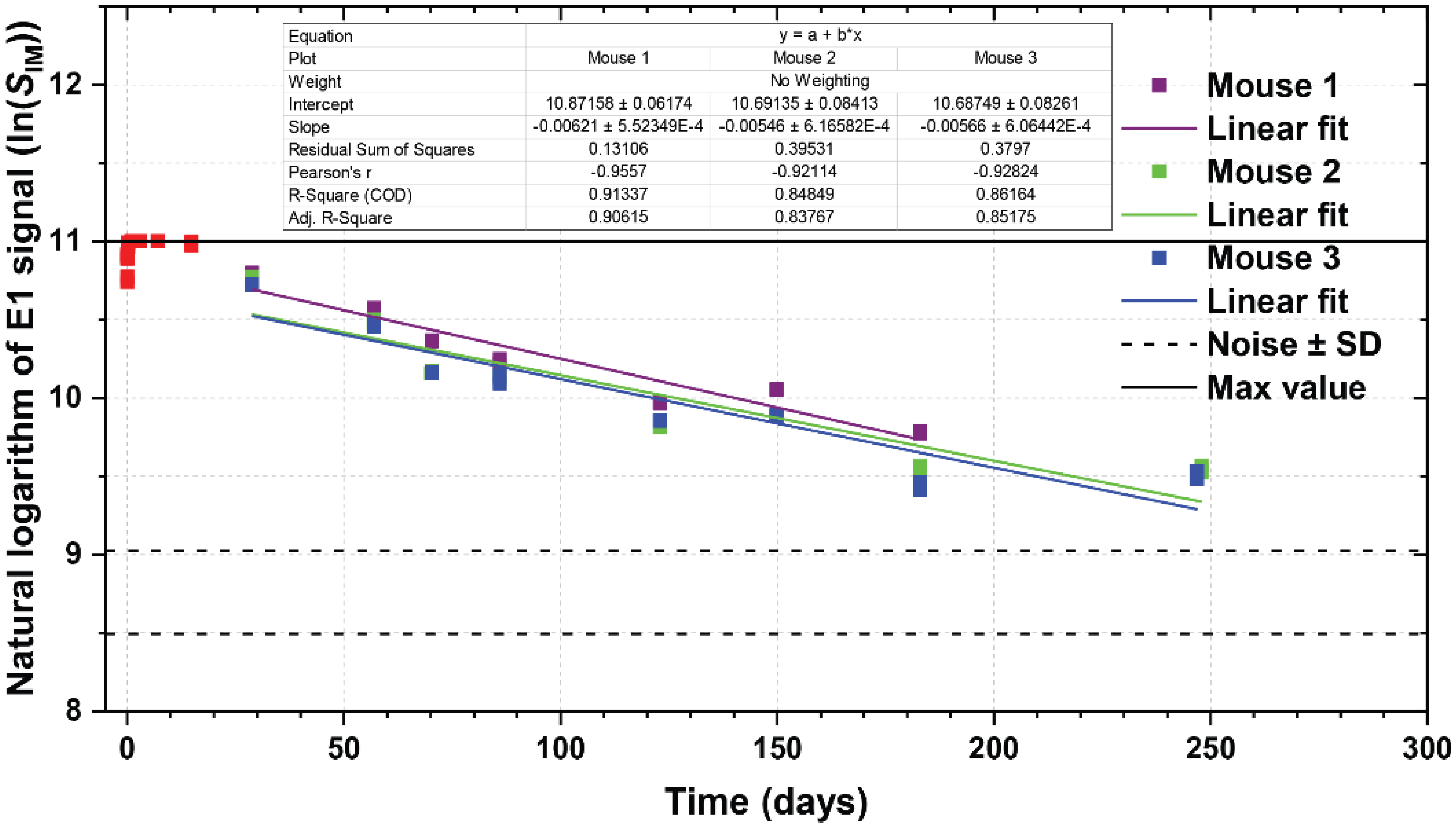

Figure S77. Depot size of E1 (pixels) in mice M1, M2, and M3 as a function of time. **Phase 1a** and **Phase 3a** are marked in red.

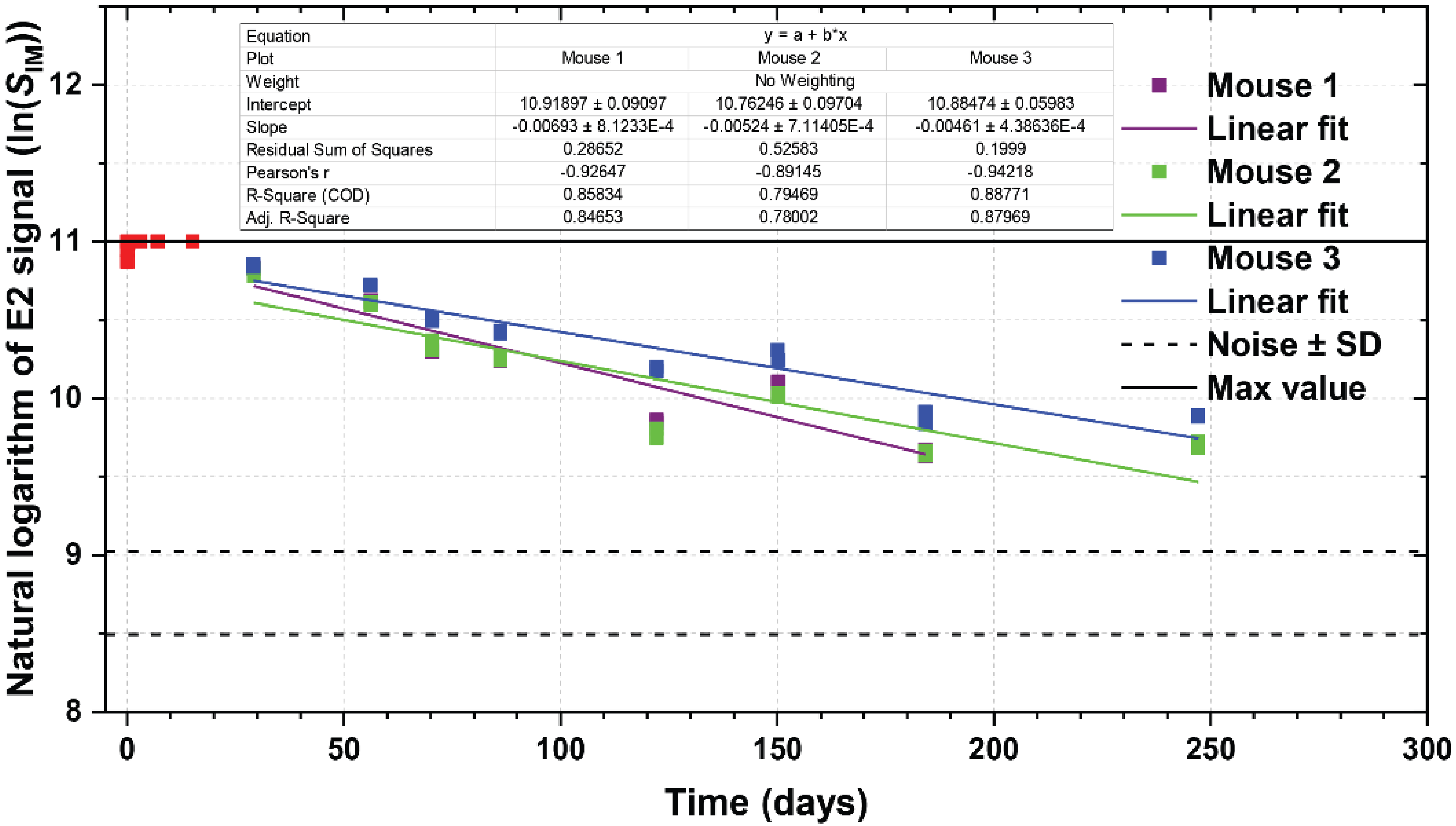

Equation	y = a + b*x		
Plot	Mouse 1	Mouse 2	Mouse 3
Weight	No Weighting		
Intercept	10.91897 ± 0.09097	10.76246 ± 0.09704	10.88474 ± 0.05983
Slope	-0.00693 ± 8.1233E-4	-0.00524 ± 7.11405E-4	-0.00461 ± 4.38636E-4
Residual Sum of Squares	0.28652	0.52583	0.1999
Pearson's r	-0.92647	-0.89145	-0.94218
R-Square (COD)	0.85834	0.79469	0.88771
Adj. R-Square	0.84653	0.78002	0.87969

Figure S78. Depot size of E2 (pixels) in mice M1, M2, and M3 as a function of time. **Phase 1a** and **Phase 3a** are marked in red.

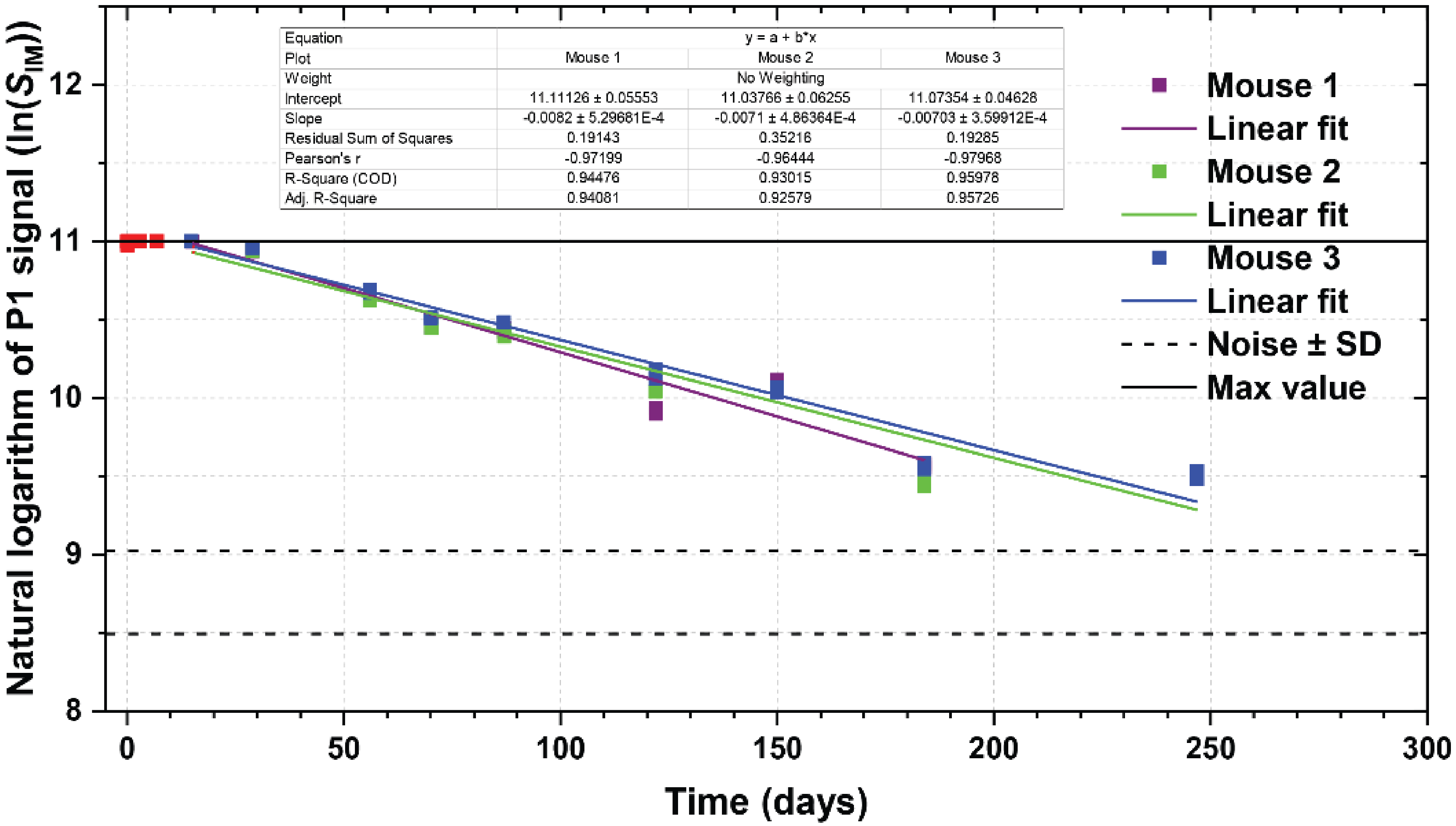

Figure S79. Depot size of **P1** (pixels) in mice M1, M2, and M3 as a function of time. **Phase 1a** and **Phase 3a** are marked in red.

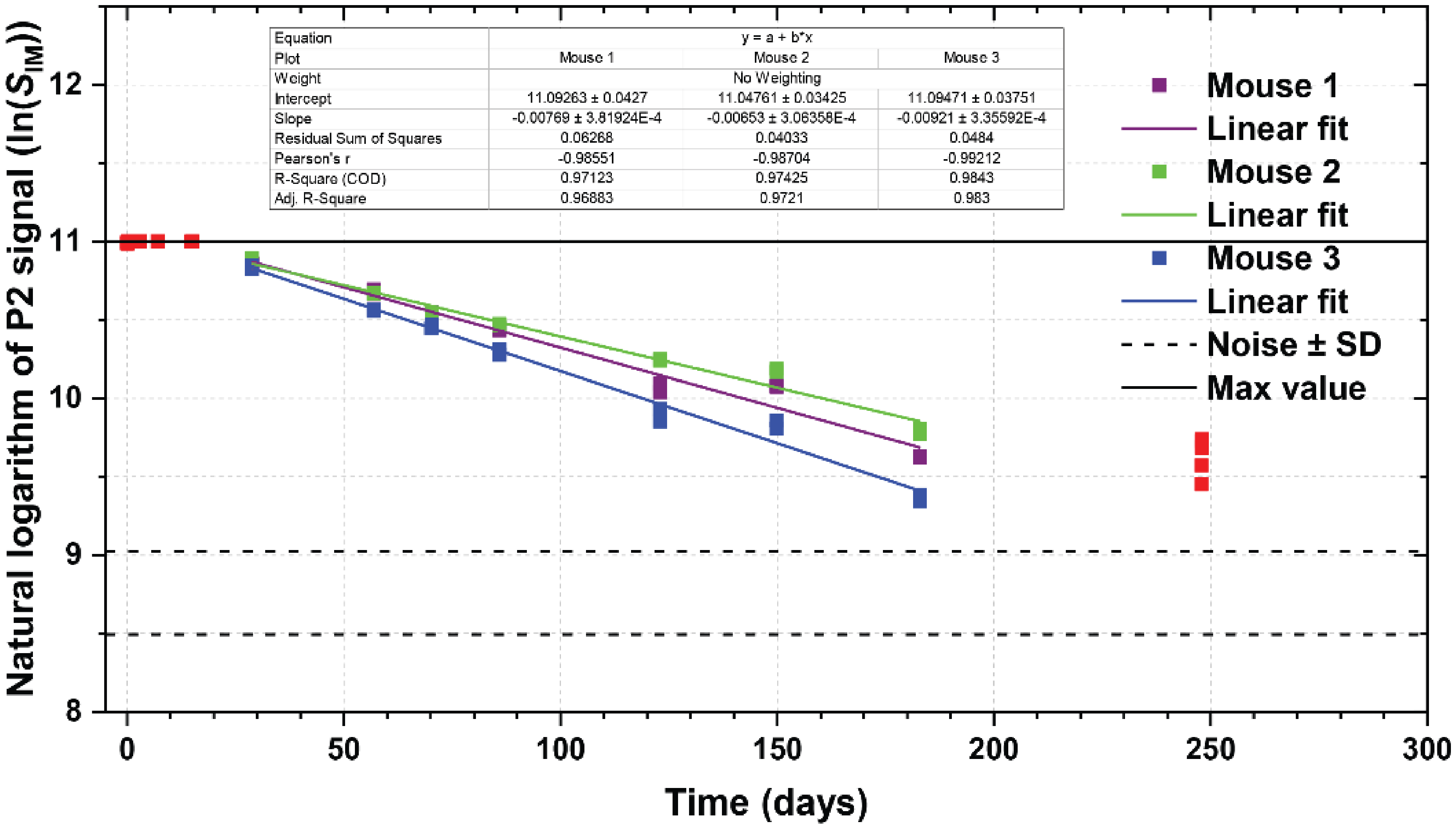

Equation	y = a + b*x		
Plot	Mouse 1	Mouse 2	Mouse 3
Weight	No Weighting		
Intercept	11.09263 ± 0.0427	11.04761 ± 0.03425	11.09471 ± 0.03751
Slope	-0.00769 ± 3.81924E-4	-0.00653 ± 3.06358E-4	-0.00921 ± 3.35592E-4
Residual Sum of Squares	0.06268	0.04033	0.0484
Pearson's r	-0.98551	-0.98704	-0.99212
R-Square (COD)	0.97123	0.97425	0.9843
Adj. R-Square	0.96883	0.9721	0.983

Figure S80. Depot size of **P2** (pixels) in mice M1, M2, and M3 as a function of time. **Phase 1a** and **Phase 3a** are marked in red.

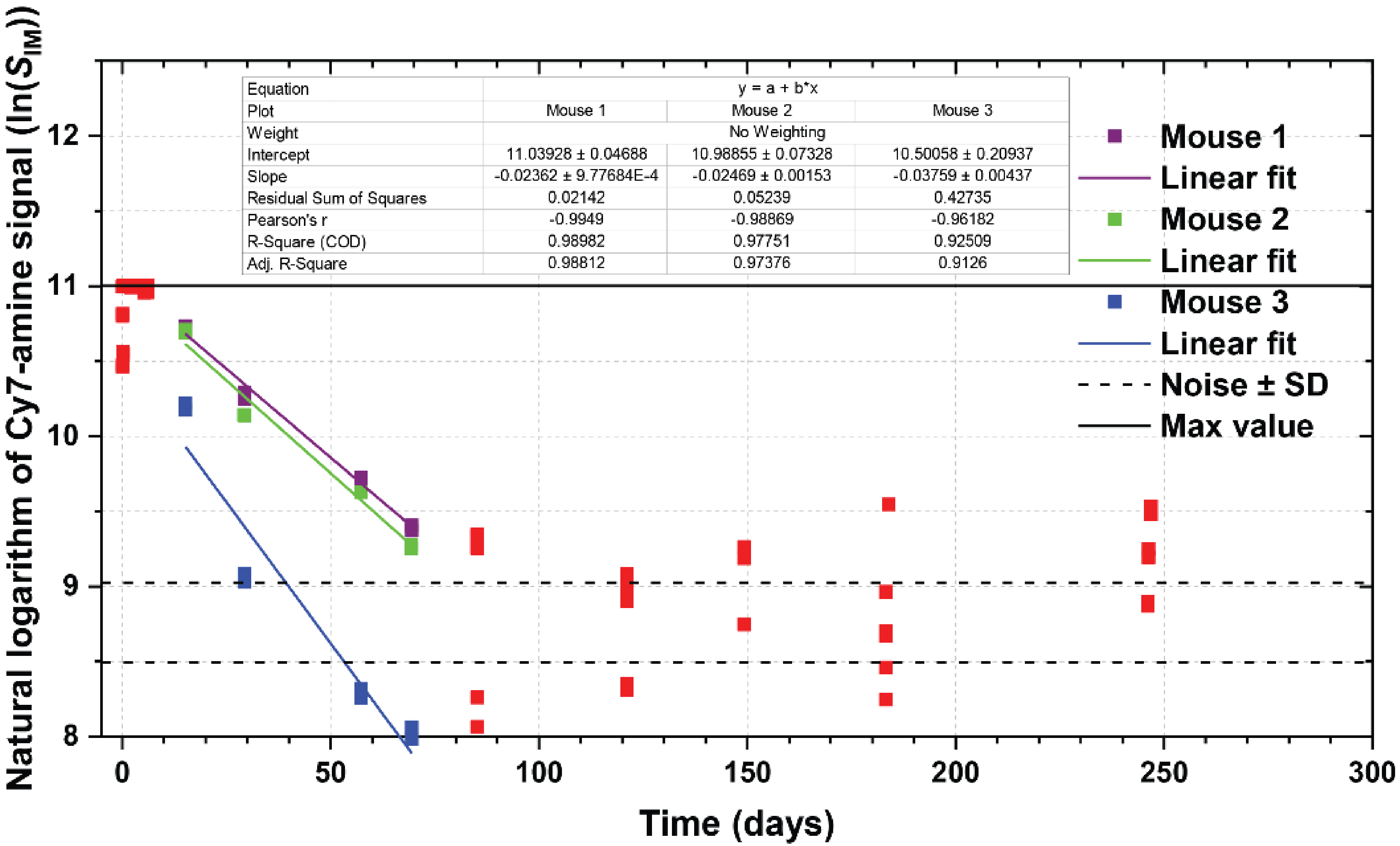

Figure S81. Depot size of **Cy7-amine** (pixels) in mice M1, M2, and M3 as a function of time. **Phase 1a** and **Phase 3a** are marked in red.

 DOI: XXXX.YYYY/ZZZZ

S12.2.3. Intramuscular depot dissolution data – distribution index (K_{10})

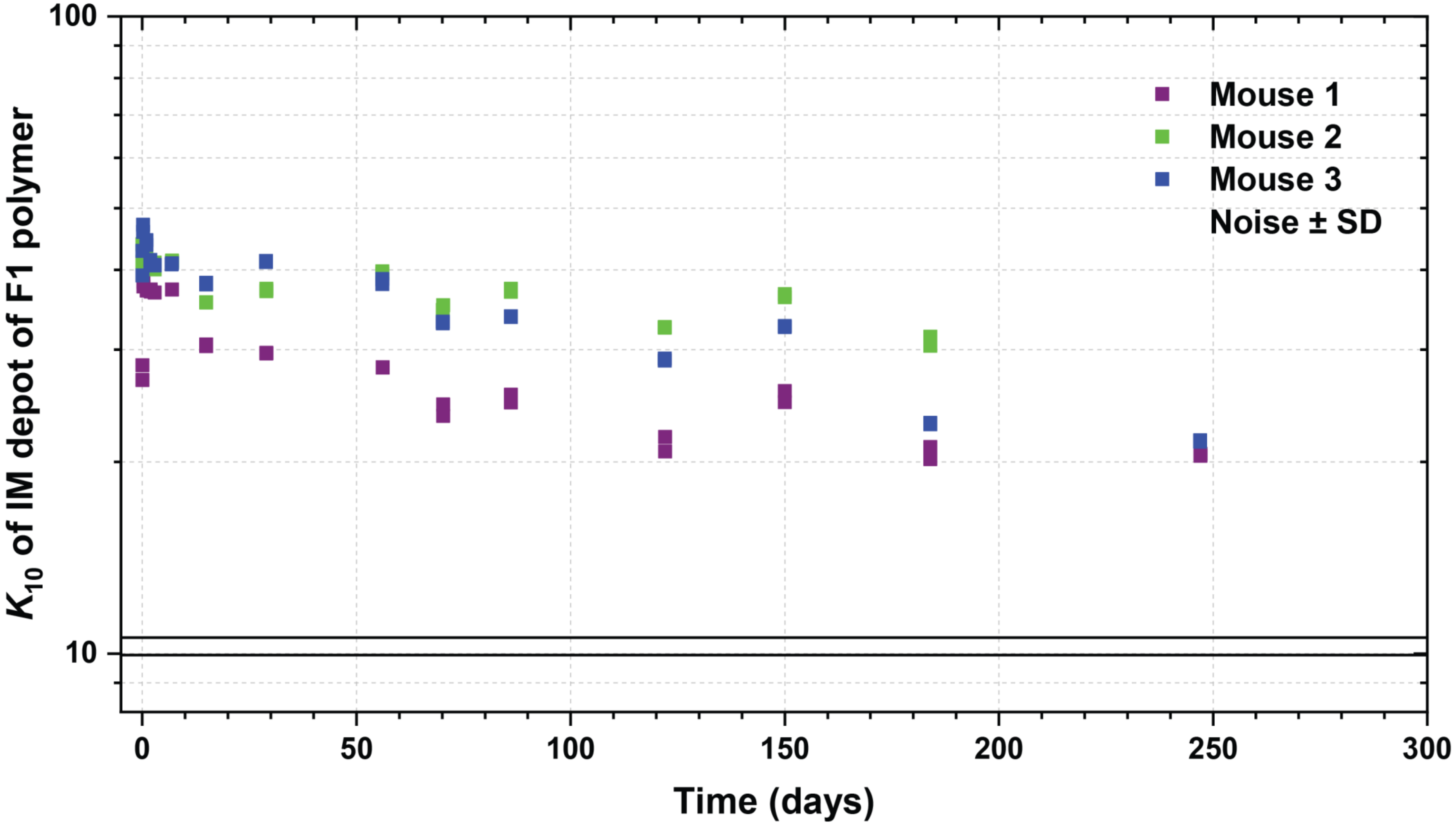

Figure S82. Distribution index (K_{10}) of **F1** in mice M1, M2, and M3 as a function of time. **Phase 1a** and **Phase 3a** are marked in red.

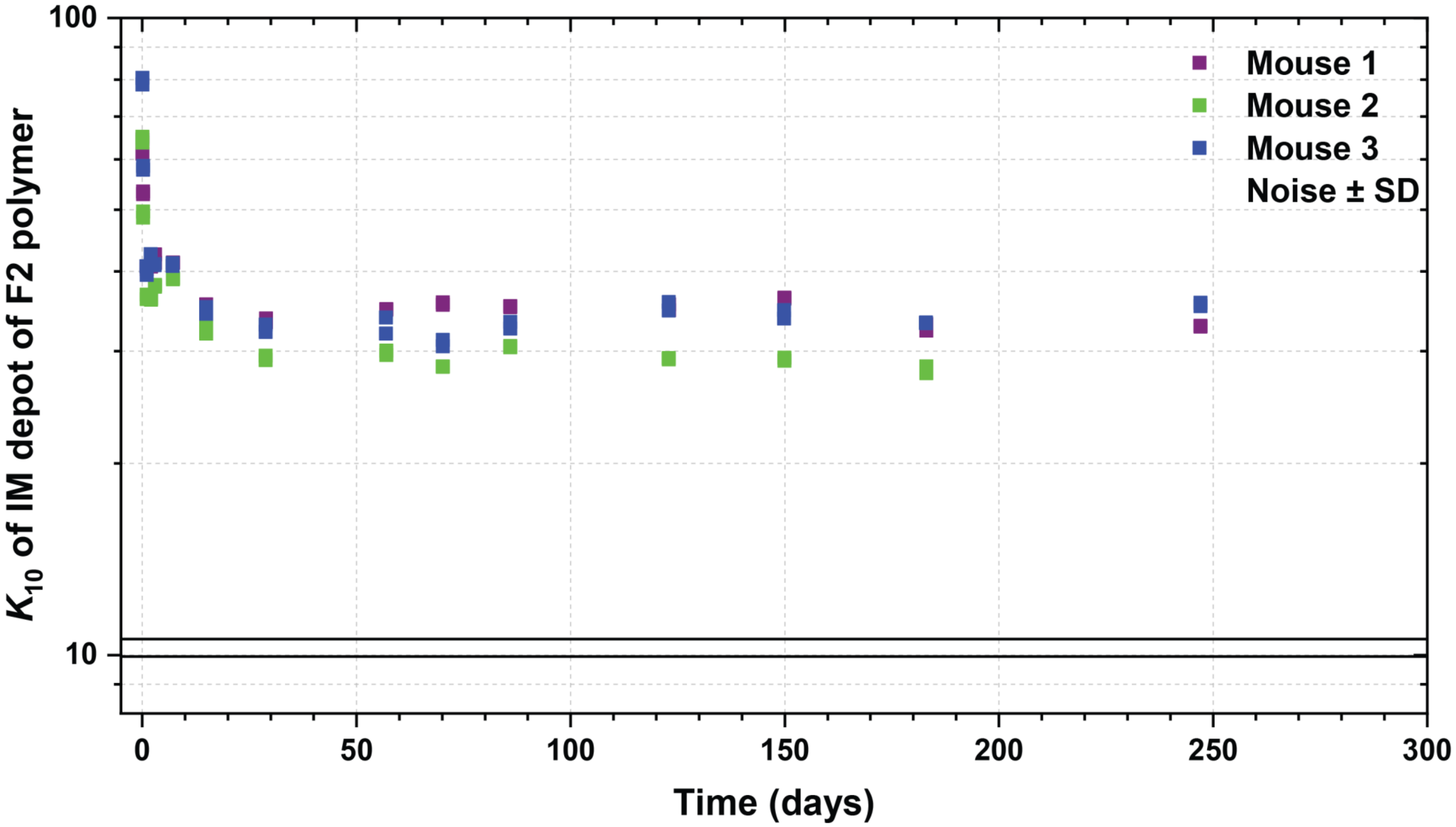

Figure S83. Distribution index (K_{10}) of **F2** in mice M1, M2, and M3 as a function of time. **Phase 1a** and **Phase 3a** are marked in red.

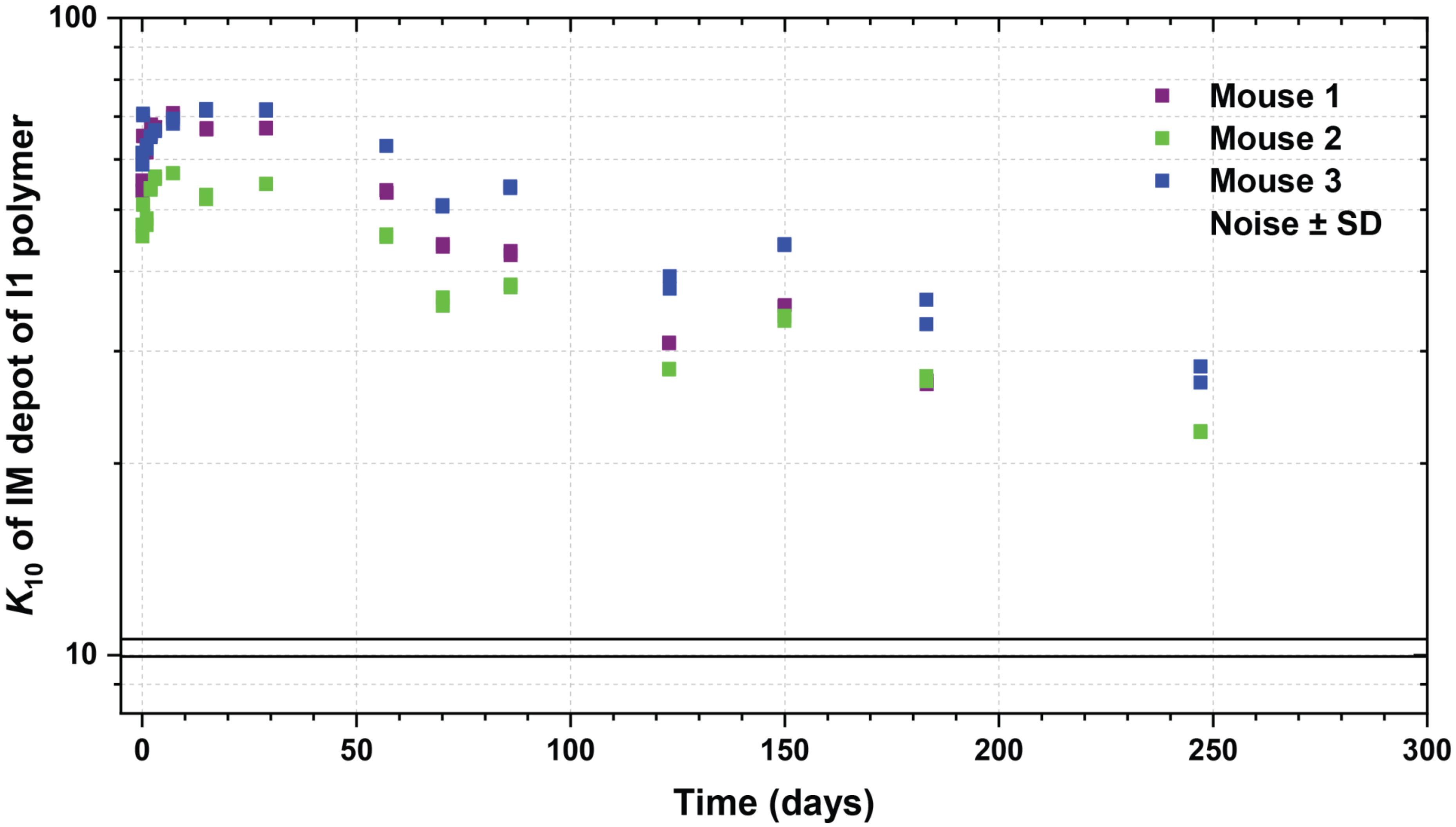

Figure S84. Distribution index (K_{10}) of **I1** in mice M1, M2, and M3 as a function of time. **Phase 1a** and **Phase 3a** are marked in red.

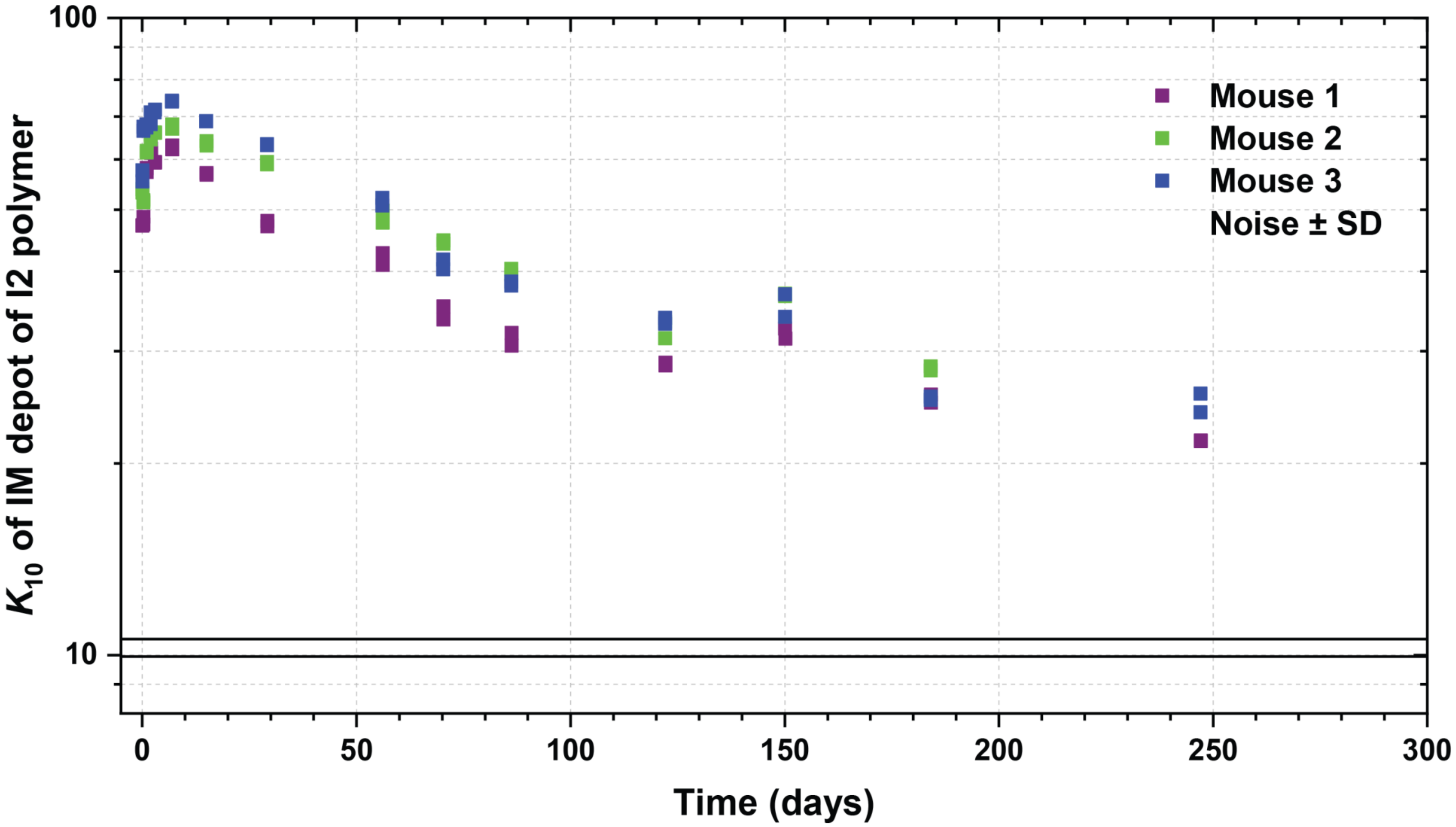

Figure S85. Distribution index (K_{10}) of I2 in mice M1, M2, and M3 as a function of time. **Phase 1a** and **Phase 3a** are marked in red.

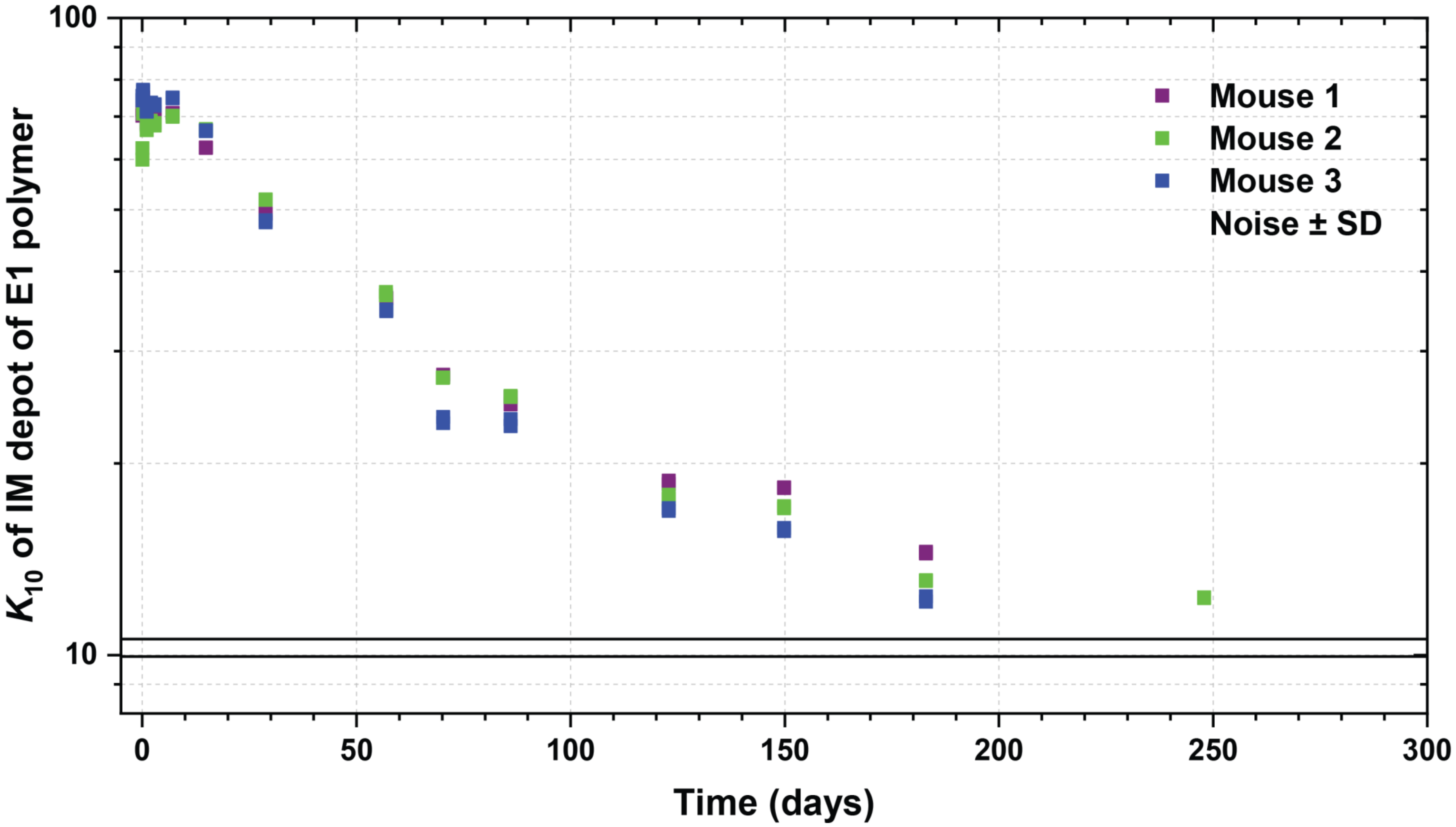

Figure S86. Distribution index (K_{10}) of **E1** in mice M1, M2, and M3 as a function of time. **Phase 1a** and **Phase 3a** are marked in red.

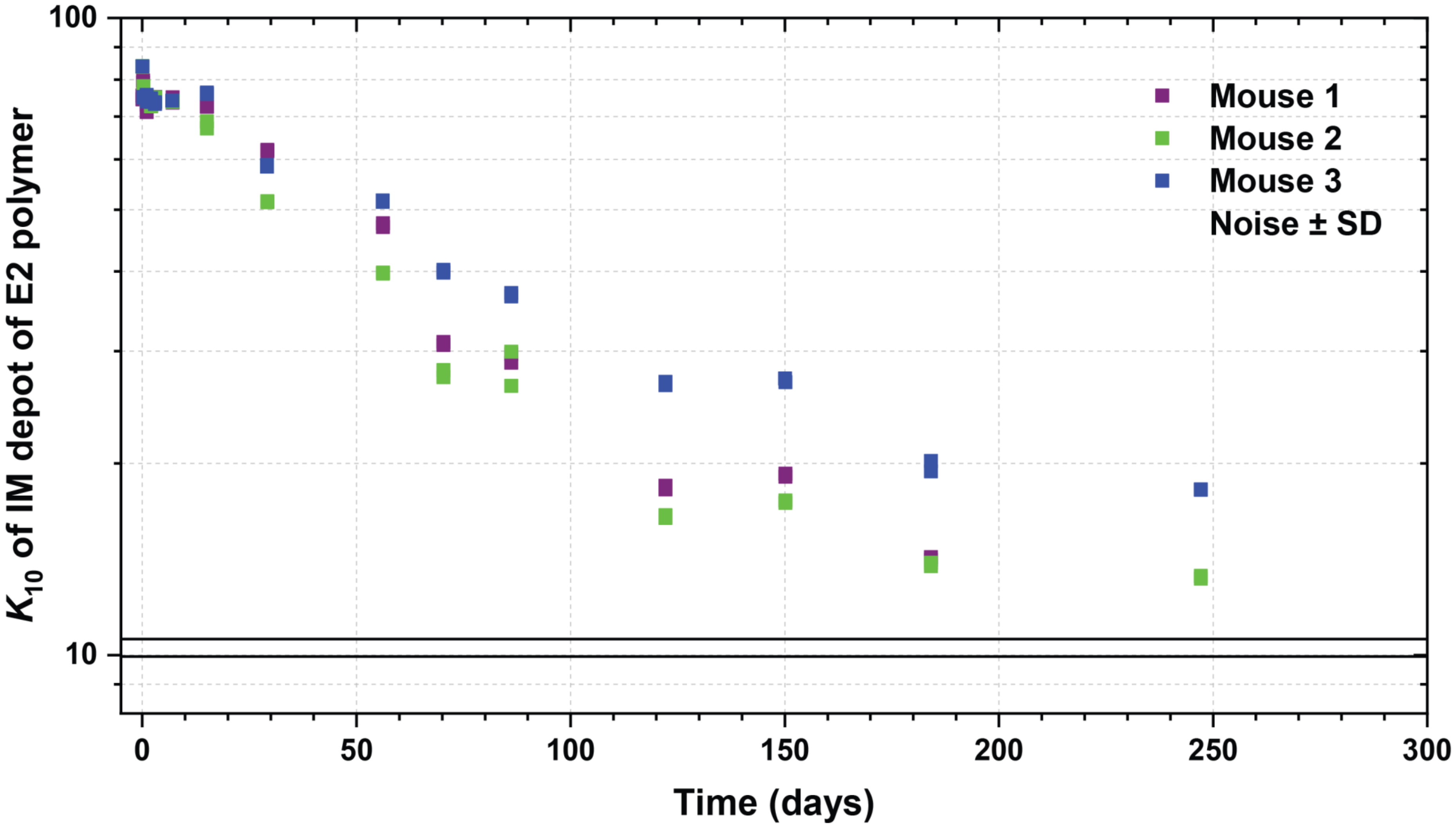

Figure S87. Distribution index (K_{10}) of E2 in mice M1, M2, and M3 as a function of time. **Phase 1a** and **Phase 3a** are marked in red.

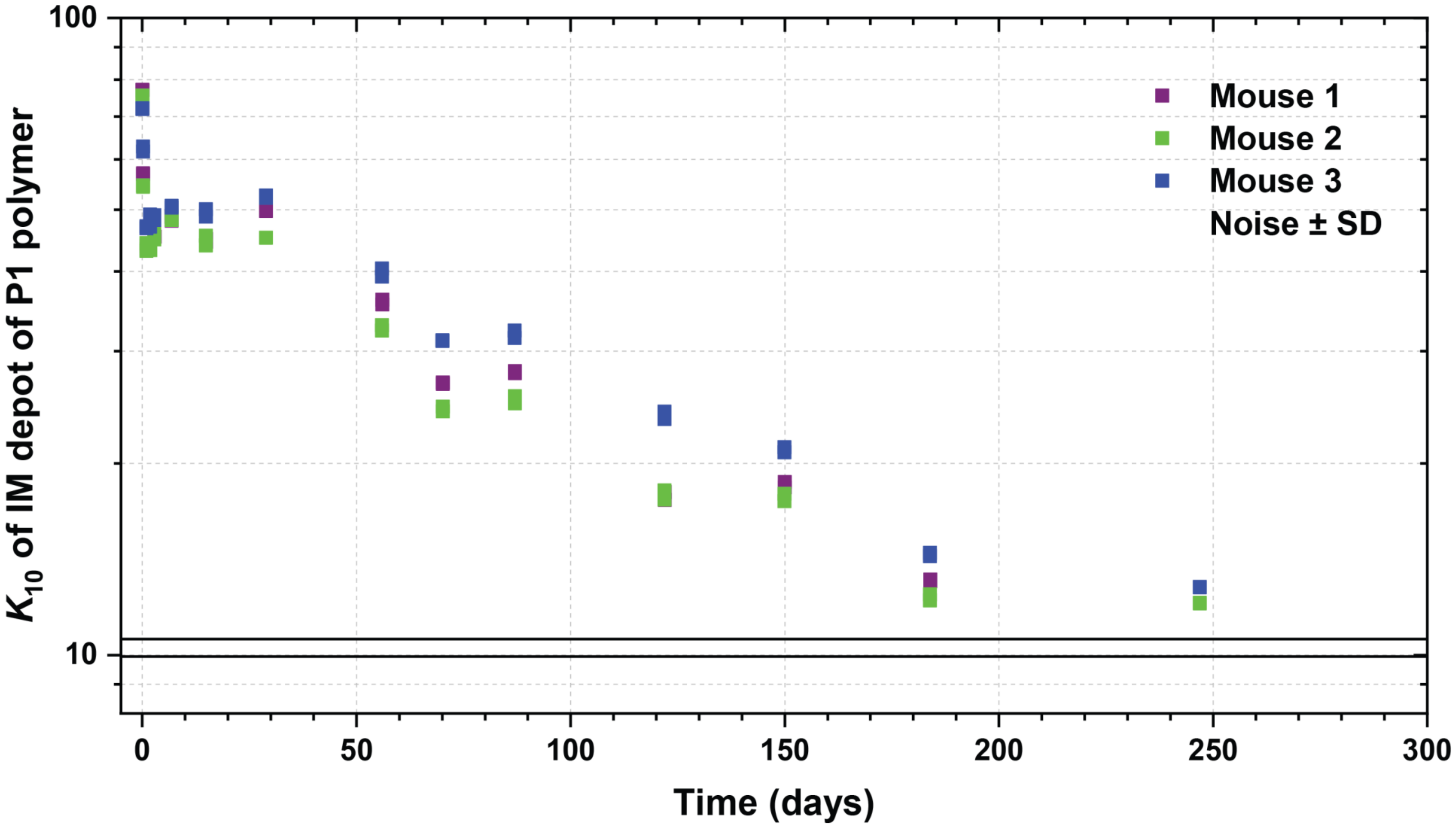

Figure S88. Distribution index (K_{10}) of **P1** in mice M1, M2, and M3 as a function of time. **Phase 1a** and **Phase 3a** are marked in red.

DOI: XXXX.YYYY/ZZZZ

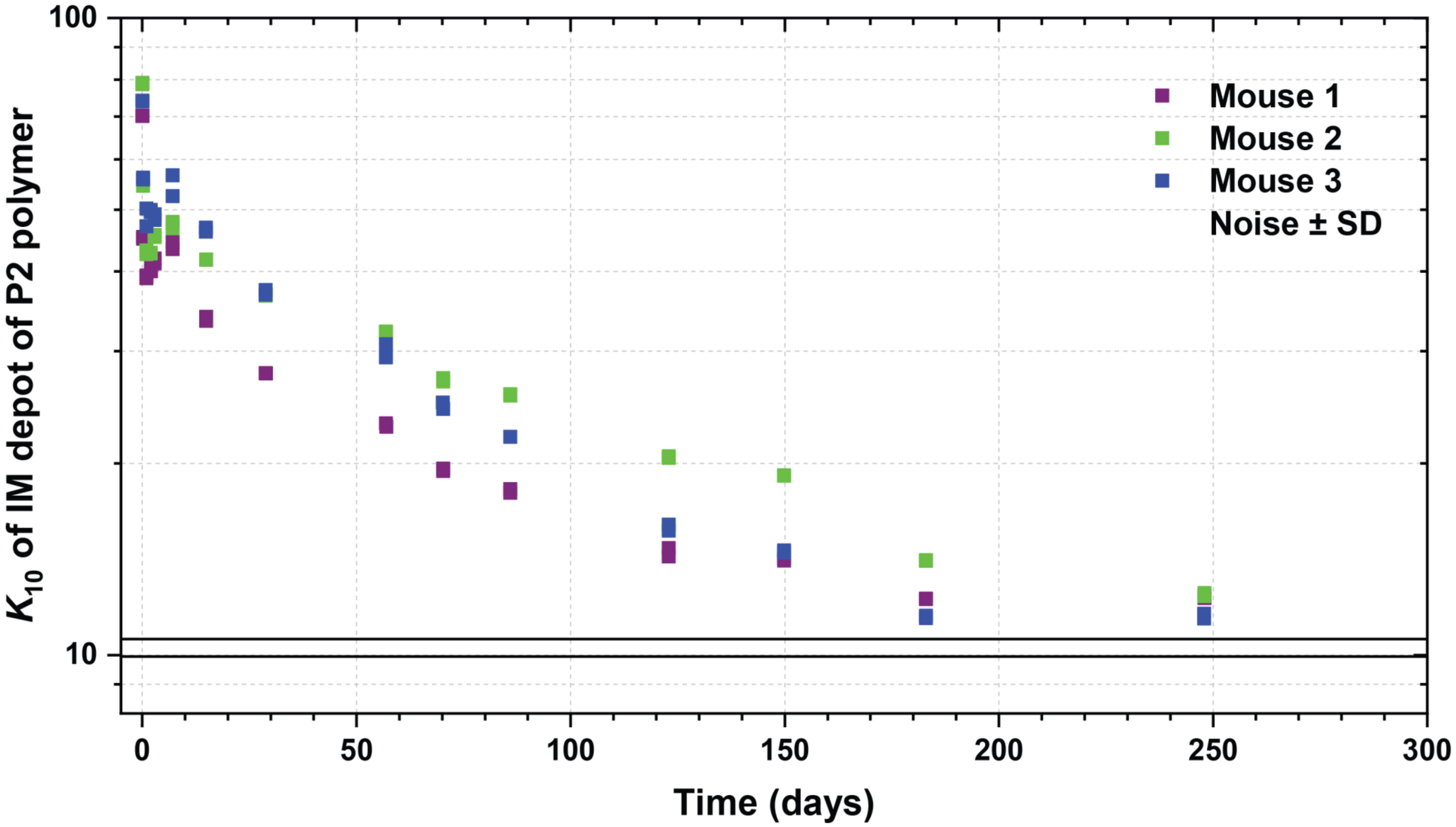

Figure S89. Distribution index (K_{10}) of **P2** in mice M1, M2, and M3 as a function of time. **Phase 1a** and **Phase 3a** are marked in red.

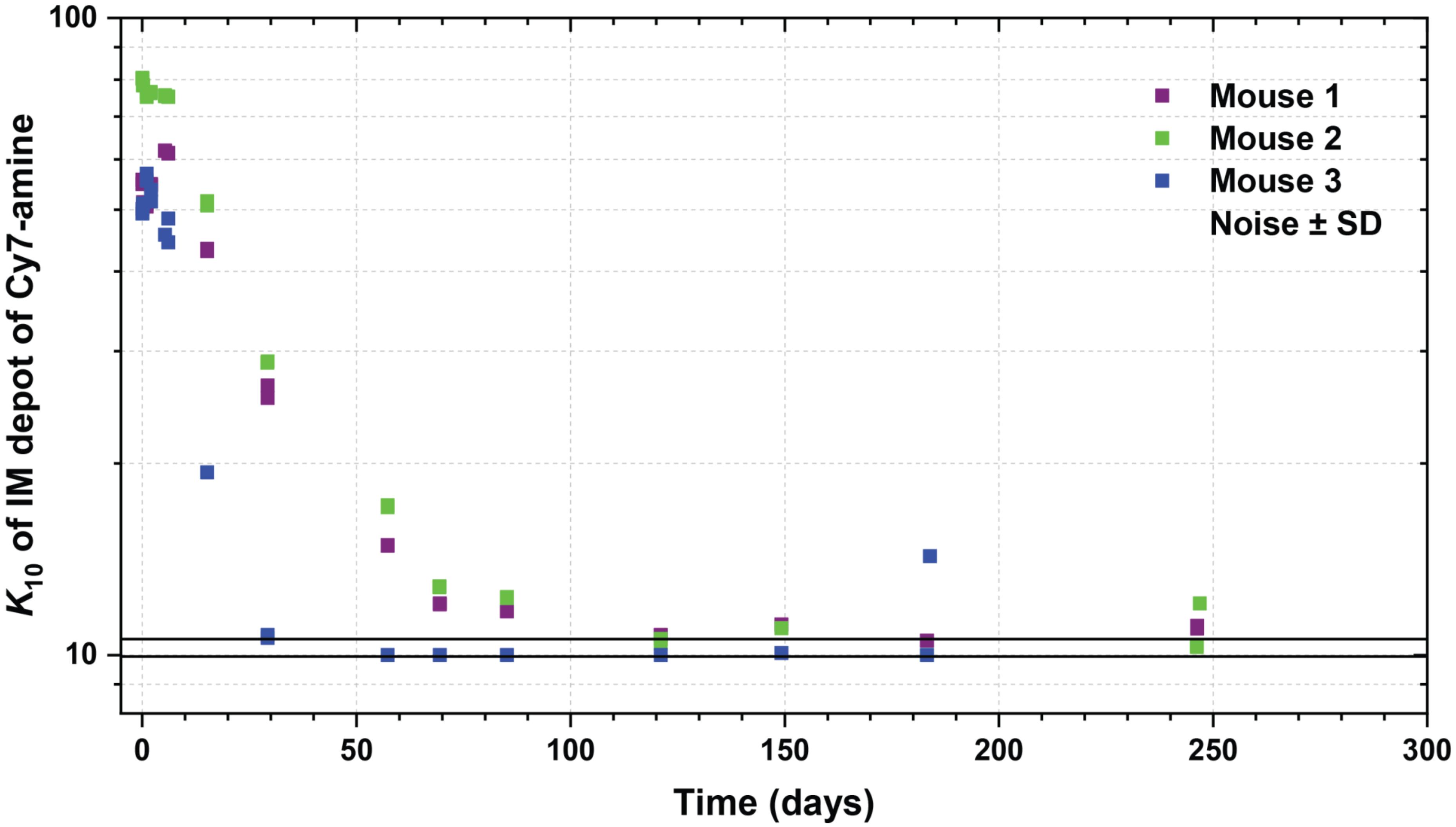

Figure S90. Distribution index (K_{10}) of **Cy7-amine** in mice M1, M2, and M3 as a function of time. **Phase 1a** and **Phase 3a** are marked in red.

S12.2.4. Kidney depot dissolution – signal as a function of time

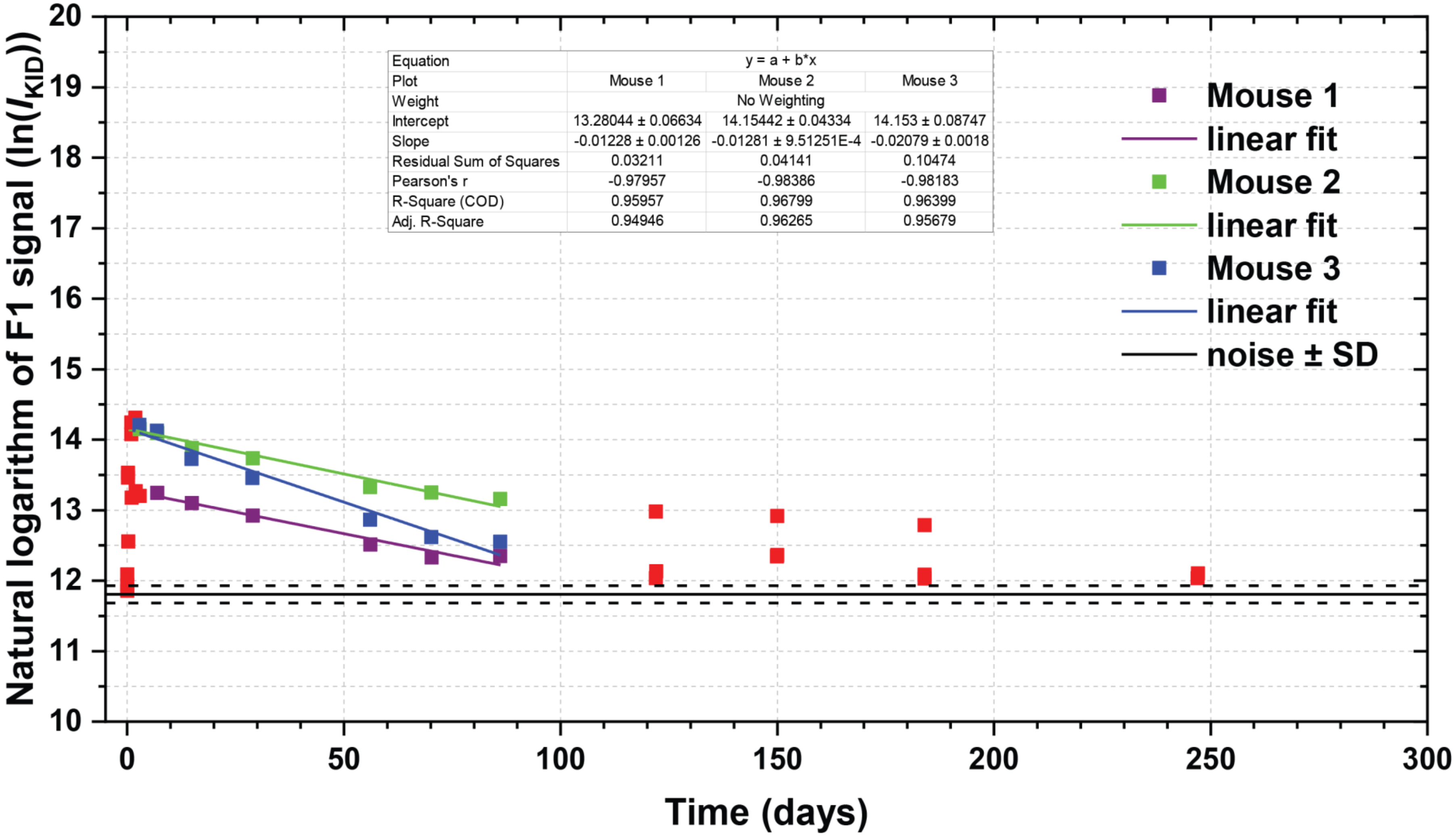

Figure S91. Signal of **F1** (arbitrary units) in **kidney** depot in mice M1, M2, and M3 as a function of time. **Phase 1b**, **3b** and **4b** are marked in red.

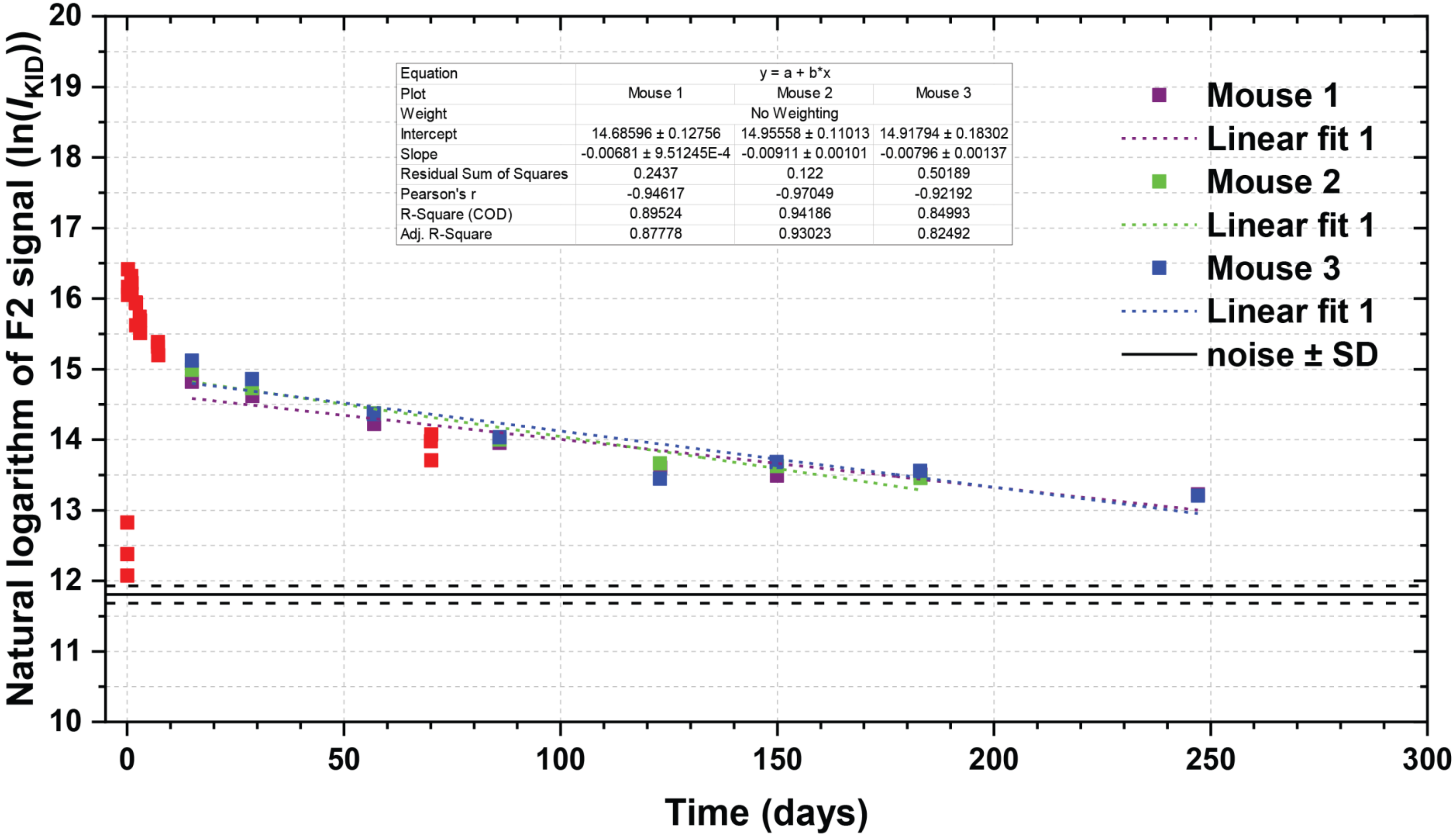

Figure S92. Signal of F2 (arbitrary units) in **kidney** depot in mice M1, M2, and M3 as a function of time. **Phase 1b** are marked in red. In this case, we determined both the kinetics of both **Phase 2b** and **3b**.

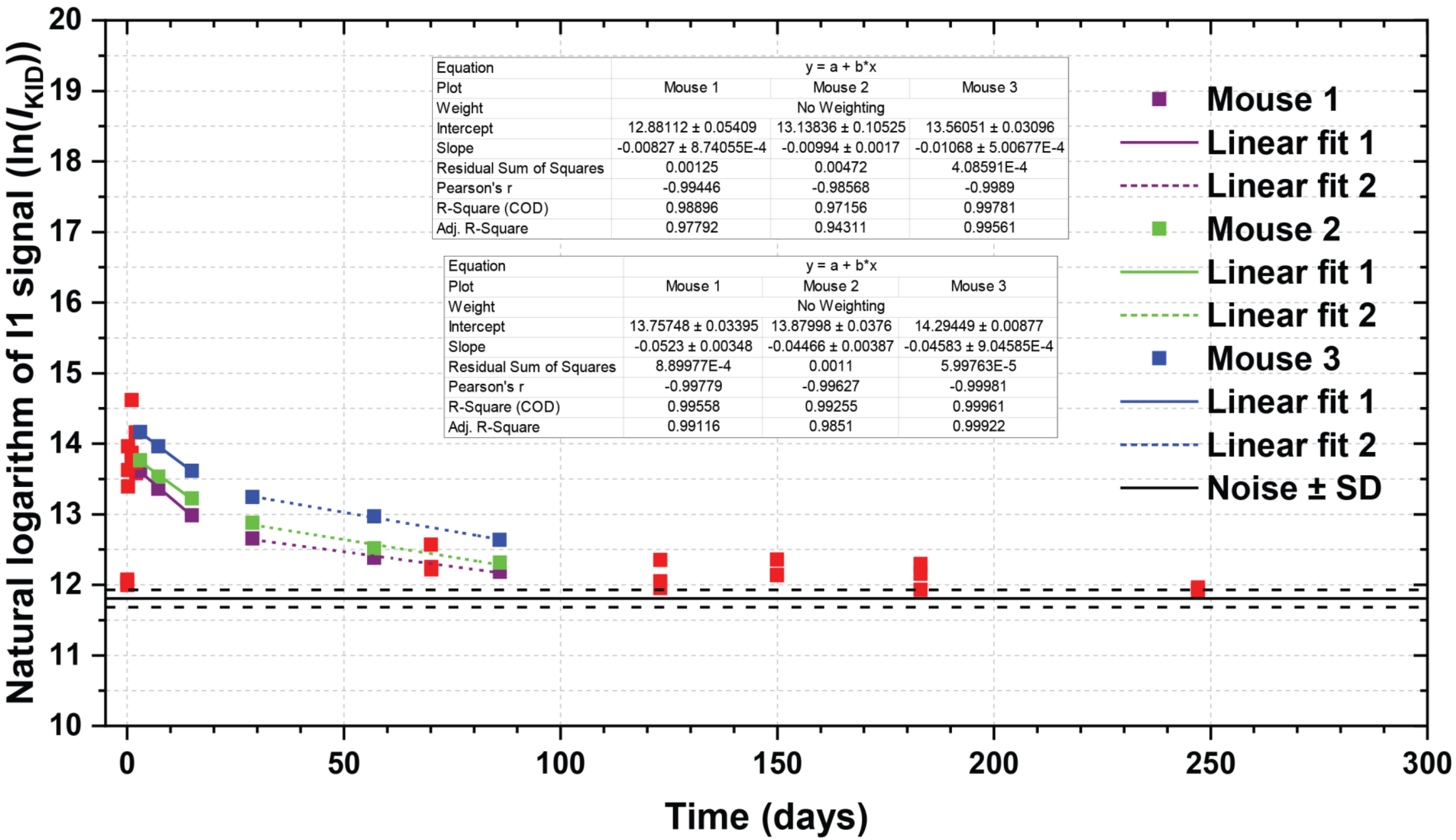

Figure S93. Signal of **I1** (arbitrary units) in **kidney** depot in mice M1, M2, and M3 as a function of time. **Phase 1b** are marked in red. In this case, we determined both the kinetics of both **Phase 2b** and **3b**.

DOI: XXXX.YYYY/ZZZZ

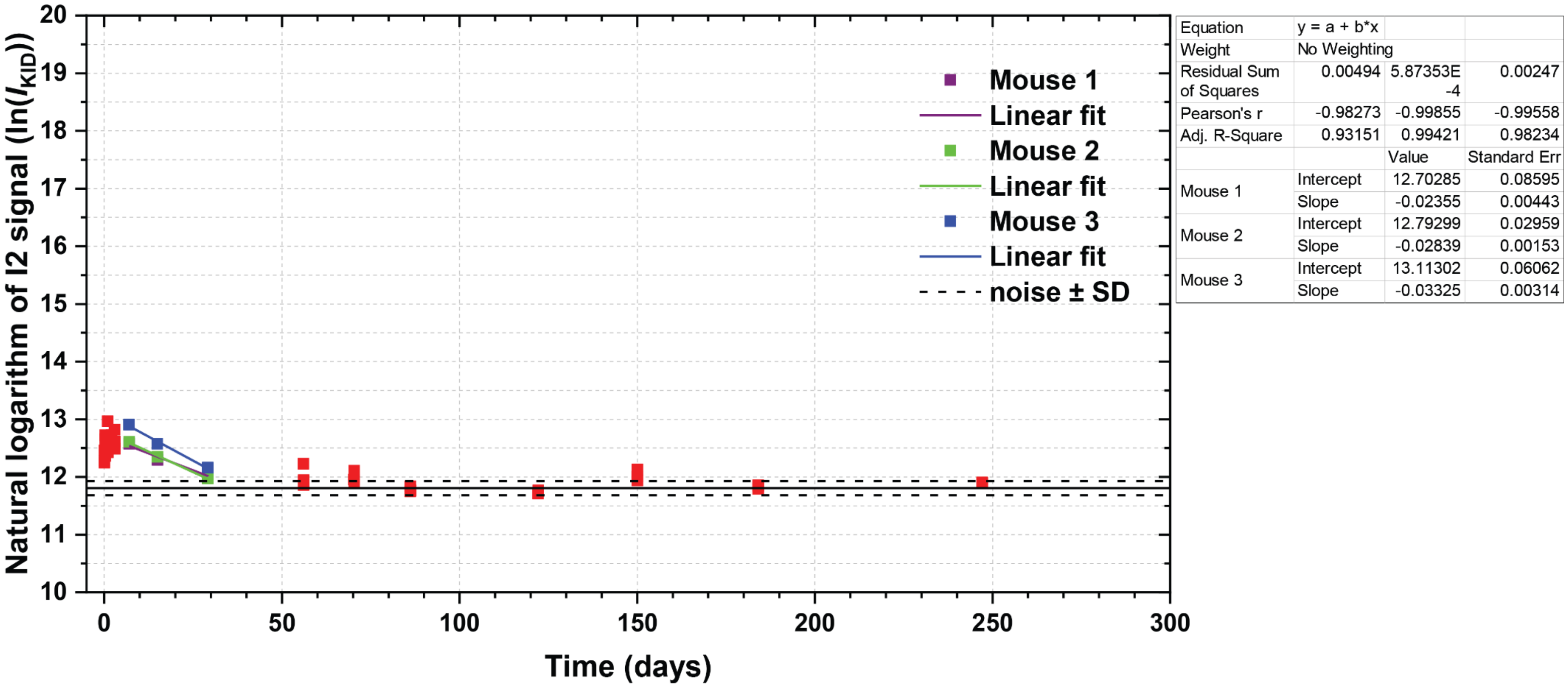

Equation	y = a + b*x		
Weight	No Weighting		
Residual Sum of Squares	0.00494	5.87353E-4	0.00247
Pearson's r	-0.98273	-0.99855	-0.99558
Adj. R-Square	0.93151	0.99421	0.98234
		Value	Standard Err
Mouse 1	Intercept	12.70285	0.08595
	Slope	-0.02355	0.00443
Mouse 2	Intercept	12.79299	0.02959
	Slope	-0.02839	0.00153
Mouse 3	Intercept	13.11302	0.06062
	Slope	-0.03325	0.00314

Figure S94. Signal of I2 (arbitrary units) in **kidney** depot in mice M1, M2, and M3 as a function of time. **Phase 1b**, **3b** and **4b** are marked in red.

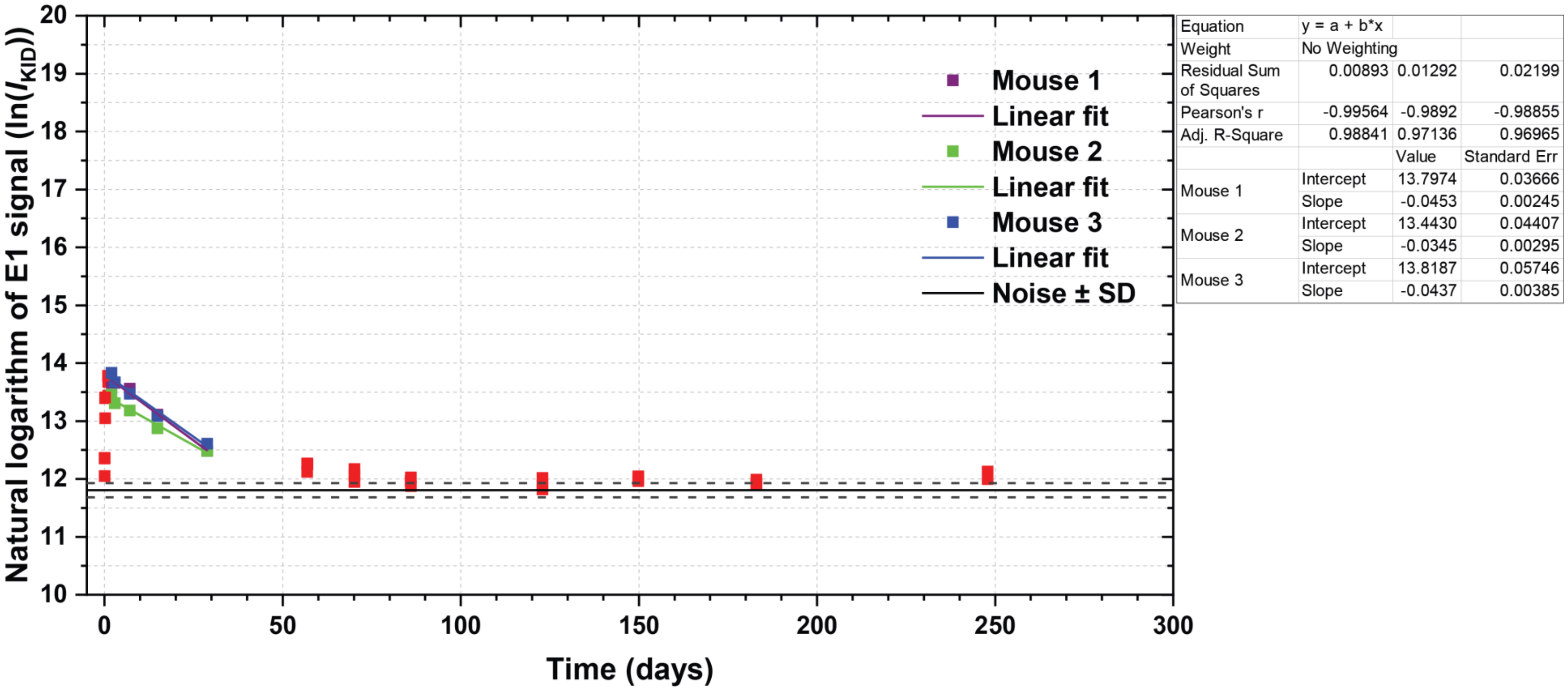

Equation	y = a + b*x		
Weight	No Weighting		
Residual Sum of Squares	0.00893	0.01292	0.02199
Pearson's r	-0.99564	-0.9892	-0.98855
Adj. R-Square	0.98841	0.97136	0.96965
		Value	Standard Err
Mouse 1	Intercept	13.7974	0.03666
	Slope	-0.0453	0.00245
Mouse 2	Intercept	13.4430	0.04407
	Slope	-0.0345	0.00295
Mouse 3	Intercept	13.8187	0.05746
	Slope	-0.0437	0.00385

Figure S95. Signal of E1 (arbitrary units) in **kidney** depot in mice M1, M2, and M3 as a function of time. **Phase 1b**, **3b** and **4b** are marked in red.

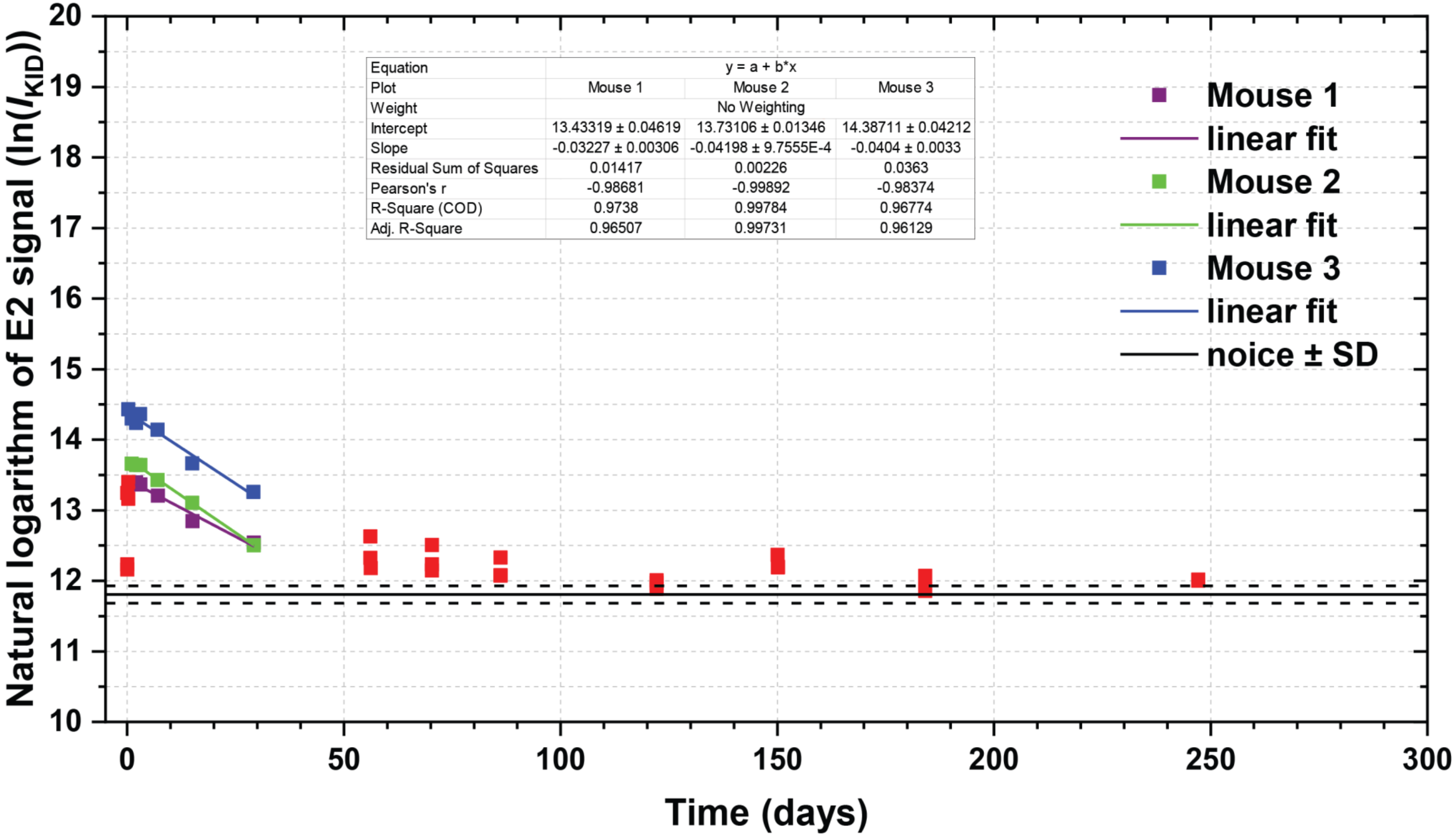

Figure S96. Signal of E2 (arbitrary units) in **kidney** depot in mice M1, M2, and M3 as a function of time. **Phase 1b**, **3b** and **4b** are marked in red.

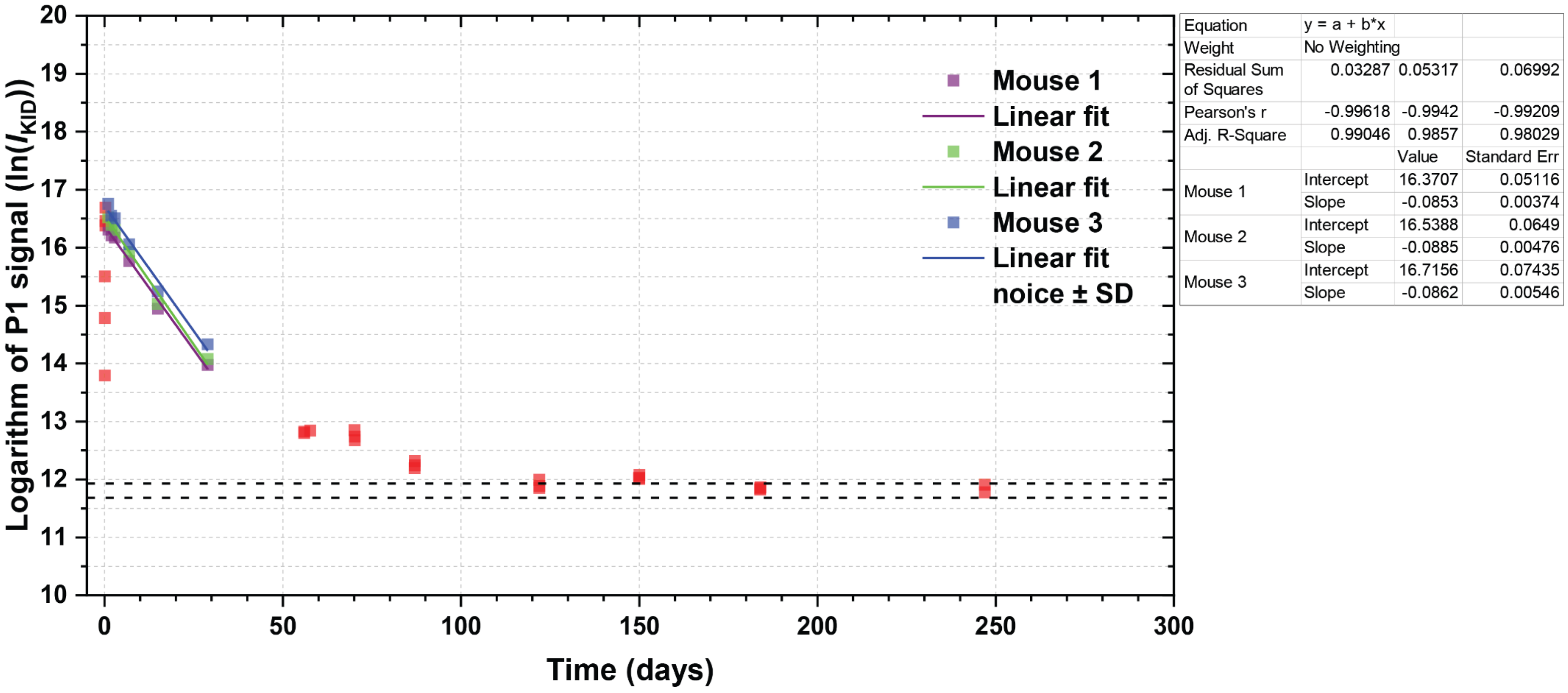

Equation	y = a + b*x		
Weight	No Weighting		
Residual Sum of Squares	0.03287	0.05317	0.06992
Pearson's r	-0.99618	-0.9942	-0.99209
Adj. R-Square	0.99046	0.9857	0.98029
		Value	Standard Err
Mouse 1	Intercept	16.3707	0.05116
	Slope	-0.0853	0.00374
Mouse 2	Intercept	16.5388	0.0649
	Slope	-0.0885	0.00476
Mouse 3	Intercept	16.7156	0.07435
	Slope	-0.0862	0.00546

Figure S97. Signal of **P1** (arbitrary units) in **kidney** depot in mice M1, M2, and M3 as a function of time. **Phase 1b**, **3b** and **4b** are marked in red.

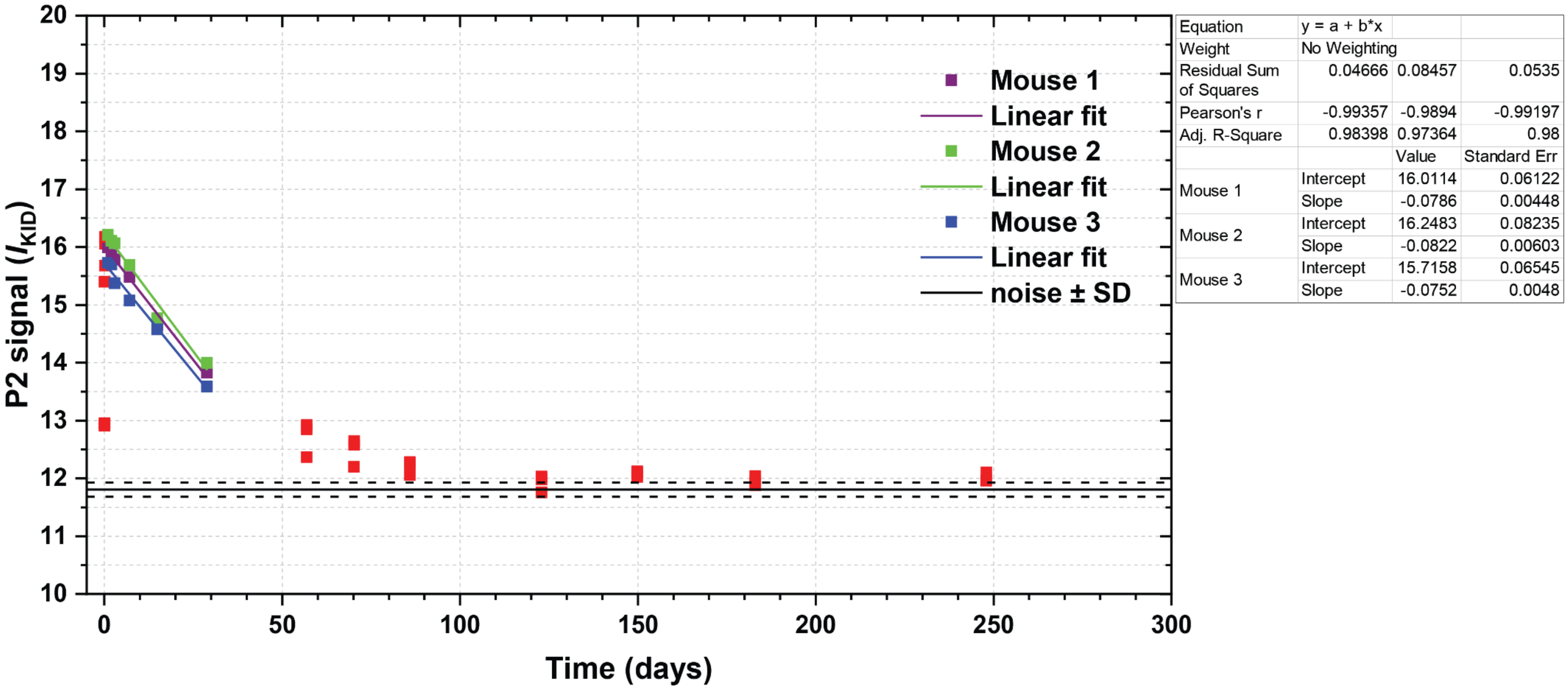

Equation	y = a + b*x		
Weight	No Weighting		
Residual Sum of Squares	0.04666	0.08457	0.0535
Pearson's r	-0.99357	-0.9894	-0.99197
Adj. R-Square	0.98398	0.97364	0.98
		Value	Standard Err
Mouse 1	Intercept	16.0114	0.06122
	Slope	-0.0786	0.00448
Mouse 2	Intercept	16.2483	0.08235
	Slope	-0.0822	0.00603
Mouse 3	Intercept	15.7158	0.06545
	Slope	-0.0752	0.0048

Figure S98. Signal of **P2** (arbitrary units) in **kidney** depot in mice M1, M2, and M3 as a function of time. **Phase 1b**, **3b** and **4b** are marked in red.

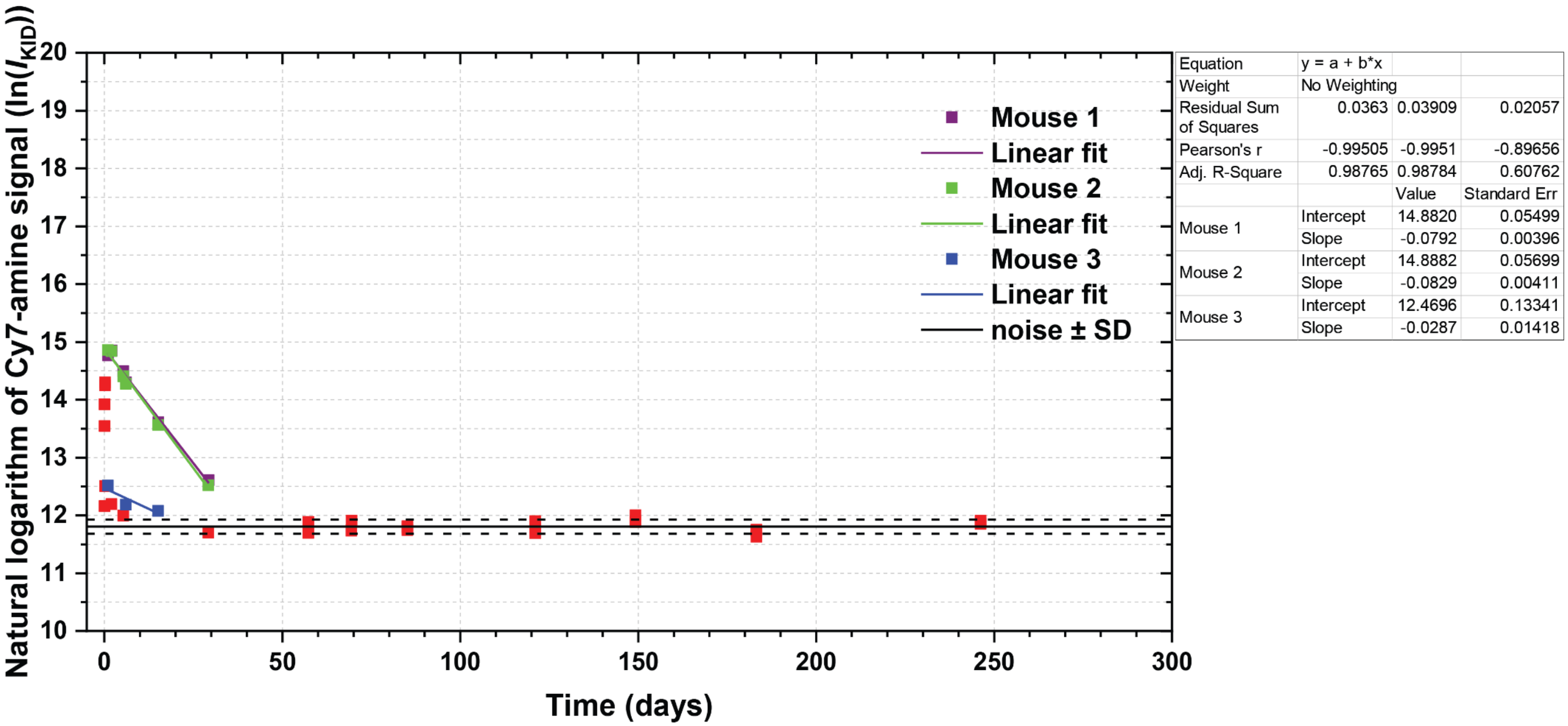

Equation	y = a + b*x		
Weight	No Weighting		
Residual Sum of Squares	0.0363	0.03909	0.02057
Pearson's r	-0.99505	-0.9951	-0.89656
Adj. R-Square	0.98765	0.98784	0.60762
		Value	Standard Err
Mouse 1	Intercept	14.8820	0.05499
	Slope	-0.0792	0.00396
Mouse 2	Intercept	14.8882	0.05699
	Slope	-0.0829	0.00411
Mouse 3	Intercept	12.4696	0.13341
	Slope	-0.0287	0.01418

Figure S99. Signal of **Cy7-amine** (arbitrary units) in **kidney** depot in mice M1, M2, and M3 as a function of time. **Phase 1b**, **3b** and **4b** are marked in red.

S12.2.5. Liver depot dissolution – signal as a function of time

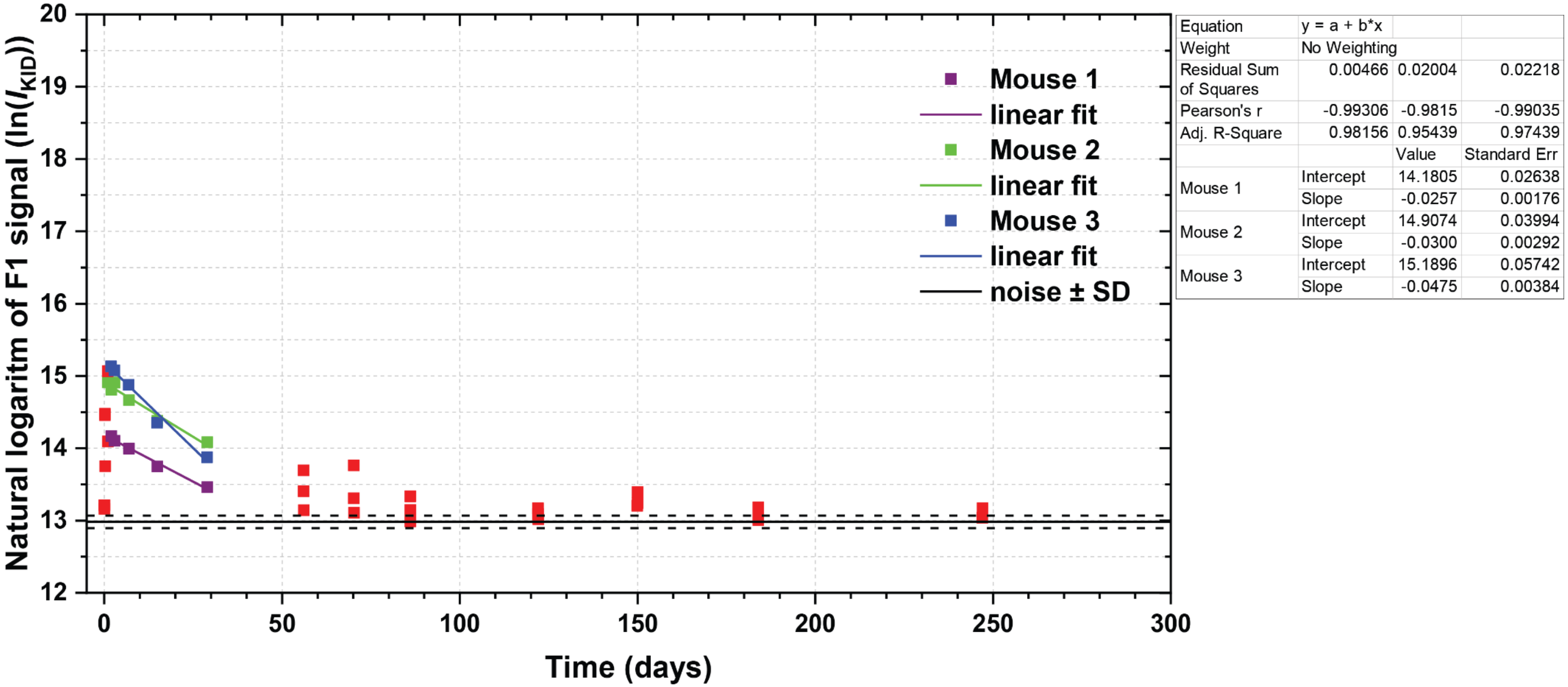

Equation	y = a + b*x		
Weight	No Weighting		
Residual Sum of Squares	0.00466	0.02004	0.02218
Pearson's r	-0.99306	-0.9815	-0.99035
Adj. R-Square	0.98156	0.95439	0.97439
		Value	Standard Err
Mouse 1	Intercept	14.1805	0.02638
	Slope	-0.0257	0.00176
Mouse 2	Intercept	14.9074	0.03994
	Slope	-0.0300	0.00292
Mouse 3	Intercept	15.1896	0.05742
	Slope	-0.0475	0.00384

Figure S100. Signal of **F1** (arbitrary units) in **liver** depot in mice M1, M2, and M3 as a function of time. **Phase 1b**, **3b** and **4b** are marked in red.

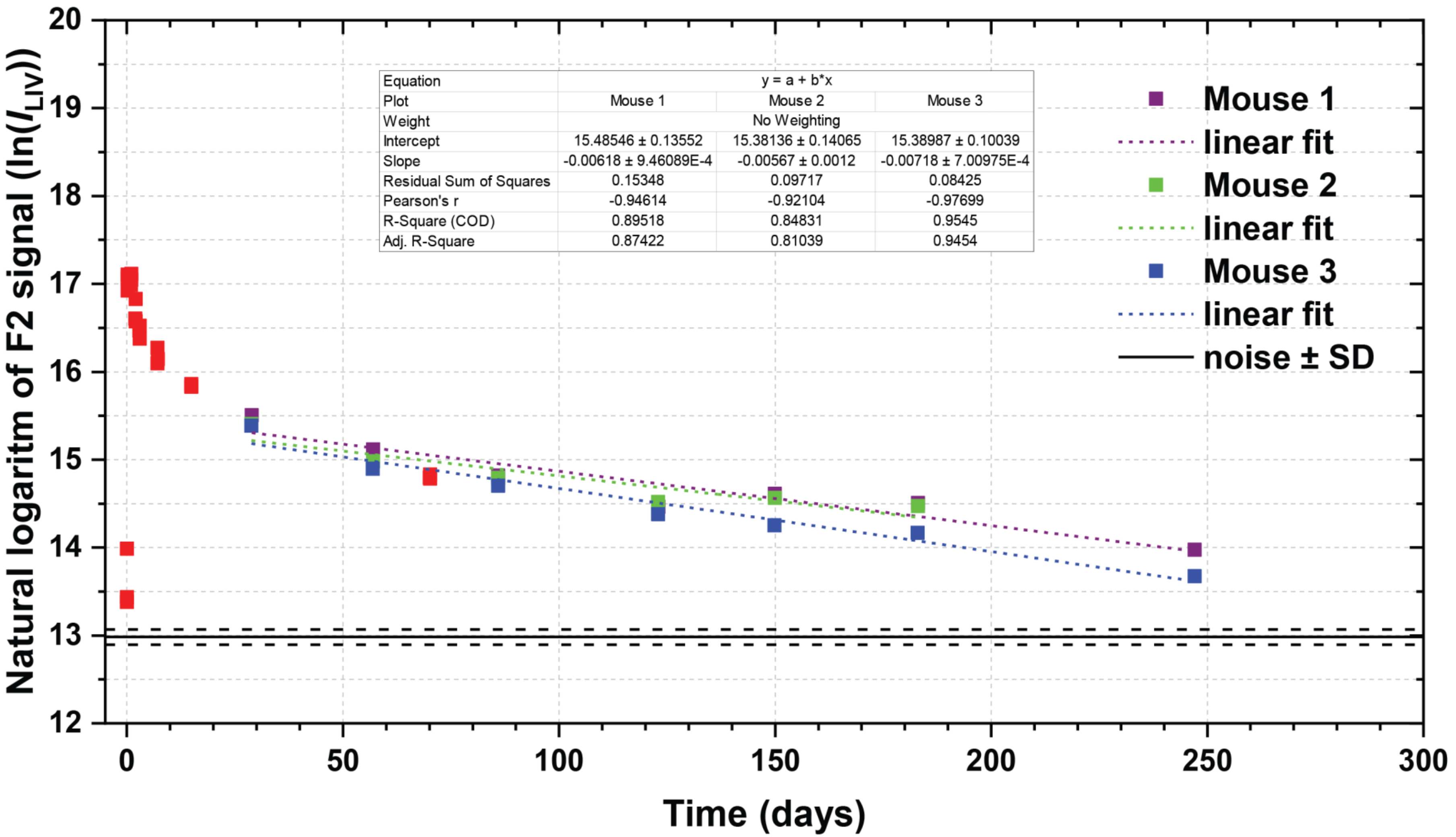

Figure S101. Signal of **F2** (arbitrary units) in **liver** depot in mice M1, M2, and M3 as a function of time. **Phase 1b** are marked in red. In this case, we determined both the kinetics of both **Phase 2b** and **3b**.

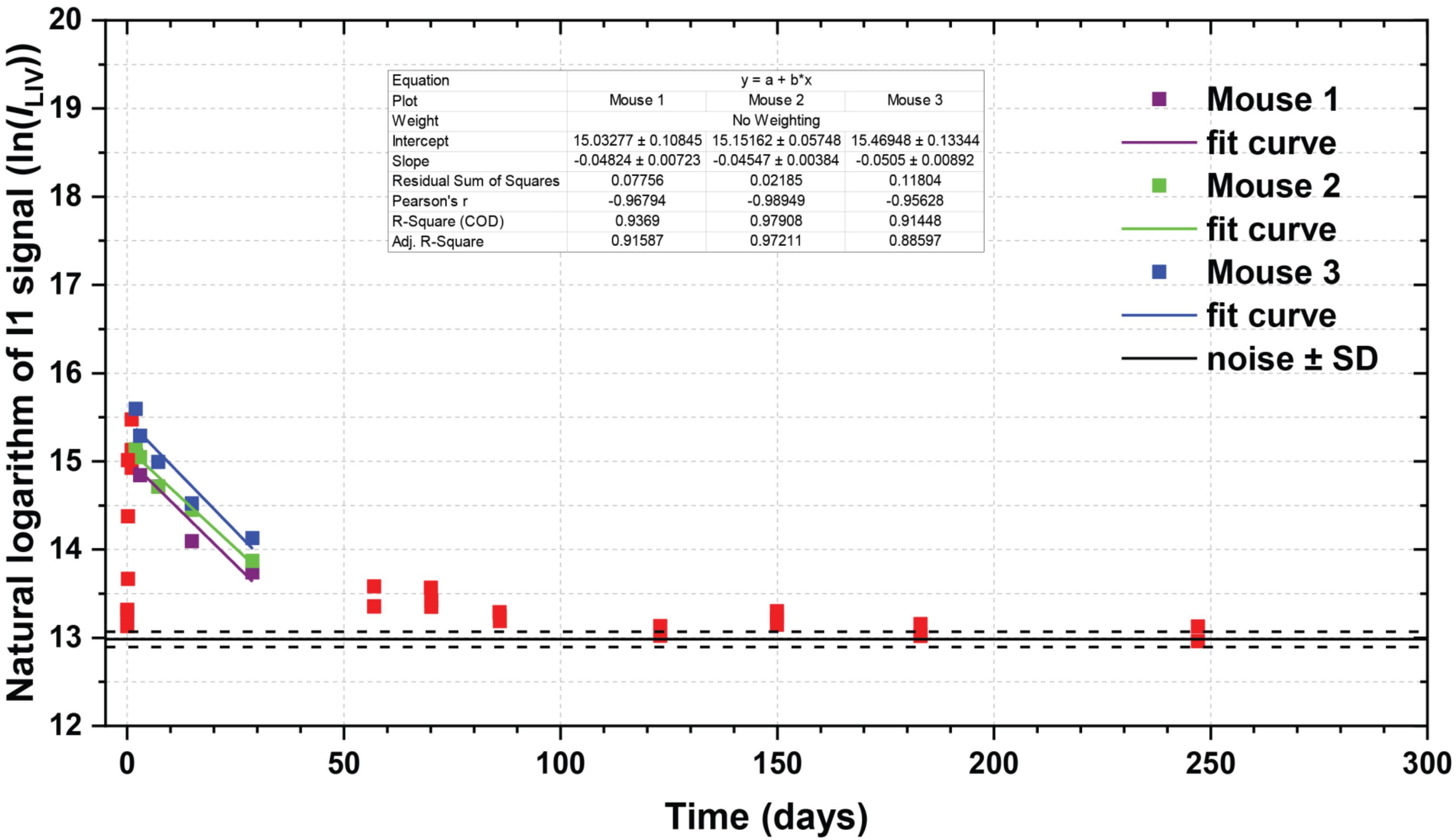

Figure S102. Signal of **I1** (arbitrary units) in **liver** depot in mice M1, M2, and M3 as a function of time. **Phase 1b**, **3b** and **4b** are marked in red.

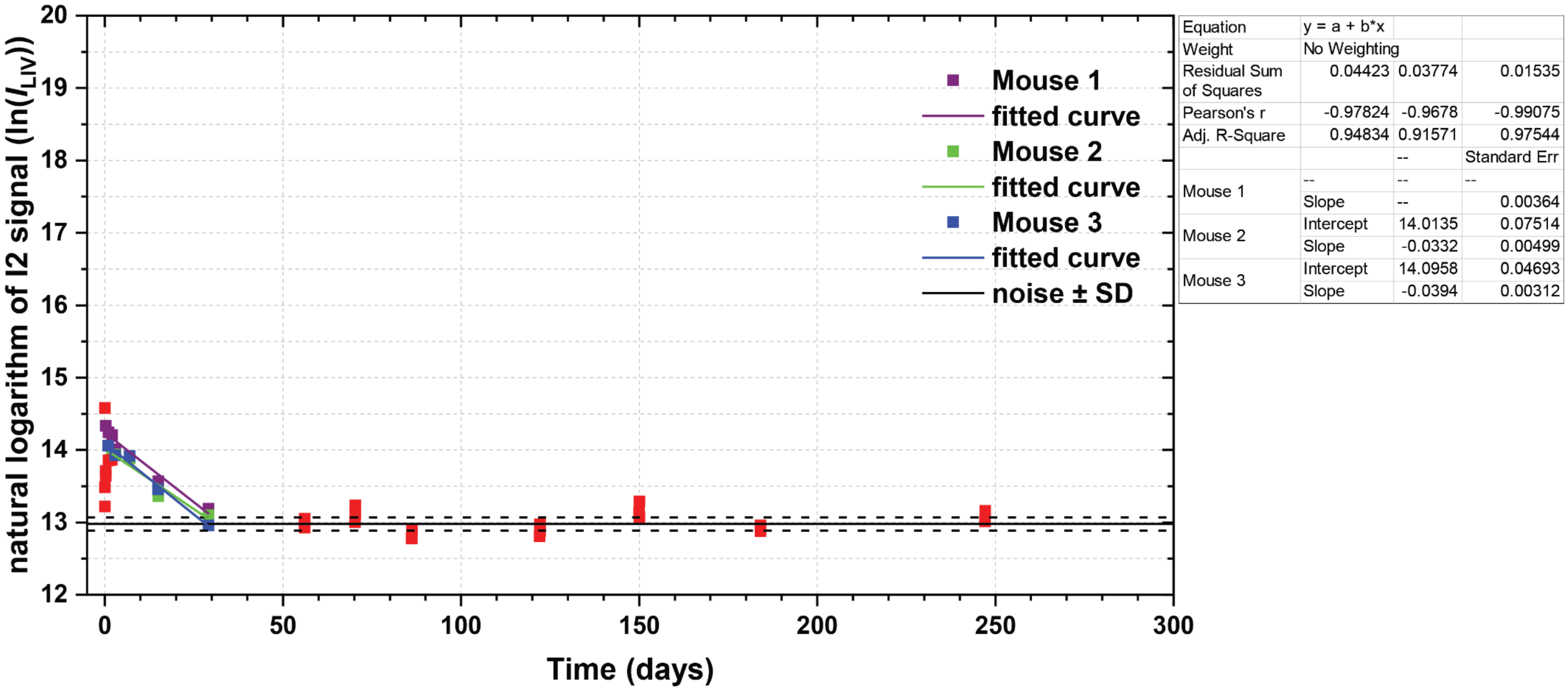

Equation	y = a + b*x		
Weight	No Weighting		
Residual Sum of Squares	0.04423	0.03774	0.01535
Pearson's r	-0.97824	-0.9678	-0.99075
Adj. R-Square	0.94834	0.91571	0.97544
		--	Standard Err
Mouse 1	--	--	--
	Slope	--	0.00364
Mouse 2	Intercept	14.0135	0.07514
	Slope	-0.0332	0.00499
Mouse 3	Intercept	14.0958	0.04693
	Slope	-0.0394	0.00312

Figure S103. Signal of I2 (arbitrary units) in **liver** depot in mice M1, M2, and M3 as a function of time. **Phase 1b**, **3b** and **4b** are marked in red.

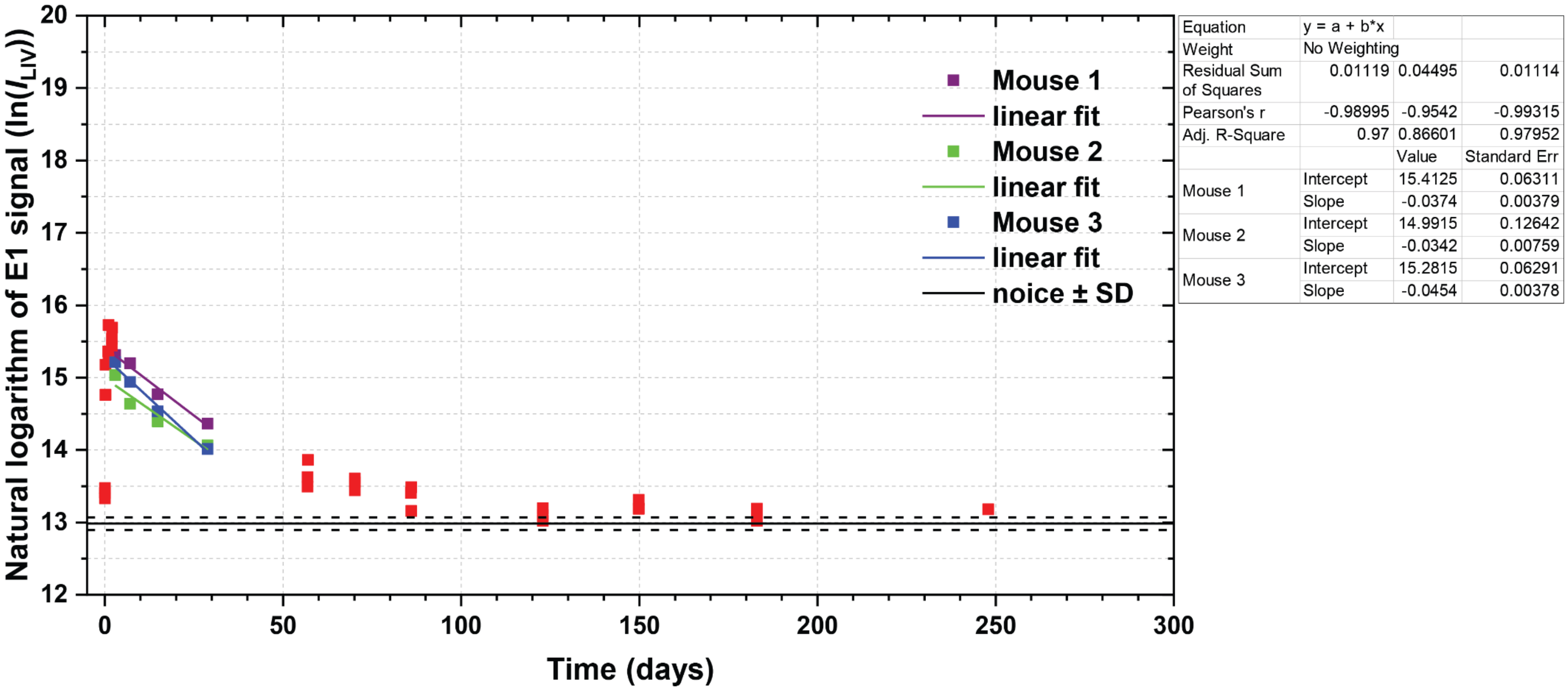

Equation	y = a + b*x		
Weight	No Weighting		
Residual Sum of Squares	0.01119	0.04495	0.01114
Pearson's r	-0.98995	-0.9542	-0.99315
Adj. R-Square	0.97	0.86601	0.97952
		Value	Standard Err
Mouse 1	Intercept	15.4125	0.06311
	Slope	-0.0374	0.00379
Mouse 2	Intercept	14.9915	0.12642
	Slope	-0.0342	0.00759
Mouse 3	Intercept	15.2815	0.06291
	Slope	-0.0454	0.00378

Figure S104. Signal of **E1** (arbitrary units) in **liver** depot in mice M1, M2, and M3 as a function of time. **Phase 1b**, **3b** and **4b** are marked in red.

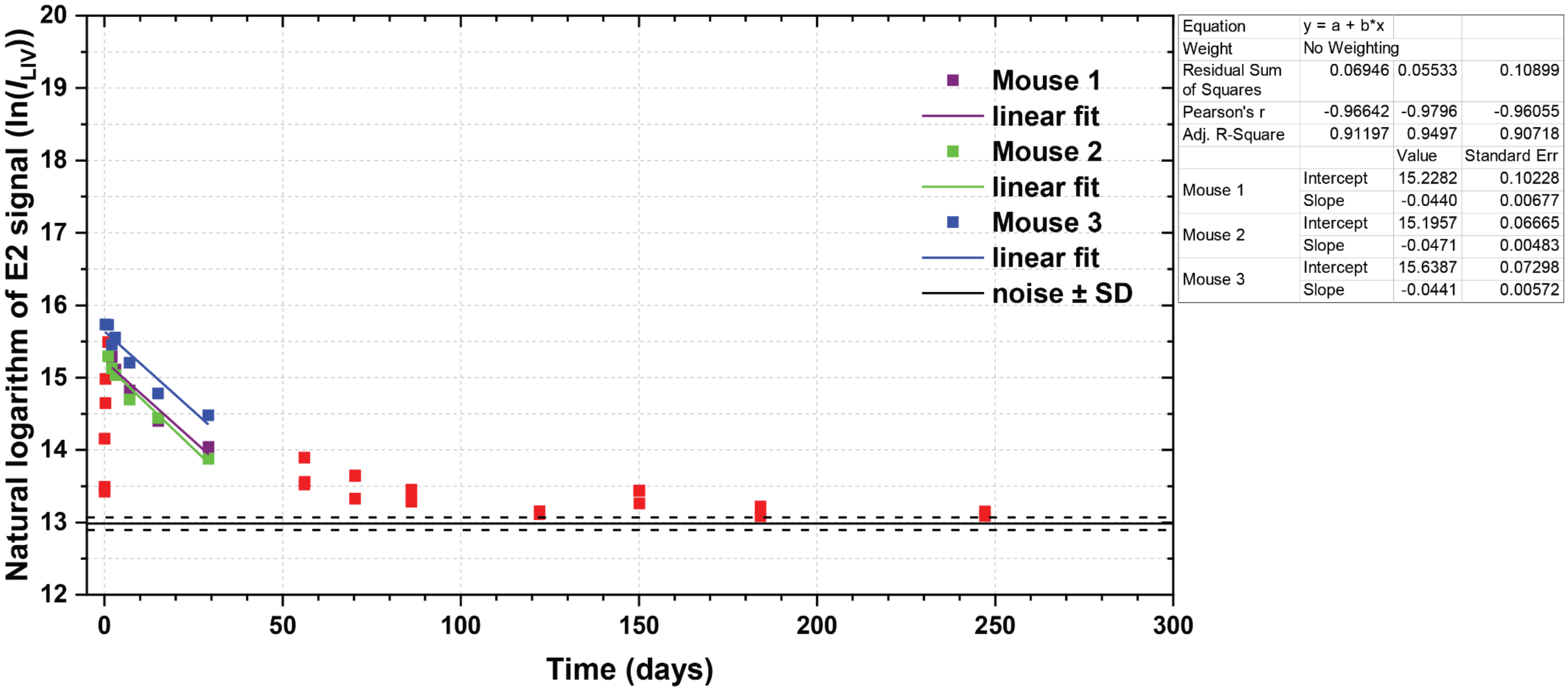

Equation	y = a + b*x		
Weight	No Weighting		
Residual Sum of Squares	0.06946	0.05533	0.10899
Pearson's r	-0.96642	-0.9796	-0.96055
Adj. R-Square	0.91197	0.9497	0.90718
		Value	Standard Err
Mouse 1	Intercept	15.2282	0.10228
	Slope	-0.0440	0.00677
Mouse 2	Intercept	15.1957	0.06665
	Slope	-0.0471	0.00483
Mouse 3	Intercept	15.6387	0.07298
	Slope	-0.0441	0.00572

Figure S105. Signal of E2 (arbitrary units) in **liver** depot in mice M1, M2, and M3 as a function of time. **Phase 1b**, **3b** and **4b** are marked in red.

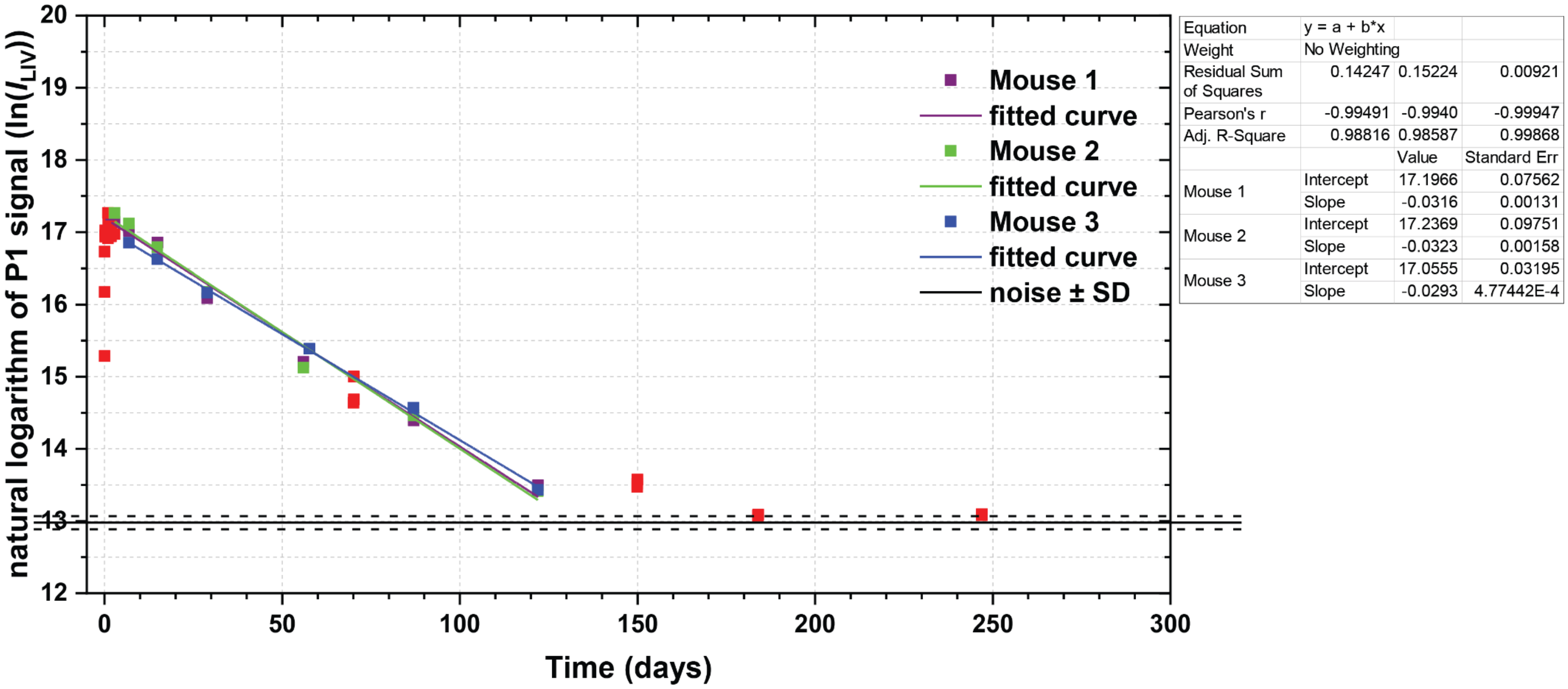

Equation	y = a + b*x		
Weight	No Weighting		
Residual Sum of Squares		0.14247 0.15224	0.00921
Pearson's r		-0.99491 -0.9940	-0.99947
Adj. R-Square		0.98816 0.98587	0.99868
		Value	Standard Err
Mouse 1	Intercept	17.1966	0.07562
	Slope	-0.0316	0.00131
Mouse 2	Intercept	17.2369	0.09751
	Slope	-0.0323	0.00158
Mouse 3	Intercept	17.0555	0.03195
	Slope	-0.0293	4.77442E-4

Figure S106. Signal of **P1** (arbitrary units) in **liver** depot in mice M1, M2, and M3 as a function of time. **Phase 1b**, **3b** and **4b** are marked in red.

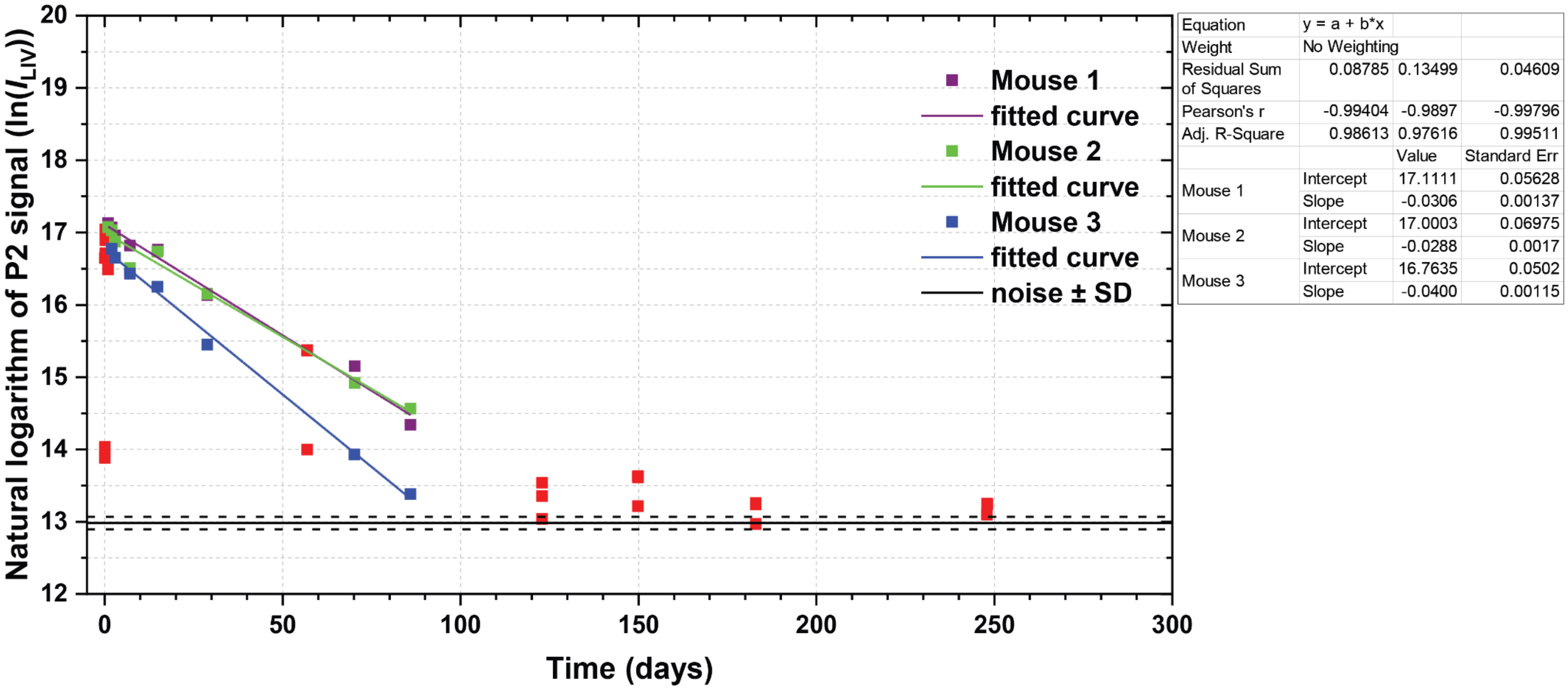

Equation	y = a + b*x		
Weight	No Weighting		
Residual Sum of Squares	0.08785	0.13499	0.04609
Pearson's r	-0.99404	-0.9897	-0.99796
Adj. R-Square	0.98613	0.97616	0.99511
		Value	Standard Err
Mouse 1	Intercept	17.1111	0.05628
	Slope	-0.0306	0.00137
Mouse 2	Intercept	17.0003	0.06975
	Slope	-0.0288	0.0017
Mouse 3	Intercept	16.7635	0.0502
	Slope	-0.0400	0.00115

Figure S107. Signal of **P2** (arbitrary units) in **liver** depot in mice M1, M2, and M3 as a function of time. **Phase 1b**, **3b** and **4b** are marked in red.

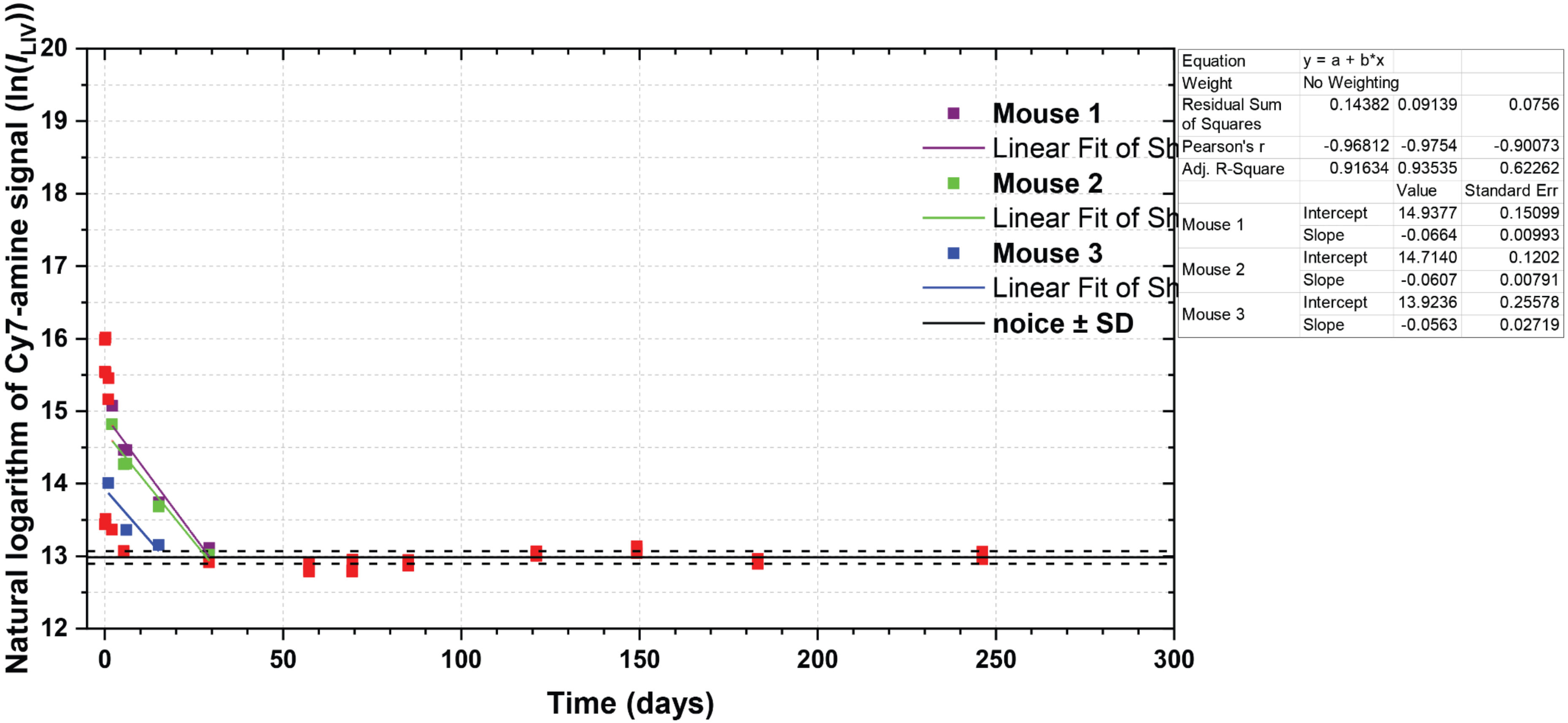

Equation	y = a + b*x		
Weight	No Weighting		
Residual Sum of Squares	0.14382	0.09139	0.0756
Pearson's r	-0.96812	-0.9754	-0.90073
Adj. R-Square	0.91634	0.93535	0.62262
		Value	Standard Err
Mouse 1	Intercept	14.9377	0.15099
	Slope	-0.0664	0.00993
Mouse 2	Intercept	14.7140	0.1202
	Slope	-0.0607	0.00791
Mouse 3	Intercept	13.9236	0.25578
	Slope	-0.0563	0.02719

Figure S108. Signal of **Cy7-amine** (arbitrary units) in **liver** depot in mice M1, M2, and M3 as a function of time. **Phase 1b**, **3b** and **4b** are marked in red.

S13. Polymer pharmacokinetics – results

Table S80. Intercepts of linear fit of intramuscular depots for Mouse 1, 2 and 3. Pink cells indicate outlier data (these data were not used in final calculations)

	Mouse 1		Mouse 2		Mouse 3	
	Mean	SD	Mean	SD	Mean	SD
F1	**17.14555**	0.05582	**18.01932**	0.05302	**18.16249**	0.06389
F2	**18.68192**	0.05357	**18.66596**	0.08778	**18.55071**	0.05208
I1	**19.62194**	0.0708	**19.35725**	0.09089	**19.94452**	0.05867
I2	**18.62149**	0.08597	**18.95959**	0.05375	**19.34599**	0.07024
E1			**20.37736**	0.11825	**20.37574**	0.14177
E2	**20.65803**	0.07725	**20.44365**	0.17365	**20.79868**	0.09785
P1	**20.49036**	0.10125	**20.56353**	0.10967	**20.66441**	0.09077
P2	**20.34815**	0.14559			**20.34805**	0.17013
Cy7	**20.77434**	0.16268	**21.07118**	0.16029	**18.694**	0.02813
Noise	**11.80571**	0.121409				

Table S81. Slopes of linear fit of intramuscular depots for Mouse 1, 2 and 3. Pink cells indicate outlier data (these data were not used in final calculations)

	Mouse 1		Mouse 2		Mouse 3	
	Mean	SD	Mean	SD	Mean	SD
F1	**-0.00322**	4.16E-04	**-0.0042**	4.87E-04	**-0.00693**	4.76E-04
F2	**-0.00456**	3.74E-04	**-0.00497**	7.48E-04	**-0.0048**	3.64E-04
I1	**0.01566**	7.38E-04	**0.01297**	9.47E-04	**0.01432**	6.11E-04
I2	**-0.00951**	7.16E-04	**-0.01064**	4.91E-04	**-0.01144**	5.85E-04
E1	**0.04457**	0.0031	**0.04199**	0.00267	**0.04443**	0.0032
E2	**-0.04234**	0.00175	**-0.04283**	0.00393		
P1	**-0.04034**	0.00208	**-0.04104**	0.00226	**-0.03867**	0.00187
P2	**-0.0422**	0.003	**-0.04072**	0.00244	**-0.04469**	0.00351
Cy7	**-0.1357**	0.00958	**0.13452**	0.00945	**0.15126**	0.00429

Table S82. Number of fitted points in linear fit of intramuscular depots for Mouse 1, 2 and 3

	Mouse 1	Mouse 2	Mouse 3
	Mean	Mean	Mean
F1	16	14	16
F2	14	12	14
I1	18	18	18
I2	20	20	20
E1	12	12	12
E2	12	12	12
P1	10	10	10
P2	10	10	10
Cy7	8	8	14

Table S83. Intercepts of linear fit of liver depots for Mouse 1, 2 and 3. Pink cells indicate outlier data (these data were not used in final calculations)

	Mouse 1		Mouse 2		Mouse 3	
	Mean	SD	Mean	SD	Mean	SD
**F1**	14.18052	0.02638	14.90748	0.03994	15.18969	0.05742
**I1**	15.03277	0.10845	15.15162	0.05748	15.46948	0.13344
**I2**	14.23788	0.0465	14.01358	0.07514	14.09581	0.04693
**E1**	15.41254	0.06311	14.99159	0.12642	15.28155	0.06291
**E2**	15.22827	0.10228	15.19578	0.06665	15.63879	0.07298
**P1**	17.19661	0.07562	17.2369	0.09751	17.05551	0.03195
**P2**	17.11112	0.05628	17.00039	0.06975	16.76352	0.0502
**Cy7**	14.93779	0.15099	14.71405	0.1202	13.92362	0.25578
**Noise**	**11.80571**	**0.121409**				

Table S84. Slopes of linear fit of liver depots for Mouse 1, 2 and 3. Pink cells indicate outlier data (these data were not used in final calculations)

	Mouse 1		Mouse 2		Mouse 3	
	Mean	SD	Mean	SD	Mean	SD
**F1**	-0.02572	0.00176	-0.03002	0.00292	-0.04751	0.00384
**I1**	-0.04824	0.00723	-0.04547	0.00384	-0.0505	0.00892
**I2**	-0.03839	0.00364	-0.03324	0.00499	-0.03948	0.00312
**E1**	-0.03748	0.00379	-0.03427	0.00759	-0.04542	0.00378
**E2**	-0.04408	0.00677	-0.04719	0.00483	-0.04415	0.00572
**P1**	-0.03165	0.00131	-0.03235	0.00158	-0.02935	4.77E-04
**P2**	-0.03066	0.00137	-0.02886	0.0017	-0.04007	0.00115
**Cy7**	-0.06647	0.00993	-0.06073	0.00791	-0.05638	0.02719

Table S85. Number of fitted points in linear fit of liver depots for Mouse 1, 2 and 3

	Mouse 1 Mean	Mouse 2 Mean	Mouse 3 Mean
**F1**	**5**	**6**	**5**
**I1**	**5**	**5**	**5**
**I2**	**7**	**5**	**5**
**E1**	**4**	**4**	**4**
**E2**	**5**	**5**	**5**
**P1**	**8**	**7**	**6**
**P2**	**8**	**8**	**7**
**Cy7**	**4**	**4**	**3**

Table S86. Intercepts of linear fit of kidney depots for Mouse 1, 2 and 3. Pink cells indicate outlier data (these data were not used in final calculations)

	**Mouse 1**		**Mouse 2**		**Mouse 3**	
	**Mean**	**SD**	**Mean**	**SD**	**Mean**	**SD**
**F1**	13.28044	0.06634	14.15442	0.04334	14.153	0.08747
**I1**	13.75748	0.03395	13.87998	0.0376	14.29449	0.00877
**I2**	12.70285	0.08595	12.79299	0.02959	13.11302	0.06062
**E1**	13.79741	0.03666	13.44307	0.04407	13.81879	0.05746
**E2**	13.43319	0.04619	13.73106	0.01346	14.38711	0.04212
**P1**	16.37078	0.05116	16.53882	0.0649	16.71568	0.07435
**P2**	16.01147	0.06122	16.24837	0.08235	15.71587	0.06545
**Cy7**	14.88209	0.05499	14.88823	0.05699		
**Noise**	**11.80571**	**0.121409**				

Table S87. Slopes of linear fit of kidney depots for Mouse 1, 2 and 3. Pink cells indicate outlier data (these data were not used in final calculations)

	**Mouse 1**		**Mouse 2**		**Mouse 3**	
	**Mean**	**SD**	**Mean**	**SD**	**Mean**	**SD**
**F1**	-0.01228	0.00126	-1.28E-02	9.51E-04	-0.02079	0.0018
**I1**	-0.0523	0.00348	-0.04466	0.00387	-0.04583	9.05E-04
**I2**	-0.02355	0.00443	-0.02839	0.00153	-0.03325	0.00314
**E1**	-0.04539	0.00245	-0.03452	0.00295	-0.04371	0.00385
**E2**	-0.03227	0.00306	-0.04198	9.76E-04	-0.0404	0.0033
**P1**	-0.08537	0.00374	-0.08852	0.00476	-0.08628	0.00546
**P2**	-0.07865	0.00448	-0.0822	0.00603	-0.07526	0.0048
**Cy7**	-0.07927	0.00396	-0.0829	0.00411		

Table S88. Number of fitted points in kidney fit of liver depots for Mouse 1, 2 and 3. Pink cells indicate outlier data (these data were not used in final calculations)

	**Mouse 1 Mean**	**Mouse 2 Mean**	**Mouse 3 Mean**
**F1**	**7**	**7**	**7**
**I1**	**3**	**3**	**3**
**I2**	**3**	**3**	**3**
**E1**	**5**	**5**	**5**
**E2**	**5**	**6**	**7**
**P1**	**6**	**6**	**6**
**P2**	**6**	**6**	**6**
**Cy7**	**6**	**6**	

Table S89. Parameters of dissolution of secondary depots in kidney and liver: kidney/ liver polymer accumulation factor (f_{KID} and f_{LIV}); range of maximum signal ($t_{I,\,\mathrm{max}}$); and biological half-lives ($t_{½}$). The results are expressed as mean ± SD (based on pooled variances of fits). Asterisk (*) indicates skewed data caused by too intense delocalized signal.

**Polymer**		**Kidney signal**		**Liver signal**		**Amplitude to noise**		**Accumulation factor**		**Selectivity**
		$t_{I,\,\mathrm{max}}$ (d)	$t_{½,I,\mathrm{KID}}$ (d)	$t_{I,\,\mathrm{max}}$ (d)	$t_{½,I,\mathrm{LIV}}$ (d)	$I_{\mathrm{KID}}/I_{\mathrm{noise}}$	$I_{\mathrm{LIV}}/I_{\mathrm{noise}}$	f_{KID} (%)	f_{LIV} (%)	$f_{\mathrm{LIV}}/f_{\mathrm{KID}}$
**pDFEA**	**F1**	1 to 7	**45.3 ± 4.1**	1 to 2	**20.3 ± 1.8**	10.5 ± 1.5	19.4 ± 2.5	2.45 ± 0.21	4.75 ± 0.33	1.94 ± 0.21
	**F2**	0.2 to 1	*	0.2 to 1	*	*	*	*	*	*
**pNIPAM**	**I1**	1 to 3	**14.6 ± 0.9**	1 to 2	**14.4 ± 2.1**	8.8 ± 1.1	30.3 ± 4.8	0.31 ± 0.02	1.16 ± 0.15	3.77 ± 0.56
	**I2**	0.2 to 7	**24.4 ± 2.8**	1 to 2	**18.6 ± 2.0**	2.9 ± 0.4	10.2 ± 1.4	0.15 ± 0.01	0.71 ± 0.06	4.86 ± 0.56
**pDEA**	**E1**	1 to 3	**16.8 ± 1.3**	1 to 2	**17.7 ± 2.4**	6.6 ± 0.9	30.7 ± 4.6	0.11 ± 0.01	0.56 ± 0.09	5.34 ± 1.08
	**E2**	0.2 to 3	**17.9 ± 1.2**	0.2 to 1	**15.4 ± 2.0**	8.1 ± 1.0	34.8 ± 5.1	0.10 ± 0.01	0.50 ± 0.07	4.72 ± 0.89
**pAP**	**P1**	0.2 to 1	**8.0 ± 0.4**	0.2 to 3	**22.2 ± 0.9**	114 ± 16	213.6 ± 30.3	1.76 ± 0.21	3.31 ± 0.41	1.88 ± 0.32
	**P2**	0.2 to 2	**8.8 ± 0.6**	0.2 to 3	**21.1 ± 0.9**	66 ± 9.2	174.4 ± 23.5	1.26 ± 0.22	3.38 ± 0.57	2.68 ± 0.62
**Cy7**		0.2 to 2	**8.5 ± 0.4**	< 1	**10.9 ± 1.5**	21.7 ± 2.9	16.0 ± 3.3	0.64 ± 0.08	0.47 ± 0.10	0.72 ± 0.17

The peak signal found in kidney and liver depots varied in different polymers. These data show polymers showed variable levels of affinities to liver and kidneys, which can be attributed to various mechanisms of uptake in kidney and liver cells. Note that liver, kidney, and muscle depots are situated in various depths below the body-surface of the mice, therefore the fluorescence signal in these organs experiences various levels of shielding. As a result, the absolute values of f correlate with the polymer uptake into liver or kidney but are not equal to it. Nonetheless, f values are still useful for estimating (i) which polymers were the most likely to end up in kidneys or liver and (ii) which polymers showed higher relative affinity towards a particular organ.

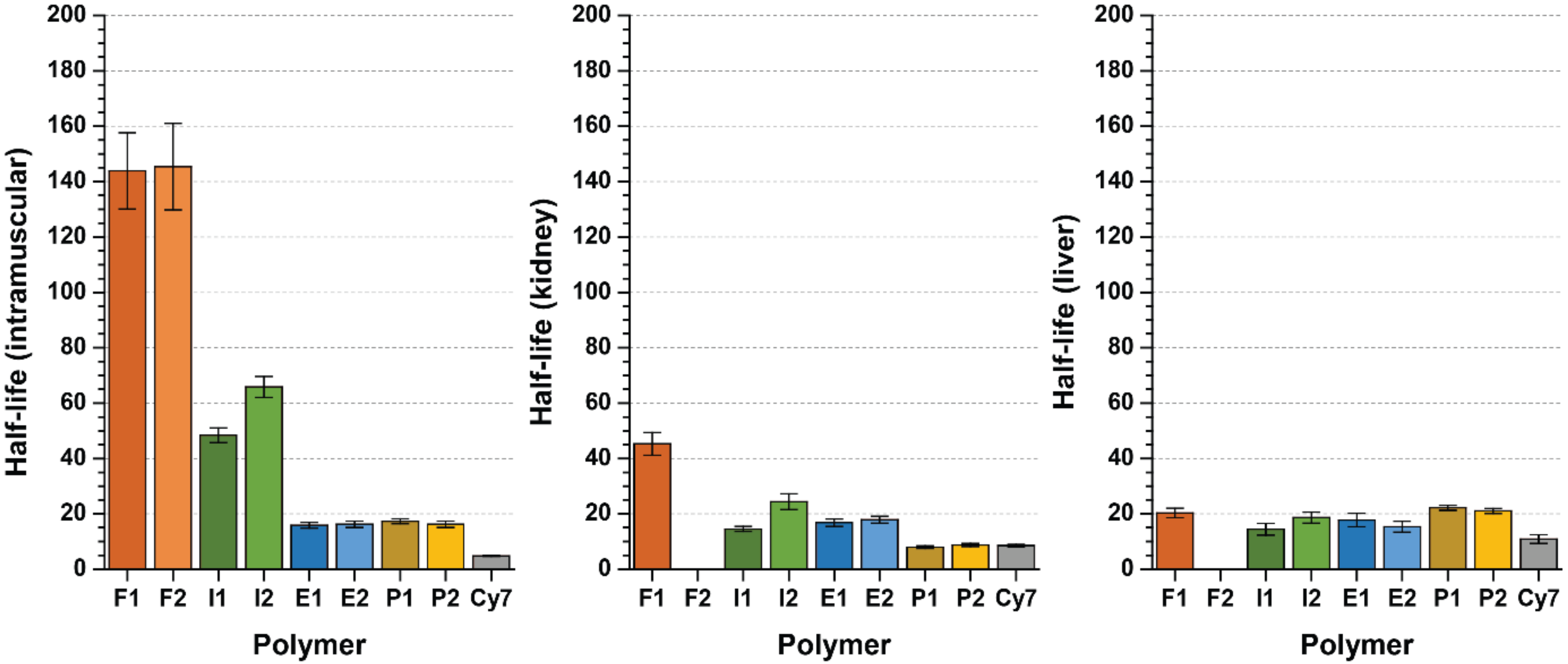

Figure S109. Biological half-lives of all polymers in various tissues; half-lives of **F2** in kidney and liver were not determined

S14. Statistical analysis

We constructed a correlation matrix of $\bar{k}_T$ and $f_{\mathrm{LIV/KID}}$ with known variables of the studied polymers (their heat properties, *section S8.3*; and their enthalpies of interactions with serum proteins temperatures below ($H_{\mathrm{prot,\ low\text{-}temp,\ sol}}$) and above ($H_{\mathrm{prot,\ high\text{-}temp,\ sol}}$) their T_{CP},[1] **Table S90**). Given the low sample size (number of tested polymers), we consider correlation coefficient in range of 0.7 to 1.0 as an indicator of strong correlation and 0.7 to 0.5 as an indicator of moderate correlation. We also consider strongly non-correlating variables in range of correlation coefficients 0.2 to –0.2 as statistically significant.

Under these constraints, we conclude that:

For $\bar{k}_{\mathrm{LIV}}$:

I. Strong positive correlation with $f_{\mathrm{LIV/KID}}$ and strong negative correlation with $\Delta H_{\mathrm{aag,\ neat}}$.
II. Moderate positive correlation with $Đ_{\mathrm{M}}$, $T_{\mathrm{CP,\ 10.0\ mg/mL}}$ and $T_{\mathrm{CP,\ 1.25\ mg/mL}}$ and moderate negative correlation with $H_{\mathrm{prot,\ low\text{-}temp,\ sol}}$.
III. No correlation with M_{w}, $H_{\mathrm{prot,\ high\text{-}temp,\ sol}}$ and $\bar{k}_{\mathrm{IM}}$.

For $\bar{k}_{\mathrm{KID}}$:

I. Strong negative correlation with $T_{\mathrm{CP,\ 10.0\ mg/mL}}$ and $T_{\mathrm{CP,\ 1.25\ mg/mL}}$.
II. Moderate positive correlation with $\Delta H_{\mathrm{aag,\ neat}}$, and moderate negative correlation with $Đ_{\mathrm{M}}$, $T_{\mathrm{g,\ neat}}$, $H_{\mathrm{prot,\ low\text{-}temp,\ sol}}$ and $\bar{k}_{\mathrm{IM}}$.
III. No correlation with M_{w} and $H_{\mathrm{prot,\ high\text{-}temp,\ sol}}$.

For $\bar{k}_{\mathrm{IM}}$:

I. No strongly correlated variables.
II. Moderate positive correlation with $T_{\mathrm{CP,\ 10.0\ mg/mL}}$ and $T_{\mathrm{CP,\ 1.25\ mg/mL}}$, $Đ_{\mathrm{M}}$, and $H_{\mathrm{prot,\ high\text{-}temp,\ sol}}$ and moderate negative correlation with $\bar{k}_{\mathrm{KID}}$
III. No correlation with M_{w}, $T_{\mathrm{g,\ neat}}$, k_{LIV}, $H_{\mathrm{prot,\ low\text{-}temp,\ sol}}$ and $\Delta H_{\mathrm{aag,\ neat}}$

For $f_{\mathrm{LIV/KID}}$

I. Strong positive correlation with $\Delta H_{\mathrm{aag,\ neat}}$ and $H_{\mathrm{prot,\ high\text{-}temp,\ sol}}$
II. Moderate negative correlation with $Đ_{\mathrm{M}}$, $T_{\mathrm{CP,\ 10.0\ mg/mL}}$ and $T_{\mathrm{CP,\ 1.25\ mg/mL}}$, $T_{\mathrm{g,\ neat}}$ and $\bar{k}_{\mathrm{LIV}}$.
III. No correlation with M_{w}, H_{Tg}, $H_{\mathrm{prot,\ low\text{-}temp,\ sol}}$ and $\bar{k}_{\mathrm{IM}}$

Table S90. Correlation matrix of of $\bar{k}_T$ and $f_{LIV/KID}$ with known polymer variables, color-coded by correlation coefficient (– 1.0 to – 0.7 (orange), – 0.7 to – 0.5 (light-orange), – 0.5 to 0.5 (white), 0.5 to 0.7 (light-green), and 0.7 to 1.0 (green).

	$\bar{k}_{LIV}$	$\bar{k}_{KID}$	$\bar{k}_{IM}$	$f_{LIV/KID}$
M_w	0.06	0.10	0.15	0.18
$Đ_M$	0.55	– 0.52	0.59	– 0.58
$T_{CP, 10.0 mg/mL}$	0.62	– 0.94	0.52	– 0.61
$T_{CP, 1.25 mg/mL}$	0.63	– 0.87	0.47	– 0.64
$T_{g, neat}$	0.38	– 0.58	– 0.20	– 0.53
H_{tg}	– 0.28	0.24	– 0.76	0.07
$\Delta H_{aag, neat}$	– 0.82	0.54	– 0.14	0.91
$H_{prot, low-temp, sol}$	– 0.57	0.03	0.59	0.85
$H_{prot, high-temp, sol}$	– 0.06	– 0.62	0.15	0.10
$\bar{k}_{LIV}$	1.00	– 0.36	0.10	– 0.61
$\bar{k}_{KID}$	– 0.36	1.00	– 0.65	0.39
$\bar{k}_{IM}$	0.10	– 0.65	1.00	0.16
$f_{LIV/KID}$	0.71	– 0.24	– 0.26	1.00

Table S91. Polymer parameters used in eigenvalues of correlation analysis

	M_w (kg/mol)	$Đ_M$ (Mw/Mn)	$T_{CP, 10.0}$ (°C)	$T_{CP, 1.25}$ (°C)	$T_{g, neat}$ (°C)	H_{tg} (J/g)	$\Delta H_{aag, neat}$ (J/g)	$H_{prot, low-temp, sol}$ (J/g)	$H_{prot, high-temp, sol}$ (J/g)
**F1**	26.2	1.08	22.6	33.9	113	0.336	8	-86.7	-559.6
**F2**	36.2	1.03	30.1	48	113	0.336	8	-86.7	-559.6
**I1**	20.2	1.03	25.7	33.6	136	0.506	36	-14.2	19.5
**I2**	31.6	1.03	24.4	30.9	136	0.506	36	-14.2	19.5
**E1**	22.3	1.06	24.4	35.7	95	0.245	35	52.5	-287.2
**E2**	34.7	1.09	25.8	32.6	95	0.245	35	52.5	-287.2
**P1**	19.6	1.11	56.7	75	144	0.303	2	-36.7	-19.6
**P2**	36	1.09	48.6	51.2	144	0.303	2	-36.7	-19.6

Table S92. Principal component analysis vectors

	Coefficients of PC1	Coefficients of PC2	Coefficients of PC3	Coefficients of PC4
M_w	-0.0325	0.13438	-0.04091	0.92623
$Đ_M$	0.307	0.31082	-0.18552	0.01775
$T_{CP, 10.0 mg/mL}$	0.39078	0.03268	0.1917	0.02483
$T_{CP, 1.25 mg/mL}$	0.38452	0.03732	0.13167	-0.21496
$T_{g, neat}$	0.24386	-0.39906	0.23195	0.15612
H_{tg}	-0.12927	-0.51611	0.17185	0.0457
$\Delta H_{aag, neat}$	-0.3745	0.04095	0.2416	-0.08502
$H_{prot, low-temp, sol}$	-0.20455	0.38864	0.33929	-0.05885
$H_{prot, high-temp, sol}$	0.09905	-0.21063	0.5724	0.13933
$\bar{k}_{LIV}$	0.31044	-0.03049	-0.21919	0.16343
$\bar{k}_{KID}$	-0.33292	-0.07773	-0.35383	0.04382
$\bar{k}_{IM}$	0.15266	0.46832	0.26868	0.02004
$f_{LIV/KID}$	-0.32487	0.18405	0.28272	0.09678

S15. Histopathological examination

Lastly, we performed a thorough histopathological examination of all groups (*section S15.1*) 202 days after the administration, because such time was sufficient for some pathologies to manifest.[48] We examined the site of administration (and compared it to contralateral muscles) and organs in which polymers accumulated in our or in previous studies (liver, kidneys, hearts).[21,39,41] In these organs, we observed no pathology, nor any major difference between test and control groups (*section S15.1*).[48,49] Our results show that the polymers did not cause any harm to the animals upon chronic use, therefore, such materials might be considered for human medicinal applications.

 DOI: XXXX.YYYY/ZZZZ

S15.1. Histopathological findings (overview)

We observed no pathology in any group.[48,49] Furthermore, we saw no major difference between control groups and study groups. For details, see **Table S93**.

Table S93. List of all findings from autopsy and subsequent histopathological examination.

Polymer	Autopsy findings (macroscopic description)	Spleen	Liver	Kidney	Injected muscle	Control muscle
**F1**	**Liver**: size normal; darker than in DMSO<br>**Kidney**: Larger than in F2 or DMSO, colour normal<br>**Spleen**: normal size and colour<br>**Lungs**: normal size and colour<br>**Heart**: normal size and colour<br>**Pancreas**: minor haemorrhage, normal size and colour<br>**Muscles**: normal size and colour	No sign of chronic congestion, fibrosis, or hemosiderin accumulation; well developed white and red pulp, no sign of presence of foreign material	No sign of steatosis, fibrosis, or ischemia; no sign of pathologic Kupffer cell proliferation	No sign of fibrosis or any other pathologic change	No sign of fibrosis or chronic inflammation. Cells are basophilic, cytoplasm is slightly granular; presence of mast cells.	No sign of fibrosis or chronic inflammation. Cells are basophilic, cytoplasm is slightly granular; presence of mast cells.
**F2**	**Liver**: Smaller than F1 or DMSO, colour normal<br>**Kidney**: Larger than in DMSO, colour normal<br>**Spleen**: normal size and colour<br>**Lungs**: normal size and colour<br>**Heart**: normal size and colour<br>**Pancreas**: darker than in F1 or DMSO.<br>**Muscle**: normal colour	No sign of chronic congestion, fibrosis, or hemosiderin accumulation; well developed white and red pulp, no sign of presence of foreign material	No sign of steatosis, fibrosis, or ischemia; no sign of pathologic Kupffer cell proliferation	No sign of fibrosis or any other pathologic change	No sign of fibrosis or chronic inflammation. Cells are basophilic, cytoplasm is slightly granular; presence of mast cells.	No sign of fibrosis or chronic inflammation. Cells are basophilic, cytoplasm is slightly granular; presence of mast cells.
**P1**	**Liver**: Larger than DMSO, colour normal. Small haemorrhage in the capsule<br>**Kidney**: larger and lighter than P2 and DMSO, one haemorrhage.<br>**Spleen**: normal size and colour<br>**Lungs**: small fibrotic fusions with pleura<br>**Heart**: Larger than in P2 and DMSO, one haemorrhage<br>**Pancreas**: smaller than in DMSO.<br>**Muscle**: normal colour	No sign of chronic congestion, fibrosis, or hemosiderin accumulation; well developed white and red pulp, no sign of presence of foreign material	No sign of steatosis, fibrosis, or ischemia; no sign of pathologic Kupffer cell proliferation	No sign of fibrosis or any other pathologic change	No sign of fibrosis or chronic inflammation. Cells are basophilic, cytoplasm is slightly granular; presence of mast cells.	No sign of fibrosis or chronic inflammation. Cells are basophilic, cytoplasm is slightly granular; presence of mast cells.
**P2**	**Liver**: smaller than in P1 and DMSO, lighter than in DMSO; capsule enlargement in one lobe.<br>**Kidneys**: Normal size and colour; visible vein<br>**Spleen**: normal size and colour	No sign of chronic congestion, fibrosis, or hemosiderin	No sign of steatosis, fibrosis, or ischemia;	No sign of fibrosis or any other	No sign of fibrosis or chronic inflammation. Cells are basophilic, cytoplasm is slightly	No sign of fibrosis or chronic inflammation. Cells are basophilic,

	**Lungs**: normal size and colour<br>**Heart**: normal size and colour<br>**Pankreas**: N/A<br>**Muscle**: normal colour	accumulation; well developed white and red pulp, no sign of presence of foreign material	no sign of pathologic Kupffer cell proliferation	pathologic change	granular; presence of mast cells.	cytoplasm is slightly granular; presence of mast cells.
**E1**	**Liver**: Larger than DMSO, colour uneven, local capsule enlargement.<br>**Kidneys**: Normal size, lighter than in E2 and DMSO.<br>**Spleen**: normal size and colour<br>**Lungs**: normal size and colour<br>**Heart**: normal size and colour<br>**Pancreas**: smaller than in DMSO<br>**Muscle**: normal colour	No sign of chronic congestion, fibrosis, or hemosiderin accumulation; well developed white and red pulp, no sign of presence of foreign material	No sign of steatosis, fibrosis, or ischemia; no sign of pathologic Kupffer cell proliferation	No sign of fibrosis or any other pathologic change	No sign of fibrosis or chronic inflammation. Cells are basophilic, cytoplasm is slightly granular; presence of mast cells.	No sign of fibrosis or chronic inflammation. Cells are basophilic, cytoplasm is slightly granular; presence of mast cells.
**E2**	**Liver**: same as in E1, colour normal. Dark stripe on one lobe<br>**Kidneys**: Normal size and colour, accumulation of adipose tissue around the capsule.<br>**Spleen**: Normal size and colour, accumulation of adipose tissue around the capsule.<br>**Lungs**: normal size and colour<br>**Heart**: normal size and colour<br>**Pancreas**: normal colour, smaller than DMSO<br>**Muscle**: normal colour	No sign of chronic congestion, fibrosis, or hemosiderin accumulation; well developed white and red pulp, no sign of presence of foreign material	No sign of steatosis, fibrosis, or ischemia; no sign of pathologic Kupffer cell proliferation	No sign of fibrosis or any other pathologic change	No sign of fibrosis or chronic inflammation. Cells are basophilic, cytoplasm is slightly granular; presence of mast cells.	No sign of fibrosis or chronic inflammation. Cells are basophilic, cytoplasm is slightly granular; presence of mast cells.
**I1**	**Liver**: Smaller and lighter than in DMSO. Light stripe on one lobe.<br>**Kidney**: larger than in I2 or DMSO, normal colour.<br>**Spleen**: normal size and colour, accumulation of adipose tissue around the capsule.<br>**Lungs**: normal size and colour<br>**Heart**: normal size and colour<br>**Pancreas**: normal size and colour<br>**Muscle**: normal colour	No sign of chronic congestion, fibrosis, or hemosiderin accumulation; well developed white and red pulp, no sign of presence of foreign material	No sign of steatosis, fibrosis, or ischemia; no sign of pathologic Kupffer cell proliferation	No sign of fibrosis or any other pathologic change	No sign of fibrosis or chronic inflammation. Cells are basophilic, cytoplasm is slightly granular; presence of mast cells.	No sign of fibrosis or chronic inflammation. Cells are basophilic, cytoplasm is slightly granular; presence of mast cells.
	**Liver**: smaller than in I1 and DMSO, normal colour.					

**I2**	**Kidneys**: Larger than in DMSO, normal colour; large haemorrhage in capsule.<br>**Spleen**: normal size and colour; adipose tissue accumulation around the capsule.<br>**Lungs**: normal size and colour<br>**Heart**: normal size and colour<br>**Pancreas**: normal size and colour<br>**Muscle**: normal colour	No sign of chronic congestion, fibrosis, or hemosiderin accumulation; well developed white and red pulp, no sign of presence of foreign material	No sign of steatosis, fibrosis, or ischemia; no sign of pathologic Kupffer cell proliferation	No sign of fibrosis or any other pathologic change	No sign of fibrosis or chronic inflammation. Cells are basophilic, cytoplasm is slightly granular; presence of mast cells.	No sign of fibrosis or chronic inflammation. Cells are basophilic, cytoplasm is slightly granular; presence of mast cells.
**Saline**	**Liver**: Normal size and colour<br>**Kidney**: Normal colour, larger size than in DMSO.<br>**Spleen**: Larger than in DMSO.<br>**Lungs**: two small haemorrhages.<br>**Heart**: Normal size, lighter than in Cy7 or DMSO<br>**Pankreas**: smaller than in DMSO.<br>**Muscle**: normal colour	No sign of chronic congestion, fibrosis, or hemosiderin accumulation; well developed white and red pulp, no sign of presence of foreign material	No sign of steatosis, fibrosis, or ischemia; no sign of pathologic Kupffer cell proliferation	No sign of fibrosis or any other pathologic change	No sign of fibrosis or chronic inflammation. Cells are basophilic, cytoplasm is slightly granular; presence of mast cells.	No sign of fibrosis or chronic inflammation. Cells are basophilic, cytoplasm is slightly granular; presence of mast cells.
**Cy7**	**Liver**: normal size, lighter than in DMSO<br>**Kidney**: normal size and colour<br>**Spleen**: larger than in DMSO, less fat.<br>**Lungs**: small haemorrhage. Small fibrotic granule<br>**Heart**: normal size and colour<br>**Pancreas**: normal size and colour<br>**Muscle**: normal colour	No sign of chronic congestion, fibrosis, or hemosiderin accumulation; well developed white and red pulp, no sign of presence of foreign material	No sign of steatosis, fibrosis, or ischemia; no sign of pathologic Kupffer cell proliferation	No sign of fibrosis or any other pathologic change	No sign of fibrosis or chronic inflammation. Cells are basophilic, cytoplasm is slightly granular; presence of mast cells.	No sign of fibrosis or chronic inflammation. Cells are basophilic, cytoplasm is slightly granular; presence of mast cells.
**DMSO**	**Liver**: used as reference for the rest of the samples<br>**Kidney**: used as reference for the rest of the samples<br>**Spleen**: used as reference for the rest of the samples<br>**Lungs**: used as reference for the rest of the samples<br>**Heart**: used as reference for the rest of the samples<br>**Pancreas**: used as reference for the rest of the samples<br>**Muscles**: used as reference for the rest of the samples	No sign of chronic congestion, fibrosis, or hemosiderin accumulation; well developed white and red pulp, no sign of presence of foreign material	No sign of steatosis, fibrosis, or ischemia; no sign of pathologic Kupffer cell proliferation	No sign of fibrosis or any other pathologic change	No sign of fibrosis or chronic inflammation. Cells are basophilic, cytoplasm is slightly granular; presence of mast cells.	No sign of fibrosis or chronic inflammation. Cells are basophilic, cytoplasm is slightly granular; presence of mast cells.

*Colour changes of liver, kidney, spleen, and heart were probably caused by anesthesia and autopsy (redistribution of blood in organs). Hemorrhages were probably caused by autopsy (*post mortem* changes).

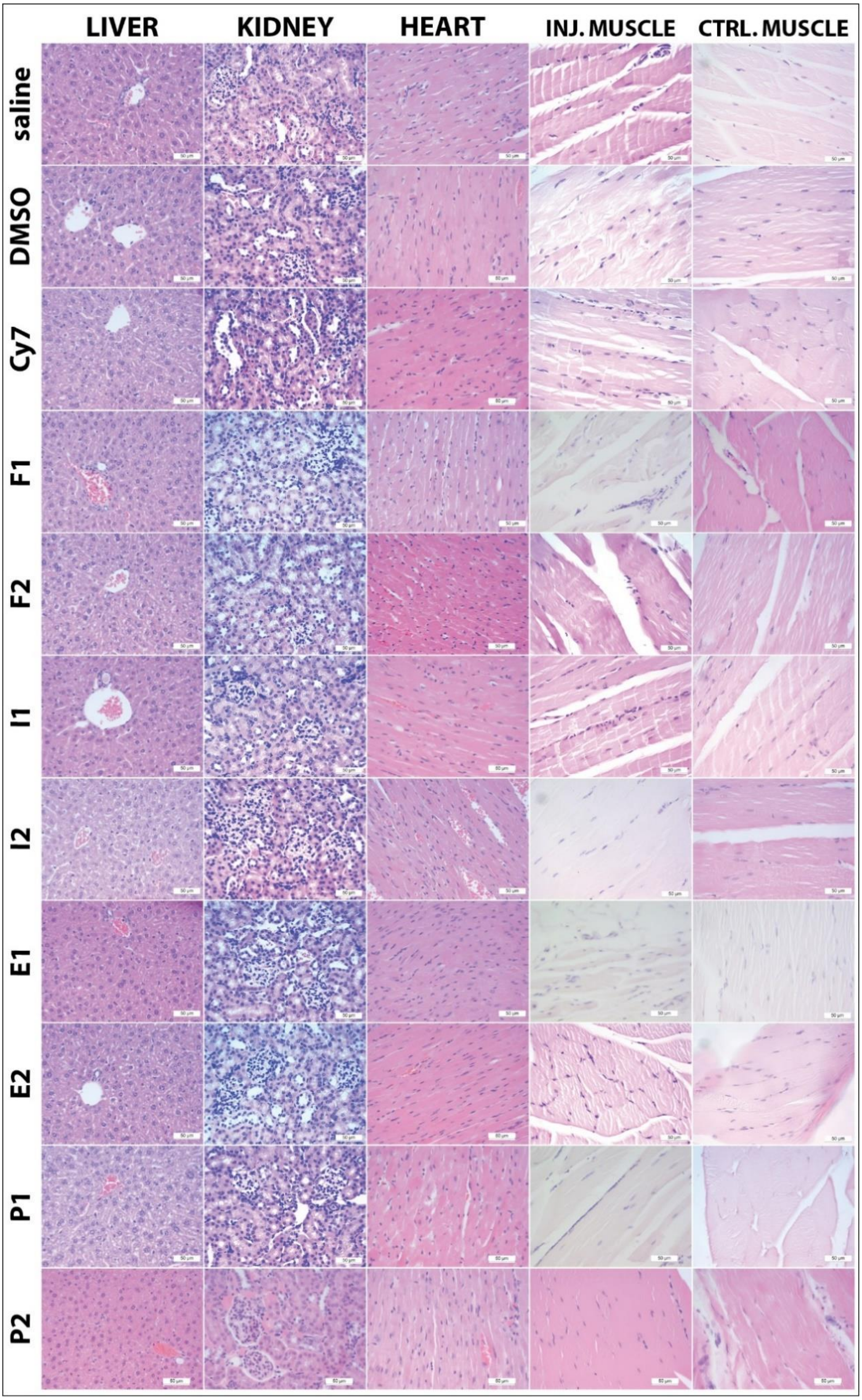

Figure S110. Histological examination of mice (202 days past administration), stained with H&E.

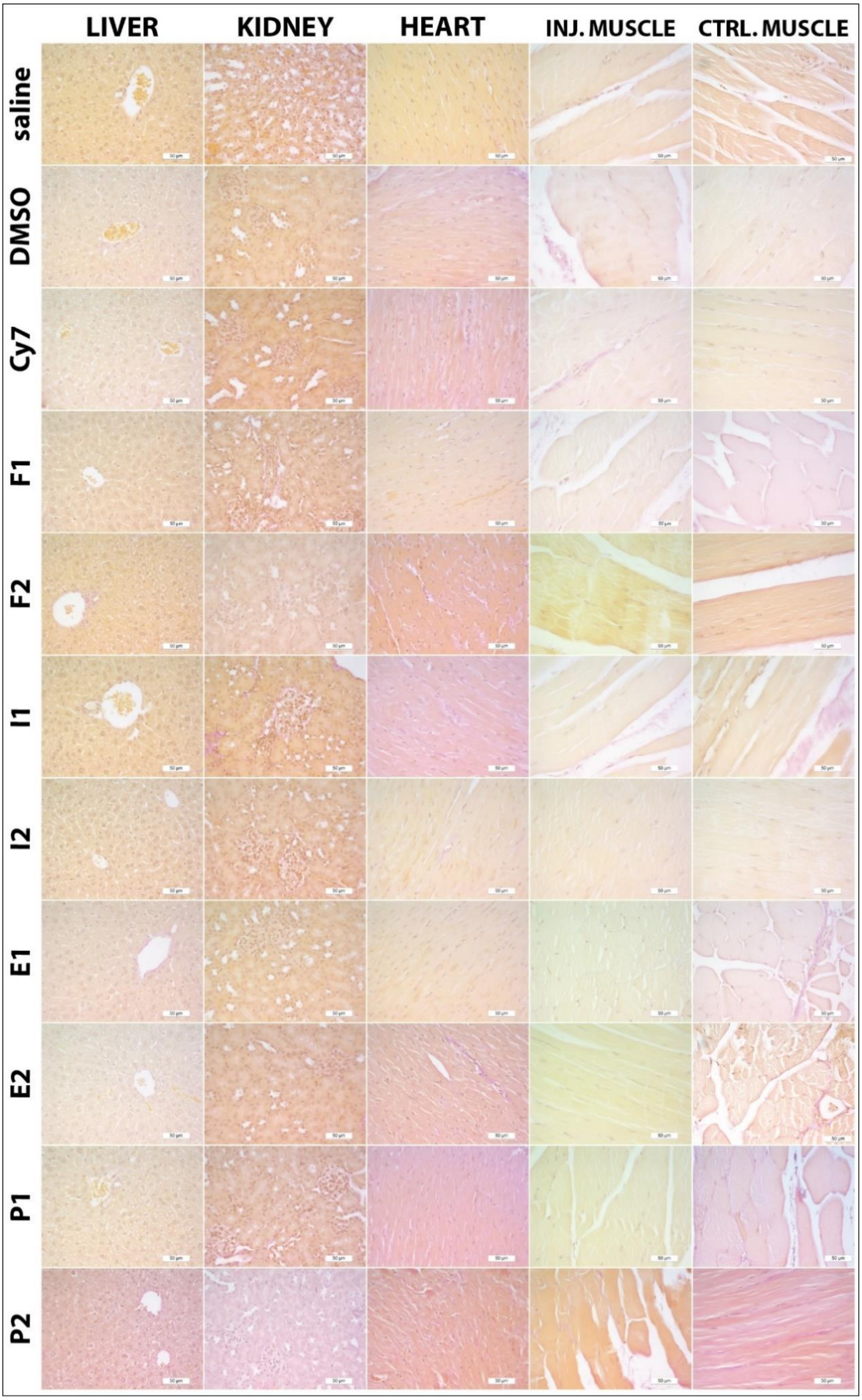

Figure S111. Histological examination of mice (202 days past administration), stained with WvG.

S16. *In vivo* parameters

S16.1. Mice weights

We recorded mice weights of mice, because sudden increase/decrease of weights or any major deviations from normal growth (typically retardation of weight gain) indicate pathology and/or discomfort to mice. However, we observed no systematic difference in feeding patterns or weight trends between the test groups or the control groups (**Figure S112**).

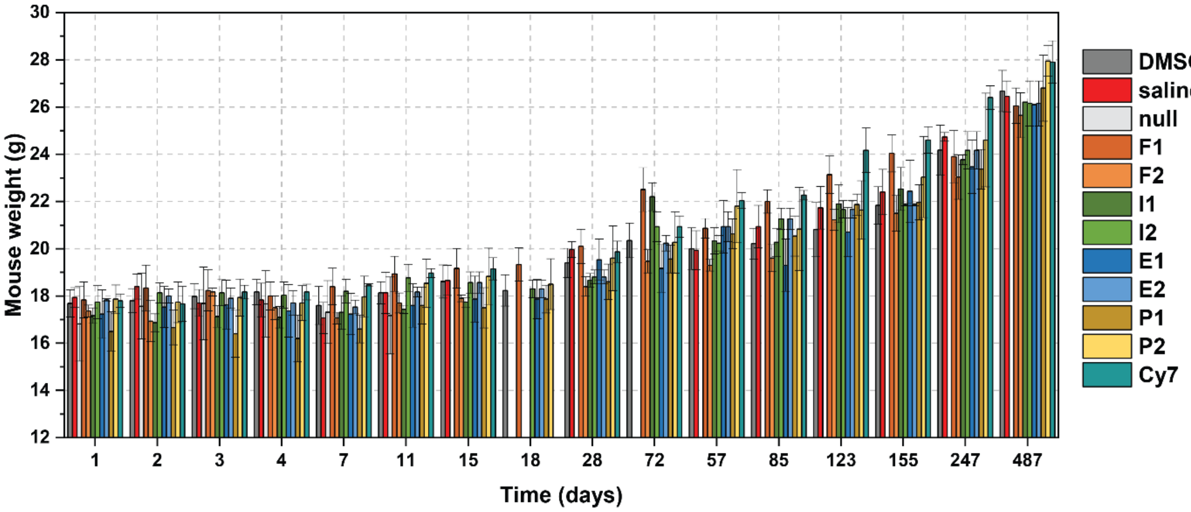

Figure S112. Weights of mice from all groups as a function of time

Table S94. Weight of mice from each group (**DMSO**, **saline**, **F1**, **F2**, **I1**, and **I2**), presented as mean and standard deviation (SD) in grams (g); continuation in **Table S95**.

	DMSO		saline		F1		F2		I1		I2	
**day**	**mean**	**SD**	**mean**	**SD**	**mean**	**SD**	**mean**	**SD**	**mean**	**SD**	**mean**	**SD**
**1**	17.68	0.58	17.93	0.42	17.83	0.76	17.37	0.25	17.17	0.33	17.73	0.70
**2**	17.80	0.51	18.40	0.54	18.33	0.97	16.93	0.87	16.87	0.38	18.13	0.42
**3**	17.98	0.52	17.70	0.57	18.23	0.87	18.17	0.17	17.13	0.45	18.13	0.56
**4**	18.17	0.53	17.83	0.71	18.00	0.59	17.50	0.51	17.10	0.45	18.03	0.56
**7**	17.60	0.81	17.07	0.66	18.40	0.78	17.07	0.25	17.30	0.71	18.20	0.51
**9**	N/D	N/D	N/D	N/D	N/D	N/D	N/D	N/D	N/D	N/D	N/D	N/D
**11**	18.13	0.47	18.13	0.87	18.93	0.74	17.70	0.42	17.43	0.17	18.77	0.57
**15**	18.61	0.70	18.66	0.64	19.17	0.84	17.90	0.14	17.73	0.21	18.57	0.45
**18**	18.23	0.66	N/D	N/D	19.33	0.71	N/D	N/D	N/D	N/D	18.30	0.37
**28**	19.40	0.62	19.97	0.34	20.10	0.73	18.40	0.41	18.67	0.29	18.80	0.29
**57**	20.00	0.88	19.93	0.83	20.87	0.42	19.30	0.24	20.33	0.56	20.23	0.33
**72**	20.40	0.73	N/D	N/D	22.50	0.93	19.50	0.49	22.2	0.59	20.9	0.63
**85**	20.22	0.65	20.93	0.90	22.00	0.50	19.60	0.57	20.27	0.60	21.27	0.45
**123**	20.80	1.14	21.70	0.90	23.10	0.79	21.20	0.45	21.9	0.8	21.7	0.37
**155**	21.80	0.79	22.40	0.96	24.00	0.78	21.50	0.75	22.5	0.91	21.9	0.05
**247**	24.18	1.06	24.73	0.19	23.90	1.10	23.03	0.94	23.77	0.21	24.17	0.79
**487**	26.68	0.87	26.45	0.65	26.05	0.75	25.65	0.95	26.2	0.00	26.15	0.95

Table S95. Weight of mice from each group (**E1**, **E2**, **P1**, **P2**, **Cy7**, and **naive group**), presented as mean and standard deviation (SD) in grams (g); continuation of **Table S94**.

	**E1**		**E2**		**P1**		**P2**		**Cy7**		**naive**	
**day**	**mean**	**SD**	**mean**	**SD**	**mean**	**SD**	**mean**	**SD**	**mean**	**SD**	**mean**	**SD**
**1**	17.23	1.03	17.83	0.05	16.50	0.83	17.87	0.60	17.80	0.28	16.82	1.58
**2**	17.53	0.87	18.00	0.29	16.67	0.74	17.73	0.87	17.67	0.74	17.57	1.39
**3**	17.63	1.03	17.90	0.43	16.40	0.99	17.93	0.78	18.17	0.29	17.68	1.54
**4**	17.37	1.11	17.70	0.51	16.20	0.99	17.70	0.73	18.17	0.33	17.67	1.41
**7**	17.23	0.87	17.53	0.29	16.60	0.59	17.97	0.87	18.47	0.05	17.32	1.33
**9**	N/D	N/D	N/D	N/D	N/D	N/D	N/D	N/D	N/D	N/D	17.63	1.22
**11**	17.60	0.92	18.17	0.21	17.60	0.78	18.53	1.02	18.97	0.19	17.18	1.63
**15**	17.87	0.97	18.57	0.45	17.50	0.85	18.83	1.19	19.15	0.47	N/D	N/D
**18**	17.87	0.83	18.30	0.37	17.87	0.59	18.50	1.07	N/D	N/D	N/D	N/D
**28**	19.53	0.88	18.80	0.29	18.60	0.75	19.60	1.37	19.87	0.45	N/D	N/D
**57**	19.17	1.01	20.23	0.33	19.57	0.59	20.27	1.30	20.93	0.45	N/D	N/D
**72**	20.90	1.11	20.90	0.63	20.60	0.63	21.80	1.55	22.00	0.34	N/D	N/D
**85**	19.30	1.10	21.27	0.45	20.53	0.85	20.83	1.76	22.27	0.21	N/D	N/D
**123**	20.70	1.04	21.70	0.37	21.90	0.45	21.60	1.24	24.20	0.95	N/D	N/D
**155**	22.40	1.31	21.90	0.05	22.00	0.74	23.00	1.72	24.60	0.57	N/D	N/D
**247**	23.47	1.13	24.17	0.79	23.37	0.84	24.60	1.98	26.40	0.50	N/D	N/D
**487**	26.1	0.00	26.15	0.95	26.8	1.40	27.95	0.65	27.9	0.90	N/D	N/D

S16.2. Observations of mice behavior after the polymer administration

One hour after the administration, most mice showed at least some level of agitation, discomfort to pain in the left hind leg. By the day 2, the mice exhibited no agitation or fear of researchers, but 36% (13/36) showed at least some decrease of strength in the administered leg and/or pain; 31% (4/13) of them showed a complete paralysis and limping. By the day 8, the 25% (9/36) of all mice showed some level of hind leg/foot weakness; the paralyzed mice showed a major recovery, all mice were able to use both their hind legs. By the day 11, only 3% (1/36) showed any limping and decreased strength in its hind feet. By the day 16, this mouse began to use their hind leg normally with normal grip force (**Figure S113**).

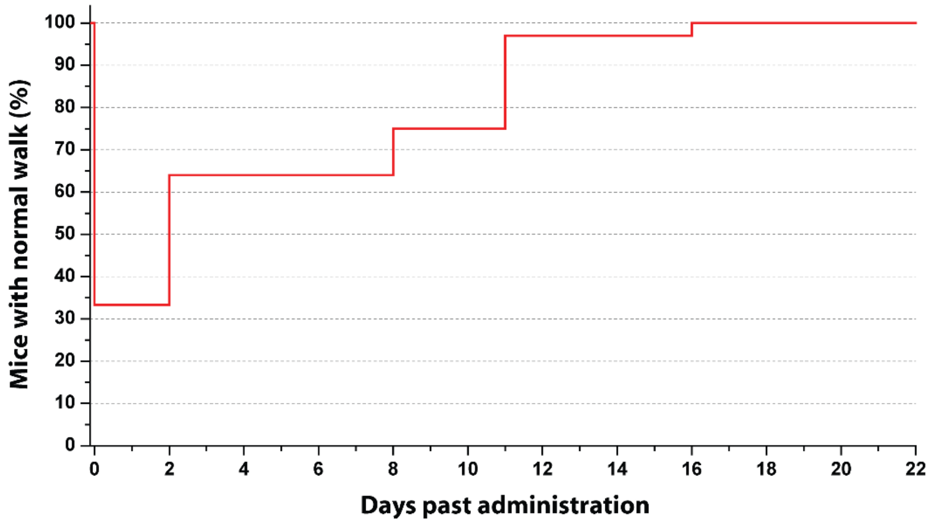

Figure S113. Percentage of mice with normal walking as a function of time.

Shortly after administrations, the urine and stool of our mice were strongly fluorescent, further corroborating that the polymers are excreted *via* kidneys and liver.[41,42]

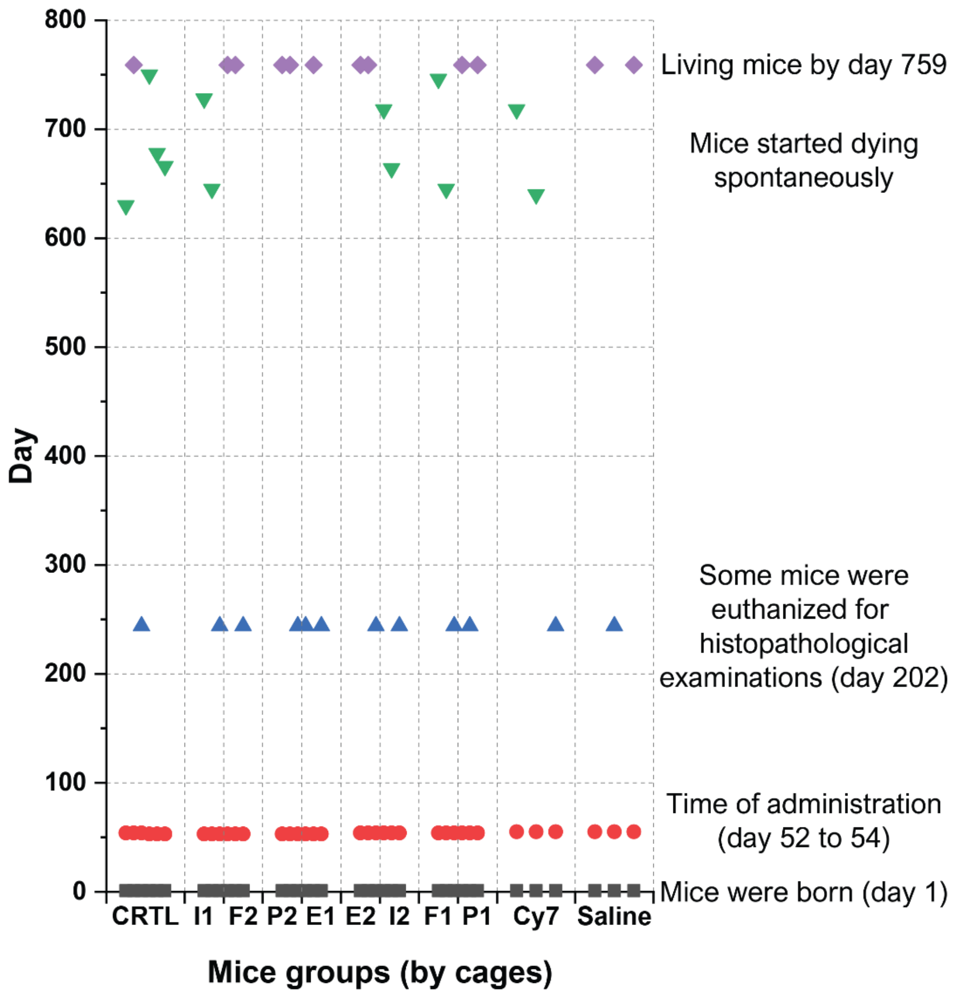

Figure S114. Life diagram of study mice. Some mice were euthanized on day 202 for histopathological examinations. The remaining mice were observed for following 557 days whether their lifespan would be affected by the polymer administration. Some mice died spontaneously, but a few mice were still alive by the end of the experiment. These data suggest that polymer administration did not shorten the life of mice (in comparison with control mice and with reported lifespans of BALB/c mice[50]), which further corroborates that our polymers were safe for mammals.

ADDITIONAL INFORMATION

S17. Applicability of Cy7 for long-term studies

Although Cy7 and its derivatives are commonly used fluorophores in medicine and biology research[12,13] with high chemical and photochemical stability,[51] their long-term *in vivo* stability remained unknown thus far. Because all our polymers have identical chemical bond between polymer and Cy7 trace, the rate of Cy7 degradation (due to hydrolysis or metabolism) was identical in all polymers. The fluorescence of **pDFEA** decayed with biological half-lives *ca*. 150 ± 49 days (**Table 2**, the polymer with longest half-life of all polymers). This biological half-life is in line with that of **pDFEA** copolymers in our previous study[21] (≈ 200 days in rats, determined by ^{19}F MRS), which shows that the rate of Cy7 degradation is slow to negligible. Therefore, the degradation of Cy7 affected the observed pharmacokinetics of our polymers only negligibly. Additionally, we can safely presume that the rate of fluorescence decay in **pAP**, **pDEA**, and **pNIPAM** (biological half-lives were *ca*. 15 to 60 days, **Table 2**) were only negligibly influenced by the degradation of Cy7 trace. As a result, we demonstrated that Cy7 tracers may be used in long term *in vivo* studies for tracking polymers (the biological half-life of Cy7 amine trace can be estimated to ≥ 150 days).

S18. Additional figures

S18.1. Formulas of dyes and labels

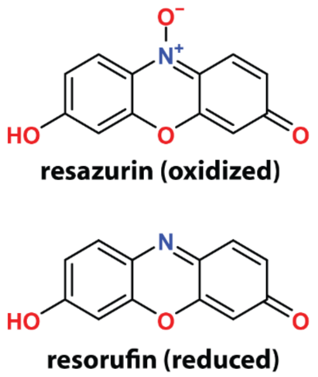

Figure S115. Formula of resazurin (in PrestoBlue™ Cell Viability Reagent) – oxidized and reduced form; CAS numbers 550-82-3 and 635-78-9

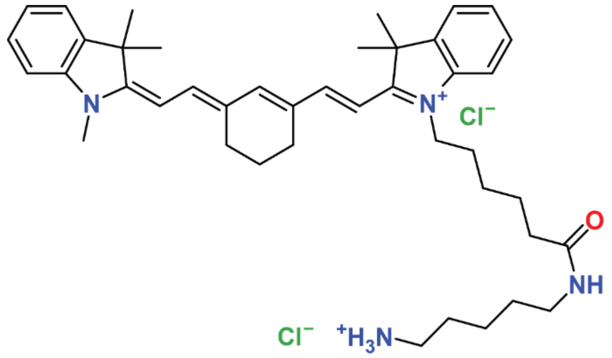

Figure S116. Formula of Cyanine7 amine dichloride (Cy7-amine), redrawn according to scheme provided by vendor (Lumiprobe)

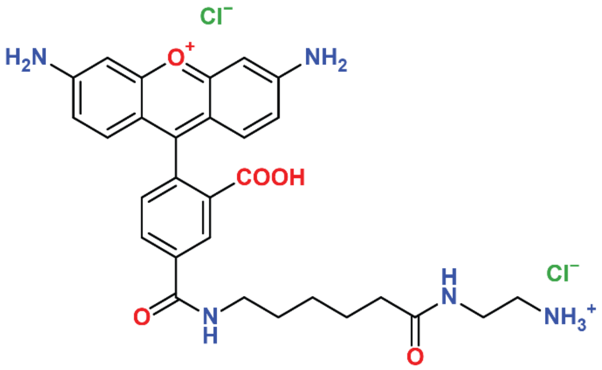

Figure S117. Formula of Dy505 aminoderivative dichloride (Dy505-amine), redrawn according to scheme provided by vendor (Dyomics)

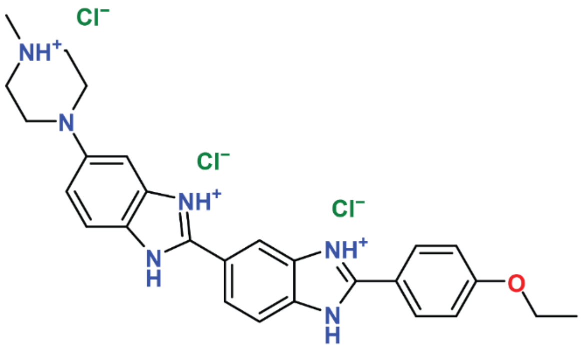

Figure S118. Formula of Hoechst 33342, redrawn according to scheme provided by vendor (Thermo Fisher Scientific), CAS number 875756-97-1

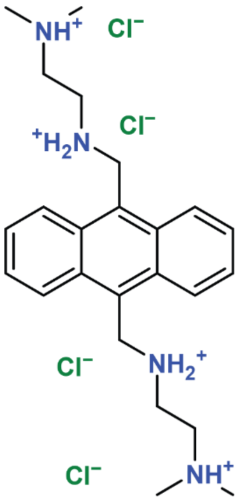

Figure S119. Formula of LysoTracker™ Blue DND-22, redrawn according to scheme provided by vendor (Thermo Fisher Scientific)

 DOI: XXXX.YYYY/ZZZZ

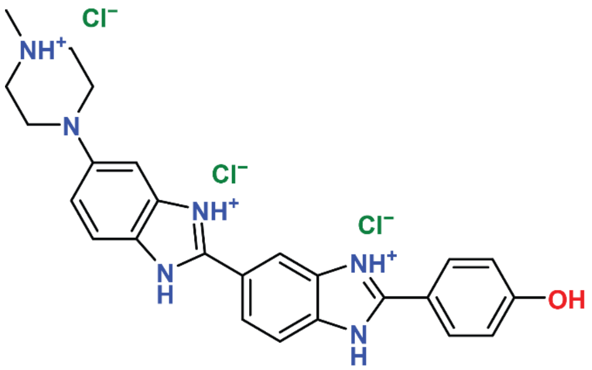

Figure S120. Formula of Hoechst 33258, redrawn according to scheme provided by vendor (Thermo Fisher Scientific), CAS number: 23491-45-4

S18.2. Moieties in the study polymers

As polyacrylamides, all four polymers act as hydrogen bond acceptors, but both **pDFEA** and **pNIPAM** can also act as hydrogen bond donors because they contain secondary amide moieties. In addition, **pDFEA** contains -CF_2H moieties, *i.e.*, lipophilic hydrogen bond donors[52] (see **Figure S121**).

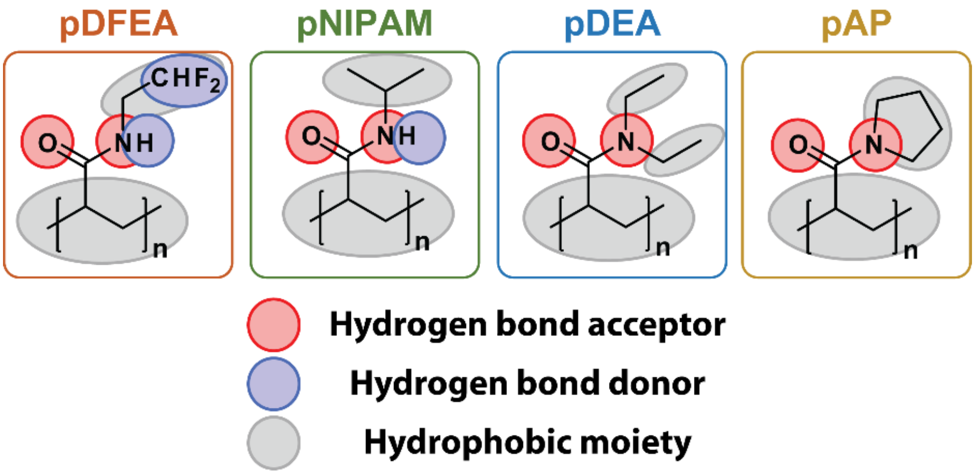

Figure S121. Hydrogen bond acceptors, donors, and hydrophobic moieties in the molecules.

S18.3. Properties of the study polymers

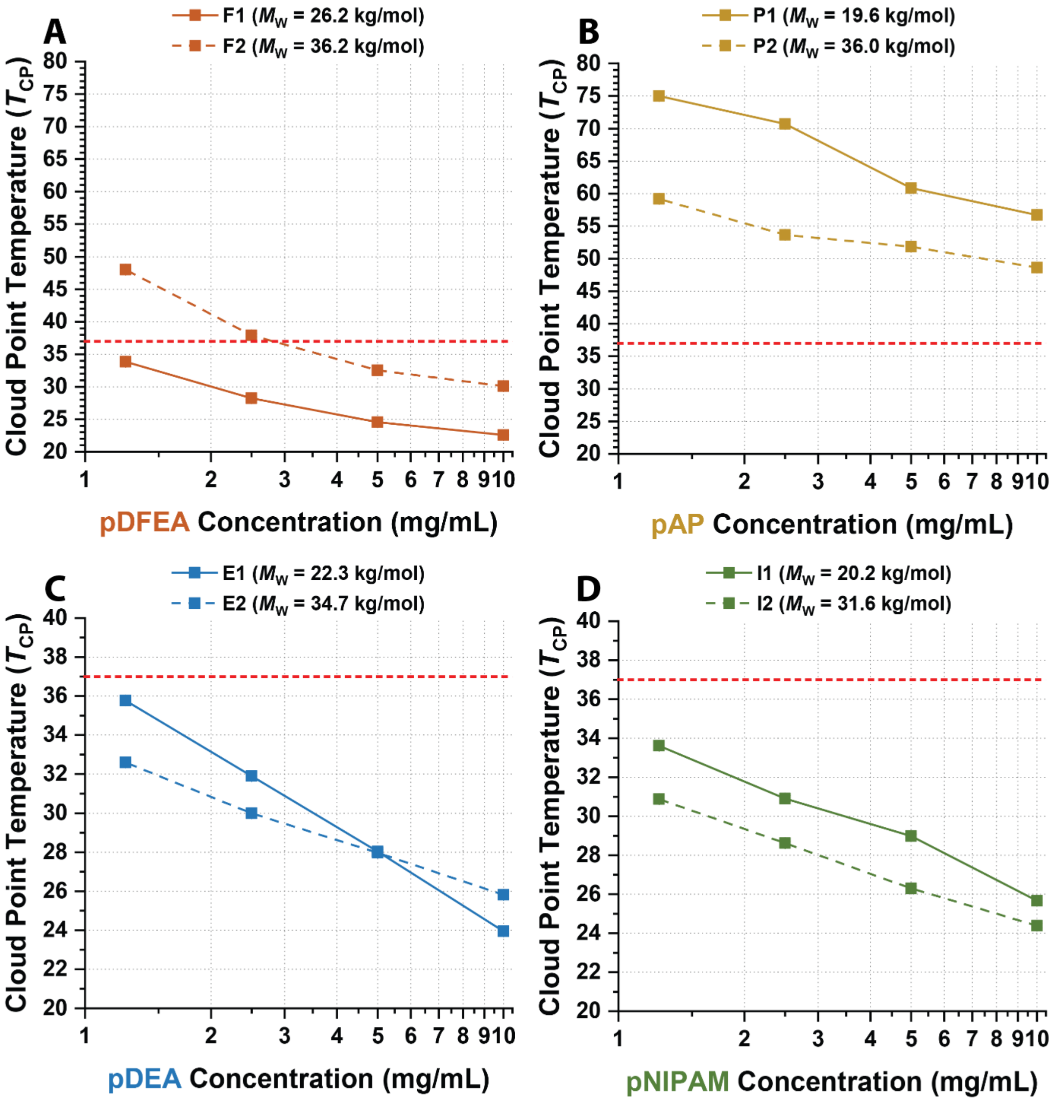

Figure S122. Plot of T_{CP} of the polymers **pDFEA** (**A**), **pAP** (**B**), **pNIPAM** (**C**) and **pDEA** (**D**) in FBS as a function of polymer concentrations. All T_{CP} are expressed as the mean of 6 measurement cycles ± standard deviation. Red dashed line indicates the body temperature (37 °C). Asterisks (*) indicate potential outliers. This figure was adapted from the literature.[1]

S18.4. Preparation of phantoms for confocal fluorescence microscopy

Confocal microscopes required phantoms to test the imaging quality. For this purpose, we mixed the polymer particles described in a previous article[53] (G-gel, 10.0 mg, 67 µmol epoxide moieties) and Cy7-amine (0.50 mg, 0.69 µmol) or Dy505-amine (0.20 mg, 0.32 µmol) in methanol (0.50 mL). The reaction mixture was stirred for 3 days at room temperature. Afterwards, we decanted off the supernatant and washed the particles with methanol, dried and used to optimize the detection parameters of the microscope for the given dye (**Figure S123**).

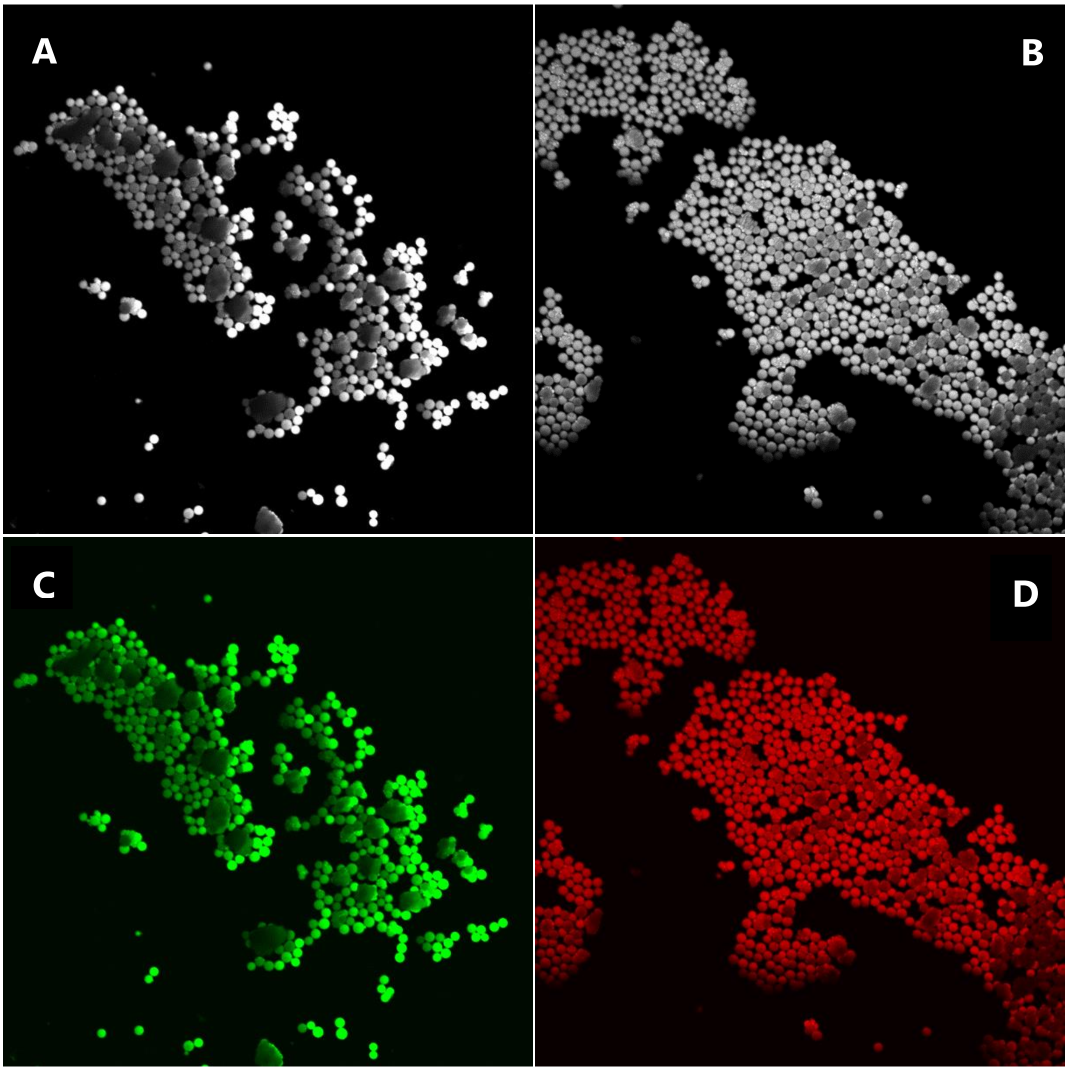

Figure S123: Phantoms (cross-linked (glycidyl)methacrylate beads, size 15 to 50 µm)[53], labelled with Dy505-amine, (**A**, **C**), and Cy7-amine (**B**, **D**), visualized with white light (**A**, **B**), λ_{ex} = 400 nm, λ_{em} = 505 nm (**C**), or λ_{ex} = 750 nm, λ_{em} = 775 nm (**D**). Magnified 200-fold. Dark spots are caused by larger clusters (that have lower excitation light penetration).

S18.5. Binning test

To verify the binning compensation by re-calculation, we acquired image of phantom object with various binning number (1, 2, 4 and 8) and evaluated the mean pixel value as a function of binning coefficient (binning number squared). The results (**Figure S124**) show that our setup enables the acquisition of image with different binning and to recalculate the average signal to a different level of binning without any distorsion.

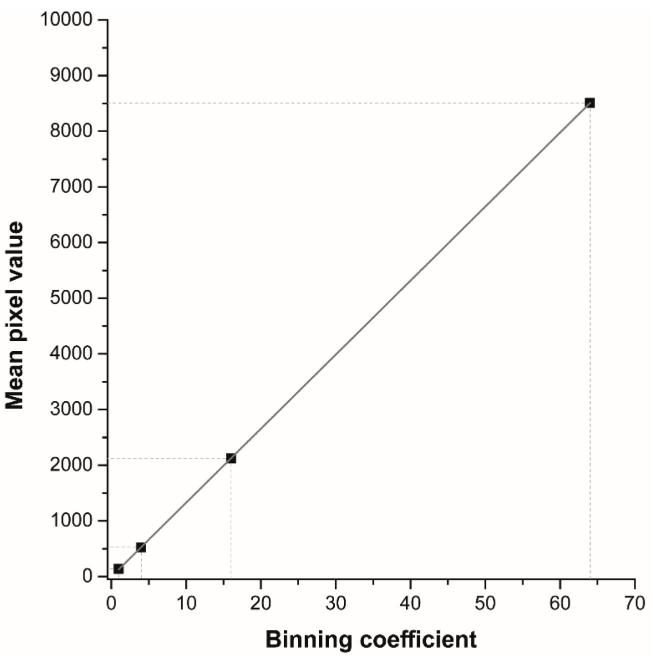

Figure S124. Mean pixel value of images acquired with various binning coefficients, fitted with $R^2 = 1.000$.

S18.6. Raw image from fluorescence imaging (Xtreme)

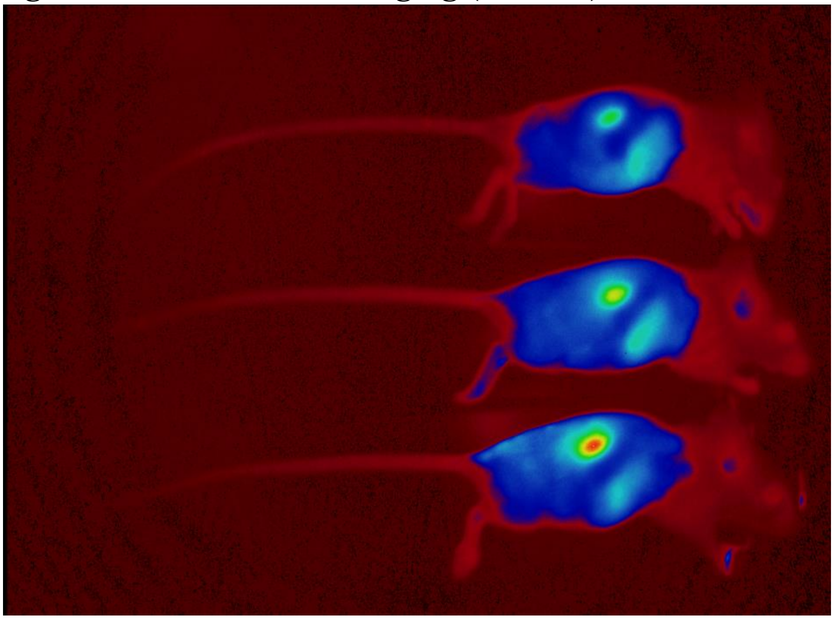

Figure S125. Mice from **P1** group, 48 hours past polymer administration, lying on their right side (opposite the site of administration); as an example of "raw image" from fluorescence imaging.

S18.7. Preparation of phosphate saline buffer (PBS)

For purposes of *in vitro* experiments, we prepared PBS solution enriched with magnesium and calcium ions. This PBS contained sodium chloride (NaCl; 138 mM), sodium phosphate dibasic (Na_2HPO_4; 15.2 mM), potassium chloride (KCl; 2.67 mM), potassium phosphate monobasic (KH_2PO_4; 1.47 mM), calcium chloride ($CaCl_2$, 0.90 mM), and magnesium chloride ($MgCl_2 \cdot 6H_2O$, 0.50 mM). This PBS was used only in *in vitro* experiments (*section S3.2.1*).

S18.8. Images from mice autopsy

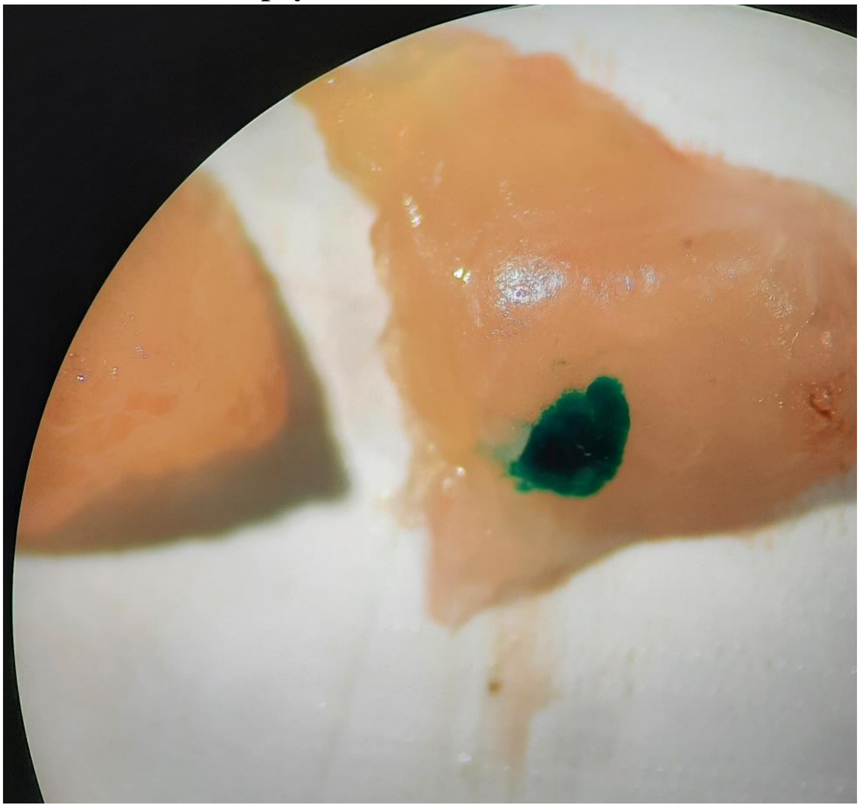

Figure S126. Intramuscular depot of polymer **F1**, 4 days after administration; magnified

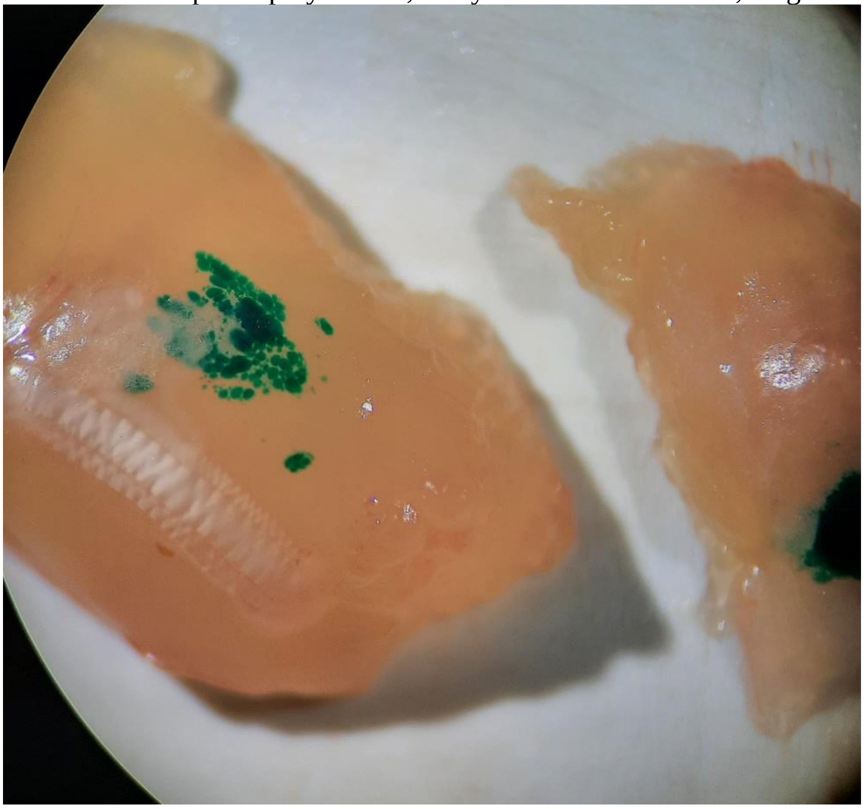

Figure S127. Intramuscular depot of polymer **F1**, 4 days after administration; magnified

S19. List of abbreviations

**Abbreviation**	**Explanation (base unit)**
^{1}H NMR	^{1}H nuclear magnetic resonance
^{1}H-^{13}C HSQC-edit NMR	^{1}H-^{13}C multiplicity edited, heteronuclear single quantum coherence spectroscopy nuclear magnetic resonance
^{13}C{^{1}H} NMR	^{13}C nuclear magnetic resonance with ^{1}H decoupling
a	intercept of a fitting linear curve ($f(x) = a + b \cdot x$)
$a_{I,\mathrm{IM}}$	intercept of linear fit of intensity-based dissolution of IM depot
$a_{I,\mathrm{KID}}$	intercept of linear fit of intensity-based dissolution of kidney depot
$a_{I,\mathrm{LIV}}$	intercept of linear fit of intensity-based dissolution of liver depot
$a_{S,\mathrm{IM}}$	intercept of linear fit of area-based dissolution of IM depot
$a_{V,\mathrm{IM}}$	intercept of linear fit of volume-based dissolution of IM depot
A	fraction of the administered polymer which enters the intracellular depot (and therefore enters **Phase 2a**)
au	arbitrary unit (also abbreviated as a.u.)
a.u.	arbitrary unit (also abbreviated as au)
ACVA	4,4'-[(E)-diazenediyl]bis(4-cyanopentanoic acid)
AIBN	2,2′-azobis(2-methylpropionitrile)
ANOVA	analysis of variance
b	slope of a fitting linear curve ($f(x) = a + b \cdot x$)
$b_{I,\mathrm{IM}}$	slope of linear fit of intensity-based dissolution of IM depot
$b_{I,\mathrm{KID}}$	slope of linear fit of intensity-based dissolution of kidney depot
$b_{I,\mathrm{LIV}}$	slope of linear fit of intensity-based dissolution of liver depot
$b_{S,\mathrm{IM}}$	slope of linear fit of area-based dissolution of IM depot
$b_{V,\mathrm{IM}}$	slope of linear fit of volume-based dissolution of IM depot
ca.	*circa*, approximately
c_{Ar}	concentration of argon
CI	confidence interval
Corp.	corporation; legal concept of a company in the USA
CPT	cloud point temperature
CTA	chain transfer agent
CA	California, state of the USA
Cy7-amine [hydrochloride]	1-(6-((5-ammoniopentyl)amino)-6-oxohexyl)-3,3-dimethyl-2-(2-(3-(2-(1,3,3-trimethylindolin-2-ylidene)ethylidene)cyclohex-1-en-1-yl)vinyl)-3*H*-indol-1-ium dichloride (**Figure S116**)
d*n*/d*c*	specific refractive index increment ($\mathrm{L \cdot g^{-1}}$)
DMEM	Dulbecco's Modified Eagle Medium
DMF	dimethylformamide
DMSO	dimethyl sulfoxide
DSC	differential scanning calorimetry
Dy505-amine	3,6-diamino-9-(4-((6-((2-ammonioethyl)amino)-6-oxohexyl) carbamoyl)-2-carboxyphenyl)xanthylium dichloride (**Figure S117**)
**E**	poly[(*N*,*N*-diethyl)acrylamide]
**E1**	poly[(*N*,*N*-diethyl)acrylamide], M_w = 22.3 kg/mol, $Đ_\mathrm{M}$ = 1.06
**E2**	poly[(*N*,*N*-diethyl)acrylamide], M_w = 34.7 kg/mol, $Đ_\mathrm{M}$ = 1.09
EU	European Union

**F**	poly[(*N*-2,2-difluoroethyl)acrylamide]
**F1**	poly[(*N*-2,2-difluoroethyl)acrylamide], M_w = 26.2 kg/mol, $Đ_M$ = 1.08
**F2**	poly[(*N*-2,2-difluoroethyl)acrylamide], M_w = 36.2 kg/mol, $Đ_M$ = 1.03
f_{KID}	polymer kidney accumulation factor<br>(a fraction of administered polymer that enters the kidney depot)
f_{LIV}	polymer kidney accumulation factor<br>(a fraction of administered polymer that enters the liver depot)
FBS	foetal bovine serum (fetal bovine serum in US English)
e.g.	*exempli gratia*, for example
GA UK	Grantová agentura UK (the Charles University Grant Agency)
GmbH	private limited company in legal structure of Germany or Austria<br>(german *Gesellschaft mit beschränkter Haftung*)
GPC	gel permeation chromatography
$H_{prot, low-temp, sol}$	enthalpy of interaction of polymer with serum proteins at temperature below T_{CP}, aggregation, adapted from literature[1]
$H_{prot, high-temp, sol}$	enthalpy of interaction of polymer with serum proteins at temperature above T_{CP}, aggregation, adapted from literature[1]
H&E	haematoxylin & eosin stain
HF	human fibroblasts
Hoechst 33342	2'-(4-ethoxyphenyl)-5-(4-methylpiperazin-1-ium-1-yl)-1*H*,1'*H*-[2,5'-bibenzo[d]imidazole]-3,3'-diium trichloride (**Figure S118**)
HPLC	high-performance liquid chromatography
HSQC	heteronuclear single quantum coherence spectroscopy
$\bar{I}'_{0,T}$	reduced tissue depot initial signal amplitudes
$\bar{I}'_{0,KID}$	reduced tissue depot kidney signal amplitudes
$\bar{I}'_{0,LIV}$	reduced tissue depot liver signal amplitudes
$\bar{I}'_{0,LIV}$	reduced tissue depot intramuscular signal amplitudes
I	intensity of fluorescence signal
I_+	total signal of pixels in the depot
$I_{+,0.9}$	the total intensity in the top 10% most intensive pixels (the intensity of top 0.9 percentile of all pixels above)
I_{IM}	intensity of fluorescence signal in intramuscular depot
$I_{0,KID}$	fluorescence intensity in KID depot, extrapolated to time zero
$I_{0,LIV}$	fluorescence intensity in LIV depot, extrapolated to time zero
$I_{0,IM}$	fluorescence intensity in IM depot, extrapolated to time zero
$I_{noise,IM}$	background noise value of fluorescence in intramuscular depot<br>(for Xtreme, *i.e.* for long-term monitoring)
$I_{noise,IM,PH}$	background noise value of fluorescence in intramuscular depot<br>(for photoacoustics, *i.e.* for short-term monitoring)
I_{KID}	intensity of fluorescence signal in kidney<br>(for Xtreme, *i.e.* for long-term monitoring)
I_{LIV}	intensity of fluorescence signal in liver<br>(for Xtreme, *i.e.* for long-term monitoring)
**I**	poly[(*N*-isopropyl)acrylamide]
**I1**	poly[(*N*-isopropyl)acrylamide], M_w = 20.2 kg/mol, $Đ_M$ = 1.03
**I2**	poly[(*N*-isopropyl)acrylamide], M_w = 31.6 kg/mol, $Đ_M$ = 1.03

i.e.	*id est*; that is
Inc.	incorporation; legal concept of company in the USA
IR	infrared light, subtype A (700 to 1400 nm)
ITC	isothermal titration calorimetry
J	spin-spin coupling constant (J-coupling constant)
K_{10}	distribution index (see *Section S5*)
k	kinetics constant
k_1	kinetics constant of polymer dissolution determined for mouse #1
k_2	kinetics constant of polymer dissolution determined for mouse #2
k_3	kinetics constant of polymer dissolution determined for mouse #3
$k_{I,\mathrm{IM}}$	kinetics constant of intensity-based dissolution of IM depot
$k_{I,\mathrm{KID}}$	kinetics constant of intensity-based dissolution of kidney depot
$k_{I,\mathrm{LIV}}$	kinetics constant of intensity-based dissolution of liver depot
$k_{S,\mathrm{IM}}$	kinetics constant of area-based dissolution of IM depot
$\bar{k}_T$	mean of dissolution kinetics constants
$k_{V,\mathrm{IM}}$	kinetics constant of volume-based dissolution of IM depot
k_{UP}	kinetics constant of polymer uptake by cells
k_{E}	kinetics constant of elimination of primary extracellular depot into bloodstream
LCST	lower critical solution temperature
LSCM	laser scanning confocal microscopy
Ltd.	Limited company; legal concept of company in the USA
Lyso®Tracker DND-22	N^1,$N^{1'}$-(anthracene-9,10-diylbis(methylene))bis(N^2,N^2-dimethylethane-1,2-diaminium) tetrachloride (**Figure S119**)
NMR	nuclear magnetic resonance
m_{x}	mass of compound x
M_n	number-average molar mass
M_t	midrange of biological half-lives
M_w	weight-average molecular weight
MA	Massachusetts, state of the USA
MALS	multiangle light scattering
MeOH	methanol
MeOH-4d	perdeuterated methanol
MRI	magnetic resonance imaging
n_{x}	molar amount of compound x (mol)
N	sample size; number of points
N_1	number of fitted points in mouse #1
N_2	number of fitted points in mouse #2
N_3	number of fitted points in mouse #3
$N_{i,T}$	number of fitted points
NA	numerical aperture
n/a	not applicable
N/A	not available
N/D	not determined
No.	number
NY	New York, state of the USA

norm.	normalized
p	*p*-value
**P**	poly[(*N*-acryloyl)pyrrolidine]
**P1**	poly[(*N*-acryloyl)pyrrolidine], M_w = 19.6 kg/mol, $Đ_M$ = 1.11
**P2**	poly[(*N*-acryloyl)pyrrolidine], M_w = 36.0 kg/mol, $Đ_M$ = 1.09
PAI	photoacoustic imaging
**pAP**	poly[(*N*-acryloyl)pyrrolidine
PBS	phosphate saline buffer
PCC	Pearson correlation coefficients
PDI	(poly)dispersity index ($Đ_M = M_n/M_w$)
**pDFEA**	poly[(*N*-2,2-difluoroethyl)acrylamide]
**pDEA**	poly[(*N*,*N*-diethyl)acrylamide]
**pNIPAM**	poly[(*N*-isopropyl)acrylamide]
$PrNH_2$	propylamine, propylamine
ppm	parts per million
PyBOP	benzotriazol-1-yloxytripyrrolidinophosphonium hexafluorophosphate
®	registered trademark (Lanham (Trademark) Act of 1946)
RI	refractive index
resazurin	7-hydroxy-3-oxo-3H-phenoxazine 10-oxide (**Figure S115**)
rMSC	rat mesenchymal stem cells
ROI	region of interest
rpm	revolutions per minute; rotations per minute
RAFT	reversible addition−fragmentation chain-transfer polymerization
S	depot area
s	standard deviation, usually fit-based (also abbreviated as SD)
$s_{k,1,T}$	standard deviation of fit for mouse #1
$s_{k,2,T}$	standard deviation of fit for mouse #2
$s_{k,3,T}$	standard deviation of fit for mouse #3
$s_{k,T}$	pooled standard deviations
S_{IM}	area (size) of intramuscular depot
$S_{0,\mathrm{IM}}$	a size of fluorescent intramuscular depot extrapolated to time zero
$S_{\mathrm{noise,IM}}$	average area of background noise in intramuscular depot
Sb.	legal code (Czech *Sbírka zákonů*)
SD	standard deviation
SEC	size exclusion chromatography
SPECT	single photon emission computer tomography
s.r.o.	private limited company in legal structure of the Czech Republic
t	triplet (in NMR)
T	tissue type (intramuscular (IM), kidney (KID), liver (LIV))
$t_{½}$	half-life
$\bar{t}_{1/2,T}$	mean biological half-life
$t_{½,\mathrm{I}}$	half-life of **intensity** dissolution of depot
$t_{½,\mathrm{S}}$	half-life of **depot area** dissolution of depot
$t_{½,\mathrm{V}}$	half-life of **volume** dissolution of depot
$t_{½,\mathrm{I,KID}}$	half-life of **intensity** dissolution of kidney depot
$t_{½,\mathrm{I,LIV}}$	half-life of **intensity** dissolution of liver depot

$t_{\mathrm{I,\ max}}$	time (range) of maximum **intensity** of the depot signal
$t_{\mathrm{S,\ max}}$	time (range) of maximum **depot area**
$t_{\mathrm{V,\ max}}$	time (range) of maximum **depot volume**
T_{CP}	cloud point temperature
TCEP	tris(2-carboxyethyl)phosphine hydrochloride
TEA	triethylamine
TGC	time gain compensation
™	unregistered trademark symbol (Lanham Act of 1946)
UCST	upper critical solution temperature
UK	United Kingdom of Great Britain and Northern Ireland
US	ultrasound
US-PAI	ultrasound-photoacoustic imaging
USA	United States of America
UV	ultraviolet [light]
UV/VIS	ultraviolet/visible light
V	volume
V_{IM}	volume of intramuscular depot
$V_{0,\mathrm{IM}}$	volume of fluorescence in IM depot, extrapolated to time zero
$V_{\mathrm{noise,IM}}$	volume of background noise of fluorescence in intramuscular depot
V_{x}	volume of compound x
v/v	volume fraction, volume per volume
WA	Washington, state of the USA
WG	Weigert van Gieson stain (also known as Van Gieson stain)
$\bar{x}$	mean value of quantity x
z	confidence level value
$\Delta H_{\mathrm{aag,\ neat}}$	enthalpy change for the aggregation
δ	chemical shift (ppm)
λ	wavelength (m^{-1})
$\sigma_{\bar{I}_{0,T}}$	standard deviations of $\bar{I}_{0,T}$
$\sigma_{\bar{t}_{1/2,T}}$	standard deviations of $\bar{t}_{1/2,T}$
$Đ_{\mathrm{M}}$	dispersity index, defined as $M_{\mathrm{w}}/M_{\mathrm{n}}$
*	asterisk
‡	dagger (double dagger)

S20. Authors Contributions

**Beneš, Hynek (H.B.)**	Investigation, Resources, Methodology, Validation
**Beneš, Jiří (J.B.)**	Resources, Funding acquisition
**Dalecká, Linda (L.D.)**	Investigation, Methodology
**Dunlop, David (D.D.)**	Formal Analysis, Investigation, Validation, Data curation, Software, Methodology, Writing – review & editing
**Groborz, Ondřej (O.G.)**	Conceptualization, Supervision, Methodology, Data curation, Investigation, Formal analysis, Visualization, Project administration, Writing – original draft, Writing – review & editing, Funding acquisition, Resources, Software
**Hoogenboom, Richard (R.H.)**	Validation, Methodology, Resources, Funding acquisition, Supervision
**Hovořáková, Mária (M.Ho.)**	Investigation, Methodology, Data curation
**Hrubý, Martin (M.Hr.)**	Resources, Validation, Funding acquisition, Writing – review & editing, Supervision
**Kadlec, Jan (J.K.)**	Methodology, Data curation, Investigation, Formal analysis, Visualization, Software
**Keša, Peter (P.K.)**	Investigation, Methodology, Data curation, Validation, Formal analysis
**Kolouchová, Kristýna (K.K.)**	Investigation, Methodology, Data curation, Writing – review & editing, Project administration, Funding acquisition
**Krunclová, Tereza (T.K.)**	Investigation, Methodology, Data curation, Validation, Visualization, Formal analysis, Writing – review & editing
**Loukotová, Lenka (LL)**	Validation, Writing – review & editing
**Matouš, Petr (P.M)**	Investigation, Methodology
**Melo, Carlos V. (C.V.M)**	Methodology, Data curation, Validation, Writing – review & editing
**Pankrác, Jan (J.P.)**	Investigation, Methodology, Data curation, Validation, Formal analysis, Writing – review & editing
**Páral, Petr (P.P.)**	Investigation, Methodology
**Pavlíková, Zuzana (Z.P.)**	Investigation, Methodology
**Pierzynová, Aneta (A.P.)**	Investigation, Methodology, Data curation, Writing – review & editing
**Slanina, Tomáš (T.S.)**	Validation, Methodology
**Šefc, Luděk (L.Še.)**	Resources, Funding acquisition
**Šrámek, Jaromír (J.Š.)**	Investigation, Methodology, Data curation
**Švec, Pavel (P.Š.)**	Validation, Writing – review & editing
**Štěpánek, Lubomír (L.Št.)**	Formal Analysis, Investigation, Validation, Data curation, Software, Methodology
**Vlierberghe, Sandra van** (S.v.V.)	Resources, Funding acquisition, Validation, Methodology

Project design and administration: O.G. conceived the presented ideas, supervised and administrated the project. K.K., O.G., R.H., M.Hr., M.Ho., J.P., P.K., and P.Š. were involved in methodology design. O.G., K.K., R.H., J.P., D.D., M.Ho., L.Šv., S.v.V. and C.V.M. have proposed the study strategies, validated data, and formulated the conclusions of the study. M.Hr., R.H., O.G., T.K., J.P., P.K., J.B., L.Še. S.v.V., and K.K. have secured the resources for this study, M.Hr., O.G., J.B., L.Š., and R.H. have secured its funding. O.G. and K.K., and C.V.M. wrote major parts of the manuscript, R.H., P.Š, S.v.V., M.Hr., J.P., P.K., and L.L. wrote and/ or reviewed parts of the manuscript.

Polymers*:* K.K., O.G. synthesized, modified, purified, and characterized the polymers (SEC, NMR). K.K. and O.G. assessed polymers' purity and stability. H.B. assessed and evaluated the calorimetric experiments.

***In vivo* experiments**: J.P., P.K., P.P., O.G., and P.M. performed the *in vivo* biological experiments. O.G., J.P., and P.M. prepared mice for the experiments, P.K., P.M. and P.P. injected the polymer into mice. P.K. monitored and P.M. evaluated the long-term photoacoustic imaging and evaluated corresponding data. J.P. monitored mice's weights and acquired the long-term fluorescence imaging signal *in vivo*.

Pharmacokinetics model: J.P. and P.K measured the pharmacokinetics data; J.P., P.P., J.K, and O.G. evaluated and curated the data (J.P. and P.M. visualized the data). O.G. and J.K. derived the pharmacological models. O.G., J.K., D.D. and L.Š. evaluated the pharmacokinetics and validated data.

Data processing: J.P., P.K. and P.M. acquired and evaluated the biological data. J.P., J.K., and O.G. processed these data and obtained mice depot descriptors. O.G. and D.D. fitted the data, D.D., L.Št., and O.G. performed statistical analyses and O.G., D.D., J.K., J.P., R.H., and L.Št. formulated the conclusions of these analyses.

***In vivo* histopathology:** A.P., L.D., and J.Š. have assessed and evaluated the histological examinations.

***Ex vivo* biodistribution:** M.Ho., L.D. and O.G. designed the *ex vivo* experiment. P.M., O.G., and P.P. prepared the mice and injected them with polymers, M.Ho, L.D., and Z.P. processed the tissues, assessed and evaluated the *ex vivo* biodistributions.

***In vitro* cellular model:** T.K. assessed and evaluated *in vitro* cytotoxicity on cell lines and assessed the cellular biodistribution.

Graphics: O.G. designed and prepared most figures in manuscript and ESI (with help from other co-authors).

S21. Ethic code

All experiments and the subsequent data evaluation were performed according to ethic codes of First Faculty of Medicine, Charles University and Czech Academy of Sciences and the valid legislative of the Czech Republic as of 2019 to 2022.

We avoided referencing journals that were listed in Beall's list or journals with score 0 in Norwegian Register for Scientific Journals, Series and Publishers.[54]

REFERENCES

(1) Kolouchová, K.; Lobaz, V.; Beneš, H.; Rosa, V. R. de la; Babuka, D.; Švec, P.; Černoch, P.; Hrubý, M.; Hoogenboom, R.; Štěpánek, P.; Groborz, O. Thermoresponsive Properties of Polyacrylamides in Physiological Solutions. *Polym. Chem.* **2021**. https://doi.org/10.1039/d1py00843a.

(2) Qiu, X.-P.; Winnik, F. M. Facile and Efficient One-Pot Transformation of RAFT Polymer End Groups via a Mild Aminolysis/Michael Addition Sequence. *Macromol. Rapid Commun.* **2006**, *27* (19), 1648–1653. https://doi.org/10.1002/marc.200600436.

(3) Azagarsamy, M. A.; Anseth, K. S. Bioorthogonal Click Chemistry: An Indispensable Tool to Create Multifaceted Cell Culture Scaffolds. *ACS Macro Lett.* **2013**, *2* (1), 5–9. https://doi.org/10.1021/mz300585q.

(4) Korthals, B.; Morant-Miñana, M. C.; Schmid, M.; Mecking, S. Functionalization of Polymer Nanoparticles by Thiol−Ene Addition. *Macromolecules* **2010**, *43* (19), 8071–8078. https://doi.org/10.1021/ma100966w.

(5) Chiefari, J.; Chong, Y. K. (Bill); Ercole, F.; Krstina, J.; Jeffery, J.; Le, T. P. T.; Mayadunne, R. T. A.; Meijs, G. F.; Moad, C. L.; Moad, G.; Rizzardo, E.; Thang, S. H. Living Free-Radical Polymerization by Reversible Addition−Fragmentation Chain Transfer: The RAFT Process. *Macromolecules* **1998**, *31* (16), 5559–5562. https://doi.org/10.1021/ma9804951.

(6) McNaught, A. D.; Wilkinson, A. *IUPAC. Compendium of Chemical Terminology*, 2nd ed.; Blackwell Scientific Publications: Oxford, 1997.

(7) Zhou, C.; Hillmyer, M. A.; Lodge, T. P. Micellization and Micellar Aggregation of Poly(Ethylene-*Alt*-Propylene)-*b*-Poly(Ethylene Oxide)-*b*-Poly(*N*-Isopropylacrylamide) Triblock Terpolymers in Water. *Macromolecules* **2011**, *44* (6), 1635–1641. https://doi.org/10.1021/ma102786q.

(8) Burns, J. A.; Butler, J. C.; Moran, J.; Whitesides, G. M. Selective Reduction of Disulfides by Tris(2-Carboxyethyl)Phosphine. *J. Org. Chem.* **1991**, *56* (8), 2648–2650. https://doi.org/10.1021/jo00008a014.

(9) Van Durme, K.; Van Assche, G.; Van Mele, B. Kinetics of Demixing and Remixing in Poly(*N*-Isopropylacrylamide)/Water Studied by Modulated Temperature DSC. *Macromolecules* **2004**, *37* (25), 9596–9605. https://doi.org/10.1021/ma048472b.

(10) Zhao, J.; Hoogenboom, R.; Van Assche, G.; Van Mele, B. Demixing and Remixing Kinetics of Poly(2-Isopropyl-2-Oxazoline) (PIPOZ) Aqueous Solutions Studied by Modulated Temperature Differential Scanning Calorimetry. *Macromolecules* **2010**, *43* (16), 6853–6860. https://doi.org/10.1021/ma1012368.

(11) Aleksandrova, R.; Philipp, M.; Müller, U.; Riobóo, R. J.; Ostermeyer, M.; Sanctuary, R.; Müller-Buschbaum, P.; Krüger, J. K. Phase Instability and Molecular Kinetics Provoked by Repeated Crossing of the Demixing Transition of PNIPAM Solutions. *Langmuir* **2014**, *30* (39), 11792–11801. https://doi.org/10.1021/la5026763.

(12) Alander, J. T.; Kaartinen, I.; Laakso, A.; Pätilä, T.; Spillmann, T.; Tuchin, V. V.; Venermo, M.; Välisuo, P. A Review of Indocyanine Green Fluorescent Imaging in Surgery. *Int. J. Biomed. Imaging* **2012**, *2012*, e940585. https://doi.org/10.1155/2012/940585.

(13) Reinhart, M. B.; Huntington, C. R.; Blair, L. J.; Heniford, B. T.; Augenstein, V. A. Indocyanine Green: Historical Context, Current Applications, and Future Considerations. *Surg Innov* **2016**, *23* (2), 166–175. https://doi.org/10.1177/1553350615604053.

(14) Rao, J.; Dragulescu-Andrasi, A.; Yao, H. Fluorescence Imaging in Vivo: Recent Advances. *Current Opinion in Biotechnology* **2007**, *18* (1), 17–25. https://doi.org/10.1016/j.copbio.2007.01.003.

(15) Shi, C.; Wu, J. B.; Pan, D. Review on Near-Infrared Heptamethine Cyanine Dyes as Theranostic Agents for Tumor Imaging, Targeting, and Photodynamic Therapy. *JBO* **2016**, *21* (5), 050901. https://doi.org/10.1117/1.JBO.21.5.050901.

(16) Luo, S.; Zhang, E.; Su, Y.; Cheng, T.; Shi, C. A Review of NIR Dyes in Cancer Targeting and Imaging. *Biomaterials* **2011**, *32* (29), 7127–7138. https://doi.org/10.1016/j.biomaterials.2011.06.024.

(17) Frangioni, J. V. In Vivo Near-Infrared Fluorescence Imaging. *Current Opinion in Chemical Biology* **2003**, *7* (5), 626–634. https://doi.org/10.1016/j.cbpa.2003.08.007.

(18) International Organization for Standardization. *Biological Evaluation of Medical Devices — Part 5: Tests for in Vitro Cytotoxicity (ISO 10993-5:2009)*; International Organisation for Standardization, 2009.

(19) Bak, J. M.; Kim, K.-B.; Lee, J.-E.; Park, Y.; Yoon, S. S.; Jeong, H. M.; Lee, H. Thermoresponsive Fluorinated Polyacrylamides with Low Cytotoxicity. *Polym. Chem.* **2013**, *4* (7), 2219–2223. https://doi.org/10.1039/C2PY20747H.

(20) Naha, P. C.; Bhattacharya, K.; Tenuta, T.; Dawson, K. A.; Lynch, I.; Gracia, A.; Lyng, F. M.; Byrne, H. J. Intracellular Localisation, Geno- and Cytotoxic Response of PolyN-Isopropylacrylamide (PNIPAM) Nanoparticles to Human Keratinocyte (HaCaT) and Colon Cells (SW 480). *Toxicol. Lett.* **2010**, *198* (2), 134–143. https://doi.org/10.1016/j.toxlet.2010.06.011.

(21) Kolouchova, K.; Jirak, D.; Groborz, O.; Sedlacek, O.; Ziolkowska, N.; Vit, M.; Sticova, E.; Galisova, A.; Svec, P.; Trousil, J.; Hajek, M.; Hruby, M. Implant-Forming Polymeric ^{19}F MRI-Tracer with Tunable Dissolution. *J. Control. Release* **2020**, *327*, 50–60. https://doi.org/10.1016/j.jconrel.2020.07.026.

(22) Kelava, T. Biological Actions of Drug Solvents. *Period biol* **2011**, *113* (3), 10.

(23) Yamauchi, H.; Maeda, Y. LCST and UCST Behavior of Poly(*N* -Isopropylacrylamide) in DMSO/Water Mixed Solvents Studied by IR and Micro-Raman Spectroscopy. *J. Phys. Chem. B* **2007**, *111* (45), 12964–12968. https://doi.org/10.1021/jp072438s.

(24) Costa, R. O. R.; Freitas, R. F. S. Phase Behavior of Poly(*N*-Isopropylacrylamide) in Binary Aqueous Solutions. *Polymer* **2002**, *43* (22), 5879–5885. https://doi.org/10.1016/S0032-3861(02)00507-4.

(25) Marquardt, R. [Treatment of dry eye with a new gel in eyedrop form]. *Klin Monbl Augenheilkd* **1986**, *189* (1), 51–54. https://doi.org/10.1055/s-2008-1050750.

(26) Xiao, Q.; Hu, Y.; Chen, F.; Chen, X. A Comparative Assessment of the Efficacy of Carbomer Gel and Carboxymethyl Cellulose Containing Artificial Tears in Dry Eyes. *J Huazhong Univ Sci Technolog Med Sci* **2008**, *28* (5), 592–595. https://doi.org/10.1007/s11596-008-0523-9.

(27) Anderson, D.; Burnham, K. Model Selection and Multi-Model Inference. *Second. NY: Springer-Verlag* **2004**, *63* (2020), 10.

(28) Taylor, J. R. *An Introduction to Error Analysis: The Study of Uncertainties in Physical Measurements*, 2nd ed.; University Science Books.

(29) Bevington, P. R.; Robinson, D. K. *Data Reduction and Error Analysis for the Physical Sciences*, 3rd ed.; McGraw-Hill, 2002.

(30) Kluyver, T.; Ragan-Kelley, B.; Pérez, F.; Granger, B.; Bussonnier, M.; Frederic, J.; Kelley, K.; Hamrick, J.; Grout, J.; Corlay, S.; Ivanov, P.; Avila, D.; Abdalla, S.; Willing, C. Jupyter Notebooks – a Publishing Format for Reproducible Computational Workflows. In *Positioning and Power in Academic Publishing: Players, Agents and Agendas*; Loizides, F., Schmidt, B., Eds.; IOS Press, 2016; pp 87–90.

(31) Hunter, J. D. Matplotlib: A 2D Graphics Environment. *Computing in Science Engineering* **2007**, *9* (3), 90–95. https://doi.org/10.1109/MCSE.2007.55.

(32) Harris, C. R.; Millman, K. J.; van der Walt, S. J.; Gommers, R.; Virtanen, P.; Cournapeau, D.; Wieser, E.; Taylor, J.; Berg, S.; Smith, N. J.; Kern, R.; Picus, M.; Hoyer, S.; van Kerkwijk, M. H.; Brett, M.; Haldane, A.; del Río, J. F.; Wiebe, M.; Peterson, P.; Gérard-Marchant, P.; Sheppard, K.; Reddy, T.; Weckesser, W.; Abbasi, H.; Gohlke, C.; Oliphant, T. E. Array Programming with NumPy. *Nature* **2020**, *585* (7825), 357–362. https://doi.org/10.1038/s41586-020-2649-2.

(33) Reback, J.; McKinney, W.; jbrockmendel; Bossche, J. V. den; Augspurger, T.; Cloud, P.; gfyoung; Sinhrks; Klein, A.; Roeschke, M.; Hawkins, S.; Tratner, J.; She, C.; Ayd, W.; Petersen, T.; Garcia, M.; Schendel, J.; Hayden, A.; MomIsBestFriend; Jancauskas, V.; Battiston, P.; Seabold, S.; chris-b1; h-vetinari; Hoyer, S.; Overmeire, W.; alimcmaster1; Dong, K.; Whelan, C.; Mehyar, M. Pandas-Dev/Pandas: Pandas 1.0.3, 2020. https://doi.org/10.5281/zenodo.3715232.

(34) Collette, A. *Python and HDF5*; O'Reilly, 2013.

(35) Virtanen, P.; Gommers, R.; Oliphant, T. E.; Haberland, M.; Reddy, T.; Cournapeau, D.; Burovski, E.; Peterson, P.; Weckesser, W.; Bright, J.; van der Walt, S. J.; Brett, M.; Wilson, J.; Millman, K. J.; Mayorov, N.; Nelson, A. R. J.; Jones, E.; Kern, R.; Larson, E.; Carey, C. J.; Polat, İ.; Feng, Y.; Moore, E. W.; VanderPlas, J.; Laxalde, D.; Perktold, J.; Cimrman, R.; Henriksen, I.; Quintero, E. A.; Harris, C. R.; Archibald, A. M.; Ribeiro, A. H.; Pedregosa, F.; van Mulbregt, P.; SciPy 1.0 Contributors. SciPy 1.0: Fundamental Algorithms for Scientific Computing in Python. *Nature Methods* **2020**, *17*, 261–272. https://doi.org/10.1038/s41592-019-0686-2.

(36) Seabold, S.; Perktold, J. Statsmodels: Econometric and Statistical Modeling with Python. In *9th Python in Science Conference*; 2010.

(37) Waskom, M. L. Seaborn: Statistical Data Visualization. *Journal of Open Source Software* **2021**, *6* (60), 3021. https://doi.org/10.21105/joss.03021.

(38) Clark, A. Pillow (PIL Fork) Documentation, 2015.

(39) Bertrand, N.; Fleischer, J. G.; Wasan, K. M.; Leroux, J.-C. Pharmacokinetics and Biodistribution of N-Isopropylacrylamide Copolymers for the Design of PH-Sensitive Liposomes. *Biomaterials* **2009**, *30* (13), 2598–2605. https://doi.org/10.1016/j.biomaterials.2008.12.082.

(40) Wyffels, L.; Verbrugghen, T.; Monnery, B. D.; Glassner, M.; Stroobants, S.; Hoogenboom, R.; Staelens, S. MPET Imaging of the Pharmacokinetic Behavior of Medium and High Molar Mass ^{89}Zr-Labeled Poly(2-Ethyl-2-Oxazoline) in Comparison to Poly(Ethylene Glycol). *J. Control. Release* **2016**, *235*, 63–71. https://doi.org/10.1016/j.jconrel.2016.05.048.

(41) Kučka, J.; Hrubý, M.; Lebeda, O. Biodistribution of a Radiolabelled Thermoresponsive Polymer in Mice. *Appl. Radiat. Isot.* **2010**, *68* (6), 1073–1078. https://doi.org/10.1016/j.apradiso.2010.01.022.

(42) Seymour, L. W.; Duncan, R.; Strohalm, J.; Kopeček, J. Effect of Molecular Weight (M_w) of *N*-(2-Hydroxypropyl)Methacrylamide Copolymers on Body Distribution and Rate of Excretion after Subcutaneous, Intraperitoneal, and Intravenous Administration to Rats. *J. Biomed. Mater* **1987**, *21* (11), 1341–1358. https://doi.org/10.1002/jbm.820211106.

(43) Yamaoka, T.; Tabata, Y.; Ikada, Y. Fate of Water-Soluble Polymers Administered via Different Routes. *J. Pharm. Sci.* **1995**, *84* (3), 349–354. https://doi.org/10.1002/jps.2600840316.

(44) Yamaoka, T.; Tabata, Y.; Ikada, Y. Body Distribution Profile of Polysaccharides after Intravenous Administration. *Drug Delivery* **1993**, *1* (1), 75–82. https://doi.org/10.3109/10717549309031345.

(45) Glassner, M.; Palmieri, L.; Monnery, B. D.; Verbrugghen, T.; Deleye, S.; Stroobants, S.; Staelens, S.; wyffels, L.; Hoogenboom, R. The Label Matters: MPET Imaging of the Biodistribution of Low Molar Mass ^{89}Zr and ^{18}F-Labeled Poly(2-Ethyl-2-Oxazoline). *Biomacromolecules* **2017**, *18* (1), 96–102. https://doi.org/10.1021/acs.biomac.6b01392.

(46) Akimoto, J.; Nakayama, M.; Sakai, K.; Okano, T. Temperature-Induced Intracellular Uptake of Thermoresponsive Polymeric Micelles. *Biomacromolecules* **2009**, *10* (6), 1331–1336. https://doi.org/10.1021/bm900032r.

(47) Novy, Z.; Lobaz, V.; Vlk, M.; Kozempel, J.; Stepanek, P.; Popper, M.; Vrbkova, J.; Hajduch, M.; Hruby, M.; Petrik, M. Head-To-Head Comparison of Biological Behavior of Biocompatible Polymers Poly(Ethylene Oxide), Poly(2-Ethyl-2-Oxazoline) and Poly[N-(2-Hydroxypropyl)Methacrylamide] as Coating Materials for Hydroxyapatite Nanoparticles in Animal Solid Tumor Model. *Nanomaterials* **2020**, *10* (9), 1690. https://doi.org/10.3390/nano10091690.

(48) Maxie, M. G.; Jubb, K. V. F. *Jubb, Kennedy, and Palmer's Pathology of Domestic Animals*; 2016.

(49) Tsang, A. P.; Fujiwara, Y.; Hom, D. B.; Orkin, S. H. Failure of Megakaryopoiesis and Arrested Erythropoiesis in Mice Lacking the GATA-1 Transcriptional Cofactor FOG. *Genes Dev.* **1998**, *12* (8), 1176–1188.

(50) Goodrick, C. L. Life-Span and the Inheritance of Longevity of Inbred Mice. *Journal of Gerontology* **1975**, *30* (3), 257–263. https://doi.org/10.1093/geronj/30.3.257.

(51) Weinstain, R.; Slanina, T.; Kand, D.; Klán, P. Visible-to-NIR-Light Activated Release: From Small Molecules to Nanomaterials. *Chem. Rev.* **2020**. https://doi.org/10.1021/acs.chemrev.0c00663.

(52) Zafrani, Y.; Yeffet, D.; Sod-Moriah, G.; Berliner, A.; Amir, D.; Marciano, D.; Gershonov, E.; Saphier, S. Difluoromethyl Bioisostere: Examining the “Lipophilic Hydrogen Bond Donor” Concept. *J. Med. Chem.* **2017**, *60* (2), 797–804. https://doi.org/10.1021/acs.jmedchem.6b01691.

(53) Groborz, O.; Poláková, L.; Kolouchová, K.; Švec, P.; Loukotová, L.; Miriyala, V. M.; Francová, P.; Kučka, J.; Krijt, J.; Páral, P.; Báječný, M.; Heizer, T.; Pohl, R.; Dunlop, D.; Czernek, J.; Šefc, L.; Beneš, J.; Štěpánek, P.; Hobza, P.; Hrubý, M. Chelating Polymers for Hereditary Hemochromatosis Treatment. *Macromol. Biosci.* **2020**, *20* (12), 2000254. https://doi.org/10.1002/mabi.202000254.

(54) The Norwegian Ministry of Education and Research. *Norwegian Register for Scientific Journals, Series and Publishers*. https://kanalregister.hkdir.no/publiseringskanaler/Forside.

(55) Ioannidis, J. P. A. Why Most Published Research Findings Are False. *PLOS Medicine* **2005**, *2* (8), e124. https://doi.org/10.1371/journal.pmed.0020124.

(56) Schoenfeld, J. D.; Ioannidis, J. P. Is Everything We Eat Associated with Cancer? A Systematic Cookbook Review. *The American Journal of Clinical Nutrition* **2013**, *97* (1), 127–134. https://doi.org/10.3945/ajcn.112.047142.

(57) Miyakawa, T. No Raw Data, No Science: Another Possible Source of the Reproducibility Crisis. *Molecular Brain* **2020**, *13* (1), 24. https://doi.org/10.1186/s13041-020-0552-2.

(58) Schooler, J. W. Metascience Could Rescue the ‘Replication Crisis.’ *Nature* **2014**, *515* (7525), 9–9. https://doi.org/10.1038/515009a.

(59) Ioannidis, J. P. A.; Fanelli, D.; Dunne, D. D.; Goodman, S. N. Meta-Research: Evaluation and Improvement of Research Methods and Practices. *PLOS Biology* **2015**, *13* (10), e1002264. https://doi.org/10.1371/journal.pbio.1002264.

(60) Groborz, O. Pharmacokinetics of Intramuscularly Administered Thermoresponsive Polyacrylamides. Diplomová práce (Master thesis), Department of Organic Chemistry, Faculty of Science, Charles University, Prague, 2021.

ATTACHED FILES

Facing the current reproducibility crisis in science,[55,56] we decided to provide as much data (both raw and processed) in the ESI as possible to improve reproducibility of this study.[57–59] Several additional can be found on the website of the journal.

S22. Attachments

S22.1. Video reconstruction of intramuscular depots (PAI-US)

We provide a few examples of video reconstruction of PAI-US IM polymer depots.

Table S96. List of all files in archive **Photoacoustic_Videos.rar** (762,024 kiB)

File name	Polymer	Mouse	Time of acquisition after polymer injection
M5 E1 0h.mp4	**E1**	M2	ca. 5 minutes
M5 E1 1w.mp4	**E1**	M2	1 week
M5 E1 2w.mp4	**E1**	M2	2 weeks
M5 E1 1M.mp4	**E1**	M2	1 month
M5 E1 2M.mp4	**E1**	M2	2 months
M5 E1 3M.mp4	**E1**	M2	3 months
M5 E1 4M.mp4	**E1**	M2	4 months
M5 E1 5M.mp4	**E1**	M2	5 months
M5 E1 6M.mp4	**E1**	M2	6 months
M5 E1 8M.mp4	**E1**	M2	8 months
M2 F1 0h.mp4	**F1**	M2	ca. 5 minutes
M2 F1 1w.mp4	**F1**	M2	1 week
M2 F1 2w.mp4	**F1**	M2	2 weeks
M2 F1 1M.mp4	**F1**	M2	1 month
M2 F1 2M.mp4	**F1**	M2	2 months
M2 F1 3M.mp4	**F1**	M2	3 months
M2 F1 4M.mp4	**F1**	M2	4 months
M2 F1 5M.mp4	**F1**	M2	5 months
M2 F1 6M.mp4	**F1**	M2	6 months
M2 F1 8M.mp4	**F1**	M2	8 months
M6 F2 0h.mp4	**F2**	M3	ca. 5 minutes
M6 F2 1w.mp4	**F2**	M3	1 week
M6 F2 2w.mp4	**F2**	M3	2 weeks
M6 F2 1M.mp4	**F2**	M3	1 month
M6 F2 2M.mp4	**F2**	M3	2 months
M6 F2 3M.mp4	**F2**	M3	3 months
M6 F2 4M.mp4	**F2**	M3	4 months
M6 F2 5M.mp4	**F2**	M3	5 months
M6 F2 6M.mp4	**F2**	M3	6 months
M6 F2 8M.mp4	**F2**	M3	8 months
M2 I1 0h.mp4	**I1**	M2	ca. 5 minutes
M2 I1 1w.mp4	**I1**	M2	1 week
M2 I1 2w.mp4	**I1**	M2	2 weeks
M2 I1 1M.mp4	**I1**	M2	1 month
M2 I1 2M.mp4	**I1**	M2	2 months
M2 I1 3M.mp4	**I1**	M2	3 months
M2 I1 4M.mp4	**I1**	M2	4 months
M2 I1 5M.mp4	**I1**	M2	5 months
M2 I1 6M.mp4	**I1**	M2	6 months
M2 I1 8M.mp4	**I1**	M2	8 months
M6 P1 0h.mp4	**P1**	M3	ca. 5 minutes
M6 P1 1w.mp4	**P1**	M3	1 week
M6 P1 2w.mp4	**P1**	M3	2 weeks
M6 P1 1M.mp4	**P1**	M3	1 month
M6 P1 2M.mp4	**P1**	M3	2 months
M6 P1 3M.mp4	**P1**	M3	3 months
M6 P1 4M.mp4	**P1**	M3	4 months
M6 P1 5M.mp4	**P1**	M3	5 months
M6 P1 6M.mp4	**P1**	M3	6 months
M6 P1 8M.mp4	**P1**	M3	8 months

S22.2. NMR files

Archive **NMR-export.rar** (7,223 kiB) contains both raw ^{1}H NMR (Bruker Topspin 3.6.1 format) and processed (in MestReNova *.mnova file and as images) of all polymers. Additionally, we provide processed and analyzed ^{13}C and multiplicity-edited ^{1}H-^{13}C heteronuclear single quantum coherence spectra (images) for peak matching.

S22.3. Size exclusion chromatograms

Archive **SEC-export.rar** (6,543 kiB) contains both raw (in Origin file, *.opj, and Excel file, *.xlsx) and processed size-exclusion chromatograms (images) of all polymers (both before and after labelling with Dy505-amine or Cy7-amine).

S22.4. Fluorescence imaging

Archive **Fluorescence_imaging_(long_term).rar** (179,143 kiB) contains processed data of kidney and liver depots. Additionally, the archive contains both raw (*.tif files) and processed (Excel file, *.xlsx) of intramuscular depots and their descriptors.
